# Genome-Wide Identification, Classification and Expression Analysis of the MYB Transcription Factor Family in *Petunia*

**DOI:** 10.3390/ijms22094838

**Published:** 2021-05-03

**Authors:** Guanqun Chen, Weizhi He, Xiangxin Guo, Junsong Pan

**Affiliations:** 1 School of Design, Shanghai Jiao Tong University, Shanghai 200240, China; hwz849179102@sjtu.edu.cn (W.H.); July1026@sjtu.edu.cn (X.G.); 2 School of Agriculture and Biology, Shanghai Jiao Tong University, Shanghai 200240, China

**Keywords:** MYB family, *Petunia hybrida*, transcription factor, gene expression, anthocyanin biosynthesis

## Abstract

A lot of researches have been focused on the evolution and function of MYB transcription factors (TFs). For revealing the formation of petunia flower color diversity, MYB gene family in petunia was identified and analyzed. In this study, a total of 155 MYB genes, including 40 1R-MYBs, 106 R2R3-MYBs, 7 R1R2R3-MYBs and 2 4R-MYBs, have been identified in the *Petunia axillaris* genome. Most R2R3 genes contain three exons and two introns, whereas the number of PaMYB introns varies from 0 to 12. The R2R3-MYB members could be divided into 28 subgroups. Analysis of gene structure and protein motifs revealed that members within the same subgroup presented similar exon/intron and motif organization, further supporting the results of phylogenetic analysis. Genes in subgroup 10, 11 and 21 were mainly expressed in petal, not in vegetative tissues. Genes in subgroup 9, 19, 25 and 27 expressed in all tissues, but the expression patterns of each gene were different. According to the promoter analysis, five R2R3-MYB and two MYB-related genes contained MBSI cis-element, which was involved in flavonoid biosynthetic regulation. *PaMYB100/DPL* has been reported to positively regulate to pigmentation. However, although *PaMYB82*, *PaMYB68* and *Pa1RMYB36* contained MBSI cis-element, their function in flavonoid biosynthesis has not been revealed. Consistent with existing knowledge, PaMYBs in subgroup 11 had similar function to AtMYBs in subgroup 6, genes in which played an important role in anthocyanin biosynthesis. In addition, *PaMYB1* and *PaMYB40* belonged to P9 (S7) and were potentially involved in regulation of flavonoid synthesis in petunia vegetative organs. This work provides a comprehensive understanding of the MYB gene family in petunia and lays a significant foundation for future studies on the function and evolution of MYB genes in petunia.

## 1. Introduction

As one of the largest transcription factor (TFs) families in plants, MYB genes play an important role in plant biology, including development, environmental adaptation and metabolic regulation [1,2,3]. The first plant MYB-encoding gene was isolated from maize (*Zea mays*), named as COLORED1 (C1), indicating its function in anthocyanin biosynthesis [4]. Subsequently, more and more MYB genes were identified in plants, and the MYB superfamily is dramatically expanded in plants [5]. Currently, 22,032 MYB and 15,369 MYB-related sequences are available in the plant TF database (http://planttfdb.gao-lab.org/, accessed on 1 February 2021) [6].

MYB transcription factor harbors a conserved MYB DNA binding domain (DBD) at the N terminus and a diverse C-terminal modulator region responsible for the regulatory activity of the protein. The MYB domain frequently consists of up to four imperfect repeats, namely 1R-MYB (MYB-related), R2R3-MYB, R1R2R3-MYB and 4R-MYB based on the number of MYB repeats [7]. MYB subfamily containing two repeats, characterized by R2R3-type MYB proteins, is the largest within the MYB family in plants [8,9]. In general, each repeat possesses 50–55 amino acids residues, each forming three α-helices structures. The helix-turn-helix (HTH) structure constructed by the second and third helices plays an important role in DNA binding [10,11]. In *Arabidopsis thaliana* was identified 198 MYB genes in genome, containing 126 of R2R3-MYB proteins [5,12]. Soybean has over 700 predicted MYB proteins and 244 R2R3-MYB proteins [13]. On the contrary, the number of 4R-MYB is the least, and the function of them is largely unknown. Single-repeat MYB proteins have been identified in plants and animals in increasing numbers, and the majority of single-repeat MYB genes have been characterized in plants [14].

R2R3-MYB proteins are involved in an extensive diversification of functions, especially for regulation of secondary metabolism and abiotic stresses [1,3]. Anthocyanins are colored products of the flavonoid biosynthetic pathway, produced in response to a range of developmental and environmental signals [15]. In vegetative tissues, they are frequently produced in response to stress. R2R3-MYB proteins directly bind to the promoters of structural genes or to genes encoding bHLH regulators and activate their gene expression. For instance, two *MYB* genes, *VvMYBA1* and *VvMYBA2*, control the anthocyanin biosynthesis through regulating *VvUFGT* expression in grapevine [16,17]. Similarly, *PAP1* and *PAP2* in *Arabidopsis* [18,19], *MdMYB10* in apple [20] and *FvMYB10* in strawberry [21] are positive regulators to anthocyanin biosynthesis. On the contrary, others are served as anthocyanin biosynthesis repressors, such as *FaMYB1* in strawberry [22] and *AtMYB4* and *AtMYB3* in *Arabidopsis* [23,24]. R2R3-MYB proteins were further classified into 25 subgroups according to evolutionary analysis in *Arabidopsis* [5]. Each subgroup shares similar functions, but there are exceptions. In subgroup 7, *AtMYB12* and *AtMYB111* controlled flavanol biosynthesis in seedlings [25]. In subgroup 11, *AtMYB41* and *AtMYB102* contributed to plant resistance against wounding and osmotic stress [26,27]. In subgroup 20, *AtMYB2* was induced by dehydration and salt stress [28], while *AtMYB62* is reported to be involved in phosphate starvation [29].

*Petunia* is one of the classic model systems for investigating anthocyanin biosynthesis and regulation, including floral pigmentation [30] and anthocyanin in vegetative tissues (stems, leaves and sepals) in response to stressors such as high light [31]. Several MYB proteins have been characterized in petunia, such as positive regulators of *AN2*, *AN4*, *DPL, PHZ* and *ASR* [32,33,34,35], negative regulators of *PhMYB27* and *PhMYBx* [36]. The whole-genome sequencing and assembly of two wild parents of petunia, *P. axillaris* and *P. inflata* [37], bring significant benefits to identify and characterize MYB genes on a genome-wide scale. However, lots of MYB proteins have still not yet been identified. Considering the multiple functions of MYB genes, especially their important roles in anthocyanin biosynthesis, research was conducted concerning the evolution and expression properties of the MYB TF family in petunia. Firstly, we performed a genome-wide identification of MYB genes in *Petunia*. Then, we carried out the phylogenetic relationship of PaMYB and AtMYB proteins, the genomic structure, and other structural features. The cis-elements of PaMYBs was predicted and analyzed for transcriptional regulation. Based on the above analysis, the genes related to anthocyanin biosynthesis were identified and quantified. Our research may serve as a foundation for future research into the functional roles of petunia MYB genes.

## 2. Results

### 2.1. Identification of PaMYBs in Petunia

Two-way BLAST method was performed to identify *PaMYBs* using sequences of *Arabidopsis* MYB genes as reference. Approximately 168 candidate genes were isolated. All putative genes were subsequently verified in the SMART (Simple Modular Architecture Research Tool) and Pfam databases to confirm the existence and number of the MYB DNA binding domain (DBD) domains. A total of 155 PaMYBs were identified. Based on the number of MYB DBD domain, 155 PaMYB proteins contained 106 R2R3-MYB proteins, 7 R1R2R3-MYB proteins, 2 4R-MYB proteins and 40 MYB-related proteins. The coding sequence, amino acid sequence, molecular weights (MWs), isoelectric point (PI) and subcellular localizations of all *PaMYB* genes are shown in supplementary Tables S1 and S2. Peaxi162Scf00110g00004 was the smallest protein with 73 amino acids, because it obtained only one DBD domain. Peaxi162Scf00523g00004 was the largest protein with 1051 amino acids. Consequently, the protein MWs varied from 8.63 kDa (Peaxi162Scf00110g00004) to 116.60 kDa (Peaxi162Scf00523g00004), and the pIs ranged from 4.66 (Peaxi162Scf00944g00140) to 10.02 (Peaxi162Scf00118g00310). The subcellular localization of all the PaMYB proteins was the nucleus, indicating that PaMYBs were nucleoproteins. The sequence similarity between PaMYBs and AtMYBs ranged from 38% to 99%.

### 2.2. Analysis of the Gene Structure and Conserved MYB Domains in PaMYBs

**Figure 1:**
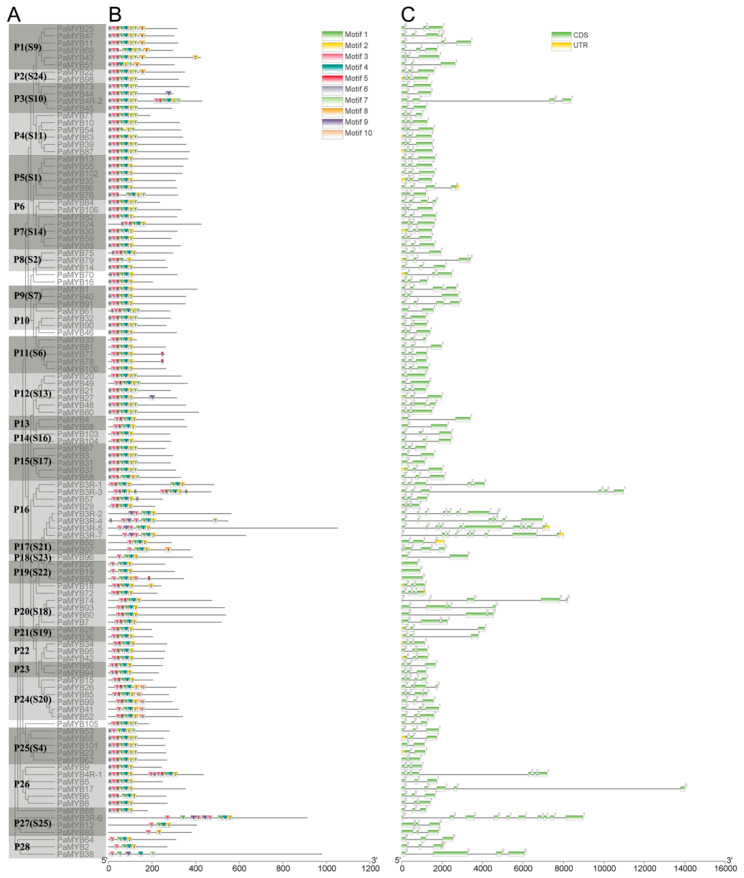
Phylogenetic and gene structure analysis of 2R-, 3R- and 4R-MYB genes in *Petunia*: (**A**) The phylogenetic tree was constructed with MEGA 10.1.8 using the neighbor-joining (NJ) method with 1000 bootstrap replicates based on a multiple alignment of amino acid sequences of MYB genes. The subgroup was classified and marked by alternated dark and light gray; (**B**) Protein motif. Schematic diagram of the conserved motifs in the MYB proteins, which were elucidated using MEME. Each motif is represented by a number in the colored box. The black lines represent the non-conserved sequences; (**C**) Gene structures of PaMYBs. The exons are represented by green box, the black lines connecting two exons represent introns and untranslated regions are indicated by yellow box.

According to the result of pre-screening, the sequence of 1R-MYB showed lowest similarity to other types of MYB genes. Thus, the analysis of gene structure and conserved domain was separated into two parts, the 1R-MYB part and the other MYBs part, respectively (Figure 1 and Supplementary Figure S1).

**Figure 2:**
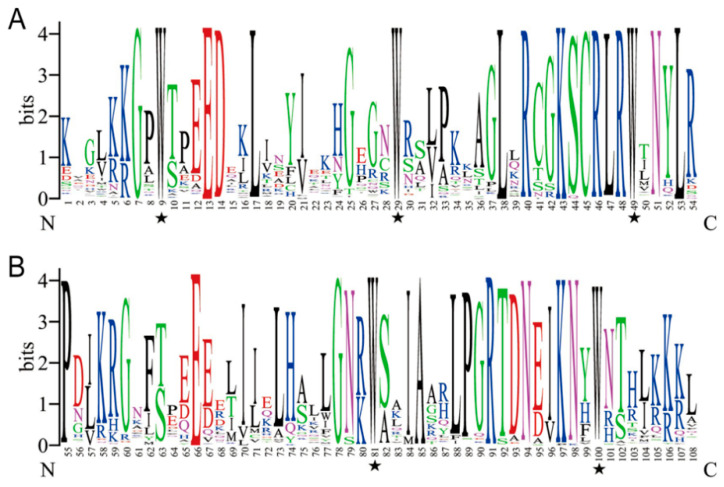
Consensus sequence and the level of conservation of R2R3-MYB domains in *Petunia*. The sequence logos of the R2 (**A**) and R3 (**B**) repeats are based on full-length alignments of petunia R2R3-MYB domains. The bit score indicates the information content for each position in the sequence. Asterisks indicate the conserved residues Trp (W) in the MYB domain.

In total, there are 10 conserved motifs identified in part of 2R-,3R-and 4R-MYB and part of 1R-MYB by MEME, respectively (Supplementary Tables S3 and S4). In order to better understanding the evolutionary relationships of MYB proteins in *Petunia*, an unrooted phylogenetic tree was constructed (Figure 1A and supplementary Figure S1A). Only motif 3 existed in every 2R-, 3R- and 4R-MYB protein. Besides this, most of the genes with close evolutionary relationships shared the same motif compositions, suggesting functional similarity of MYB proteins in the same group. To investigate the MYB DBD domain sequence features of petunia, the frequency of amino acid sequences of R2- and R3-repeats of 106 R2R3-MYB proteins was performed by WebLogo and multiple alignment analysis (Figure 2). R2-repeats contained motif 3, 5 and 1, and R3-repeats contained motif 4 and 2. In general, the basic regions of MYB domains had 108 basic residues, including the linker region. The R2 and R3 MYB repeats of the PaR2R3-MYBs contained characteristic amino acids, including three conserved Trp (W) residues identified in R2-repeat, while only the second and third Trp (W) were conserved in R3-repeat. The first Trp (W) in R3-repeat was usually replaced by F (69%)\I (13%) in most R2R3-MYBs. In R2-repeat, a total of 29 (out of 54) positions were occupied by a single residue in >70% of the proteins. For R3-repeat, the equivalent number was 30 (out of 54), indicating that R3-repeat was slightly more conserved than R2-repeat. The linker region between R2- and R3-repeats was highly conserved, and that four amino acids in the first half of the linker (LRPD, 53–56 amino acid) formed a highly conserved motif [38]. It is noteworthy that a similar motif exists in the linker region in tomato MYB proteins as well [39].

To understand the structure of *PaMYB* genes, the exon and intron organizations were studied by comparing the complementary DNA (cDNA) sequences with the corresponding genomic DNA sequences (Figure 1C and Supplementary Figure S1C). According to the results of gene structure analysis, the number of introns varies from 0 to 12, and most genes in the same subgroup have similar exon/intron structures. Subgroup P1 possessed no introns and all the members in P5 had multiple introns. Most of the R2R3-MYBs in petunia were typical splicing of three exons and two introns (73 of the 106 R2R3-MYBs, 67%). Although the lengths of intron and exon are different, the structure of R2R3-MYB in the same subfamily is highly conservative. On average, the 1R-MYBs were disrupted by 2.1 introns, 2R-MYBs were disrupted by 1.97 introns, 3R-MYBs were disrupted by 6.7 introns and 4R-MYBs were disrupted by 4 introns.

### 2.3. Phylogenetic Analysis and Classification of R2R3-MYB Proteins in Petunia

**Figure 3:**
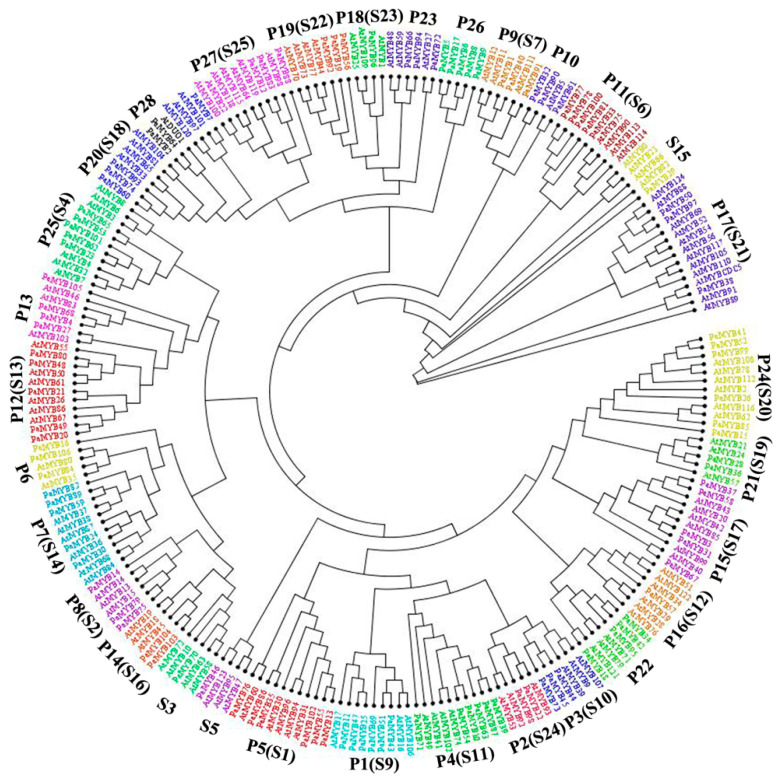
Phylogenetic tree of R2R3-MYB proteins between *Petunia axillaris* (P) and *Arabidopsis thaliana* (S). Neighbor-joining phylogeny was determined by MEGA 10.1.8. The colored shadow marks the subgroups of the R2R3-MYBs.

R2R3-MYB subfamily occupied the largest proportion with 68.39% of the total petunia MYB TF family. In order to better understanding the feature of R2R3-MYB in *Petunia*, an unrooted phylogenetic tree was constructed depending on the full-length sequences of R2R3-MYB proteins from *Petunia* and *Arabidopsis* (Figure 3 and Supplementary Table S5). Relying on the group classification of R2R3-MYB in *Arabidopsis*, the PaR2R3-MYBs were subdivided into 28 subgroups (designated P1-P28 in this study) based on the topology of the tree [40]. Most petunia proteins were found to be shared among the R2R3-MYBs from *Arabidopsis*. In contrast, seven clades (P6, P10, P13, P22, P23, P26, P28) were not clustered together with *Arabidopsis* MYBs and several clades were found only containing one species (P11, P18).

### 2.4. Promoter Analysis of PaMYB Genes

**Figure 4:**
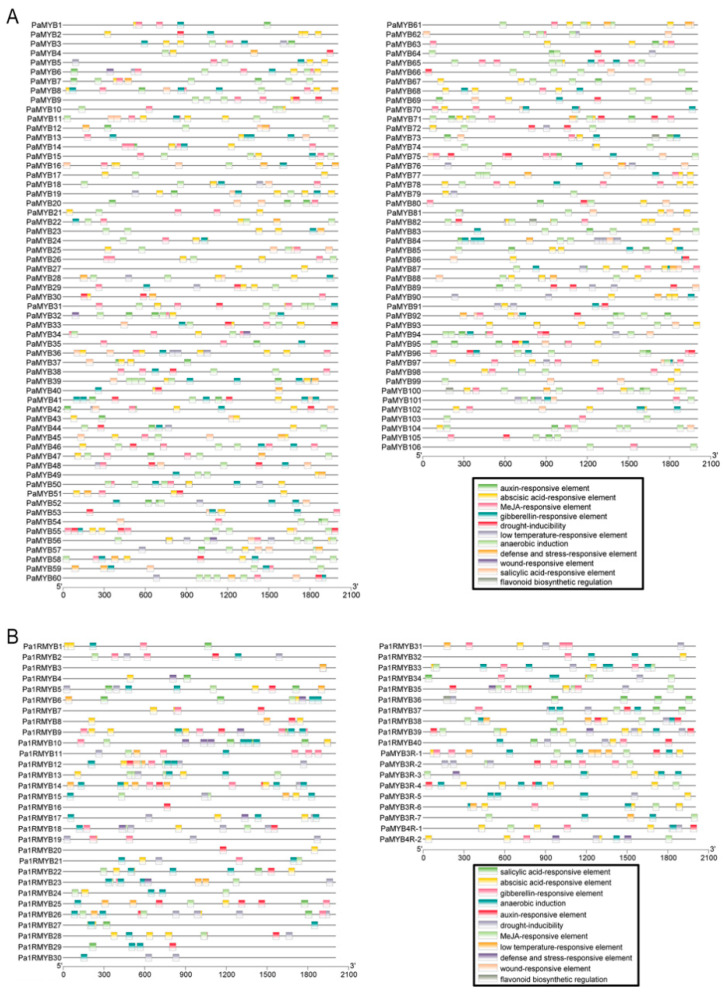
Regulatory elements in the promoter region of MYB genes in *Petunia*: (**A**) Regulatory elements in R2R3-MYB genes promoter. Different colors of box indicate different cis-elements; (**B**) Regulatory elements in 3R, 4R and 1R-MYB genes promoter.

For exploring the transcriptional regulation characteristics of PaMYBs, cis-elements were predicted using PlantCARE and marked on promoter regions (Figure 4). The hormone and stress responsive elements were widely distributed in PaMYBs promoter. By calculating the number of different cis-elements, MeJA-responsive element (CGTCA-motif and TGACG-motif) was the most frequent in PaMYBs promoter, followed by abscisic acid-responsive element (ABRE) and anaerobic induction (ARE) (Table 1). Notably, the common cis-elements in 1R-and R2R3-MYB promoters were significantly different to that in 3R- and 4R-MYB promoters. For example, flavonoid biosynthetic regulation (MBSI) and wound-responsive elements (WUN-motif) only existed in 1R- and R2R3-MYB promoters. None of 4R-MYB genes contained low temperature-responsive element (LTR) and salicylic acid-responsive element (TCA-element and SARE). This result suggested that transcriptional regulation of different types of PaMYB gene was diverse, indicating the diversity of PaMYBs function.

### 2.5. Expression of PaMYB Genes in Different Tissues

**Figure 5:**
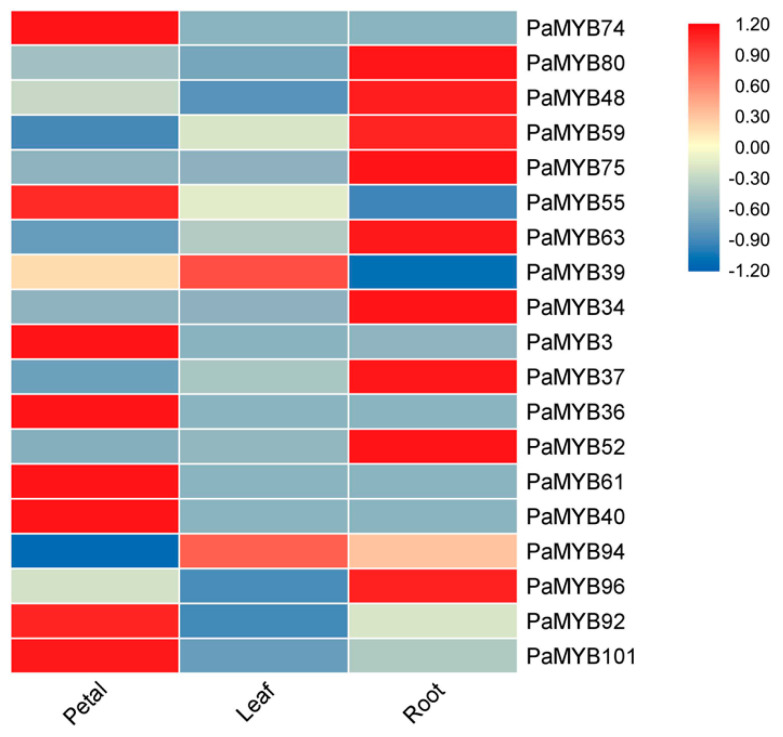
Tissue-specific gene expression of 19 R2R3-MYB genes of *Petunia*. The heat map shows the quantitative real-time polymerase chain reaction (qRT-PCR) analysis the expression level of PaMYB genes in in petal, leaf and roots. The gene expression level of tissue with highest expression level was considered as 1. Blocks with colors indicate decreased (blue) or increased (red) transcript accumulation relative to the respective control.

Based on RNA-seq database (unpublished data) owned by our lab, the expressions of all the *PaMYB* genes in petal, leaf and root were extracted (supplementary Table S5). A percentage of 32.9% of *PaMYB* genes showed no expression or were undetected. Thirty-two out of 155 *PaMYB* genes expressed in all the three tissues. Genes in subgroup 10, 11 and 21 were mainly expressed in petal, not in vegetative tissues. Genes in subgroup 9, 19, 25 and 27 expressed in all tissues, but the expression patterns of each gene were different. Among these three tissues, there are 27, 2 and 17 genes only expressed in petal, leaf and root, respectively. This may be caused by the different genetic background between varieties of sequencing and used in this work. To explore the specific functions of different subgroup of R2R3-MYB, we selected several *PaMYB* genes from each subgroup and determined their expression levels in petal, leaf and root by quantitative real-time polymerase chain reaction (qRT-PCR) (Figure 5). The expression patterns of each gene were different. For example, *PaMYB75* had the highest expression level in petal compared to leaf and root, while *PaMYB52* was mostly expressed in leaf. On the other hand, the transcripts of *PaMYB74*, *PaMYB59* and several genes were fewer in flower. This indicated that MYB genes in different subgroups may perform different functions during the growth and development of petunia.

### 2.6. Expression of PaMYB genes in Anthocyanin Biosynthesis

**Figure 6:**
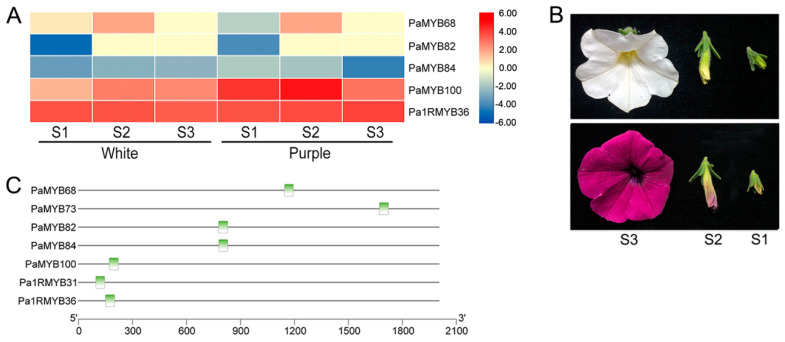
The expression and promoter analysis of 7 PaMYB genes that contained flavonoid biosynthetic regulation elements: (**A**) Heatmap showed the expression pattern of 5 *PaMYBs* in different coloration stage of white and purple petunia. S1 indicates budding period, S2 indicates extension period, S3 indicates blooming period; (**B**) The pictures of white and purple flowers; (**C**) The distribution of flavonoid biosynthetic regulation elements in 7 MYB genes. Green box indicates the flavonoid biosynthetic regulation elements.

Based on the promoter analysis, the expression of seven genes that contained MBSI cis-element, which was involved in flavonoid biosynthetic regulation, were quantified during petal coloring process of white and purple colored petunia (Figure 6). Two genes were not represented probably because that they were pseudogenes or not examined in these varieties. *PaMYB100* (annotated as *Deep purple, DPL*) showed relative higher expression level than other genes, and positive related to the coloration. This is consistent to its previously report [34]. Although *Pa1RMYB36* had a high expression, the differences in the coloring process and in different color plants were not significant. Except for flavonoid biosynthetic regulation element, drought-inducibility and SA-responsive element existed, indicating that *Pa1RMYB36* should have a prominent function in some coloring process related to abiotic stress response. The mRNA level of *PaMYB82* was only detected in bud. *PaMYB68* was not expressed in blooming. This result indicated that their function in flavonoid biosynthesis was regulated by other environment or development signals.

**Figure 7:**
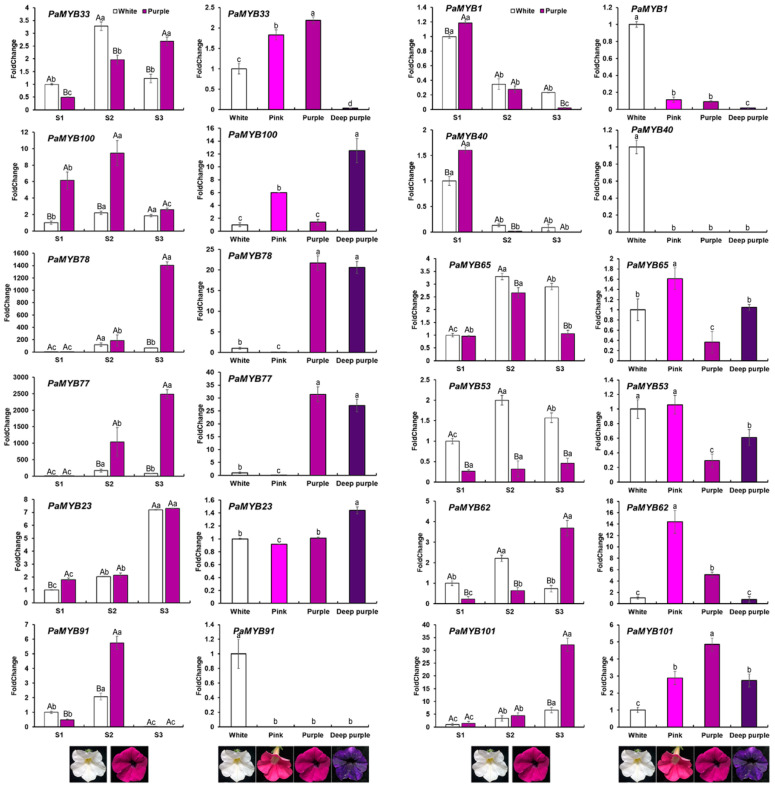
The significantly expressed MYB genes in *Petunia* in the pigmentation. The color of histogram represents the color of the petal. Each column represents the mean ± SE of three independent experiments each with three replicates. Different capitals indicate significant difference in the same stage. Different lowercases indicate significant difference in different stages.

Because genes in S3, P25 (S4), S5, P11 (S6), P9 (S7) and P16 (S12) were related to primary and secondary metabolic processes in *Arabidopsis*, we examined the expression level of these genes in pigmentation and different colored petals (Figure 7). Four colors of petals were selected, e.g., white, pink, purple and deep purple. To determine the gene expression level in different pigmentation, the expression level of R2R3-MYBs was detected in different colored petals. Because they have different total anthocyanin contents and anthocyanin components. On the other hand, to determine the gene expression level in anthocyanin biosynthesis, the expression pattern of R2R3-MYB genes was analyzed by comparing the three development stages of white and purple flowers. The expression pattern of genes in the same subgroup was similar in pigmentation and different colored petals. *PaMYB33/AN2* was belonged to P11 (S6), which was involved in flavonoid biosynthesis [41]. The expression level of *PaMYB33/AN2* was elevated during pigmentation. Genes in S9 (P7) were related to the flavonoid synthesis in vegetative organs, such as *AtMYB111* and *AtMYB12* [42,43]. *PaMYB1* and *PaMYB40* were mainly expressed in the budding period and colorless petal, suggesting that they were involved in regulation of flavonoid synthesis in petunia vegetative tissues. The expression pattern of genes in P16 (S12) was not significantly related to the pigmentation. We speculated that they may act as regulators in other secondary metabolic processes.

## 3. Discussion

The MYB transcription factor (TF) family was one of the largest TF families in plants. Although many PaMYBs have been reported, it is necessary to build an integral MYB family in *Petunia* for promoting phylogenetic and functional cognition. On the other hand, knowledge of the functions of certain members should facilitate the confirmation of paralogous and orthologous functional relationships.

In this study, the MYB members in *Petunia axillaris* genome were identified, including 106 R2R3-MYBs, 7 R1R2R3-MYBs, 2 4R-MYBs and 40 1R-MYB genes. R2R3-MYB subfamily is the most abundant subfamily in petunia, which is consistent with previous reports, with 126 R2R3-MYB members in *A. thaliana* [12], 121 in tomato [39]. MYB genes have undergone large-scale expansion in higher plants [44]. Restricted by the draft genome of petunia, we cannot localize the PaMYB genes to chromosomes. However, based on the number of PaMYBs, the MYB genes should be experienced gene expansion related to the genome duplications. For example, *PaMYB/ASR* was a duplication of the genomic fragment containing the other three R2R3-MYB genes with roles in pigmentation that were previously defined, the ANTHOCYANIN4-DEEP PURPLE-PURPLE HAZE (AN4-DPL-PHZ) cluster, through comparative, functional and phylogenic analysis of anthocyanin R2R3-MYB genes [35].

The number of intron and exon in the same group (subgroup) was similar. Interestingly, the most complex structure was 3R-MYB, not 4R-MYB. This result indicated that the number of conserved domains was not related to the complexity of gene structure. The motif composition and distribution orders in the same group (subgroup) were also similar, while the structures among the different groups significantly diverged. For example, only group 24 contained motif 10. The motif composition in group 16 was significantly different with other groups. Conserved motifs within the same TF family may play crucial roles in protein-specific functions. Three Trp (W) in R2-repeat and the second and third W in R3-repeat were conserved in petunia, as reported in plant MYB binding domain [45]. Like other plant R3-repeat, the first Trp (W) was usually replaced by other amino acid residue. W was replaced by F (69%)\I (13%) in most PaR2R3-MYBs, e.g., F/I in potato [9], F in soybean [13]. Except for the sequence conservation of R2- and R3-repeats, the sequence in the linkage region of PaMYBs were also highly conserved, which was consistent to tomato, explaining by the closely evolutionary relationship within Solanaceae [39].

As the most type of MYB genes, a phylogenetic tree of R2R3-MYB protein of *Petunia* and *Arabidopsis* was constructed for further explaining the evolutionary relationship of R2R3-MYB in petunia. R2R3-MYB in *Petunia* was divided into 28 subgroups referred to *Arabidopsis* [40], while seven of them were not clustered with *Arabidopsis*. In general, the orthologs clustered in a subgroup share similar gene structure and functions, indicating common evolutionary origins. AtMYB4 (subgroup 4) can repress the synthesis of sinapoylmalate [46]. In subgroup 25 of petunia, the clustered group of S4, *PaMYB101/PhMYB4* functioned in the repression of C4H transcription, indirectly regulating the floral volatile signature of petunia [47]. This reflected similar functions in the same subgroup, also it revealed that *PaMYB23*, *PaMYB53*, *PaMYB62* and *PaMYB65* may also be involved in primary and secondary metabolism. In P21 (S19), *PaMYB36/EOBI* and *PaMYB28/EOBII* were the homologous genes of *AtMYB21* and *AtMYB24*, respectively [48,49,50]. In *Arabidopsis*, MYB99 (belongs to S17) acts in a regulatory triad with MYB21 and MYB24. PaMYB3/ODORANT1 (belongs to P15), a putative ortholog of MYB99, could act with MYB21 and MYB24 to co-regulate petunia scent biosynthesis genes [51]. In S7 (P9), *AtMYB12*, *AtMYB11* and *AtMYB111* were significantly up-regulated by UV-B light to produce flavonols to protect from light damage [52]. PaMYB91/MYB-FL was the major determinant of differences in flavonol levels and UV absorption. The gain of UV absorbance in the derivation of *P. axillaris* from *P. inflata* was caused by upregulation of the MYB-FL promoter, whereas a frameshift mutation in PaMYB91/MYB-FL was responsible for the difference in UV absorbance between *P. axillaris* and *P. exserta* [53]. This result suggested the similar function within S7 (P9). In addition, genes in S7 (P9) were related to the flavonoid synthesis in vegetative tissues (stems, leaves and sepals) in response to stressors such as high light [42]. *PaMYB1* and *PaMYB40* were mainly expressed in the bud stage and colorless petal, suggesting that they were involved in regulation of flavonoid synthesis in petunia vegetative organs.

In *Arabidopsis*, the anthocyanin regulator *MYB75/PAP1* and MYB90/PAP2 belonged to subgroup 6 (S6) [18,41], which was clustered with subgroup 11 (P11) in petunia, with five PaR2R3-MYB genes included. *PaMYB33/AN2* played a central role in petal limb pigmentation, while *PaMYB77/PaMYB78/AN4* was the regulator of anthocyanin in petal tube and anthers [32,33]. The mRNA level of *PaMYB33/AN2* and *PaMYB77/PaMYB78/AN4* were elevated in the process of pigmentation (Figure 7). *PaMYB100/DPL* regulated vein-associated anthocyanin pigmentation in the flower tube, while *PaMYB81/PHZ* determined light-induced anthocyanin accumulation on exposed petal surfaces (bud-blush) [34]. Similarity, the expression level in purple petal of *PaMYB100/DPL* and *PaMYB81/PHZ* were higher than that in white petal. Among these five genes, only promoter of *PaMYB100/DPL* contained flavonoid biosynthetic regulation (MBSI) element, suggesting that MBSI was not necessary for regulation of anthocyanin biosynthesis. Genes in subgroup 9 were related to the flavonoid synthesis in vegetative organs, such as *AtMYB111* and *AtMYB12* [42,43]. In petunia subgroup 7, *PaMYB1* and *PaMYB40* were mainly expressed in the bud stage and colorless petal, suggesting that they were involved in regulation of flavonoid synthesis in petunia vegetative organs. In addition, ANTHOCYANIN SYNTHESIS REGULATOR (ASR) was the novel anthocyanin regulators in the genome of the purple flowering *P. inflata* S6 wild accession, while it was absent in the white flowering species *P. axillaris* [35]. Apart from positive regulators of anthocyanin, negative regulators were also reported. *PaMYB105/PhMYB27*, a putative R2R3-MYB anthocyanin repressor that functions as part of the MYB-bHLH-WD40 (MBW) complex and represses transcription through its C-terminal EAR motif, was significantly up-regulated in white petal. Another anthocyanin repressor, *PhMYBx* (R3-MYB) was a competitive repressor of AN1, which was an essential component of the MBW activation complex for pigmentation [36]. However, MYBx was not identified in the white flowering *P. axillaris* in this work.

Based on cis-element analysis, seven gene promoters contained flavonoid biosynthetic regulation element, indicating their regulatory roles in flavonoid biosynthesis. Except for *PaMYB100/DPL*, another six genes were not reported as the regulator of flavonoid biosynthesis. In the most cases, R2R3-MYBs were the main regulators of flavonoid biosynthesis. In our study, a MYB-related gene, *Pa1RMYB36* contained a flavonoid biosynthetic regulation element, suggesting that it was a potential regulator of flavonoid biosynthesis. Although its expression has little relationship to the coloration, it may play an important role in another flavonoid biosynthetic pathway. The regulator of grape berry skin, *VvmybA1*, was a MYB-related gene. In addition, its homologs, *VvMYBA2r*, *VlmybA1-1*, *VlmybA1-2*, and *VlmybA2*, also regulated anthocyanin biosynthesis [54]. Through the identification, classification and evolutionary analysis of MYB gene family in *Petunia*, our result was supported by published data. Except for existing knowledge, many new MYB genes were explored and were of research value. Nevertheless, due to the limitation of the reference genome, there are still some MYB genes that have not been identified, so follow-up research will expand the genome information in order to obtain more comprehensive PaMYB family data.

## 4. Materials and Methods

### 4.1. Identification of Petunia MYB Proteins

To identify potential members of petunia *MYB* gene family, we performed multiple database searches. The amino acids sequences of the *Arabidopsis* MYB proteins were downloaded from the Arabidopsis Information Resource (TAIR, http://www.Arabidopsis.org/, accessed on 1 June 2020), and were employed as a query in BLAST searches against the *Petunia axillaris* genome database (https://www.sgn.cornell.edu/, accessed on 1 June 2020) [37]. All tentative consensus sequences and singlet sequences with an e-value of <1.0 were selected after the homology search. Any uncompleted sequences or somewhat different conserved domains were discarded from the data list. Then, the cDNA sequences, amino acid sequences and genomic sequences were extracted for further analysis. The theoretical isoelectric point (PI) and molecular weights (MW) of the PaMYB proteins were predicted by Compute PI/MW tool on the ExPASy server (http://web.expasy.org/compute_pi/, accessed on 1 June 2020).

### 4.2. Phylogenetic Analysis

The alignments of full-length amino acid sequences of PaMYBs and AtMYBs were performed using ClustalX 2.1 with default settings [55]. An unrooted phylogenetic tree was constructed based on the alignments using MEGA 10.1.8 with the Neighbor-Joining (NJ) method [56]. The parameters used in the tree construction were JTT model plus gamma-distributed rates determined by ProtTest 3.0 [57] and 1000 bootstraps. The pairwise gap deletion mode was used to ensure that the more divergent C-terminal domains could contribute to the topology of the NJ tree. We further performed a NJ phylogenetic tree of the R2R3-MYB protein sequences of *Petunia* and *Arabidopsis* for classifying PaR2R3-MYB.

### 4.3. Conserved Motif Identification and Gene Structure Analysis

The deduced amino acid sequences of the PaMYBs were analyzed by Multiple Em for Motif Elicitation (MEME; http://memesuite.org/tools/meme, accessed on 1 July 2020) for motif analysis [58]. To identify conserved motifs in these sequences, selection of the maximum number of motifs was set to 10 and optimum width of motif from 6 to 200. The motif with an e-value less than 1e^−10^ was retained for further analysis. The amino acid sequence in PaMYB motifs were manually adjusted using Weblogo online program (http://weblog.berkeley.edu/logo.cgi, accessed on 1 July 2020) to display the characteristics of the MYB domains [59]. The exon-intron structure of these genes was graphically displayed by the Gene Structure Display Server [60] based on the genomic sequences and the corresponding coding sequences (CDS).

### 4.4. Promoter Cis-Element Analysis

The promoter sequences (2000 bp upstream of start codon) of PaMYB genes were collected. The cis-acting elements are predicted in PlantCARE [61]. Statistical analysis and data visualization were done by Excel and TBtools [62].

### 4.5. Plant Materials and Growth Condition

Four varieties of *Petunia × hybrida* inbred lines with different petal colors were used in this research. These inbred lines showed different petal colors. The plants were grown in a chamber with a 16-h light/8-h dark cycle under 25 °C. The petal, leaf and root of purple flowering variety were used as samples for tissue specific expression detection. To determine the gene expression level in anthocyanin biosynthesis, the petals were taken in different developmental stages of white and purple flowering petunia, including budding period (S1), expansion period (S2) and blooming period (S3). To determine the gene expression level in different pigmentation, four colored petals in S3 were employed. Three biological replicates were conducted for each group.

### 4.6. Quantitative RT-PCR Analysis

RNA of all the samples was extracted using plant RNA extraction kit (TaKaRa Bio Inc., Dalian, China) and reversed to single strand cDNA using Prime-Script RT reagent kit (TaKaRa Bio Inc., Dalian, China) according to the instructions. qRT-PCR was performed by SYBR Premix EX Taq II (TaKaRa Bio Inc., Dalian, China) on the Eppendorf Mastercycler ep Realplex system. Primers are listed in Supplementary Table S6. The gene expression quantification was calculated using the 2^−ΔΔCt^ method.

## 5. Conclusions

In this work, 155 MYB genes in *P. axillaris* were identified, including 106 R2R3-MYB, 7 3R-MYB, 2 4R-MYB and 40 MYB-related genes. The gene structure and motif composition were highly conserved in petunia MYB family. Through analysis of evolutionary relationship of R2R3-MYB genes between *Arabidopsis* and *Petunia*, genes in the same subgroup shared similar functions, which was supported by published data. We also obtained several unknown MYB genes had potential function in anthocyanin biosynthesis by means of promoter analysis. This work provides a comprehensive understating of the MYB gene family in *Petunia* and lays a significant foundation for future studies on the function and evolution of MYB genes in petunia.

## Figures and Tables

**Table 1:** The summary of cis-element involved in hormone, stress and development responses in PaMYB promoters.

Cis-Element	1R-MYB	2R-MYB	3R-MYB	4R-MYB	Total
MeJA-responsive element	86	238	20	4	348
abscisic acid-responsive element	67	210	16	7	300
anaerobic induction	71	186	17	2	276
gibberellin-responsive element	38	98	6	2	144
salicylic acid-responsive element	26	76	1	0	103
auxin-responsive element	28	64	4	1	97
drought-inducibility	42	50	4	3	99
low temperature-responsive element	20	49	6	0	75
defense and stress-responsive element	12	34	1	2	49
flavonoid biosynthetic regulation	2	5	0	0	7
wound-responsive element	1	4	0	0	5
Total	86	238	20	4	348

## Data Availability

The genomic data of *Petunia axillarix* are openly available in Sol Genomics Network at doi:10.1038/nplants.2016.74, reference [37]. The other data presented in this study are available in Supplementary Materials.

## Supplementary Materials

The following are available online at https://www.mdpi.com/article/10.3390/ijms22094838/s1.

## References

[1] Roy S. (2016). Function of MYB domain transcription factors in abiotic stress and epigenetic control of stress response in plant genome. Plant Signal. Behav..

[2] Baldoni E., Genga A., Cominelli E. (2015). Plant MYB Transcription Factors: Their Role in Drought Response Mechanisms. Int. J. Mol. Sci..

[3] Chen C., Zhang K., Khurshid M., Li J., He M., Georgiev M.I., Zhang X., Zhou M. (2019). MYB Transcription Repressors Regulate Plant Secondary Metabolism. Crit. Rev. Plant Sci..

[4] Paz-Ares J., Ghosal D., Wienand U., Peterson P.A., Saedler H. (1987). The regulatory c1 locus of Zea mays encodes a protein with homology to myb proto-oncogene products and with structural similarities to transcriptional activators. EMBO J..

[5] Dubos C., Stracke R., Grotewold E., Weisshaar B., Martin C., Lepiniec L. (2010). MYB transcription factors in *Arabidopsis*. Trends Plant Sci..

[6] Tian F., Yang D.-C., Meng Y.-Q., Jin J., Gao G. (2019). PlantRegMap: Charting functional regulatory maps in plants. Nucleic Acids Res..

[7] Li X., Guo C., Ahmad S., Wang Q., Yu J., Liu C., Guo Y. (2019). Systematic Analysis of MYB Family Genes in Potato and Their Multiple Roles in Development and Stress Responses. Biomolecules.

[8] Martin C., Paz-Ares J. (1997). MYB transcription factors in plants. Trends Genet..

[9] Ramsay R.G., Gonda T.J. (2008). MYB function in normal and cancer cells. Nat. Rev. Cancer.

[10] Zhu K., Fan P., Mo Z., Tan P., Feng G., Li F., Peng F. (2020). Identification, Expression and Co-Expression Analysis of R2R3-MYB Family Genes Involved in Graft Union Formation in Pecan (*Carya illinoinensis*). Forests.

[11] Ogata K., Kanei-Ishii C., Sasaki M., Hatanaka H., Nagadoi A., Enari M., Nakamura H., Nishimura Y., Ishii S., Sarai A. (1996). The cavity in the hydrophobic core of Myb DNA-binding domain is reserved for DNA recognition and transactivation. Nat. Genet..

[12] Chen Y.H., Yang X.Y., He K., Liu M.H., Li J.G., Gao Z.F., Lin Z.Q., Zhang Y.F., Wang X.X., Qiu X.M. (2006). The MYB transcription factor superfamily of *Arabidopsis*: Expression analysis and phylogenetic comparison with the rice MYB family. Plant Mol. Biol..

[13] Du H., Yang S.-S., Liang Z., Feng B.-R., Liu L., Huang Y.-B., Tang Y.-X. (2012). Genome-wide analysis of the MYB transcription factor superfamily in soybean. BMC Plant Biol..

[14] Prouse M.B., Campbell M.M. (2012). The interaction between MYB proteins and their target DNA binding sites. Biochim. Biophys. Acta (BBA).

[15] Grotewold E. (2006). The Genetics and Biochemistry of Floral Pigments. Annu. Rev. Plant Biol..

[16] Kobayashi S., Goto-Yamamoto N., Hirochika H. (2005). Association of vvmyba1 gene expression with anthocyanin production in grape (*Vitis vinifera*) skin-color mutants. J. Jpn. Soc. Hortic. Sci..

[17] Zhang P., Dong Y., Wen H., Liang C., Wen P. (2019). Knockdown of vvmyba1 via virus-induced gene silencing decreases anthocyanin biosynthesis in grape berries. Can. J. Plant Sci..

[18] Teng S., Keurentjes J., Bentsink L., Koornneef M., Smeekens S. (2005). Sucrose-Specific Induction of Anthocyanin Biosynthesis in Arabidopsis Requires the MYB75/PAP1 Gene. Plant Physiol..

[19] Li N., Wu H., Ding Q., Li H., Li Z., Ding J., Li Y. (2018). The heterologous expression of *Arabidopsis* PAP2 induces anthocyanin accumulation and inhibits plant growth in tomato. Funct. Integr. Genom..

[20] Espley R.V., Hellens R.P., Putterill J., Stevenson D.E., Kutty-Amma S., Allan A.C. (2007). Red coloration in apple fruit is due to the activity of the MYB transcription factor, MdMYB10. Plant J..

[21] Chen G., Xu P., Pan J., Li Y., Zhou J., Kuang H., Lian H. (2020). Inhibition of FvMYB10 transcriptional activity promotes color loss in strawberry fruit. Plant Sci..

[22] Aharoni A., De Vos C.H.R., Wein M., Sun Z., Greco R., Kroon A., Mol J.N.M., O’Connell A.P. (2001). The strawberry FaMYB1 transcription factor suppresses anthocyanin and flavonol accumulation in transgenic tobacco. Plant J..

[23] Wang X.C., Wu J., Guan M.L., Zhao C.H., Geng P., Zhao Q. (2020). *Arabidopsis* MYB4 plays dual roles in flavonoid biosynthesis. Plant J..

[24] Zhou M., Zhang K., Sun Z., Yan M., Chen C., Zhang X., Tang Y., Wu Y. (2017). LNK1 and LNK2 Corepressors Interact with the MYB3 Transcription Factor in Phenylpropanoid Biosynthesis. Plant Physiol..

[25] Stracke R., Ishihara H., Huep G., Barsch A., Mehrtens F., Niehaus K., Weisshaar B. (2007). Differential regulation of closely related R2R3-MYB transcription factors controls flavonol accumulation in different parts of the *Arabidopsis thaliana* seedling. Plant J..

[26] Denekamp M., Smeekens S.C. (2003). Integration of Wounding and Osmotic Stress Signals Determines the Expression of the AtMYB102 Transcription Factor Gene. Plant Physiol..

[27] Lippold F., Sanchez D.H., Musialak M., Schlereth A., Scheible W.-R., Hincha D.K., Udvardi M.K. (2009). AtMyb41 Regulates Transcriptional and Metabolic Responses to Osmotic Stress in *Arabidopsis*. Plant Physiol..

[28] Abe H., Urao T., Ito T., Seki M., Shinozaki K., Yamaguchi-Shinozaki K. (2003). *Arabidopsis* AtMYC2 (bHLH) and AtMYB2 (MYB) Function as Transcriptional Activators in Abscisic Acid Signaling. Plant Cell.

[29] Devaiah B.N., Madhuvanthi R., Karthikeyan A.S., Raghothama K.G. (2009). Phosphate Starvation Responses and Gibberellic Acid Biosynthesis are Regulated by the MYB62 Transcription Factor in *Arabidopsis*. Mol. Plant.

[30] Cornu A., Maizonnier D., Janick J. (1983). The Genetics of Petunia. Plant Breeding Reviews.

[31] Albert N.W., Lewis D.H., Zhang H., Irving L.J., Jameson P.E., Davies K.M. (2009). Light-induced vegetative anthocyanin pigmentation in *Petunia*. J. Exp. Bot..

[32] Quattrocchio F., Wing J., van der Woude K., Souer E., de Vetten N., Mol J., Koes R. (1999). Molecular analysis of the anthocyanin2 gene of petunia and its role in the evolution of flower color. Plant Cell.

[33] Brugliera F., Barri-Rewell G., Holton T.A., Mason J.G. (1999). Isolation and characterization of a flavonoid 3’-hydroxylase cDNA clone corresponding to the Ht1 locus of *Petunia hybrida*. Plant J..

[34] Albert N.W., Lewis D.H., Zhang H., Schwinn K.E., Jameson P.E., Davies K.M. (2011). Members of an R2R3-MYB transcription factor family in Petunia are developmentally and environmentally regulated to control complex floral and vegetative pigmentation patterning. Plant J..

[35] Zhang H., Koes R., Shang H., Fu Z., Wang L., Dong X., Zhang J., Passeri V., Li Y., Jiang H. (2019). Identification and functional analysis of three new anthocyanin R2R3-MYB genes in *Petunia*. Plant Direct.

[36] Albert N.W., Davies K.M., Lewis D.H., Zhang H., Montefiori M., Brendolise C., Boase M.R., Ngo H., Jameson P.E., Schwinn K.E. (2014). A Conserved Network of Transcriptional Activators and Repressors Regulates Anthocyanin Pigmentation in Eudicots. Plant Cell.

[37] Bombarely A., Moser M., Amrad A., Bao M., Bapaume L., Barry C.S., Bliek M., Boersma M.R., Borghi L., Bruggmann R. (2016). Insight into the evolution of the Solanaceae from the parental genomes of *Petunia hybrida*. Nat. Plants.

[38] Hegvold A.B., Gabrielsen O.S. (1996). The importance of the linker connecting the repeats of the c-Myb oncoprotein may be due to a positioning function. Nucleic acids Res..

[39] Li Z., Peng R., Tian Y., Han H., Xu J., Yao Q. (2016). Genome-Wide Identification and Analysis of the MYB Transcription Factor Superfamily in *Solanum lycopersicum*. Plant Cell Physiol..

[40] Stracke R., Werber M., Weisshaar B. (2001). The R2R3-MYB gene family in *Arabidopsis thaliana*. Curr. Opin. Plant Biol..

[41] Zuluaga D.L., Gonzali S., Loreti E., Pucciariello C., Degl’Innocenti E., Guidi L., Alpi A., Perata P. (2008). *Arabidopsis thaliana* MYB75/PAP1 transcription factor induces anthocyanin production in transgenic tomato plants. Funct. Plant Biol..

[42] Mehrtens F., Kranz H., Bednarek P., Weisshaar B. (2005). The *Arabidopsis* Transcription Factor MYB12 is a Flavonol-Specific Regulator of Phenylpropanoid Biosynthesis. Plant Physiol..

[43] Stracke R., Jahns O., Keck M., Tohge T., Niehaus K., Fernie A.R., Weisshaar B. (2010). Analysis of Production of Flavonol Glycosides-Dependent Flavonol Glycoside Accumulation in *Arabidopsis Thaliana* Plants Reveals MYB11-, MYB12- and MYB111-Independent Flavonol Glycoside Accumulation. New Phytol..

[44] Feller A., Machemer K., Braun E.L., Grotewold E. (2011). Evolutionary and comparative analysis of MYB and bHLH plant transcription factors. Plant J..

[45] Ambawat S., Sharma P., Yadav N.R., Yadav R.C. (2013). MYB transcription factor genes as regulators for plant responses: An overview. Physiol. Mol. Biol. Plants.

[46] Hemm M.R., Herrmann K.M., Chapple C. (2001). AtMYB4: A transcription factor general in the battle against UV. Trends Plant Sci..

[47] Colquhoun T.A., Kim J.Y., Wedde A.E., Levin L.A., Schmitt K.C., Schuurink R.C., Clark D.G. (2011). PhMYB4 fine-tunes the floral volatile signature of *Petunia x hybrida* through PhC4H. J. Exp. Bot..

[48] Mandaokar A., Browse J. (2009). MYB108 Acts Together with MYB24 to Regulate Jasmonate-Mediated Stamen Maturation in *Arabidopsis*. Plant Physiol..

[49] Mandaokar A., Thines B., Shin B., Lange B.M., Choi G., Koo Y.J., Yoo Y.J., Choi Y.D., Choi G., Browse J. (2006). Transcriptional regulators of stamen development in Arabidopsis identified by transcriptional profiling. Plant J..

[50] Spitzer-Rimon B., Farhi M., Albo B., Cna’Ani A., Ben Zvi M.M., Masci T., Edelbaum O., Yu Y., Shklarman E., Ovadis M. (2012). The R2R3-MYB–Like Regulatory Factor EOBI, Acting Downstream of EOBII, Regulates Scent Production by Activating ODO1 and Structural Scent-Related Genes in Petunia. Plant Cell.

[51] Battat M., Eitan A., Rogachev I., Hanhineva K., Fernie A., Tohge T., Beekwilder J., Aharoni A. (2019). A MYB Triad Controls Primary and Phenylpropanoid Metabolites for Pollen Coat Patterning. Plant Physiol..

[52] Stracke R., Favory J.J., Gruber H., Bartelniewoehner L., Bartels S., Binkert M., Funk M., Weisshaar B., Ulm R. (2010). The *Arabidopsis* bZIP transcription factor HY5 regulates expression of the PFG1/MYB12 gene in response to light and ultraviolet-B radiation. Plant Cell Environ..

[53] Sheehan H., Moser M., Klahre U., Esfeld K., Dell’Olivo A., Mandel T., Metzger S., Vandenbussche M., Freitas L., Kuhlemeier C. (2016). MYB-FL controls gain and loss of floral UV absorbance, a key trait affecting pollinator preference and reproductive isolation. Nat. Genet..

[54] Azuma A., Kobayashi S., Mitani N., Shiraishi M., Yamada M., Ueno T., Kono A., Yakushiji H., Koshita Y. (2008). Genomic and genetic analysis of Myb-related genes that regulate anthocyanin biosynthesis in grape berry skin. Theor. Appl. Genet..

[55] Thompson J.D., Higgins D.G., Gibson T.J. (1994). CLUSTAL W: Improving the sensitivity of progressive multiple sequence alignment through sequence weighting, position-specific gap penalties and weight matrix choice. Nucleic Acids Res..

[56] Tamura K., Stecher G., Peterson D., Filipski A., Kumar S. (2013). MEGA6: Molecular Evolutionary Genetics Analysis version 6.0. Mol. Biol. Evol..

[57] Darriba D., Taboada G.L., Doallo R., Posada D. (2011). ProtTest 3: Fast selection of best-fit models of protein evolution. Bioinformatics.

[58] Bailey T.L., Johnson J., Grant C.E., Noble W.S. (2015). The MEME Suite. Nucleic Acids Res..

[59] Crooks G.E., Hon G., Chandonia J.-M., Brenner S.E. (2004). WebLogo: A Sequence Logo Generator. Genome Res..

[60] Hu B., Jin J., Guo A.-Y., Zhang H., Luo J., Gao G. (2015). GSDS 2.0: An upgraded gene feature visualization server. Bioinformatics.

[61] Lescot M., Déhais P., Thijs G., Marchal K., Moreau Y., Van de Peer Y., Rouzé P., Rombauts S. (2002). PlantCARE, a data-base of plant cis-acting regulatory elements and a portal to tools for in silico analysis of promoter sequences. Nucleic Acids Res..

[62] Chen C., Chen H., Zhang Y., Thomas H.R., Frank M.H., He Y., Xia R. (2020). TBtools: An Integrative Toolkit Developed for Interactive Analyses of Big Biological Data. Mol. Plant.

