# Supplementary material for: Genome-Wide Identification, Classification and Expression Analysis of the MYB Transcription Factor Family in Petunia
Source: Int J Mol Sci. 2021 May 3;22(9):4838. doi: 10.3390/ijms22094838 (PMC8124715; doi:10.3390/ijms22094838)
Supplement: Supplementary file 1 [file ijms-22-04838-s001.zip › supplementary files/supplementary Table S1.pdf]

Supplementary Table S1. The sequences of all identified PaMYB genes.

Part I. The nucleotide sequences of PaMYB gene family.

Part II. The protein sequences of PaMYB gene family.

Part I. The nucleotide sequences of PaMYB gene family.

>Peaxi162Scf00001g00231.1

ATGGGAAGAGCACCTTGTTGCGAGAAAGTGGGTCTCAAGAGAGGTAGATGGACT
GCAGAGGAAGATGAAACCCTCACCAACTATATTTTGACTAACGGCGAAGGTTCTT
GGAGGTCATTACCCAAAAATGCTGGCTTACTCAGATGTGGGAAAAGTTGCCGACT
GAGATGGATTAATTACTTGAGATCTGATTTGAAGAGAGGTAACATAACTTCTGAAG
AGGAAAACATAATCATCAAGTTGCATGCAACTTTGGGTAACAGATGGTCTCTAATA
GCCGGACATTTGCCAGGTAGAACAGACAATGAGATTAAAAACTACTGGAACTCTC
ATCTAAGTAGGAAAGTTGAAAGCTTAAGGATTCCAAGTGATGAAAAGCTGCCTCA
AGCTGTAGTAGATTTGGCTAGAAAGGGAGCATTAAAGCAAATCAAACGTAAAGTT
GGCCGAACAAGCCGATCTACCATGATGAGAAACAGGAAGTCAAAAAATTCCGCTG
TTTCAAGTTTGTCAATGCCAAAGCAACTTAAAGAAAGTAGTGAACCTTTAAATGCA
ACAGTCCCTATCATGCCGTCAACCCCAAACTTGGAGAAAGAGGCCTTATCTAGGAC
TAGCACCATTAGCTCATGGCTAGATGGTAATAATGCCATGGATTCCATGCAAAAAGA
GGTAGCAAACATAGCGGCGCCAAATCCTTTGATGGAGTCTAGAGAGGCTCATAGC
AGCCTTAGTTCAGATGGGATGGAGTGGCTTGAAGAGATTATGCCACTGGTCATTGA
TGATCAAGATATGGACCCAAATTTCATATTTACTTGTTTAGAGAATGGGCAGGGAG
AAAGCGCAGTAAAAGTCACCGAGGAGGCAGATAATAACTTCTTGAACACAAGTAA
AATTAATGAACGTGACAACAATAACAGAGAATCAAGTGAGGCAGCTGTATCAATA
AATATTCATGATGAACAAGTACACGAGAAAAGCAACGAAACGAGTTCTTTAATGA
AGGATGAAGGTACATTTGATCAATGGGATTGGAAAGAGATAACTCATGATGGAGGA
AAAGGCTGGCCATGGGATGACAACAATTCGCTGCAGCTATGGGATACGACTACTG
ATGACACCGGGTTTTTTCAAAACTGTATAAATGAAGCAACGGTGGAAATGGATTCC
GTGCACTTTGAAAACCAAAAGCATAGTGCTCTTGTCGCTTGGCTTTTGTCTTAG

>Peaxi162Scf00001g00599.1

ATGGAAGGAAGAAGACCATCATCTTCATATCATCATTATCGTTATCATTTTCATCATG
AAGATGAACTTGGAGAAATAAAGAAAGGACCATGGAAAGCAGAAGAAGGTGAAG
TGTTATTAAATCATGTGAAGAAATATGGTCCAAGAGATTGGAGCTCCATTCGATCCA
AAGGTTTACTGCAACGTACTGGAAAATCTTGTCGTCTTTATTGGGTTAATAAACTTC
GACCCAACTTGAAGAATGGAGTGAAGTTCTCAGCAGAGGAGGAAAGGACAGTGA

TTGAACTTCAGGCACAATTTGGGAACAAATGGGCAAGAATTGCTACACATTTGCAT
GGAAGGACTGACAATGATGCACCTAAATTCAGTTCATCAGCAGATCAAGAAGAGT
TTTTATCCAAGTCTCAATCTTGTTCATCATCCTATATTGATAACTCTAACATGATCTAC
CTGGTTCCATTGAATCCAAATTCAACTGATTTTGAAGCAAACTTGCTTCAACTTGAT
TTCACTGCTAATGAGAAAAAACTCAAAATAGATTCACATATTCAGCTCCCTTTTACT
AAGCTTCAAAATGACTTTGCTCTGCCGTTACAAACTCATGAATTCCCTATGCCCAA
CTTTATAGATGTTTTCGGTCAGCAGCTCAATGGTTCTGAATTAGATAATGTGCAAGT
CCCATTTGTATCGACGTGCTCGGGACCAGATAGATCATGTTTTGAGAATCCTTCAAG
TCCAGATAGCTTCATTGATGATTTTCCTTTGGATATGTTTGATCACATTGAACCTCTG
CCTAGCCAATCCGAA
>Peaxi162Scf00002g00037.1
ATGGGAAGACAACCTTGTTGTGACAAACTTGGAGTGAAGAAAGGTCCATGGACA
GCTGAAGAAGACAAGAAACTCATAAGTTTTATTCTTACAAATGGCCAATGTTGTTG
GCGTGCTGTTCCTAAACTTGCTGGTCTTAGACGTTGTGGTAAGAGTTGTCGACTGA
GATGGACTAATTATCTTCGACCTGATTTGAAAAGAGGCCTTCTTAGTGATGCTGAG
GAGAAACTGGTTATTGATCTCCATTCTCGTCTTGGAAACAGGTGGTCCAAGATTGC
TGCAAGATTACCAGGAAGGACGGATAATGAGATTAAAAATCATTGGAACACACATA
TTAAGAAAAAGCTTCTCAAAATGGGGATTGATCCTGTTACACATGAACCACTCAAG
AAAGAAGCAAATCTAAGTGACCAGCCTAATACAGAATCTGATCAAAATAAAGAAA
ATGGTCATCAGCAGGTACAAGTTGTACCACAGAGTACAAATGTCACAGCGGCAGC
AGCCACCTCCACAGAATTTGATAATAACTCTTCCTTCTCTTCTTCAGCTTCTTCGTC
CGAAAACTCTTCATGCACCACTAATGAATCCAAGCTTATCTTTGATAACCTTAGTGA
AAATGATCCACTACTAAGCTGCCTACTGGAAGCTGATACTCCTCTTATCGATTCGCC
ATGGGAATTTCCAATGTCTTCTACTACTACTGCTGAAGAACCAAAAAGTTTCGATA
GCATTATTAGTAACATGACATCCTGGGAAGACACTTTCAATTGGCTTTCGGGTTGTG
AAGAATTTGGTATCAATGACTTTGGTTTTGATAATTGCTTCAACCATGTCGAATTGG
ACATCTTCAAAACCATAGATAACGTAGAGAACAGGCACGGATAA
>Peaxi162Scf00004g02328.1
ATGAGAAAGCCTGAGTTCTCCTCCTCCGGCAAGAATGGAACCAACAGTAACAACA
ACATCAATAACGCGAACATGAAGTTAAGAAAAGGCTTGTGGTCGCCGGAGGAAGA

TGAGAAGCTCATGCATTATATGTTGACAAATGGACAAGGTTGTTGGAGTGATGTGG
CAAGAAATGCTGGGTTACAAAGGTGTGGGAAGAGTTGTAGACTAAGGTGGATTAA
TTATTTAAGGCCTGACCTTAAAAGAGGTGCATTTTCACCTCAAGAAGAAGAGCTTA
TTATCCATTTACATTCCCTTCTTGGTAACAGGTGGTCTCAAATAGCTGCACGTATGC
CTGGACGTACTGATAATGAAATCAAGAATTTTTGGAATTCAACATTGAAAAAGAGG
CTAAAGAACTCATCATCTTGCTCTACACCATCACCAAATGCAAGTGACTCATCCTC
AGAACCCTGCAAAGATAATCTCAACATGGGAATCAATCAAGGATTGATCATGTCAA
TGCAAAATCATAACCTAATGTCCATGTTCATGGACTCAACCTCGTCTTCTTCCTCGT
CTATGGCCCTAAATACCATTATTGAACCTTTGCCCATGCTCGAGCAAACCCTAATAA
ACATGCCTAATGGATTGAGTGCACCTCCATACTTGACTACACAACCATGCATGACA
CAAGGAAGGAGTATTGTGACCAATGGAAGTCTTTTTATGGGAATAATCATGGCAT
ATTTGGAGGAAATCTTGGTATGGAAGGAGAGCTCTATATTCCACCTTTGGAGAGTG
TAAGCATTCATGAATATCAAAATGTTGAAAATGGGAATCTAGTTGAAAGGAGCAGT
CAGAACAATAATAACCCTACTAACAGTATGACCAACTTGACTAGCCATTTCAATAGT
AGTAGTAATAATATCAAAGTAGAAAATTTTGGAGGGGTGGGGAATTATTGGGAAGG
AGATGAGCTAAAAGTTGGAGAATGGGACTTGGAAGAATTGATGAAGGATGTTTCA
CCTTTCCCTTTTCTTGATTTCCAAGTTGAATAA

>Peaxi162Scf00006g00098.1

ATGGCAAGGACACGTTGCTATGACAAAAGTGGCTTGAAGAAAGGTACTTGGACTC
CTGAAGAAGATAGGAAATTAGCTGCTTATGTTACTAGATATGGTTGCTGGAACTGG
CGTCAACTTCCCAAATTTGCTGGATTAGCAAGGTGCGGGAAGAGCTGCAGACTGA
GATGGCTGAACTATCTCCAGCCTAATATCAAAAGAGGAAATTACACCAAAGAAGA
AGACCAGATCATTATGATGCTTCATGCAGAAATTGGGAATAAATGGTCAGCCATTG
CTGCTCACTTACCTGGAAGATCAGACAGTGAAATTAAGAATCACTGGCACACTTCC
CTCAAAAAGCGATCAACTCAAGAAGAATCAACTTCAAGTACTAACTCAAAGAAGA
GATCACCTATTAACAGTAAATACATCTCCAGCAGTCGAAAAAAGAGAAGTGGAAG
TGGAAATCAAATAAGTGTGAAGTCTAATGTTCCTCAATTGTCACCAAAGCAATCAT
GTAGTGAGGTTTCCTCTTGTGCTTCAATACATGTGGAAAGTATGCATGTAGAATCAG
AGGGTTATCAAGAAGAATATTTATTTGAGGAATCAAGTGGAATAAGCTTTTGGACT
GAACCATTTTTAGTAGATAATTCTAGTACCACAAATGATTTTTTTGTGCCTTCAGAA

CTTGACCATGGGCTTGTATCTCCATTTTCTCCTGTTATCAGTTTTGATGAATTTATAT
GCTCCTATGATTAA

>Peaxi162Scf00007g00207.1

ATGGTGAGAACACCTTGCTGCGACGAAAATGGACGGAAGAAGGGGACATGGACT
CCTGAAGAAGACAGGAAGTTAGCAGCATATGTTACTAAATATGGTTGCTGGAACTG
GCGCCAACTTCCCAAGTATGCTGGTGAGAATTTCTGCACAATTTACACCAATATTG
CTCCTCGTGAAGGTCTAGCAAGATGTGGAAAGAGCTGCAGACTTCGATGGATGAA
TTACCTAAGACCCAATGTTAAAAGAGGTAACTACACCAAAGAAGAAGACGAGATC
ATCTTGAAGTTGCATGCTGAACTTGGAAATAGGTGGTCAGCTATTGCTGCTCAGTT
GCCAGGAAGATCAGACAATGAGATAAAGAATCATTGGCACACCTCCCTTAAGAAG
CGTGCTAACTATGGGCCAAACTCAAGTGAATCAAGCAAGAAATGCAATAAGAATA
GTGAAAGTAAGAGAAAGAGAGTGGAAAATCAAAATGCAAGTCATGAAACAATAC
TGGAAAGTTCCCATATGTCACCAAAGCAGTCAATAGGTGAAGAACTCTGCTCTAAC
ACCACAGATTATAAACAACAGGATGTCGCGAGCGTCATTAGAGAAGAGGAATTATA
TTACGAAGAAGCTTTGGCAGAAATTAGTGGAAACTTTTGGACAGAACCATTTCTAC
TGGATAGTTTCAGCAATAGATTTGATTTTCGAGCTCCTTCAATTGATTGTGGACTTG
TGTGTCCACCTTCTCCTTTTATAGGCCATGAACTTCTTTCTTCGTTTGACTTTGATGA
TTCTAATTGGTGA

>Peaxi162Scf00007g00823.1

ATGGCCCCAGATGATAGAGGAATGAAGAATGGTGGGGCATCAACAGGAAGATCAA
ATGGAGCTGGATCAAGTAGACAAGTATTAAAGAAAGGGCCATGGACAGCAGCAGA
AGACTCGATTTTAATGGAGTACGTGAAAAAACATGGAGAAGGGAATTGGAATGCT
GTTCAAAGGAATTCTGGATTAATGAGATGTGGAAAAAGTTGTAGATTAAGATGGGC
TAATCATTTGAGACCAAATCTTAAGAAAGGTGCTTTTACTGTTGAAGAAGAGAGA
ATTATTATTGAACTTCATGCTAAACTTGGTAACAAATGGGCTCGCATGGCTGCTCAG
TTGCCTGGAAGAACAGACAATGAAATCAAAAATTACTGGAACACAAGGCTAAAGC
GAAGACAAAGAGCTGGTTTGCCCATATACCCTCAAGAATTACAACAACAAAATCA
ACATGAAAATAATAATCAACCCCACAGTCTTCTTTCTTCATCATATGATCCTCAAAA
TTCGACTAATTACAATTCTCCTTCTCTTTCCCTTTTGGATATCTTCAATCCTTCCACC
ATGAAACCTTCAATTACACAACAATTCCCTATTAATACTCCTTCTCTATGTCTGCCA

AGTACCAATAATAACAATATTTTTCGCAACACTCCCAAAGGTCTTTCCTTAACATTG
CCATCATCTATGAGAAATTCACAGTTTTCTTCATTGCCTAATAACAATTTTACCCAA
GGCCTATCATCCAATTCAATACAAGTTCCGCCGTTTCAACATAACTATCCAAATTTT
AACATAAATAGGCCATTTACGGGGATTTCTTCGAATCCAAATGGATTAATCTGCGGT
ATGGGCATTAATACTATTAATTATCCATCAGGGCAATCATCCATGCCTGTAACAGCAT
CATCTTCTGAGAATACAGGCAGCGATTTTGGTTCAAGTGATAATGCTAATAATTATG
CAAATACTAATGGATTATCGCGGGGAAATAGTGGATTATTAGAAGATTTATTGGAGG
AGTCACAAACTTTGAACCGGCCTGGTATGAAAATAGAAGACAATTTTCTTGATTTA
AAAGAGGATCAAGAAGCTGATTATAAAGGAAAATCAATGTTGTGGGAAGATTATG
GATTAGTGGAAGATGCTGAAGAAGCAATTTTAACTGAAGAATCAGCTTATAGCTTT
GCTCATGGTGTTGATCATGTCGCACAAAATAAAAATTCTGAAAGCTCTAGCCCTCA
TAGCCCTCCTAATTCATCTTCAGGGATATTCATGAAAAAGGAAGATTCATTTCATGG
AACTAATCAAGCAGACGACGATATAATGTGCCTTCTTGATAATTTTCCATTAGCTGT
ACCAGTTCCTGAATGGTATGAAGATGAGGATGACAAAAATAATTGTAATGGGCAAT
CGTCAAACGTGACCAATTGTGATCACATTGCTGAAAATCAGGCTGAAGATTCCAA
GTCACCAGCTTTGACATTAAATTCAGGGACTAGGAACCATGATTGGGAATTTGGAG
GATGTTGCTGGAATAATATGCCTTCATTTTGCTAG

>Peaxi162Scf00007g02122.1

ATGGTGAGAACACCTTGCCGCGACGAATATGGAAGGAAGAAGGGGACTTGGACTC
CCGAAGAAGACAGGAAATTAGCAGCATATGTTACTAAAAATGGCTGTTTGAACTG
GCGCCAACTTCCCAAGCATGCTGGTCTAGCAAGGTGTGGAAAGAGTTGCAGACTT
CGATGGATGAATTACCTAAGACCCAATGTTAAAAGAGGGAACTACACCAATGAAG
AAGACGGCATCATCTTGAAGCTGCATACTCAACTTGGAAATAGGTGGTCGGCCATT
GCTGCTCACTTACCTGGAAGATCAGACAATGATATAAAGAACCATTGGCACACTTC
CCTTAAGAAGCGGGCTAATTATGAATCAAGCAAGAAATACAATAAAAATAGGGAA
AGTACTAATACGAAGAATCAGGCCAGCAGTAGAAGAAAGAGTACTGTAGAAAACG
AAAATGCAAGCTCTCTAAGCTGTAGTATGAGTACTCATGAAACTATACTAGAAGGT
TCACAATGGTCACTTAAGCAGTCATCAAGTGAAGAAATCTCCTCTTGCACCGCAGA
TTATCAACAACTGGATATTGCGAGTGACATTAGAGAAGTTAAAGTTTTTGAAGAAG
AAGCTTATGTGGCAGAAAGTTGTGGAAGCTTTTGGACAGAGCCATTTGTAGTAGAT

AGTTTCAGCAGTAGACTTCATGATTGTGTAGCGCCTTCAATTGATTTTGAACTTGTG
TCTCCCCCCTCTCCTTTCATAGGTCATGGATTTATTTCCTCGTTTAACTTAGATGATT
ACCTCATGCTATCATCGCTGTTTGACTAG

>Peaxi162Scf00007g02125.1

ATGGTAAGAACACCTTCAATTGATAAAAATGGAATGAAGAGAGGTGCATGGAGTG
AAGAAGAAGACAACAAACTTAGAGCTTATGTTGAAAGATTTGGCCATCCCAATTG
GCGAAAATTGCCTAGATATGCTGGTCTAATGAGATGTGGGAAGAGCTGCAGATTGA
GATGGATGAATAACTTGAGGCCAGGTTTGAAGAAAGGGAATTATAGTCATGAAGA
AGAGCAACTCATTATTAAATTACACAACCAACTTGGAAACAGGTGGTCAACCATAG
CAGCAAAATTACCAGGAAGATCAGACAACGATATCAAAAACCACTGGCATGCTCA
TCTCAAGAAACGTACAAAGACAAACGCCAATTCATCAACAATGGAACAAATGAGT
ACTGAGTCTTCACAATCTGGATGTCAAGCTGAGCAATCTAACTCTAAACTTCCAGA
ACTTGAGAACTACTGTGATCCTATTGACACTTCATCTCCTCCAGAGGTTTCATCAAG
TGATTTATCAAAGTTATTTTCCAGTTGTTCCCTCTTAAATGGAATGGATTGGATTGA
AGATGATCAGATCAGGTCAATGGAACAACTATCAAATATTGATTCAGTGGAACCAC
TTCTAGACTCCTTTAGTTGCTGGACAAAGCCCATTGACAACTTTCAGACAGAACAT
TGCTTTGACAATGTTTGGTCCGAACACTTTGATAATTTCTGGACACAACCTTTCCTT
TAA

>Peaxi162Scf00007g02128.1

ATGGTAAGAACTCCTTCTGTAGACAAAAATGGAGTGAAGAGAGGTGCATGGACTG
AAGAAGAAGACAACAAACTTAGAGCTTATGTTGAAAGATTTGGCCATTGGAATTG
GCGACAATTGCCTAAATATGCTGGACTAATGAGGTGCGGGAAGAGCTGCAGACTG
AGATGGATGAATTATTTGAGGCCTGGTTTGAAGAAAGGAAATTATAGTAATGAAGA
AGGGCAACTCATTATTAAATTACATAACGAACTTGGAAACAGATGGTCAGCAATTG
CAGAAAAATTACCAGGAAGATCTGACAATGATATCAAAAATCAGTGGCATTCTCAT
CTTAAGAAACGTGCCAAAACAAATTCCTATTCATCAACATTAATGGAACAAATGAG
TACTGAATCTTCTCAATCAGGATCCCAAAGTGAGCAATCTTGTAAACTTTCAGAAC
TTGAGGCTTGTGATAAGAAAGAAGTGACTTCTGCTGCTATAGATCGTCTAGATTCA
GTACCACCAGTGTCATTAGAGGTTTCATCAAGTTCCCTTTTAAATGGAATAATGGAC
TACTGGATGGAAGAAGAAGATCGCATGTTCAGGCCGTCAATGGAACTACTTTCAAT

AATGGTGAGAACTCCTTCCATTGACAAAAGTGGAATGAAGAGAGGTTCATGGAGT
GAAGAAGAAGACGACAAACTTAGATCTTATGTAGAAAGATATGGCCATCCCAATTG
GCGACAATTGCCTAGATATGCTGGACTAATGAGATGCGGGAAGAGCTGCAGATTGA
GATGGATGAATTACTTGAGGCCAGGTTTGAAGAAAGGAAAGTATAGCCATGAAGA
AGAGCAACTCATCATTAAGTTACACAACCAACTTGGAAACAGATGGTCAACAATT
GCAGAAAAACTACCAGGAAGATCAGACAATGATATCAAAAACCACTGGCATGCTC
ATCTCAAGAAACGCTCCAAATCAAATACTAACTCATCAGCAATAATGATGAAGCAA
CTTATTGAATGTTCTCAATCTGAATCTCAAGACGACGAACATTCTAATTCTAAATTT
GCGGAACATGACAGCTTTTCTCCTATAGATTCAGTACAACCAGTGTCCTCAGATGT
TTCATCGAGTTCCGTTTTAAATGGGATGGACTGCTGGTTCGAAGAAGATAATCACA
TATTCAGTTCAATGGAACCATTGCCAGACTTCTTTAACTTTAGCTGGACAAAACCC
ATCTACAATTTTCAGACAGAACCCTTTGATCATTATTGGACAGAAGCCATGGATAAT
TTTTGGACAGAACCATTCTTTTAA

>Peaxi162Scf00007g02414.1

ATGGGAAGATCACCATGTTGTGATAAAAATGGACTCAAGAAAGGTCCATGGACAC
CTGAAGAAGATCTTAAACTTATCCAATATATTCAAGTTCATGGTCCTGGAAACTGGC
GAACTCTCCCTAAGAATGCTGGACTTCAAAGGTGTGGAAAGAGTTGTCGTCTTCG
TTGGACTAATTATTTGAGACCTGATATTAAGAGAGGAAGATTCTCTTTTGACGAAG
AAGAAACAATTATCCAACTTCATAGTGTTCTTGGCAACAAGTGGTCAGCTATAGCA
GCTCGTTTGCCAGGAAGAACGGACAATGAAATCAAGAATTATTGGAACACTCATAT
TAGAAAAAGGCTTCTAAGAAATGGTATTGATCCAGTGACACATAGTCCCCGTCTTG
ATTTATTAGACTTATCATCTCTTCTAAATTCCACTCAGTTTAACCTATCGAGCTTACT
TGGACTACAAGCACTTGTAAACCCTGAAGTCTTTAGACTTGCAACTACTCTTTTGG
CATCACACAATAATGAAAATCCAGAGTTGTTACTTCAAAAACTTCAAGAAAACCA
ATTGTTGAACACTCAACTTCAACAAAATCAATTATTGAATACACAATTGGGAAATC
ACCTTCAAGTTTTTCAACCTAATAGTCAATTCCAAAATCAAATTCAAGAAATACCA
ACTTTCACCCCATCAAATGTTCCTTGTTCATCTTCACAGCCCATGCAGCTAGGACCC
GTGGAATTATCCTATTTGATGAATGGCCAAATGCTACCTCCACAATATGGTTATTGTG
CTTCTAATGTATCCGACAGTTCCAATTTGCAATCACTAAACAACAGCAACAATCAG
AATAGCAGTAATTTCAGCTTGGATTCGGTGTTGTCAACGCCTTTGTCTAGCACAGA

AGATGAGAAAGAAAGCTATTGCAGTAATTTAATGAAATTCGAAATCCCAGCCAGTT
TAAATTTTGATGATTTTATGTAA

>Peaxi162Scf00008g02024.1

ATGGGAAGAGCACCATGTTGTGATAAGAAAGGTTTAAAGAAAGGACCATGGGCAC
CTGAAGAAGATGAGAAACTTATTGAATATATCAAGAATAATGGTCATAATGGTTGGC
GTTCACTTCCAAAGTTAGCAGGTCTCCTTCGATGTGGTAAAAGTTGCCGTCTTCGA
TGGACGAATTATCTGAGGCCAGACATCAAACGAGGTCCGTTTAGCGAGGAGGAAG
AAAAGCTTATTATACAGTTACATGGCATTCTTGGCAACAGATGGGCTGCAATAGCA
GCACAACTTCCTGGAAGGACGGACAATGAGATCAAGAACTTGTGGAATACTCACC
TGAAGAAGCGCCTATTTTCCATGGGCATTGATCCTCATATTTATGAACCATCATCTG
CGCCTAGTGGGCTGGTTAGGAGACCACCTGCGTCATCTTCAACACGTCACATGGC
ACAATGGGAAAGTGCTAGGCTCCAAGCTGAAGCTCGTCTTTCTAAGGACTCACAT
TTATCGATTCCCTCTTTAGTAGGAAAGTCTCAGAGCGATTACATTTTGCGCATCTGG
AATTCTGAGATAGGAGAAGCATTTAGAAATTTCAAAGGAGTAGAAAAGACTACTT
CTGAATCTTCATCTCCAAAATGTGGATCAACTTCAAGCATCACAACCGAAATGGAC
ATTACTCTTACTGGTTCCTCAGTTGAAGGAACTAACCAACATAAAGATGCAGAGTG
GAAGAATTGTAAACTATATACTGGAAATATCTTGCGTGGATCAGACACATCTAGCTC
CAACGATTTTGAGGAATCTTCTGAATCAGCACTGCAACTTCTTTTGGATGTCCCTA
GTAAATATGACTTGAGCTTTCTAGGACAAAGTGATGCATACAGCATGTACCCTGCC
TTTTTAAGTGAAAGCTGCTTGAACTGCTCTTCTACAGAACATGAAGTTTGCTATGT
TTAA

>Peaxi162Scf00009g00022.1

ATGGAGTTTGATCATAGTAGTAATCAAAACCTCTCTCTTCAGCCATATAACAACAGC
TTGATACGTGGAGAAATGGATGATGTTTTTTGCACAGTCTCTAAAGATTATCTTCAA
GATTTTCATCACCTTGATCTTACCTTCAACAACCCTCATGATAATTTATTGATCGAAA
CGAATGGTTATGACTCGTTATTTGATCCAATCTTGAATGAGGGTAATCTCTCATCAC
TGGATCATCAAGATTTCAACTTGAATTATGAGTTCAAGCCATTTGAGCAAAATAGT
GCAAGTGGTAGTACACTAGTCATGAAGAATTTTGAGAATTCAATGGATATGCACAC
GTCGAATGATGAGAACCTTTTGTCATTGTGTTCTGAAGATATGAAGCCTCTCAGCTT
TGTTAATGTGCAGGATGAAAGTTCATGCATTACTGCTGATAATAACTATTATGATAAT

AAAATTTGTCGAAGAAAGAAGAATAAAAAAGTTAGCAGCAAGGAGAATATTTTAT
CTCCTTGTATGGGAAAACTAGGAAAAGGTAAAAAGAAGTCTAAATCAGCCAAAGG
GCAATGGACTACTGAAGAAGACAGGCTTTTGATCCATTTGGTAGAGAAGTTTGGA
GTTAGAAAATGGTCTCAAATTGCTCAAATGCTTAAAGGGAGGATAGGCAAGCAAT
GTCGAGAGAGATGGTACAATCATCTTAGACCTGATATCAAAAAGGATTTGTGGACA
GAGGATGAAGACAGGATATTAATCGAAGCTCATGCAGAAGTAGGAAACAAATGGA
CGGAAATCGCAAAGAGACTGCCTGGAAGAACTGAAAATTCCATTAAGAACCATTG
GAATGCAACAAAAAGAAGGCAATTCTCAAGACGAAAATGTCGAACCAAATGGGC
AAGGCCAAGCTGTCTCCTCCAAAATTATATCAAAAGTTTGAACTTCGAAAAGAGTA
TTAGGAGCAGCCATGACACAACTATTAATGCACCTAAACAAGAGCCTTTTGGCAAT
ATTCCTGATTATGATTTAACTAGTGAAGTCCCCGAATTTGCTTTTGATGATAAATTGT
TTGATGAGAATATAAACATGGACGCTTTAATGGATCAAATTCACGACAGATGCTTG
GATTTGGAGATTCCTTATGATGAACTGCCCAAGCTGATGCAAGGTGATGCCAATAA
GGAGCCTGACTCGATAGATTTGATTTCAGAGATA

>Peaxi162Scf00009g00621.1

AGTCAGAAGGTCAGGTGGAGTAATCGTCTGAAACCCGATCTTAAGGAGGACAACT
TCACACCCCAGGAAGAGGAATTGATCATCAAGCTTCATGCCACTATAGGAAGCAG
GTGGTCCATAATAGCACAACAACTACCTGGTAAAACAGACAATGATGTGAAGAAC
TTGTGGAACACTAAGTTGAAAAAGAAGCTCTCAGCAATGGGAATTGATCCTGTTA
CCCACAAGCCTTTCTCCCAAATACTCACCGACTATGGAAACATTGGTGGCTTCCCA
AAAGCCAGAACTCATTTTGTGTCACTTAACAGAGAACTAAAGGGCGCATTTATGTC
TAGACCAGAACAATTTCAACATCCTCTAGAAATATTTTCAAATTTCAACAGCCATTG
TTTGACAACAATTAAGTTACCAAAAGCTGAAGCTTCAGAAGATTGTTTCTTAAGCA
ACACCAATGATTCTAGCAACAACCAGCCTCCTGTTGATCTACTTACCGAACTACAA
GCCATAAAATTTGTGACAGACGCCTCTAATTACAGTTCCCCCAAGGCAATTTTCTC
CCGTTCCAATCCCATAAATGACTACTCATCGTCTCCACTGTCATCCTCATCATCATCA
TCTGCATCTCATTCATTGAGCAAAACTCAAGTGGAAAGAGCATCCCCTGGCTTGAA
CTGGTGTGACTATCTTCTTGACGATGCCTTTATTCCATCAAATTTCCAAGTTCAAGA
AGACACATTAACCATAGAAGACAAGCTAGCGTCTGGGGCGGCTCAAGATGGACAA
AGCAACATGCCATCAATGAAGGATTCTGAAACCTCATTATCTACAAGGGGTGCATC

CTCATCATCATCATCATTTGTTGAAGACATGCTTGAATGCGAAAATGAGATGTTCTT
GAACTTCCCTGGACTCTCTGAGGATCCGTTTTACTAG
>Peaxi162Scf00013g00223.1
ATGGGAAGGCCACCTTGTTGTGATAAAATTGGTGTCAAGAAAGGACCATGGACAC
CAGAAGAAGATATCATTTTAGTTTCATATATTCAAGAACATGGTCCTGGAAATTGGA
GAGCTGTTCCAACTAATACTGGTTTGCTAAGATGCAGCAAAAGTTGTAGGCTTCGA
TGGACTAATTATCTTCGGCCAGGGATCAAACGTGGAAATTTCTCTGAACATGAAGA
GAAGATGATTATCCACCTCCAAGCTCTTCTTGGCAATAGATGGGCAGCTATAGCATC
ATACCTTCCACAAAGAACAGACAATGATATCAAGAATTATTGGAATACTCATTTAAA
AAAGAAGCTTAACAAGAAGATGGAAGGCCATGATCATGAGAATATTAATGAAGTG
GGAAAATCATCATCATCATCTCAATCAAATGTCCCAAAAGGACAATGGGAAAGGA
GGCTTCAAACAGATATTCACATGGCTAAACAAGCACTTTGTGAAGCTTTATCACTT
GACACACCTTCAAATGTTGATTCTCCAAATAATAACAATAATCCAACTCCAAAAATT
CTTCCTGTTGATCAACAACCAGTCCAAACATCTACCACCTATGCATCTAGTGCTGA
AAATATTGCTAAGTTGCTTGAAAATTGGATGAAAAATTCACCAAAGTCATCCGAAA
GTCGATCTAGTTCAAAAACTACTCAGATGTCATCCTTGAACAATTTATCAATTGGTG
CAGTTTCAAGCTCTAGTCCTAGTGAAGGTACAAGAAATGTTTTGGACTCAGTTTTT
AGCTTCAATTCATCCAATAATTCGGATGTGTCACAATCCATGTCTATCGATGAAGGT
GGTAATTTCACACCTGAGAATAACAATGCTACAATTTTCCAAGTTGAAAGCAAGCC
AATTAATTTGCCTAATTTCAAGGAAGACAATGGAATTTTTCAACAGGTGAGCAAGC
CAAATTTGGAGACACAACAAGTGCCATTAACTTTGCTAGAAAAGTGGTTACTTGAT
GATGCAAATGCACAAGCTGCACAAGAAGAGTTTATGGGAATTGGAATGGGAATGA
CTTTGGGTGAAACTGCTGATTTGTTTTGA
>Peaxi162Scf00013g01023.1
ATGGGTAGAACTCCATGCTGTGAGAAACTAGGGTTGAATAGAGGTCCATGGAGTA
AGAAAGAAGATGATATACTCATAAATTACATTGATAAACACGGCCACCCTAACTGG
CGTCAACTTCCCAAACTTGCAGGTCTATTGAGGTGTGGAAAGAGTTGTCGTCTCCG
ATGGACTAATTACTTGAGGCCTGAAATTAAACGAGGAAACTTTACTCCTGAAGAGG
AAGACACCATTATCAAGTTGCATCAAGTTCTTGGCAACAGGTGGTCTGTAATAGCA
GCAAAATTACCAGGACGAACAGACAACGAAATAAAAAACATTTGGCATACTCGTC

TGAAGAAGAAAATGAATCAGTCTCAGATTCAAGAGAAAACACCAGACATACGAG
AACAACCAAGTGAAACATCAAAATCCGAGGATTCAACAAATATACAAGAAAATAC
TGAAATATCAAGTCCTAAACCAAATTCCAATAATCAACACGAACCAAGTTCATCGT
TACGTTCATCGTCTTCAATAACATCATCAAGTGAAGGTTCATGTTCAAACACAACC
ACAACGAGTTCACATATTGACGAGTCTCGAGACCAAATGAACTTGGAGAATTTGCT
TGAAGTTGACGACAATTTTTGGTCAGAAGTACTATGGGCAACACCTGCAGATGATT
CTAAGGACAATAATCTTGATTTTTCATTATCTTCAGTGGAGAAAAATTACGAGCTTG
ACTCCAGCTTGAATGATAACTGGTTATGGGATGATCTCTTTTCAAGAGCCAACGAG
TTTTTGCTAGAATTCCCTGAATTAGTATGA

>Peaxi162Scf00015g01036.1

ATGACATCCTTGTCTAAAAGTTCAAGTAGCTCTAGTGATGATGATATTGGGTTGAGG
AGAGGTCCATGGACTGTTGAAGAAGATACTCTCCTTGTCCATTACATTTCCCATCAT
GGCGAAGGCCGATGGAATATTCTTGCTAAACGTGCTGGACTAAAGAGAACAGGGA
AGAGCTGCAGGTTGAGATGGCTGAATTACTTGAAACCGGACGTTAAACGAGGAAA
TCTCACTCCACAGGAACAACTCCTGATTCTTGAACTTCATTCCAAGATTGGGAACA
GATGGTCAAAAATAGCGCAATATCTCCCAGGAAGAACCGATAATGAAATCAAGAA
CTACTGGAGAACAAGGGTGCAGAAACAAGCCAGGCATTTGAAGATCGACTCTTCC
ATTTCAGAATGTTCCAATTTGATTCCACCTGGAGATTCCGGCTTAAATACATTTATC
AAGGCTCATTTCCCATTGGACGGCGAAAGTTATGATATGGATACATTCAGTCCAGC
AACTTGCAGTTTTAAAGATGTGCTATATTATGATCAGATGAGTGGAGAAAACAATG
CGCCTGGTGATGTTTTAGCTGACAGCTTTTGGAGCATGGACGAATTTTAA

>Peaxi162Scf00015g03118.1

ATGGTAAGGGCTCCTTGTTGTGAGAAGATGGGGTTGAAAAGGGGTCCATGGACTT
CAGAAGAAGATCAAATTTTGATATCTTTTATTCAAAGATATGGTCATGAAAATTGGA
GGGCACTTCCTAGACAAGCTGGTTTGTTAAGGTGTGGAAAAAGTTGCAGACTTAG
GTGGATAAATTACTTAAGGCCAGATATTAAACGAGGAAACTTTAGTGAGGAAGAA
GAAGAAACCATCATTGAGATGCATCAAGTTCTTGGAAACAGATGGTCTGCAATTGC
ATCAAGACTTCCTGGACGAACAGATAATGAAATAAAGAATTTCTGGCACACTCACT
TGAAGAAAAAATTAGAACACAATGATCTAAGAGCTACTACAACTGCCAAGAGAAG
CCCTCATGAAATGATACCTCGATGGAAAATAGAGCACCATATTTCTTCAAATTATCA

AGCTTCTCAAAATATTGCCAATCAATATCCAACTTGTCATGATGAAGATCTTCAAGA
AAATAACTCAACTTCTGACACTCAAGCAAACAAGGAGGAGAGCATGCAACCTACT
AATACTCAAATTGGACATGACGGGGACGCTAGTATTAGCAATGATATG
>Peaxi162Scf00018g00228.1
ATGGGAAGAACACGTTGCTATGACAAAAGTGGCTTGAAGAAGGGGACTTGGACTC
CTGAAGAAGATAGGAAGTTAACAGCTTATGTTACTAGATATGGTAGCTGGAACTGG
CGTCAACTTCCCAAGTTTGCTGGATTATCAAGATGTGGGAAAAGCTGCAGACTGA
GATGGCTGAACTATCTCCAGCCAAATATCAGAAGAGGAAATTACACCAAAGAAGA
AGACGAGATGATTATGAAGCTTCACGCAGAAATTGGGAATAAATGGTCAGTCATTG
CTGCTCACTTACCTGGAAGATCAGACAATGAAGTTAAGAATCACTGGCACACTTCT
CTCAAAAAGCGATCAACACAAGAAGAATCAACCTCAAGTACTGGCTCAGAAAGG
AGATCTAATAACAGTAAATACATCTCCAGCAGTCGAAACAAGAGAAGGGAAAATG
GAACTCAAATAAGTGCAAACTCTAATATTCCTCAATTGTCACCAAAGCAATCATGC
AGTGAGCTTTCCTCTTGCGCTTCAGTTGATCAACATGTGGAAAATATGCACGCAGA
ACGCGAGGTTTTTCAAGAAGAATATTTATTTGAGGAATCAAGTTTAATAAGCTTTTG
GACAGAACCATTTATAGTGGATAATTTTAGTACCACAAGTGATTTTTTTGTAACTTC
ACAAACTGACGGTGGACTTGTATCTCCATTTTCTCCTTTCACCCGTGTAATTTGGGT
TATTTTTCAATTTTGTGCATATCACCCATGGCACCTGGATGGGACACCGGACAAAC
GTGATATTTCCCTTGCTGCTCTTGTTGTGTCAAAATCAAAATCTCTTGTAGAATCAT
TTGACACAAAGCTGAAAGAGAACATGATGAATGATGAACCAAAAAAATTGATAAT
AGAGCTTAGCGTGCTAAAGATGAAGAAATTGAGAAACCATTGTCGCCCATGTGCA
AGGGCGGTCGACCCAAGAGAGAGAAGTGAAAGAAATAATGGAGGAAGGCTTGAA
AAAAGGAGAGCACCTGTCATGAATAGGCAGTCGAACAGGTGGCCGCCTCATGCAT
AA
>Peaxi162Scf00020g02349.1
ATGGTTGGAATGATGGGATGGGGAGCTAATTCTGATCAACAAGAATGGAGGAAAG
GACCTTGGACTCCTGAAGAGGATAAGTTGCTATCTGAGTATGTTAACTTGCATGGT
GAGGGAAGATGGAGTTCTGTATCTCGGTGTGCAGGATTGAATAGGACTGGGAAGA
GTTGCAGGCTAAGGTGGGTGAACTACTTGAGGCCTGGACTAAAAAGAGGTCACAT
AACACCTCAAGAGGAAGGAATTATTATTGAATTGCATGCCTTGTGGGGTAACAAAT

GGTCAACAATAGCCCGTTACTTACCTGGAAGAACAGATAATGAGATAAAAAACTAC

TGGAGGACACATTTCAAGAAGAAAGAAAAGGCTTCTTCAAAGCAAGACAAGAGA

AAAACTCTAAGGCAGAGAATTAATAACCAACCGCAACTACAACATGACACTAACT

ACAAATTTTCCCCTCAACCAGAAGAAGTGATTATGCAGAATAGCGATGAAATTAAC

GAGCACAACGACACGTTAACGTTCACTTATCATCCGAATATGGAGAATACGATCGT

GGATTTGCCACCAGTGAGGACTTCTTCAGACATTTCATATATATGGACTGACAATTT

TGTGATGGATGGACTATGGGGAGGATTGTGGAACCTAGATGTGGATGATCATTCAC

AGCCAGCTAGTGACAAGTGCAAAGTGGCTATCCAAAATCAACCAACTGATTATCA

A

>Peaxi162Scf00029g00131.1

ATGGTCCTTGGAGATGAGAAAGAGGGTATGCTCCGGAAGCAGGAGAAGAAATCCA

AGAAAAGCAATAAGAAAGCTGCCATCAATGGTCTAGCATTGACAACAGCTGATGT

TAAAGCTATAAAGGGTGATTTTAGTTTCGATGAAGAGAAGCTAACTGTTGTGGAGA

CGCTGATACAAAGGGATAAGGAAGAGATGGAAACTAAAGCAGCAGGAGAAATTG

ATCAATGTGGCGTTAAGAGAAAGAAAAAGAGAGGTGGAAATCATCTTCGCAAGTC

CAACAAGGACAGTGATGAAGATGCTGCGGAAGGTGTTGAAGAGAAGAAACATAA

GCTGAAGAAAAGCTGCAAGGATGAGCAGCAGACAATGCAGTATGCTCCTTTAGAC

ACTGAAAAGAGTGCTCCTGCAAGTGCGAGTGAAACATGGGACAATGACAAGGTG

GTTGAGACCTTCGGCGTGGTCTCCAGTGGAAATGCTGTGGATGAGATTAGGAGGA

AGAATAAATCCAAGAAAAGCATTAAGACTTCTGCCACCAGTGGTGAAGTGACAAA

CACTGAAGTTAATCAAGGTGCTGTTTTTCCAAGTATAGAAGAGAGAGAAGAAGGA

AGCAGAACAGAAAATGGTAATATCAGAAAGAGGAAAAATGTAAAATTAGGACAGA

GACCTGAAGATCCTATACATGAAAATAGCGAGAAGAGAGTGAGATTCTCTGGTCAT

GTTCAGGTTTTCCCTTCACCCAATGATCCAAGTGACGAGAAGCATGAAATCGAGG

AAGAAACTTTATTGCGCGGCAAACGATTCTCAAAATTAGAAGATGAAATTATCAAA

AATGCTGTTCATAAATACATAGAGATACATAACATGGGTGAAGAAGGGCTCCAAAA

GATTTTGAACTCTAGATCTCATCCTGAAATAAAGGGCTGCTGGAAAGAAATAGGGA

GCGCTATACCTTACAGGCCTTATATTTCGGTTTATTATCGTGCACAGGTCCTGTTTCG

AAGGTGTGAGACCCGTAAATGGACTGAAGAAGAATATGAGATGGTACGAAAATTC

CACCGAGAACACGGGCCCAAGTGGAAGGTCTTGGCTGATGAACTTGGAAAACAT

CGGTTTCATGTGAAGGATACATGGCGAAGATTAAAATTGTCCAATCAGAAGAAAG
GTCAATGGACTCAGGAGGAGTACCAGACTTTGTTTGATTTAGTAAACACTGATCTG
AGACTGAAGCTTTCTGAAGACAAGAAATCTAAGTATGGGATGCTACGGGATAATAT
TCCCTGGAGTGCAATAAGTGACAAATTGTCCACGAGAACAGCTCCATCTTGTTGCT
TGAAATGGTATGATCAATTAACGTCTCCCATGGTAGCCAAAGGTGAATGGGCAGAT
GCTGATGACTATCACCTAATTGATGCACTTTTTGTGCTGGATGCTAGCTGCGTAGAG
GATGTAGACTGGGACAATCTTCTTGACCACAGGCCTGGAGAAATATGTCGAAAGA
GATGGAACCAAATGGTTCTTCACATAGATCAATGTGGAAACAGGTCATTTGCTGAA
CAAATAGAAGTTCTAGCTAAGAGATACCGTCCGGATCTGGCTGAAGTAAGGGAGA
CTTGGGATAGTAAACCTATCGTTCCATGA
>Peaxi162Scf00033g00251.1
ATGGCTCAAAGAAAAGAATCATTGGATCGGATTAAGGGTCCGTGGAGTCCAGAAG
AAGATGAGTTATTACAAAGGCTAGTTGATCAACATGGTCCAAGAAACTGGTCATTG
ATAAGCAAATCGGTTCCCGGTCGATCCGGTAAATCATGTAGGTTACGTTGGTGTAA
CCAACTTTCACCACAAGTTGAACATCGGGCTTTTACGCCTGAAGAAGATGAAACC
ATCATTCGGGCCCATGCTAAGTTTGGTAATAAATGGGCTACTATAGCCCGTTTATTAT
CGGGCCGAACTGATAATGCTATTAAGAACCACTGGAACTCTACTTTGAAACGTAAG
TGTTGTTCTGTTTCTGAAGATTTAAGCTTTGAAACCCCTGAACAACCTCCTTTAAA
AAGATCTTCTAGTGTTGGGCCCGGGCCCGGCCCGAGTAGCCCGTCTGGATCCGACT
TGAGTGATTCGGGCCTTTCTTGTTTCCTCAGCCACCGCTTGTTTATAGACCCGTTG
CTCGGCCCGGCCCGATTTTCCCACCACCCCCACCAGTTCAAATACCCAACCCACAA
AAGCCCGACCCTAAACCCGACCCGGTTGTTCAACCCGACCCGCCTACTTCTCTTTG
CCTTTCTTTACCCGGATCCGAAAACCAGGCTGTACAATCACCAACAACAGCACAG
CTGCCACCTGTTGCACCACTGCCAGCTCAGCAAGGTTATGAATATGGTGGTGCCAC
TTCAGCAACAGAGAAGCAGTTTTTACACCAGAGTTTTTGGGAGTGTTGCAAGAA
ATGATAAGAAAAGAAGTGAGGAATTACATGTCAGCAGGGTTTGTTGAGAAAAATG
GGATGTGCATGATGCAAACTGATGCAATAAGGAATGCAGTTGTGAAGCGTATGGGA
ATTAGCAAGATTGAGTAG
>Peaxi162Scf00036g00710.1
ATGGGCCATCACTGCTGCAGCAAACAGAAAGTAAAGAGAGGTCTTTGGTCTCCAG

AAGAAGATGAGAAACTCATTAGATACATCAGCAACCATGGCCACAATTGCTGGAG
TTCTGTTCCTAAACTAACCGGATTACAAAGGTGTGGTAAAAGTTGTAGGCTCAGGT
GGATAAATTACTTGCGGCCAGATGTGAAGAGGGGTTCATTTAGTGAGGCTGAAGA
AAGAACCATAATTGATGTTCATAGAATATTGGGAAACAGATGGGCACAGATTGCAA
AACATTTACCTGGTCGCACTGATAATGAAGTCAAGAATTTCTGGAATTCTTGCATCA
AGAAAAAACCTATTTCTCAAGGCTTAGATCCAAATACCCACAATCTCCTATCTCGC
AATAGTACTCAAAACAATAAGAATAATGCAAGCTGCAAAGTCAAGTCTACCTCAGT
TTTCACTCTTGAAACAATGCCATCAAGCAATAAAGAAGTTCCCATGGACATGATCA
AATCAAGTCTTGCAGCTTTTCTACCTCTCCCTCATAATAGTAAAACTATTTCTACCG
CTGCTTACAACGATAATATGAATTCCATTAATGTTCCCAGGACCACAACAAATATAG
ATTTTCCGAGAAGTTCTTTGATGGAGAGCACTTCAAATAATTATTTATCATCTTTAAC
TCCACCAGGGTATGGAATTATTAATTATGAGAATTATTGCATGTGGGCTGTTTCTGG
CGTTGAACGACATGACTTTGGTCCCTCCATTAATGGACATGAAGAAATGCGCATAG
TAGAGCAAGAAGGAGGACAAGTAGTGCAGCTTCAGGAAAAAGTGTTTCAGGCTG
AGGAAGTCTACAAGATTAATCATGATCAGTTCAACATTAATGGACAAAAAACATAT
GTCGACGATGCTAACTTTAGTTTTGAGTCTGTCGATTCTGCATTGATGATGCCTTAC
GGAATTTACACTAGCGTAAAGTCTATAGATCACTTTGCATGGAATTAG
>Peaxi162Scf00038g01640.1
ATGGGACATCATTCATGCTGCAACAAACAAAAAGTGAAGAGAGGGCTATGGTCTC
CTGAAGAAGATGAGAAACTCATTAAGTACATTACTACTTATGGTCATGGTTGTTGG
AGTTCTGTTCCTAAGCTTGCAGGTCTTCAAAGATGTGGAAAAAGCTGCAGGTTAA
GGTGGATTAATTACTTGAGACCAGATTTGAAACGTGGGAGTTTCTCCCATCAAGAA
GCTGCACTTATCATTGAACTCCACAGTATTCTAGGCAACAGGTGGGCTCAAATAGC
TAAACATCTTCCTGGAAGAACAGATAATGAAGTCAAAAACTTTTGGAACTCTAGC
ATCAAGAAAAAGCTCCTTTCTCATGGAACCCTTTCTGATCATCTCTCCATGTTTACA
AATTTCACTAATCCAAACCCTAGTCCCTCTTTTGATAATTTCTACACTCTGATCAATC
CCAATAATCCTGTTAATTTATTGCCAGCCATAAACTCTCCCCTTATTCTACAAGTTGA
CCAAATGAATAATGTTAATGAGGATTTGAATGCAAATTCAATGGCACCCTCCCTAAT
CCCTTTCTCATTTGACTCAATCCCAAATGATCCTTCATGGTTTTTAACTATCCTCAT
TCTCAACATGATCTTGATTATAAACAAGACAACTCCAATTCCATTGTCAGCTCGAAC

ATTTCTTTGAATTACACTGATGCTGCTGGGGGGATTTAATGGATTCAAGAAATGAT
CCTATAAAAAGTAGTACTCATCAAGATTTATTGGTGGTGCCTAATTTGCTACCTAGG
CTTTCACCTCCTGCTCCATTGCAACAGAATTTTGACTCAATGGTGGTTAATACTTCC
ACAAAT

>Peaxi162Scf00038g01818.1

GCACACGTAATGATGGATGATATACTAATAAAGAAAGGGCCATGGAAAGAGGAAG
AAGATGCAGTGTTGATAAAGCATGTGAAGAAATATGGGCCAAGAGACTGGAGCTC
CATTCGATCCAAAGGCCTTTTACACCGTACTGGCAAGTCTTGTCGTCTTCGTTGGG
TTAATAAACTTCGCCCCAACTTGAAAAATGGAGTGAAGTTTTCTGCAGAGGAGGA
GACAACTGTGATTGAACTTCAGGCACAGTTTGGGAACAAATGGGCTAGAATTGCT
ACATATTTGCCTGGAAGAACTGATAATGATGTCAAGAATTTTTGGAGTAGCCGCCA
GAAAAGATTAGCTAAGATTCTGCGGAACTCAGCATCGCAGCCTAGTACGCCACAA
AAGAATAACAACAAGGAAGCCCTTGCTCTTCAAAAAGTTCCCTCAGTAGAGGAAC
CAAAATTGAGTTCACCAGCAGAGGAAAGGTCTTTGACCATGTCCCAGTGTTGCTC
GTCATCCTATATGAATAACTCTGACACAATCAACATGGTCCCATTGCCAGAGCTAGA
GAATTCAACTTCACTTCCTTTTGAGCCAAACCTGCTGCAATTTGAGTTCACTCCTA
ATGACAAGAATTACCAGTACATTGAGACACAGATGTCACTCTCCTTTCCTCAGATT
CCACTCCAAACTGATCTTGGTCATCCATTGGGAAGTCAAGAATTACCAATGAAACT
TGAGGAGACTGACTTGTTAGATTTTTTCGGGCAACTCAGTACTGCTTCTGACATAG
GAAATGTGCAAGTCCCGCTTGTACCGTTGTGTTCAGGACCAGACAAAAGCTCTGA
AATTGTTGTGAAGAGAGAAATGGACAGCCCCCTTACACAAGATAGCTTCATCGATG
ACCTCCCCATGGATATGTTTGATCTTATTGATCCACTGCCGAGCCCATCCGATTGGT
GGAATAGTGATTTGTCTATTTCAGAAAGGAAAAAGGGAAATTGTAACTAG

>Peaxi162Scf00041g01811.1

ATGGGCAGGTCTCCTTGTTGTGATGAAAATGGCCTCAAAAAAGGACCATGGACTC
CTGAAGAAGATAAGAAACTTGTTGATTATATTGACAAACATGGCCATGGAAGTTGG
AGAGCCCTTCCTAAACTTGCAGGTCTTAATAGATGTGGCAAGAGCTGCAGGTTAAG
ATGGACTAATTATTTGAGGCCTGATATCAAGAGAGGCAAATTCTCTGAAGAAGAAG
AACAAACAATTCTTCATCTCCATTCCATCCTTGGAAACAAATGGTCCGCGATCGCG
ACACATTTACCAGGAAGAACAGACAATGAGATCAAGAATTTTTGGAACACACATT

TAAAGAAGAAGCTGATTCAAATGGGATATGATCCAATGACTCATAGGCCAAGAACT
GATTTATTTGCAAGCTTGCCTAATATAATAGCTTTGGCAAGTTTACTTCAGCATCATC
CACTAGAAGATCATGCTGTAAGATTACAAGCAGAAGCTGCCCAAGTAGCTAAACTT
CAATATTTACAATGGTTATTCCAATCTTCATCATCTAGTTCAAACAATTACCCAATAG
CTACAAGCTTAAACACTACTCAGTACAGTAATCTTGAAGATTTTGGACCATTTAATT
TGTCAAATTCTACAAAGGAAAGTACCCCTTCTATAAACTTATCCCTACTTGAAAATC
AAGCACTATTTTCCAATGAAAACTCTGGCTCTCAACTGCTCCACAATCCAGATACT
TCTCTCCCTTTGACAGACACACAAGAAGTCCCTTTCAATTTCCAAGCACATTTAAA
TAATAATAATAATAGTAATAATACAAGTGGTGATAATGGTCAAGACTTTAATTTTGAA
ATTTGCTCTCCAAGTTCCCCATTACATAATATTATTCCTTCTCCACCTTCTTCAAGTC
GTTTGCCACCATTAACTGAAATTTCCATAAGCAATAATCAAGGAGATGCTAGCAGC
ACTAATTCCAGCAATGCAGCTGAAGGGACTTCCTCATATTGGCCTGAGCTATTTTTT
GAAGACCATTTCATGCATGAGATTGCTTGA

>Peaxi162Scf00042g02519.1

ATGGGAAGGTCACCTTGTTGTGAGAAGGCTCATACAAATAAAGGAGCATGGACTA
AAGAAGAAGATGAAAGACTTGTAGCTTACATTAGAGCACATGGTGAAGGTTGTTG
GAGATCACTTCCTAAAGCTGCTGGACTTCTCCGATGTGGAAAAAGTTGCCGTCTCC
GATGGATTAATTACTTGAGACCTGATCTTAAACGTGGTAACTTTACTGAAGAAGAA
GATGAACTCATTATCAAACTCCATAGCCTCCTTGGAAACAAGTGGTCACTTATAGCT
GGAAGATTACCAGGGAGAACAGATAATGAGATAAAGAATTATTGGAATACTCATAT
AAGAAGGAAGCTTTTGAGTAGGGGTATTGATCCAACAACACATCGGCCTATCAAC
GAGCAGCCTACAATAAGTACACAAAAAGTGACAACCATTTCTTTTGCTGCTGACA
ATAATAAAGATGATCAAGATCAGAAGATTATTAATATCAAATCCGAATTCGAGACAA
CGAGCAAATTACAAGATGAAATTCAAGAAAGGTGTCCTGATTTAAATCTTGAGTTA
AGAATCAGTCCTCCTCATGATCATCAACAATTTAATGATCAGAGTATTGATGAGTTA
GGAAGAAGAAACTCTTTATGTTTTGCATGCAGTTTGGGCATACAAAATAGTAAAGA
TTGTAGTTGTAATACTAATGGAAATGGGTGTAGTAGCAATGTAAGTATGAACATTGC
AAGTTATGACTTTTTAGGGTTGAAGACTAATGGTCTTTTGGACTATAGAACTTTGGA
ATCT

>Peaxi162Scf00045g00138.1

ATGGGAAGATCTCCATGTTGCACCAATAACAACTTGAACAAAGGTGCATGGACAA

AAGAAGAAGATGAAATACTTGGGAATTATGTGAAAATTCATGGTGCAGCAAAGTG

GAATATTTTGTGTAAGAAAGCAGGGCTTAATAGGTGTGGGAAAAGTTGCAGATTAA

GGTGGACTAATTACTTAAGGCCTGATATTAAGCGTGGAAATTTCTCTGAAGAAGAA

GATAGTCTCATTATCAATCTCCATTCATCTCTTGGAAATAAGTGGTCCAAAATAGCT

TCCCAACTTCCAGGAAGGACTGATAATGAGATCAAGAATCATTGGAACACTAAGCT

AAGGAAGAAACTTAACAAATTAGGAATTGAACCGCAGACACATAAACAGATTCCT

AATTTCAACCTCTTTAACTCTCTTCAAATGCCTCCTCTATTTTCACAGCAAGATGTT

ACTCAATTAGCAAAATTACAGCTCTTGCATTTTCAAACTTTATTGCAGACCATCAAT

GCTATTACTACTACATTTCCAAGTAACCCTTTGATGCCAAATATTAATTATCCTATGC

AAGATGATGTGAATGGGATCTTGAATTTTTCCATGCCCAACTTTGAAGAGGATGAT

GATAATCTAAAGAAGGGGCCATGGACAGAAGATGAAGATGAGAAACTGATTGATT

ATATAAAGCAAAATGGCCATACAAATTGGCAATCACTCGCAAGAAAAGCAGGTTTA

AACAGATGTGGAAAGAGTTGCAGATTAAGGTGGAATAATTATCTTAGGCCTGATAT

CAAGAGAGGCAGATTCTCACCTGAAGAAGAAGAGATCATTATCAATCTCCACTCTG

ATCTTGGAAATAAGTGGTCAAAAATTGCTGCACATCTTCCAGGAAGAACTGATAAT

GAAATCAAGAACTTTTACAACACTCATTTAAGGAAAAAGCTTCTGAGATTGGGAAT

TGACCCCAAGACACACAAACCAATATCAAAACTCAATTCTCTTGTGAATCTTTCTC

ATCATCTTACCTCTAACTCAAACTATTTTTTATCTTTAGCATCTGTTCTTAGTTTGCA

AGCACAATTTTCATTACTAGCCAAAATCCAACAAGTCATACAAAGCCCCCTTCCAA

CTATAAATACCATTAATCTATTGTCATCCATTCAAGGCAATATTTTAAGCAATTCAAG

TCCACAACTCGAGAGCCTTAGAAATGAGACTGAAATGTCCAAATTCTTGGATTCCT

CTCTAGCAAACACTACAACAACACCATGTTTGGATAATACTAATTTA

>Peaxi162Scf00047g01824.1

ATGGCACCAAATGGTGGAGGAGTGAAGACATCATTAGCAAGAAATATTAGTCATGG

AGGGACAAGGCATGTATTAAAGAAAGGCCCATGGACAGCAGCAGAAGATGCAATA

TTAATGGAGTATGTGAAGAAGCATGGAGAAGGGAATTGGAATGCTGTTCAAAGAA

ACTCTGGTTTAATGAGATGTGGTAAAAGTTGTAGGTTAAGATGGGCTAATCACTTG

AGGCCTAATCTTAAGAAAGGTGCTTTTTCTTTAGAAGAAGAAAGGCTTATTGTGGA

ACTTCATGCTAAGCTTGGAAATAAATGGGCTCACAATGAAATCAAGAATTACTGGA

ACACAAGGCTAAAGAGAAGACAAAGAGCTGGTTTGCCTATATACCCTCAAGATATT
CAACCACAAAACCAACATGAACATAACATCTCTTCACTATTTGATCATCCTCAAAAT
TCCAATTTCATCAATCCCCCTCTTTCACTTCTAGATATCTTCAATCCATCAACCATGA
AACCTAGTGTTAATTCACAACAATATCAATTCAATAACCCTTCTCCATTTCTCACAA
ACACCAATAATAATAACAATCACTTCAAGCTTTTTCACGACCCTCGAGTTAGCCTTT
CTTTAACATTAGCTTCATCAATAAGGAATTCACAACTTTCTTCATTAGTCGCACCCG
TGCCTAAAACTTTTAGTCAAGGCTTAACCACTTCAATGCAAGTGCCACCACTTCAA
CATAATTATCCAAGTTTTAGCACCAATACAAGACCTTATACAGCAATTTCTACGAAT
CCAAATGGTTTAATCTTAGGCATGGGCATAGAGACTACAACATGTTCAAGAAGTAC
AGGAAATGATTACATGAAGGCAACATCATCAAGTGATGCTGATAATTATAATGTTGT
TGATCCTGGATTATCACGAGGAAATAGTGGACTATTAGAAGATTTATTAGAAGAGTC
TCAAACTTTAACAAGAGCTGAGAAAATAGAAGAGAATTGTCTTGTTGATGAAAAT
GAGGCAAGTCAAGGGAAATTAGTATGGGAAGAATATGGATTAACTGAAGAATCAA
CATATAGTTTTGCTCATGGTGGTGATGATACAACCCCAAATTCATCCTCAGGAATAA
CAACAAAGGATGCTTCATTAGAGCTGGCCAATCAAGTGGATGACGATATTATGAGA
TTTCTTGATAATTTTCCAGTAGCAGTACCAGCTCCTGATTGGTGTGATGATGAAAAT
GAGCAGAATAATACGTGTAATGGCCATTATTTGAACCTCGTGACCAAATGGATTAA
>Peaxi162Scf00050g00512.1
ATGAACCCTAGAGGAGAAGACTTTTTTGGTTTTCAAAAGCATCACATCACCCACTC
TTTCTACCTATATAAACAAGTCTTCTCTTCCAACATTTTTCAACCAACAATTAATCAC
AAGTTCAAGAAAATACCTTTTGGAAGCAAATTAGTAGAAGGGAAAAAAATATTAA
AGATGGGTAGAGCTCCATGTTGTGACAAAGCAAGTGTGAAGAAGGGTCCATGGTC
ACCAGAAGAAGATGCAAGACTTAAGGCTTATATTGAAGAACATGGCACTGGTAAC
AACTGGATTGCCCTTCCCCAGAAAATAGGGCTTAAGAGATGTGGAAAAAGCTGCA
GGCTTAGATGGCTCAATTACTTAAGACCTAATATTAAACATGGTGGATTTACTGAAG
AAGAAGACAACATCATCTGCAGCCTCTATATTAGTATTGGTAGCAGGTGGTCTATTA
TTGCTGCACAACTTCCTGGAAGAACAGACAATGATATCAAAAACTACTGGAACAC
TAGATTGAAGAAGAAGTTGCTTGGAAAGAGAAAACAATCTCAAATGAACAGATTA
TTGCTTGCTGGAGGTCAAGATCTTAAGGAAACAAATGGATTAGAAGAAAATTCCTT
ACTTCAAAACCTAAGCAACTCGGCTCTCGAAAGGCTCCAACTTCATATGCAACTCC

AAAGCCTTCAAAACCCTCTTTCTTTCTATAATAATCCAGCACTTTGGCCTAAGTTAA
CCCCTCTTCAACAAAAAATGATCCAAACCCTACAAGCTACTGGCTTAAGTATTGAG
AATCAATCATCTCTTCTCGCACAGATATCTCCTAGTGCAAACCAAGTTGATCAACTA
GGTCAAAAAGTTGGGATCAATGAATTTGCTAATACCATGAGTACTAGATTCAAGGT
GAATAATGAAGTTGAAAAAAGCACCGTGAATAATGGAATATCATCTTCAGATAGTC
CAATTGACTTTAGCAATCAAAAAGATGTTCTTGACACAAACATAGGGCAAGAAAA
CACGGGAGAAATCCAGGGTATTCAAGGGTTTACTCAAGCCGAGATCGATGATCTTA
TTCTCATAAACAACAAGGGATTAATAGCTGATCAATTTGATTGTTTCAAAGAGATG
GATGGATCCTCTAGCAGGGACAACTTGGCATGGTGGTCTAATGACTTTGATACGAA
TACAACTTCCTCAAATTCTTGGGGTTCCTCTTCCAATAATATTGTTCAGCAGACTGA
AGGGATGTATCAAGATTATGCACTAGGTTATAATTTGCAATGA

>Peaxi162Scf00056g00026.1

ATGGGGAGAAGACCATGTTGTGAGAAGAAAGGTTTGAAGAAAGGACCATGGACA
CCTGATGAAGATGACAAGCTTATTGAGTATATCAACAAGAATGGTCATGGTAGCTG
GCGTTCTCTTCCTAAACTTGCAGGTCTTCTTCGGTGTGGGAAAAGTTGTCGTCTTA
GATGGACGAATTATCTAAGGCCGGACATTAAACGAGGTCCCTTTAGCCATGAAGAA
GAGAAGCTTGTCATACAGTTGCATGGCATTCTTGGAAACAGATGGGCTGCAATTGC
TGCACAGCTACCTGGAAGGACAGACAATGAAATAAAGAATTTGTGGAACACTCAC
CTGAAAAAGCGCTTGCTTTCCATGGGCATTGATCCTCAGACTCATGAACCCGCCTC
TGCACCTAACGGACTGCTGAGAAGACCACCTGCATCACTTTCAGCCCGTCACATG
GCACAATGGGAAAGTGCTAGGCTCGAAGCTGAGGCTCGTCTTTCCAGAGAATCTC
AACTCTTGGTTCCATCGCCGGTAGGAAGGTCTGAGACTGACTATTTTTTGCGCATAT
GGAACTCAGAGATAGGAGATGCATTTAGAAAATTCAAGAAAGGTGCAAAGACTGC
TTGTCATAGTTCAGCCTCTCAAGCATCTTCGTGTACTAAGTGTGGCTCAGCTTCAG
GAATCAAGACTGAGGTTGAGCTCCGTGTTGCTGCTGGTTCCCCTGTTACGGGAAG
CAACCAAACTGAATTTGCAGAGTGGAGGAATGAACAACCACATACTGAAGATATC
TTGCAAGGATCAGACACCTCTAGCTCCGATGCAATTGAGGATTCATCTGAATCAGC
ATTGCAGCTTCTTTTGGATTTCCCCAGTAATAATGACATGAGCTTTCTAGGACAAAG
TGATTCCTATAGCATGTACCCCTTTTTGACTGCAAGCTCATTGATCTGCCCTTAA

>Peaxi162Scf00058g00238.1

ATGGAGGAGGTGAAAGCTACACAGGTAACATACAGTAGCGGTATGCCAGTTACAG
TGAAGCTCGCGGAGGACAACATAGAGATGAGGAAAGGCCCCTGGACTTTGGATGA
AGACTCCATACTCATCCATTACATCTCCCTTCTAGGTCAGGGACGCTGGGATTCGCT
TGCTCAATTTGCCGGGTTGAAGCGTAGCGGTAAGAGCTGCAGGTTGAGATGGTTG
AACTATTTACGCCCTAATTTACGAGGAGGAAAATTTACTCCTCAAGAGCAGCTTCT
TATTTTATTGCTTCATTTTCGCTTTGGAAATCGTAACGACTACTACCTTTGTTTTAGA
TGGGCAAAGATAGCAGAACATTTGCCTGGAAGAACTGATAATGAGGTAAAGAATT
ACTGGAGAACAAAAGTGCAAAAGCATGCAAACCAGCTGCATTGCAATGTCAACA
GCCAAGAATTTCGAGACGTCCTCCGCTACCAGTGGCTGCCCTCTCTGGCTGAACA
AATACGTGTTACACCATCGTCGTCTCTGCACGAATATCTTATGGCTTCTTCTTCTACC
GCTCACAATAAAACTACTGAAGTCAAGGAGAATATTTCTCCTTTTCTTGAAACTGG
GATTGGGTCCACTAGCATCACATCTCCGAATGTTGTCTCATCAACTAATTCCTCCGA
AGCTGAACTTCCACCACTGATGGACAATAGTCTCTGGAGCTTCTCTAGCAGTAACT
GGTGCTCTAGCTTTGAAGATGGATACTATCTTTATTCATCTCAGGATCAGTGTCACA
GTTGGGCAAGTGTGGCAGGATCAGAACTAGTACCAGCTAATGAAACGCTAGTTTC
GATGGGTAAAGATCTTGCATCACATGGCAACATCAGTTCCGAGGACACAAAGACT
TCTTATGATGAAGAAGTCAGAAGACAATGCATCCGGCCA

>Peaxi162Scf00065g00016.1

ATGAACAATCTTAAGCCAATTCTTGAAATTAGAGGCAACTTTGCTGAGGATGAAGA
TGATCTCATTATTAGGCTTCATGCCCTTCTTGGCAACAGGTGGTCACTTATAGCGGG
GAGATTGCCATGGCGCACTGGTGATGAAGTGAAGAACTATTGGAGCTCTCATTTGA
AACAAAAACTTAGGAACATGGGAATTGATCCAATGACTTATCGGATATCTGACTAT
GTTTGTAGGAAAAGCCACCTTGACTTGTGGCATGAAAGTAGAGAGACAGAAACA
AATGAAAAACCCTGTGATGCCAGAAGCTCTTGGGGACAAGATGACCATGAACCAA
GTTCCTTGCCCCATCTCAAGTCTTAG

>Peaxi162Scf00066g01813.1

ATGGCAGAAGGTGGTGGTTCTGGTGATGATACAAGAAGTAGTTGTCCACGAGGTC
ACTGGCGGCCGGCCGAAGACGAAAGGCTCCGGCAGCTTGTAGAACAATATGGTCC
TCAAAACTGGAATTCTATTGCTGAAAAGCTTCAAGGAAGATCAGGAAAAAGTTGT
AGATTGAGATGGTTTAACCAACTTGATCCTAGAATTAATAGAAGGCCATTTACTGA

AGATGAAGAAGAAAGACTTCTTGCAGCTCATAGAGTACATGGTAACAAATGGGCA
TTGATCTCAAGATTATTTCCAGGTAGAACTGACAATGCTGTGAAGAATCATTGGCAT
GTCCTAATGGCAAGAAAGCAAAGGGAACAATCCAAGATTTGTGGCAAAAGAAGTT
ACCCACAAGATAATTTTCTAAGTGATTCTAAATCACCATCCTATGGCTTTCGAAGAA
GGAACAACAACAATAATAATACTAATATTCCAAAAACACAAGAAGGTTACAACTAT
GGCACCAAATTCAGCTTATTTGAATTTCAAAACCCAACCAAAGATAGGGTTTTCTC
AGTATCCACATATTCTTCTTCCCCTTCCTGGAATTCACCTGATCAATTGTTTCGAAG
AAATGGTAGCCATTTGCTTAAAGAAAGCTCGAATTTTTCAGGCCAAAAATCTTTAT
GTCAAAATAATTTAAGCTTTTCAACCCATGGAGGAGGAGGAGCTACTCTATTTCCG
GTTCCTAATAATTACAAAAAGACTACTGCTCGAAACCCTTTTAGTTATTCAAATAAT
GGATCTGATGGCATGACTGAGAAAGTAATGAATATTAGCAATAGCACATTTTCCTTC
AACAAGATTCTCAGGGCGAGCATTGAACAACAACGACGACAACAACAACAACAA
CATGCAGAAGAAGCAATTGAAAAGAAGGATATTCCCTTCATAGATTTTCTTGGTGT
GGGGATTTCTTCTTGA
>Peaxi162Scf00071g00135.1
ATGGGGAGGCATTCTTGCTGTTACAAGCAGAAGCTGAGGAAAGGCCTGTGGTCTC
CTGAGGAAGATGAGAAACTTATAAAGCATATTACTAAATATGGGCATGGCTGTTGG
AGTTCTGTTCCTAAACTAGCTGGGACATTCTCACAGGAAGAAGAGAATTCCATCAT
TGAACTTCATGCACTTCTAGGGAACAAGTGGTCACAAATTGCAGCTAGATTGCCTG
GCAGGACAGACAATGAAATCAAGAATCTATGGAACTCTTCCATCAAGAAAAAGCT
AAGGCAAAGTGGAATTGACCCAAACACTCACAAGCCACTTTCTGAGATTGAGAAT
GAGCATCAGAAGGCATCTACAACCAATACAAAGAACAATAATAAATCAACTTCTGA
ATGTAACAGTGCATTCAATTCTGAGAAACCAAAGCCATCTTCATTGACTATTGACC
AAAGCTATCCACTCATTGAAACCAATATGAGCAGTAGTACTGGTTATAATCTCTCCT
TTCAACAGTTGAATTATGAGCCTAACACCACCACTATTGAATTGCAAAACAACTGT
AATTTGTTTGATAACAATAATGGTTTCTCATGGGGAGCTGTAGATAGTGTGAAATCA
GAGAAAGAAGCCCAAGAAATCAAATGGTCTGAGTATCTCCAAACTCCATTTCTACT
AGGCAACACAATCCAGCAGAATCATCAAAGTACTATAATTCCTCAAGACTTGTATA
GTGAAACAAGATCAGAAACACAGTTCGCGACACAAGGTTCATTGAGTGCTACGTG
GCTTCAGAGCCAACATCAGCAGCAACCTAATTCTTTACAAACTCCAGACCTGTATC

ATAAAAGTTTTCTGAGGCTTCCAGCTGCCTTTGGACAATATTCTTAG

>Peaxi162Scf00073g01731.1

ATGGATGAGATAAAGAAGGGGGCATGGTCTCCTGAGGAAGACCAAAAATTGAAAG
CTTATATCAAGAAATATGGCATTTGGAATTGGAGTCAGATGCCTAAATTTGCAGGAC
TTTCAAGGACGGGGAAAAGTTGTAGACTTCGATGGATGAACTATCTCAGTCCTGAT
GTTAAGAGAGGACCCTTTAGCATAGACGAGGTCGAAATAGTCATCAAAACGTACC
AAGAACTTGGAAACAGATGGTCAGCTATTGCAGCAAGATTGCCTGGAAGAACTGA
CAACGAAGTTAAGAACTTCTTCCACACACACTTAAAGAAGCATATGAGCTTGAAA
AATGATCATGCCCTGATGAAGAAAAAGGCAAGAAGCAAAAGGGTTATGAAGAAA
ACCAGAGAAAATGAGAAGACAAATACTGGCAAAGCTCAAGAAAGACCTGCAGTT
GAAGTAGTTCCAACAAAAATTCCAATGATAGGATCATCCAGCAACAAAAATTCACC
ATCACCTAATAGTTCTTCATCACAGTGCAACAGCATTATTACATGTCAAGAAAATCC
AACAGACCAAGATTGCTACAACAATATTATTAGTTCATCACGTGTTGATCAACCAGT
TATTATGAATATGGAAAGTCCATTTATATTGGAGAGCAACCCGGAAACATCTGAAA
GTAGTCTTGGTGATACTAATTGCAATCAGCTGATCCATCAGTTTGATCAGTACCCTC
ATGACTCGTGTAATACTATCTCCGATGGTTCGAGCTTCTTCGACTCCTTCGATCAGT
TCGATATGATAATTTCTTTGTTCTTAACTAGTCTTTTGAACTTTAATTTGGTCATGGT
CGCAGGTCTTTCAAGAACTGGGAAAAGTTGTAGACTAAGATGGGTGAATTATCTCC
GGCCTGATATTAAGAGAGGACCCTTTAGCATGGAAGAAGTCGAAATAGTCATGAA
AATGTATCAGGAATGTGGAAATAGATGGTCAACCATTGCTGCAAATTTGCCAGGAA
GAACAGACAACGAAGTTAAAAACTTCTTTCACACACACTTAAAAATGCATTTGGG
AGTAAAAAACGACGTCACAGTGAAGACTAAGGGAAGAAGCAAAAGGGTGAAGA
AAACCAACAAAAATGAGAAGAAACTTGCCGATAATGCTCAAGAAAGATCAGCTGA
TGTTGCTTCACCCTGCAGCAGCGTTGTTATATGTGAAGAAAATCACATGATGGATG
TTATGGATTTCTCAAAAACATACCAAGATTGTTACAACGTTAGTTCACTTGTTGATC
AATCAGTTATTAACATGGAAAACACGGTAATCTTGGAGAGCAATCCTGAAACATCT
GAAACTATTGATGCTGCTAACTGCCAACTTCATCAATTTGATTACTCCCACCTATGT
TCTAGCCTTTTTGATGATCATAGATTTAACTCCTTAGATATGAATTCATTTTGGTCTG
AT

>Peaxi162Scf00074g00355.1

ATGGGGAGAGCACCATGTTGTGTTAAAGAAGGTCTACGAAAAGGTCCATGGTCTG
CAAAAGAAGACTCATTACTTACTAATTATATCAACCAACATGGTGAAGGACAATGG
AGATCTTTGCCAAAGAATGCTGGTAATCGTTGGTCACTAATTGCTGGAAGATTACC
TGGTCGTACTGATAATGAAATCAAGAATTATTGGAATACACATTTACTCAAAAAACT
CAAAAGTGTTGGAATAGTTCCAAAACCCCACAAAATATTCTCCAAAAAAGCTAGT
AAAAAAGGTCAAAGTATACAAGTTGAGAAGACTAAAGTATATGTACCAAAGCCTAT
AAGGATTTCTTGTGGAATTTCAAGAAACAATAGTTTTGAAAATGTTACATTTAGTAC
TACTTGTTCCTCTAATAGTTATGAAGATGTTGATGTTGAAATTGAAAATAATAAAGA
TGGTATTATGAATAATGAAAATGAGGTCAACTTTGATATTTGTGATGAATTTCTTATG
GAAGATTGGTGCCACTTTTCAAATAAAGGTTCATTGCCAATGAATGAGAGTATGGT
AGATAAAGTCTATGAAGAATATCTTCTACTTCTTTCTGAAAATTGTTATCTTCAAGA
AGATCAAAGGGAGCATCCTTTAACTGGAAATGTCTCTGAGTTA

>Peaxi162Scf00074g01820.1

ATGGGACACCATTCATGCTGCAATCAACAAAAGGTTAAGAGAGGACTTTGGTCTC
CTGAAGAAGATGAAAAGCTCATCAGATATATTACAACTCATGGCTATGGCTGCTGG
AGTGAAGTCCCTGAGAAAGCTGGGCTTCAAAGATGTGGCAAGAGTTGTCGCTTGA
GATGGATTAATTATTTAAGACCTGATATAAGAAGAGGAAGATTTAGTCCAGAAGAG
GAGAAGTTGATTATCAGTCTCCATGGTGCTGTAGGCAACAGGTGGGCACATATAGC
AAGTCATTTACCTGGAAGAACAGATAATGAGATAAAGAATTATTGGAATTCATGGA
TAAAAAAGAAGCTAAAAAAGCCTTCAAAACCATCAACAAACACAACTTCTTGTAC
TGAACATCAGCAGCAACAAAGATCCCAATTTTCTTATAACACAACAAGTCAACCA
GAAATTTTATTTACACAAGATCTTGGAGTAACAAAGTCTCAGATTCTTCAAGATTCT
GCCCTTTTCACTTCTCCAAATCCTTTGTTCTATTTTGATAATGGTAATTCACTCGAAA
CAATGACAAATAATGTTAACGATCGAAGTACTAATGCCTCATTATTTCAAGAAACAT
CGGTGTTGAACTCAGAATTCTGGCAAGTTGACCTACAACAACAAGTACATACATCG
TATACAACGGGAATTCATTCGAATTATTTGCCACCATTATTAGAGATGCCACACATG
GAAATACCTAGTAGTAACAATAACATGATGGTGGAAGGACAAGATCATCATCAATT
AAATGAATGGAATATGGATACACAACAATGTTGTCCAAGTTATCTATTTTGGGATCA
AGAAACAGGAACACTTGGTGGTGATCATGAGTTTGTAGACCCAAACAATACATCA
AACAATATTGGACAGATTTTATCTTCTTTCCCTTCTTCTTTATGA

>Peaxi162Scf00078g00024.1

ATGTGTAGCAGGGGCCATTGGAGGCCTCACGAAGACCAGAAGCTCAGGGAATTAG

TGGAAAAATATGGACCTCATAATTGGAATGCTATAGCAGAAAAGCTTCAAGGAAGA

TCAGGGAAGAGTTGTAGGCTGAGATGGTTCAATCAGTTGGATCCAAGAATCAATC

GAAATCCTTTCACAGAAGAGGAGGAAGAAAGGCTTCTTTCTGCTCATCGAATTCAT

GGCAATAGATGGGCTATAATAGCTCGACTTTTCCCTGGTCGTACTGATAATGCTGTC

AAGAATCATTGGCATGTTATCATGGCACGAAAATGTAGAGAACGGTCCAAGATTTA

TGCGAAAAGAGCCACTGCTGCTAGTGCTACTACTATTGCTCATCAAAAATCAACCA

GCGATGAGCAAGAATTATCGTCATTGATGCAGCAAGATAAAAGGCAAATAAGTAAT

GAGCACACTACAAGAAGCTTCAATCCTTTCGTTGATGCTCATCAGCAATATTTTTTT

TCAGAAAGATTTGCTTACCCTTGTAATTTGACGTACAACTATTCCTTGTATCCAAAG

CTGCTTCACAAGGACTACCTTTTTCATCATGCGAAGGTGAATCAAGATGAAAAGCA

ACGGACGGAGTTCTATGACTTTCTACAAGTGAACACTGACTCAAACAAAAGTGAA

GTGATAGACCATATTAATGCTAGTGCTAGAATAATGAGAGATGATGAGGAAGTTGA

ACAGGAAGCTGTTGACCTTCATCGGAGCAAATCCAGCAGAGGATTCATAGATTTCT

TATCAGTTGGAGACTCAATCTAA

>Peaxi162Scf00080g00064.1

ATGGATAAAAAACCATGCAACTCTCAAGATGCTGAAGTGAGGAAAGGACCTTGGA

CTATGGAAGAAGATTTAATTCTCATAAACTACATTGCTAATCATGGTGAAGGTGTTT

GGAATTCCTTAGCTAAATCTGCTGGTCTCAAACGTACCGGGAAAAGTTGTCGGCTT

CGGTGGCTTAATTATCTCCGGCCTGATGTCCGGAGGGGCAATATTACACCTGAAGA

ACAACTTTTGATTATGGAACTGCATGCTAAGTGGGGAAACAGGTGGTCGAAAATT

GCGAAGCATTTGCCTGGAAGAACAGATAATGAAATAAAGAACTATTGGAGGACTA

GAATTCAGAAGCACATTAAGCAAGCAGAAACCATGAATGGACAAGCAGCTTCTTC

AGAGCAAAATGATCATCAAGAAGCTTGCACTAGCCAAATGTCTAATGGTCCAAATG

ACAATACCATTGATCAGACCTACTCTCCCACTTCATACTCTGGAAATGTGGACACTT

TCCAAGCAGGCCCTAATTTTCTCACTGAAGCAAATGACAACATGTGGAGCATGGA

AGACATCTGGTCCATGCAATTGCTTAATGGTGATTAA

>Peaxi162Scf00088g01621.1

ATGAAGGATAAAGAAGTTAAAGCAAGAATGAAGAGAGGATTTTGGAAACCTGAA

GAGGACTTGATATTGAAGAATTGTGTGGAGACTCATGGAGAGGGAAACTGGGCCA
CCATTTCTGAGAAGTCAGGCTTAATGAGGAGTGGCAAGAGCTGCAGACTAAGGTG
GAAAAATTACCTGAGGCCAAATATCAAGCGAGGAATGATGTCTGAAGATGAAAAG
GACCTCATCATCAGACTACATAAGCTTCTAGGCAACCGATGGTCGCTAATTGCAGG
TAGGCTTCCTGGAAGAACGGACAATGAAGTAAAGAATTTCTGGAACACACATTTG
AACAAGAGGTCTTGTAAAGGCAAAAAGAAACACAATAAGGCAGAGGAGGCAAAT
CATCAACGCAAAAACGATCGAGAATACCCTGCTGAGACAGCGAGGAACCAAGAA
GTGGCTACCAAAACAGTTTTAGATTCATGGATAGAAGAAATGCAGGACTTCAACTG
CAGCCTACTATCACCTCTGCCAATGAATAGCGTGGCATTTCTCCAAGATGAGCCTTT
CTTCCCCATATTGGACGACATTGTTTTGCTTGAAGCATTTACAAGCACTGGCAAAG
AAGTGTGGCCTGACATTCAGCCATTCCTTTAG

>Peaxi162Scf00089g01411.1

ATGGGGCGCCATGCTTGTTGTGTGAAGCAGAAGCTAAGGAAAGGATTGTGGTCAC
CAGAAGAAGATGAGAAATTGTGCAATTATATCACCAAATTTGGTGTTGGCTCCTGG
AGTTCAGTCCCTAAGCTAGCTGGGAGAACAGATAATGAGATAAAGAATTTCTGGA
ATTCGAGTTTGAAAAAGAAACTAATGAAGCAGGGGATTGATCCAAACACCCACAT
GCCACTAAGTGAAAATACTCAAGTAAGAGATGAAGAGAATTGTACAGATAAGACC
TCAATGTTGCAGATGCCACATCTTAATGGACTACCTTCAGCTGAAATGGACCAAAC
ATTTCACACGACTAATAGAAGTTTCAACACAGAGGCAAAAAATGGACATTTAACA
GAAGCTTCAAAGGAAGACCAATTTGTGAGTAAACAAGTTTTTGATATTATGTTTCT
TCATGGATACCAATCCAATGTGAATCCCAGCGCGTATGATTCAGAAGTATTAGCTCA
ATACCAGCAAATTATTAGATCTTATGATCATCCGAGCGAATTCGAAGAAAATCCAAA
TTATGGGATTTGTTCAGAGACAAGTTTAACAAAATTTGAACATGGACAGACGACA
GAAACTGATTTTGCTAGCAGCTCAAGTAACAGTTCAAATATTTGTGGCTATCAGAA
TAATGCTACTAGAATTCGGACTAATGGAATGCTGGAAATTAATGAAACCTTGTCATG
GGATGTGGAGAACAAAATGGAATCTATTTTCCAGTATCCTTACATTGAAATCAAGA
ATGAAGAATTAAAGCAAAGACCTGGAGATTTCAGTAATTGTCCATTGAGTTGCTTA
ACTGAAGAGTTATGTGGGGCAAATCTAGATGTCATCCAGCAAATTTAA

>Peaxi162Scf00090g00168.1

ATGGGAAGGTCTCCTTGCTGTGAGAAAGAGCACACCAATAAAGGGGCATGGACTA

AAGAAGAAGATGAACGCCTTACTAAGTATATCAAAGAACATGGTGAAGGATGCTG
GAGAACTCTCCCTAAAGCTGCTGGTTTGCTTAGATGTGGAAAGAGTTGCAGACTC
AGATGGATTAATTACTTGAGACCAGATCTTAAAAGAGGAAACTTTACTGAAGAGG
AAGATGAACTTATCATCAATCTCCACAGCTTACTTGGAAACAATTCTACTAATTTGA
ACAAGAAGATCACTAGCACAACTGCAGGTGCATCTACAACCACAAGTCAGTGTTT
TCAACAACTCCAAGCTCATAGTAGTGGGACGTTGTTGTGGACAAGATCGGGATTA
AGCGACAGCATTATAAACAACATTTTCATTAGTTCTATGGTCAAGAGTGATTCGGCA
GCAGAGGATTCTAACAGCAGCAGTGATTTATCAGAAGAAATGGTGCACCCACATCT
CAACCTTGAGCTCTCCATAAGCCTACCCCAACCCCTAGAAGGAAAGGAATTGAGA
AAAGAGGAGCAGCTATTTCACGAGCCATCGTTTACCAGTGCCAGTAGTGTTACTCA
ACCA

>Peaxi162Scf00096g01718.1

ATGGGTAGAGCTCCTTGCTGTGACAAAGCCAACGTCAAAAGAGGACCATGGTCAC
CTGAAGAAGATGCTAAGCTCAAAGCCTATATCGAACTACATGGCACTGGTGGCAAC
TGGATCACTCTTCCTCAGAAAGTAGGTCTCAAGAGATGCGGGAAGAGTTGTCGGC
TTAGATGGTTGAATTATCTCCGACCAAATATCAAGCACGGAGAATTCACTGAGGAA
GAAGACAACATAATCTGCAGTCTCTATATCAGTATTGGCAGCAGGTGGTCTATTATT
GCTGCTCAATTACCTGGACGAACAGATAATGATATCAAGAACTACTGGAATACAAG
GCTGAAGAAAAAACTACTAGGCAAACAGCGAAAGGATCATCGTCATCATAAACTA
GAAATGATGAAGGAGAACGAAAATTATTTTGCTAGTCAAGATATCAATGCATATTCT
TGGCCTCCACAACCACTACTATTCTCTTCACTAGTTGCACCAACAAATGAATATCAA
CAATCTGGAAGCAGTGGAAACAGGCACAACTTTCAGTACTCCACTGAGGTTTCAG
CCCAATTTGACCCGTCCAAATTCAGCCAAAGTTCAGCATTTGTGAATCCATTGAGT
GGTAATACTTGTAATTCATTAAGTTGCTATTACCCCATAAGCAATGGAGTTATCACA
AATAGTTTCCAAGAGTACAATAATTACATTTCTGTTGGGCTGGATCATGACGTGCTT
AACGTGAGTAATCTTCAACAAGTAGATCAAAGTGTAATTGAAATGGTCAACAGCA
GCAGCACTATTATTAGCACCACATCACCAGAAATTAGTACAAGCTGGGAAGAATTG
AGTCCTATTGTTTTTCCTCCATCAGTATCCAATTTCGAAATTCAGCAAGAAATATCA
CCTTATTTTGTATTTGAAGAGCCAAGGTACTTGGGTTTGTTGAAACAGTAA

>Peaxi162Scf00102g01226.1

ATGGGAAGAAAACCTTGTTGTGACAAAGTTGGAGTGAAGAAAGGACCATGGACA
ACTGAAGAAGACAAGAAACTCATCAGCTTTCTTGTCACAAATAATATTGGCCAATG
TTGCTGGCGTTCTGTCCCTAAACTTGCTGGCCTTCGTCGTTGTGGTAAGAGTTGTC
GTCTCCGTTGGACTAATTACCTTCGTCCTGACTTGAAAAGAGGCCTTCTTACTGAA
GCTGAAGAGAATTTAGTCATTGATCTCCATTCTTGTCTTGGTAACAGGTGGTCTAA
AATTGCTGCAAGATTACCAGGAAGAACTGATAATGAGATAAAAAATCACTGGAAC
ACTCATATCAAGAAAAAGCTTCTCAAGATGGGAATTGATCCTGTTACTCATGAACC
ACTGAATAAGGAAGAAAAGTCAAGAGATCAAAGTACCAAAACTGATGATATAGAT
AATAAACAGAATGGTCATGATGATCAGCAGGTCCATGTAGTACTAGAGAGTACAAA
TGTTACAGCAGCAGCCACCTCCTTAGAACTAGAAAACTCATCATTATGTTCATCATC
ATCTTTTTCCCCTAATGAAAACTCTTCTTGCATCACTGATGAATCCCAGATGGTTCT
TGATACCTTTAGTGAAAACAATCCACTATTAAGCTCCATGCTGCATGTCGATGCTCC
TCTCATCGACTTCCAATGGGAGCTTTCAGCTTCTGGTACTACTCAAAAGCAGAATC
TCGATAATGAAAATTTCAATTGGCTAGTGGATTGTCAAGATTTTGGAATCCATGACT
TTGACTTTGATTGCTTCAACATGGAAATGGAGTACTTCTTGGACAGTATTGGTGATA
TTAAGAATGAAAAGTAG

>Peaxi162Scf00102g01858.1

ATGGGGAGAGCACCATGTTGTGCTAAGGAGGGGTTGCGTAAAGGTCCTTGGTCTG
CTAAAGAAGACTTGTTGCTCACCAATTATATCAAGGAACATGGTGAAGGCCAATGG
AGGAGCTTGCCCAACAAAGCTGGGCTTCTTAGGTGTGGAAAGAGTTGTAGGCTAA
GATGGATGAACTATCTAAAGCCAGGGATCAAGAGAGGAAACTTGAGCCAAGATGA
AGAAGATCTTATATTAAGGCTGCATTCACTTTTGGGCAATCGTTGGTCACTCATAGC
TGGAAGATTACCAGGTCGAACTGACAATGAAATCAAGAATTATTGGAACACTCATC
TCATCAAGAAGCTCAAAAGTGCTGGAATTGAACCCAAAGTTAACAAGAGTTTCTC
CAAATACTGTTCCAAAAAGCAAGCCACAACAGACAAACCAAGAAAGAAACAAGT
GAAGAAGAAGAAGAACAAAAAGATAAAAGACCAGCCTTTAGTACAAGACACTAG
TGAGCCTCCTCAAGTTGTATTTATCCCAAAACCAATCCGAATTTCCTCTGGACATTC
GAGGAATTATAGTGTGGATCAAAATGTTGCATTGAGTACTTCATCCTCAAACAGTG
CTGATAACAACAACAATATTAGTAATAATGAGGGTAAACAAGCAGAGGTTTCATTC
GATCCACTCTTTGATGAAGTTGTTCTGGATGGATGTTGTGAACTTTCACCCGAATG

GTCATTACCAACGGATGATAGTATGCTGGAAAAGGTGTATGAAGAGTATCTTCAGC
TTATTTCTGAAGAATGTTTTCTTCAATTAGATGATCCTTTGGCTGAGAATGCCTCTC
ATCATCCAATGTTAATGTAG
>Peaxi162Scf00110g00004.1
ATGCAAGAACAGGGACAGCGCAGAGGGCAATGGCTTGAAGAGGAAGATGAGAGA
TTGGCTATGATTGTAGCCAGTTTGGGCGAACGGCTGAGGAGGAGTGGGAAAAGCT
GCAGGCTGCGATGGTTAAACTACCTCCGTCCCAATCTAAATCATGGTCATATTACTG
AAGATGAAGAACGTTTGATAATTCAACTTCAGAAACAGCTTGGAAATAAGTAA
>Peaxi162Scf00114g00127.1
ATGGGGAGAGCTCCTTGTTGTGATAAAGCTAATGTGAAGAGAGGGCCATGGTCAC
CTGAAGAAGATGCTAAACTCAAAGAATTCATAGAAAAATATGGAACTGGTGGAAA
TTGGATTGCTCTTCCTCAAAAAGCCGCTTATACACCAACATTGGAAGCAGGTAAAA
AAAATATATCATATATTGAAGTTAAACTCATACATTTATGTGACAGGTGGTCAATTAT
AGCTGCTCAATTACCAGGAAGAACTGACAATGATATCAAAAACTACTGGAATACAA
AGCTCAAGAAGAAGCTCATGGGTTTAATGCATTCATCAAACCAGAGAAAATCACC
TTTCTTTCCTTCTACAACTCTTCAAACTATTACACCAACGCAGCCCCAAACAATTTC
AAGTCTTTTTAGATATTCATATCAAGAGCCTAATATTTCATCAGTCCAACCAAATTTT
ATGTACAACAATAATCACATGAACTTTCAGTTAGGTACAAACAATCAATATTCTTAT
TTCCATTGTCAAGAGAGCTTAGTGAATCCCATTAAGCAAGAGGAAATGGGTAATTG
TTTAGAGGGTCAAACTCCGAGAACTTTCGATCATGAAACGCAAAAGTTCACCCTT
GATTATGGTAACAATATTGGAGGTGGTGGTGATCACAATCTTAGCGCTTGGGCTCAT
GATCAAAAGCCAAATGGATATCTTGCGAACAACTTCCAAACTCAATTTCAGTATGA
TGTTGAAGAAATTAAGGAGCTAATTAGTAGCTCTAGCAATGGAAGTGGATGTAATA
ATAACAGTAGTTTGTTATTCGTCAATGATGAAAACAAGCCAAATGAGAGAGGAATG
TTCTATTATTGA
>Peaxi162Scf00118g00310.1
ATGAGTACTTCTAATGCATCAACATCAGGAGTAAGGAAAGGTGCATGGACCGAGG
AAGAAGATCTTTTATTGAGAGAATGCATTGAGAAGTATGGAGAAGGGAAGTGGCA
TCTAGTTCCGGTTAGAGCTGGTCTGAATAGATGCAGGAAAAGTTGCAGACTTAGGT
GGTTGAATTATCTAAGGCCACATATAAAAAGAGGGGACTTCTCTTTGGATGAAGTA

GAGCTTATTTTGAGGCTTCATAAGCTTCTAGGCAACAGATGGTCACTTATAGCTGG
AAGACTTCCGGGAAGAACTGCAAACGATGTCAAAAACTATTGGAACACGCACCTT
CGAAAGAAGTTAATTGCTCCTCATGATCAGAAACAAGAGAGCAAGAGCTAG
>Peaxi162Scf00125g01321.1
ATGCTTTGTCAATTAATGAGTAGTGAGCAAATGGGCAGCTGGGGAGTCATAGAAG
AAGGGTGGAGAAAAGGCCCTTGGACTGCTGAAGAAGACAAATTGCTCATTGAATA
TGTCAAGTTGCATGGTGAAGGCAGGTGGAATTCTGTTGCTAGGCTTGCAGGGTTG
AAAAGAAATGGAAAAAGTTGTAGGCTGAGATGGGTAAATTACCTGAGGCCAGACT
TAAAGAGGGGTCAAATAACTCCACATGAAGAGAGGATCATTCTTGAGCTTCATGCT
AGATGGGGAAATAGATGGTCAACTATTGCTCGAAGCTTACCAGGGCGAACTGATAA
CGAGATCAAGAACTACTGGAGGACACATTTTAAGAAAAAGGCCAAGAACTCTAGC
GCTAACTCTGAAAAATCACGAGCACGTCTCTGGAAAAGACAACAATTCCAACAAC
TGCAACAGCAGCAGCAACAACAACAACAACAAATCAACGATCACATCGATATCAA
AAAGATGATGTCGTTATTCGATCAGAATGAGAATAAAGGGCAGCTTATGCCACAAG
GAAAGCAAGAAATGGCCATGTTGTACCCAAACACAACTAATCAACAAGAACAAGT
TGGCTTATTCTACTCTATGCTCAATGGATGTGCTTCAGTATCACTGCCTGAGCCCTC
TTCTAATGAAGACACCATGTGGGATGGCTTATGGAATTTGGATGACTTTTATGGCCA
TTTCATCAATACTAGTACAACTTATAACAAAGCTACTCCTTGCTTGCAAACTATGGC
TACTATTCCTCCAGCTTTCTATTGA
>Peaxi162Scf00128g00742.1
ATGGGAAGACAACCTTGTTGTGACAAGATTGGACTCAAAAGAGGTCCTTGGACAA
TTGAAGAAGATCATAAGCTTATGAATTTCATCCTCAACAATGGTATACAATGTTGGC
GTCTTGTACCAAAACTAGCAGGGCGCACGGACAATGAAATCAAGAACCATTGGAA
TACAAGAATCAAGAAGAAGCTAAAACTCCTTGGCTTAGATCCTTTAACTCACAAGC
CAATTGAACAATTTGATCATTTGAAAAATGATGATATTGAACAACAAGATTCTTCAG
AAAAAGAAGAGAAGGAGAAGTTGGAATTGCTAGAGAAGAATTTACCTAATTCTTT
GGATTATACACAAGAGCAAGAGATTCAAAATTCAACATCTAGTTCAAGTTTAGATG
ACATGAATAATTTCCAAAATTCAGAAAGTTTGGAGACATTTTCCAAAGAAATGTTT
AATTCAGGACTTGAAGATCCTTTCCAGAATTGGATA
>Peaxi162Scf00129g00228.1

ATGGGTAGACCACCTTGTTGTGATAAAATAGGTGTTAAGAAAGGACCATGGACTCC
TGAAGAAGATATTATGTTGGTGTCTTATGTTCAAGAACATGGTCCAGGCAATTGGA
GAGCTGTTCCTAATAGTACAGGGTTGAGAAGATGTAGCAAGAGTTGTAGGCTAAG
GTGGACTAATTACCTTAGACCAGGAATCAAGAGGGGTAGTTTCACTGATCAAGAA
GAGAAGATGATTATCCAGCTTCAAGCTCTTTTAGGCAACAAATGGGCTGCAATAGC
TTCATATCTTCCAGAGAGAACAGACAATGATATTAAAAACTATTGGAATACTCATTT
AAAGAAGAAGCTGAAGAAACTTGAAGCTGCAAGTTGTGGAAGTGATTTATACTCT
AAAGATAGTTGTTTATCATCTTTAAGCTCAACCTCAAAGGGCCAATGGGAAAGGAC
ACTTCAAGCTGATATAAACACAGCCAAATTAGCTTTACAAAATGCCCTATCACTTG
AAAAACCAAGCGCAATTCAAGAATATATGGCAACTGATGTGAAGCCCAAAAATATT
TGTTACCCATACATAAAACAAGAGGGTAACAATTCTACTTCTACTTATGCATCAAGT
GCTGAAAACATAGCCAAATTACTCAAACAATGGACCAAAAGTAGCTCATCAACAA
ACAATTCTGAGAATTCAAAGGGTTCGTCTAGTACGCAACTCTCGTGTAATTACAAC
AATGCCACTAACGACTTCGAGTCGATATCAAATTTCGAGTCATTTGAACAGTCAAA
ATCAGATCAATTTTCACAAGCTACAACACCTGAGGAGAGTAAATTTTATGGTGAAA
GCAAGAGAGAATTGGATGATCAAGTTCCATTGTCAGTAATGCTGGAGAATTGGCTA
TTTGATGAAAATGAAGATTTATTTTAG

>Peaxi162Scf00129g01231.1

ATGGATAAAAGAACATGCAATTCTCAAGATGTTGAAGTTAGGAAAGGTCCTTGGA
CTATGGAAGAAGATTTAATTCTTATAAACTATATAGCTAATCATGGTGAAGGTGTTTG
GAATTCCTTAGCTCGATCTGCTGGTTTAAAGCGTACCGGAAAAAGTTGTCGACTTC
GGTGGCTAAATTATCTCCGGCCAGATGTCCGGAGGGGAAATATTACACCTGAAGAA
CAGCTCTTGATTATGGAACTGCATGCTAAGTGGGGAAACAGGTGGTCAAAAATTG
CAAAGCATTTGCCAGGAAGAACTGATAATGAGATAAAGAATTATTGGAGGACTAG
GATACAGAAGCACATTAAGCAAGCAGATCAAAACATGAACAAATCATCAAAATGT
GAGCAACACAATGATCAACAAGCAATTAGTACAAGTCAAGCATCTACTGGTCCTAC
AGATACCATTGACTCCTATTCTCCACCTTCCTACACTGGAGATACTAATAATAATATG
GGAAATATTACATTTCAAGGCAATTTTCCAACTGAAACAAATGAAAATATTTGGAG
CATGGAAGATCTCTGGTCCTTGCAATTGCTTAATGATGCAACCAACTAA

>Peaxi162Scf00132g01412.1

ATGGGGTGCAAGCAAATGGACAAGCCAAAGCAAAAACACAAGAGGGGATTATGG
TCTCCAGATGAAGACCAGAAGCTCAAAGATTACATCCTCAGGCATGGTCATGGCTG
TTGGAGCTCTGTGCCCATCAATGCTGGCTTGCAAAGGAATGGAAAGAGTTGTAGA
CTAAGGTGGATTAATTACTTAAGGCCAGGCCTAAAGCGAGGGACCTTTAGCATTGA
AGAGGAGGAAACAGTCCTGACCCTTCATGGCGTGTTTGGAAACAAGACTGATAAT
GAGATAAAAAACCACTGGCACTCTTATCTAAAGAAGAGAGTGATCAAAAAGGCAG
AAAATGAAGGAGATGCTAGATCTGGATACTCAAGTCCACAAATAGGAAATGAAGA
ATCTTCACATTCTTCTATGAAATTGGCTTCCCAAAATTCAAGTTTGGACTCATTTGA
ACACATAGAATGTTCATTATTAGCAGACACAGATCAATCTTCTTCCCTACAGGTTGA
CTTTGCAAGAGAAAATTGCAAAAGCAATTTACCCAGAGTATTATTCTCTGAGTGGC
TTTCATTTGATCAACTTAATGGCCAAGATTTCAAGAACTCAAGCAATCAAGAATCC
AAGAACAATTTTGGCTACAACAATTCAGTGTTCCAAGACTCTTGCATGCATGGTCC
ACTCATGAATGACATAAATCAAGCGTTAAACAGAGGGATGGTAGACGATATGTTTC
AACCAACATTGGAATTTGAGGATCATATCTCTGCTGATGGTTTTGAGGAACTTATAT
CAGGGGAGTTCAATATAAATGGAGATATGATGTACATATGA

>Peaxi162Scf00134g00189.1

ATGGGGAGACAACCTTGTTGTGACAAAGCTGGAGTAAAGAAAGGACCATGGTCA
GCTGAGGAAGACAAGAAACTCATTAACTTCATTCTTAATAATGGCCAATGCTGCTG
GAGAGCTCTCCCTAGACTTGCAGGGCTAATGAGATGTGGAAAGAGCTGCAGATTG
AGATGGACAAATTATCTGCGACCAGACTTGAAGAGGGGACTTTTGTCAGGATATGA
AGAGACGATGGTCATTGAACTTCATGCACAGCTTGGCAACAGGTGGTCCAAGATT
GCTTCTCATCTACCAGGAAGAACTGATAATGAAATCAAGAATCACTGGAATACACA
TATCAAGAAGAAGCTGAAGAAAATGGGAATTGATCCAGTCACTCATAAGCCAATTA
CTAGTGATCAACCAAACATAGAACAGCCAACAAAAGACCAGCCAATTACTAGTGA
CCAACCAACCATAGAACAACCAACAAAAAATCAACAAGAGAAACAAAACATAAT
GCCACCTTCCACAGTTCATGTTGTCCAAGAAATGGATATTGATCAGAACAAGGAAT
TGGTTGAAACTCCTATACAATCAACAGTTACAGTAACAAAATTAGAAGAAGGGAC
AAGTCCAAAAAATATGGAGCCAATTCAAGTCAATAATGGATTTTGTACAGATGAAG
TCCCTTTAATTGAACCACATGAAATTTTAGTCCCTTCTGAATCAACCCCTTCAACAT
CATCTTCATCTTCTTCTTCTCCTCTTGAAGACATGCAATTCTTGCCAAGTTTTGATAA

TTGGCAATGTGATTTCAACAATATGGACAACATTGGTATTAATTGGGCACATGATTT
CAGCAGCACTTTGGATTATTTACTTAATGATGATGATAATGACATGAAAAATATTAGT
CTCCAAGATTGGTCACAGGTGTTGGAAGTTTGA
>Peaxi162Scf00135g00313.1
ATGAGGATTATGATTAAGGGAGGTGTTTGGAAAAACACCGAAGATGAGATATTAAA
AGCTGCTGTTATGAAATATGGGAAAAATCAGTGGGCCCGTATATCGTCGTTGCTGG
TTCGTAAATCTGCTAAACAATGTAAAGCTCGTTGGTATGAATGGCTCGATCCTTCTA
TTAAAAAGGCAAAAGATGAGAAACTACTTCACCTTGCAAAGCTCATGCCCACACA
ATGGAGGACAATTGCGCCAATTGTTGGTCGTACACCATCCCAATGCCTTGAGCGAT
ATGAGAAGCTTCTTGATGCAGCATGTGCTAAGGATGAGAACTATGACCCTAATGAT
GATCCGAGAAAATTGCGTCCTGGAGAGATTGATCCTAATCCAGAATCAAAGCCTGC
TCGTCCTGATCCTGTTGATATGGACGAGGATGAGAAAGAAATGCTTTCTGAAGCAC
GGGCTCGATTAGCCAACACAAGAGGCAAGAAGGCTAAAAGGAAAGCCAGAGAAA
AGCAGCTCGAAGAGGCTCGCAGGCTTGCTTCTTTACAGAAAAGGAGAGAACTCA
AGGCAGCTGGAATAGATGCTCGTCAAAGGAAGAGAAAAAGGAGAGGGATTGATT
ACAATGCCGAAATCCCTTTTGAGAAGAAGCCTCCTCCAGGTTTCTATGATGTTGCC
GAGGAAGACCGTCCAGTAGAACAGCCCAAGTTTCCAACTACCATTGAAGAACTTG
AAGGTGAAAGGAGAGTAGACAGGGAAGCTCGTCTAAGAAAACAGGATGTTGCAA
GGAATAAGATTGCACAAAGGCAGGACGCCCCTACAGCCATACTGCATGCAAACAA
ACTTAATGATCCAGAAGCAGTGAGGAGGAGGTCGAAACTCAATCTTCCTGCACCG
CAGATTCCAGACCATGAATTGGAGGCCATAGCGAAGATAGGTATTGCCAGTGATCT
ATTAGGGGGTGATGAGCTCTCCGAAGGGAATGCTGCAACGCGTGCTCTCCTAGCA
AATTATGCCCAGACACCACAGCATGCAATGACTCCTATGCGAACACCTCAGAGAAC
CCCTGCAACTAAGCAGGACGCTATTATGATGGAAGCGGAAAATCAGCGGAGGTTG
AATCAGTCTCAAACACCATTACTAGGAGGGGATAATCCTATGTTGCACCCATCAGAT
TTTTCCGGTGTCACTCCCAAGAAAAGGGAAGTGCAGACCCCAAACCCCCTCTTAA
CCCCTTCAGCAACTCCTGGAAGCTCGGGACTTACTCCTAGAATTGGCATGACACCA
TCAAGGGATTCTTATGGCATGACCCCCAAAGGAACTCCTATGAGGGATGAGCTACA
CATTAATGAAGAAATGGATATGCACAATAATGCTAAACTTGGGCAATTAAGTTCAA
AAAAGGAATTACTTTCTGGTTTGAAAAGCCTTCCTCAGCCCAAAAATGAGTACCA

GATAGTCGTCCAACCAGCTCCTGAAGAAAATGAAGAACCAGAAGAGAAGATTGA

AGAAGACATGTCTGATAGAATTGCCAGGGAGAAGGCTGAAGAAGAAGCAAAACG

ACAAGCTTTACTCCGGAAGAGGTCAAAAGTATTGCAAAGGGAGCTACCTAGACCT

CCAATTGGTTCACTAGAACTAATAAGAAGTTCCTTATTGAGAGCTGATGGAGAACA

GAGCTCCTTCGTTCCTCCTACACTAATTGAGCAGGCTGATGAAATGATTAGGAAGG

AACTTCTTTCTTTGCTAGAGCATGATAATAGCAAGTATCCTCTAGATGAAAAAGCA

GAAAAGGAGAAGAAAAAAGGCGTCAAGCGAAAATTGGCTGCTGAACCGTTGATT

GAGGATTTTGAAGAAGATGAATTAAAAGAGGCTGATGGATTGATCAAGGATGAGA

GTCAGTTTCTTTGTGTGGCAATGGGACATGAGAGTGAATCTCTTGATGAATTTGTC

GAAGCACACAAAACAACTCTGAATGATATCATGTACTTTCCTAGCCGGAATGCTTA

TGGTCTCTCGAGTGTTGCTGGAAACATGGAAAAGCTTGCTGCTTTACAGAAGGAG

TTTGAGAATGTGAAGAAGAAAATGGATGATGACACTAAGAAAGCCACAAAGCTTG

AGCAGAAGATCAAAGTTCTTACGAATGGATATCAGATGCGAGCTGGAAAACTTTG

GTCACAGATCGAGGGGACTTTTAAGCAAATGGATACTGCAGGGACAGAACTGGAA

TGCTTCCAAGCATTACAAAAACAAGAACAGTTAGCAGCATCACACAGGATCAACA

ATCTGTGGGACGAAGTTCAGAAGCAAAAGGAGCTTGAGCGCACCTTACAGAAAA

GATATGGTGACCTCATATCAGAGAAGGAAAAGATCCAGCATATCATGGATGAGTAC

AGAATACAAGCTCAAATGCAGGAAGAAAATGCAGCAAAGAATCGTGCTCTTGAGC

TGGCCAAGGCAGAAGCTGAGGCAGTTAAGAATCAACAAGCTCCTCATCCAGTTGC

AGAGGCCCCTAACGATCAGGGGGATATGTCAGTTGACCCTGCTCCAGAGGGATCT

CTTGACGGTATGGCAGAAAAGCAGAGTTCACCTCCTGCTGCTGTTGAAGCATCTCT

GACCGATGTAGTGCAAGTGAAACTGACCGATGAAGTGCAAGTGAAACTGACCGAT

GAAGTGCAAGTGAAACCCTTAGCGACTGGGTCAGAAGCTGTCGACTCTAGTGTCA

GTTCCCAGTGTTCCAATGCAGAGGAAAATTCTGCTTCTGCCTCTCAATCTACTGTG

ATTGAAGTTTCTCCGACTGATGAAGTGCATGAGAAGCCCGTAGATACCAGCTCAGA

AGCCGTCAACACTATTGTTAGTTCCCAGTGCTCCAATCCAGAGGATAATTCTGCTTC

TGCATCTCAATCAGTTGCTAGTGAAACTCCCCCGACCTATGACCCAATTCCATCTTA

G

>Peaxi162Scf00137g01812.1

ATGGGAAGAACACCTTGCTGTGAAAAAAATGGACTTAAGAAAGGTCCATGGACAC

AAGAAGAAGATCAAAAGCTCATTGATTACATTCAAAAACATGGTTATGGAAATTGG
AGAGTACTTCCAAAAAGTGCTGGACTTCAAAGGTGTGGAAAGAGTTGCAGGCTTC
GCTGGACAAACTATTTAAGGCCAGATATCAAAAGAGGACGATTCTCTTTTGAAGAA
GAAGAGACAATCATTCAACTACATAGTATTTAGGGAACAAGTGGTCCGCAATTGC
TGCTCGCTTGCCTGGAAGGACTGATAATGAAATCAAGAACTACTGGAATACTCACA
TTAGAAAAAAGCTCTTGAGAATGGGAATTGATCCTGTGACACACACTCCTCGTCTT
GATCTTCTTGAATTTTCCTCCATCTTAAACCCTTCACTCTATAATTCATCTCAACTGA
AAGTTTCAAGGTTGTTAGGTGGACAACCTTTGGTAAATCCTGAAGTCCTCAGAATA
TTAGCGAATTCTCTTTTATCATCGCAGTACACGGTGAAACCATCTGAACTTGTCGAT
CAGAATCTATTAATGTCACGTAATCTTCAAGGAAATCAGTTGTCTAATCCCCAAGCC
CAAAACCAATTGCCACCATTAGTTCAAGATATTTCATGTGGTCCATGTTCTAATGAA
GCACAATTGATGCAACAAGCCAATCTGGAACAATTTTCATCAAATATGTCAAACTA
TAGTTCACAAAATTGCCAACTAAATGACTGGCAAAACCAAGAAATGCCATCTCATT
TGACAGAAGATTATAACTATCATCTACATAATTATGGTTACTATCAGCAGGATCAGTC
CATCATGGACCCTCCACCGTCCGATGCCTCAGCTTTTCAATCCAACGACAGCAACG
TGAGCTACCAATCTATTTTATCAACGCCTTCATCAAGTCCTACACCATTGAATTCAA
ACTCGTCATACATCAACAGCACAACTACTGAAGATGAAAGGGAAAGCTACTGCAG
CAACACGTTCAATTTTGATATTCAAAATATGTTGGATGCAAATGCACTCTTGTAA

>Peaxi162Scf00147g00136.1

ATGGGAAGAGCACCTTGTTGTGAGAAAGTAGGTCTCAAGAGAGGCAGGTGGACT
GCTGAAGAAGATGAAATCCTCACCAAATATATTCACGCTAATGGTGAAGGTTCTTG
GAGGTCATTACCCAAAAATGCAGGCTTACTGAGATGTGGAAAGAGTTGCCGACTG
AGATGGATTAATTACTTGAAGACTGATCTGAAAAGAGGAAACATAACTCCTGAAG
AAGAAGCTATAATCATCAAGTTGCGTGCAACTCTGGGCAACAGGTGGTCTTTAATA
GCAGAACATTTACCAGGTAGAACAGACAATGAGATTAAAAACTACTGGAACTCTC
ACTTGAGTAGAAAAGTAGAAAGCTTAAGGATTCCAAGTGATGAAAGATTGCCAAA
AGCAGTGGTGGAATTAGCTAAAAAAGGAACACAACAGCTAAACAAGCAAAGACG
CGGGCGAGTAAGTCGATCAGCTATGCAGAAGAACAGAAATAACCTTTCATCAACA
AAGGGTACAAGTACTGCTTCAACTTTATCAAAGCCTCAGCAGCCACCAAAGGAAA
TTAATACAATTTCTATGCCCTCTACACCAAATATAATAGAGAAAGAGGTCTTATCTA

GCACCACTACTAGCTCAGGGCAAGATGCGAGTAATACTCATTTAACAAGTGACAAC
TATGCCAGTGAACTAGATAATGAGATGCTGTGGCATGAAGAGCTAGATCCAGATTT
TATTTTCACTTGTTTATGGAACGAGGAAGGAGAAAATCTTGAAACAGTTGAGAATA
ATAATAGTCATAATAGTAATACCGAAATATTAAGTGTAAATGTGGACATGGTTAGTCC
AGGGAACTCGAAAATTAATGAAACAAGTACACTAATGGAAGATGGCACTTCGCTT
GATTGGGATTGGCAATATTTATCAGAATGGAATGAAATATGGGCAGAGCAAGAAGA
GAACATGTCAAATAATAATATTTCATCCAGACAGCTGTCTAATGAAGCATTGGTAGA
AATTGATCCAGTGCAGCAGGTTGATTATAATCATAGTGAACTAGTAGCTTGGCTTCT
GTCTTAG

>Peaxi162Scf00160g00722.1

ATGGGAAGAGCACCTTGTTGTGACAAAAACAGTGTGAAGAAAGGGCCATGGTCA
CCTGAAGAAGATGCAAAGTTGAAGGCATATATTGAGCAACATGGTACTTGTGGAA
ACTGGATTGCATTGCCTCAAAAAATTGGCCTTAAAAGATGTGGAAAGAGCTGTAG
ACTTAGATGGTTAAACTATTTGCGACCAAATATCAAGCATGGTGGATTTTCAGAAG
AAGAAGATAACATCATTTTAAGTCTCTATATTAGTATTGGAAGCAGGACTGATAATG
ATATCAAGAACTATTGGAACACAAAGCTAAAGAAGAAGTTATTTGGAAAGCAGCG
CAAAAATCTTAAAGGTAACAACCAAAAACAAGGATCAAGAAAAGGAAGAGAAAT
GAGCAGCTCCATGTCCATGGTTTCTAATGAAAATATTATTGCAAACCCTAGTTGGCC
TGAGCTTCCTATATTGCAACCAATACCATATTCAAATGAGGAACCACGTTTTAACGA
CCACTCTTCGATTAGAAAGTTGTTGATGAAACTTGGAGGGAAATTCTCAGATGATG
ATAATGATGATGAAAAACCAATGAATGAACCACCAAGTAATCCTCAATATCCTATGG
ATAATTCATTGATTCAGCCAATTTATGATCAAGATTGTATAAACATGATGCATTCTCC
TTTCACAAATAATACTCATTACAACATGGACGCGAAAGCACTCTGCTGGGCTGATA
CTGACACTGAGAGACGAAAGCTAGGGGAACGAATGGGATCAGATACCCCAGTAGT
CCCAACCGTAAACGATGGATGCAATTTTATAAATGAGCTTGAGCATATGATGTACAC
TAATCCACAAAAATTAAGTGGTCTTGAAATGTTATATGTGGATATGCTTAATAATAAG
CATGCCACTACTTTGGGAGGAAGCTTGGACTTGGAAGATATGAACAATTTGGTGTT
TCCACTTCCTCCTCTAGATGCTTCAAACACTGAAGGTCATCAACATGGAACTTTGC
TACAAGGAGGTGCACTTAATGAGCTAAGACTCCAAGCCATGTCCCACCCCCCTTGT
GTCTGGCTTCAGCAGCCTGGTAAGGGTTGGCTGTCTGAGGCCATAATGATCCGTGA

GTCTCGTGTGGAAGGGCTTTCGCTCAATGGATCAAAG

>Peaxi162Scf00164g00073.1

ATGGATCATCATCACAAAACTAAAGTTGGTTTAGGCAGAAGAGGAGAAGATTATCG

TACGAGCAACGTCATGAATCAAGAAAACATTAGGGAGGAAGATATTATGGACCTTA

GAAGGGGTCCATGGACTGTTGAAGAAGACTTCACTCTTATCAATTTCATTGCTCAT

CATGGTGAAGGTCGTTGGAATTCCCTCGCTCGTTGTGCTGGGAAAAGCTGCAGATT

AAGATGGCTTAATTATCTTCGTCCTGATGTTCGACGTGGGAATATTACTCTTGAAGA

ACAACTCTTGATTCTTGAACTACATTCTCGTTGGGGCAACCGATGGTCGAAAATTG

CTCAGCATCTACCTGGAAGAACTGATAACGAAATCAAAAACTATTGGAGAACACG

AGTGCAAAAGCATGCCAAGCAACTGAAATGTGACGTGAACAGCAAGCAATTTAAG

GATACCATGAAGTATCTTTGGATGCCAAGGTTAGTTGAAAGGATTCAGGCAGCCGC

CACGACGGCTGCTACTTCCACCACCACCACCAACACCTACATCCAGAACCTAGAA

AATCAACAATCAGTACCAAACATGAGTCATATGTCCCAATTTCTTCCTATTCAACTG

GAGAATCACAAGAAAACAAACAGTATTAATCATTCTAGCATGACTCTCACGCTTGA

GAATTCGAGCACAACTACTTCATCAGACAACTCAATTGGGCGACAAGTTTCACCA

ACATCTGACCTTACTGATTGTTACTACAATTTTTCAATTAACCAAAGCAGTAATAAT

CAAGACTATACTCCATTTAATCAAAATTATGGAGAATCCTTGATTAGCGCAACAGGT

TATTTTCCTCAAGCAGTGGATCAGCAAAATTCTCAATGGATGGATAGTGAGTATGTT

TCAGACAATTCATGGAACATTGAAGACATGTGGTTCTTACAGCAGCAATTGAATAA

TAACCTATGA

>Peaxi162Scf00164g00216.1

ATGGCTGAACAAATGGGGAACTGGGGAGTTATAGAAGATGAATGGAGAAAGGGCC

CTTGGACAGCTGAAGAAGATAGATTGCTCATTGAATATGTCAATTTGCATGGTGAA

GGCAGATGGAATTCTGTTGCTAGGCTTGCAGGATTGAAAAGAAATGGAAAAAGTT

GTAGGTTGCGATGGGTGAATTATTTAAGGCCTGACCTTAAGAGGGGACAAATAACT

CCATATGAAGAGAGGATCATTCTTGAACTTCATGCTATATGGGGTAACAGATGGTCA

ACAATTGCAAGGAGCTTGCCAGGAAGAACTGATAATGAGATCAAGAACTATTGGA

GGACACATTTCAAGAAAAAGGTCAAGAAATCAATTGACAATTCTGAAAAAACAAA

AGCAAGGCTTTTGAAAAAACAACAATTTCAACAGCACCAGCAAGAACAATTGAA

GAATCAAATATATATGAAAAAAGTCATGTCATTATTAGAAGGAAACTATGAAAATGA

AGTTCCTATCTTGCCTCAAAGGAAGCAAGAAATGGACTTTATGTACCAAAATACAA
CTGAGCAAGAGCAAGGTGGTGGATTCTTTTACTCTATGCTTAATGGATATGCTACTG
TGCCAGTGCCTGAGGTTTCATCTAACGAAGACATGATCATGTGGGATCATGGCTTAT
GGAACTTGGAAGATGTTAATGCCAATTTCAATACTGCAGCTTACAATAAAACTCTA
CCTTGCCTGCAACCTCTGGCTACTCCTTTCTATTGA
>Peaxi162Scf00165g00111.1
ATGAGGGAGAGGCAGCGTTGGCGATCTGAAGAGGATGCTTTGTTGCGTGCTTATGT
GAGACAGTATGGACCAAAAGAATGGCACCTTGTATCACAGCGTATGAACACGCCC
CTCAACAGAGACGCTAAGTCTTGTTTAGAAAGGTGGAAAAACTACCTCAAACCAG
GCATTAAGAAAGGATCACTCACTGAAGACGAGCAGCGTCTTGTTATCCAACTACA
GGCTAAACACGGGAACAAATGGAAGAAAATAGCAGCTGAAGTACCAGGTCGAAC
TGCTAAAAGATTGGGGAAGTGGTGGGAAGTATTCAAAGAGAAGCAACAGAGGGA
GCATAAAGAAAACAATAAAGTTGTTGATCCGGTAGACGAGGGAAAATACGACCAC
ATTCTGGAGACCTTCGCAGAAAAGATTGTGAAAGAACGTAGTGTTCCAGGATTAC
TTATGGCTACTTCTAATGGAGGTTACCTCCATGCTGATGCACCGGCTCCTTCACCAC
AAGCTCTTCTTCCTCCATGGCTTTCCAATTCCACTGCCGCTTCAACTGTCAGATCAC
CGTCTCCATCTGTGACCTTGAGTCTCTCCCCCTCAACGGTGCCCCCTACGCCTACT
CCTGGCATTCCATGGTTACAAACCGATAGAGGACCTGAAAATGCACCCATTGTCTT
GAGCAGTTTTCCTCATCATGGAATTGCGCCTCCTTGTGGAGAAAATCCATTTGTTAC
TGAACTTGTGGAGTGCTGTAAAGAACTGGACGAAGGGCATCGTGCTTGGGCTGCA
CATAAAAAGGAAGCAGCTTGGAGATTAAGGAGAGTAGAATTGCAGCTGGAATCAG
AGAAAGCGTGCAAAGTTAGGGAGAAGATGGAGGAAATTGAAGCAAAAATGAAAG
CTCTGAGAGAAGAGCAGAAGGCAAGTCTAGACAGGATTGAAGCAGAATACAAGG
AACAACTAGCAGGCCTGCGGAGGGATGCAGAAGCAAAGGAGCAGAAATTGGCGG
AGCAATGGGCTTCCAAACACTTGCGTCTTGCTAAGTTTCTTGAGCAGATGGGTTGC
CAATCAAGACTAGCAGAACCTAATGGTGGGCGCTAA
>Peaxi162Scf00165g00128.1
ATGGATGAATTGAAAGTAGAAGACTGCTGTACTGAGAATAAGCAATCAGCTGTTGC
TTCGAGCTCTTCGGTTTCTGAAAATAGTGGTAGTGTTACTTTAAGGTCGCCCGCAG
TTTCTAGCCCAACTCCTACGTCACCAACTCACAGGAGAACCACTGGTCCAATTAGA

CGAGCAAAGGGTGGTTGGACTCCTGAGGAGATAAAATCTCCCTTTCCACAGGATG
ATACTTTGAGGAGGGCTGTTGCAGCTTACAGAGGGAAATGTTGGAAGAAAATAGC
TGAGTTCTTCCCGGATAGATCAGAAGTGCAATGCCTGCATCGATGGCAGAAGGTTC
TCAATCCAGAACTTGTTAAAGGACCCTGGAATCGGGAGGAGGATGAAAAGATTAT
TGAACTGGTCGCTAAGTATGGGCCTACGAAATGGTCTGTCATAGCTAGATCGTTGC
CTGGTCGAATAGGAAAGCAATGTCGTGAGAGGTGGCACAATCATTTGGATCCCACT
ATAAAAAAAGATGCTTGGACACTGGAGGAGGAACGGGCACTCATGGATGCTCATC
GGATTCATGGTAACAAGTGGGCTGAAATTGCTAAGGTCCTGCCTGGAAGGACTGA
TAATGCAATCAAGAATCATTGGAATAGCTCTTTGAGGAAGAAGTTAGACTTTTATTT
AGCTACCGGTAACCTTCCGCCTGCTACTGGGGAGGGCCTTCAAAATGGTTGCAGG
AACACTGATGGAACAGCTAAAGCTGAAGAGTTTCTGCTTGGCTCAAATAAAAGAT
CAGAAGCGGCTGTTTCATCAGGAATTACAGATACGTGTAAAATTGAAAATGGTGGG
AAGAATCACTATAATGCGAGATTGCAGGTTGCAGAAATCAGTGCTTCAACTGGTGG
TCCTCAAACTGAGTCCACTGACTCGGAAGTCGCCAGGTGGGATGCTCAGTCTCCA
GAAATAGATGCTATTCAACATATAAAACCAAGTACAGAATCAGAAAAGAGGTATGA
TGGGCGGAGAATAAATAGTGCAGTAGATCAACTAAATGTAATTGAAGCACCATTTC
CTTGTGAAATCCCGACATATGGTACGTTATACTATGAACCACCAAGTTTGGAAAGT
CGTAGTCCACTGGATTCAAATCTTTTAAATATCTGCTGGGATCAATGTGAATCTGAC
GCAAGCCCAAGCCCGTCATCAAACAGTTTCTTCACTCCGCCTAGTACAAAGGGCA
GTAGCCTTTATGCACAAACTCCTGAATCTGTACTGAAATATGCTGCTAGAAGCTTTC
CAAATACGCCTTCCATATTAAGAAAGAGAAAGACTCAAGGTAACTTTTCCACGCTA
ATTAATAAAATGGGGAAGACAGGTGGAGATATCTGCAAGGACAAACCTACTGACG
CATGTGAGGTGAACAGAATTGACAGCTCCGAAAAATCTGGCATGCATAATGAAGG
TCTTCCAAGAAATTTTTCCAATGGTATTGGGCTATTCAATAGTCAACCATTTAATGC
CTCTCCTCCCTATCGATTAAGGTCTAAACGAACATCCATCTTCAAGTCTGTGGAGA
AGCAGCTTGAGTTTGCGTTCAACAACGAGAAGAAGCATGATACAAGTAATGGATT
CAGTGATTCAACTGATAACGAGATATCACATGCAGTAGAGAAGGCTACACATTCAC
CAGAGAGTGCAAGTTAA
>Peaxi162Scf00166g01118.1
ATGGGTCGATCTCCATGTTGTGATAAAGTTGGTTTGAAGAAAGGACCTTGGACACC

TGAAGAAGATCAAAAACTCTTGGCTTATATTGAAGAACATGGTCATGGTAGTTGGC
GTGCATTACCTGCAAAAGCCGGTCTTCAAAGATGTGGGAAGAGTTGCAGGCTTAG
ATGGACTAATTACTTGAGGCCTGATATTAAGAGAGGAAAATTCACTTTACAAGAAG
AACAAACCATTATTCAACTCCATGCTCTCTTAGGGAATAGGTGGTCGGCTATTGCA
ACTCACTTGCCAAAAAGAACAGACAATGAGATAAAGAATTATTGGAATACACATCT
TAAGAAACGGCTAGTAAAAATGGGCATTGATCCAGTGACTCACAAGCCCAAGAAT
GATGCCCTCTTGTCCCATGATGGTCAATCCAAGAATGCAGCTAACCTTAGCCACAT
GGCTCAGTGGGAGAGTGCTCGGCTCGAAGCCGAAGCTCGACTAGTTAGACAATCC
AAACTTCGGTCCAATAGTTTCCAAAATCCTCTTGCTTCTCAAGAATTATTCACATGT
CCTACCCCTTCTAGTCCTCTCCACAAGCCAATTGTCACACCTACAAAGGCCCCTGG
ATCCCCTCGATGTTTGGACGTACTAAAAGCTTGGAACGGTGTTTGGACCAAACCA
ATGAATGATATTCTTCGTGCCGATGGTAGCACTAGTGCTACTGTTTCTGTCAATGCA
CTCGGCTTGGACCTGGAATCTCCTACTTCTACACTAAGCTACTTTGAAAATGCGCA
ACAAATTTCTAATGGGATGATTCAAGAAAACTCTACTTCTTTATTCGAATTCGTTGG
AAATTCCTCAGGGTCAAGTGAAGGTGGAATTATGAATGAAGAAAGTGAAGAAGAT
TGGAAAGGATTTGGAAATTCATCAACAGGACATTTGCCTGAATACAAAGATGGGAT
AAATGAAAATTCAATGTCACTCAATTCAACACTTAAAGATTTAACTATGCCAATGG
ACACTACATGGACAGCAGAGTCACTAAGATCAAATGCAGAGGACATTTCCCATGG
TAATAATTTTGTGGAGACATTTACCGATCTTTTGCTTAGCACTTCCGGTGACGGCGA
CGGCGGCTTGTCGGGAAATGGCACGGATTCCGATAACGGTGGCGGTAGCGGAAAT
GATCCTAGTGAGACTTGTGGAGATAACAAGAATTACTGGAATAGTATTTTTAACTTA
GTGAATTCTTCACCCTCAGATTCAGCTATGTTCTAA

>Peaxi162Scf00170g01335.1

ATGGGGAGATATCCGTGTTGCAAATTAGACAATGATTTGAAGAAGGGACCATGGAC
TGCTGAAGAAGATGAAAAACTAAAGGAGTATATTCAAGGACATGGCCATGGAAAT
TGGCAATTAGTCCCCAAAAGAGCAGGCTTAAACAGGTGTGGGAAGAGCTGTAGAT
TAAGGTGGACAAATTATTTAAGGCCTGATATTATTAGAGGAGGTTTCTCAGATGACG
AAGAACAAATGATCATCAACCTTCATTCTGTTCTTGGAAACAAATGGTCAAGAATT
GCAGCTCATCTTCCAGGAAGAACTGATAATGAAATCAAGAATTTATGGAATACTCA
TCTCAAGAAAAAGCTTCTGAAGGCTGGAATTGATCCAGTAACACATCAACCAATA

AATGATCCAAGACTCCTCCTTAGTCTTTCTAACTTGATGAACCCTTTGGAATCTGTT
CTTAGGTTACAAGCAGAAGTTACTGAAATGGCCAAAATCCATCTTATTCAAAACAT
AATTCAAGTCCTAACTCCTCCATTAATTCCTAATTTACAAGAAAATAACTTCCCCAT
GCAACAATTATATAATCTTGCCCTATATGAAAATATTGTTACTAATGTTACACATATAG
ACGAACCTTCTCCAATTTCGAATTCATCGGACATGCTCTTAAGTCCAAGTGTAGTG
GAGAATAATATTACTGGATTTAGTAATGTGTCTAATGTTAATTCAGAACATTCACTTC
CATCACTAGTTGAAGCTACACCTGAGAATTCACCCTCTGAGCAGAACGATATAATT
CCCAATTATTTTGGTAGTTTCTCAGAAACTAAGGACTTTGATGCATGGGAAAAAAG
TTTGGATGATGAAGCTAGCAACTCCTTCTGGCAAGATATTCTTTGA
>Peaxi162Scf00170g01336.1
ATGGGGAGGTATCCATGCTGCAAGGATGATGAAGATTTAAAGAAGGGACCATGGG
CACCAGATGAAGATGAGAAATTGATGGATTATATTAACAAACATGGCCATACAAAC
TGGCAATTGCTTCCCAAAAAAGCAGGTTTAAACAGATGTGGAAAGAGTTGCAGAC
TAAGGTGGAGTAATTATCTCAAGCCTGATATCAAAAGAGGAAAATTCTCCATTGAA
GAAGAAGAAATGATCATCAACCTACATTCTCTTCTTGGAAACAAGTGGTCAAGAAT
TGCAGCTCATCTTCCTGGAAGGACAGATAATGAGATAAAGAATTTCTATAATACTCA
TTTAAGGAAAAAACTGCTCAGATTGGGAATTGACCCAAGGACTCACAAACCAATT
TCTGACCTCAATTACCTCATAAATCTTTCTCAGAGTTTTACTTCTAACAATAATAATC
ACCTGATGAATAATCCCTTAGCATCTGTTCTTAGGTTACAAGCAGAAGTCACAGAG
ATGGCCAAAATTCAACAATACTTACAAGGCCTTTTTCTTCCACCCATAAATACAAAT
ATTCCATTTTATTCATCCAATATACAAGAAAATTTTGCAAGATATTCAAGTCATTTAT
TTCCTCCACTTGAGGGTCATGTTACAAATGTTACTAACACCCAATTCTTGGATTCAT
GGCTAGTAGACTGTGCTAAAAGTCCAAACTTGGATAATTCTACTTCTTGCATTTCAA
ATTCATCACAATCCTATCATCAAGGTGAATTGAACTCAAAGGTAGATAATTTTGAAG
GACTAAACAGCAGCTCAAAGACATATAATGATTATAGTAACTCACTTCCAGTACTTG
CTCCATCTTCTCCAGAGGCTGCTCAT
>Peaxi162Scf00171g00047.1
ATGTGTAGCAGAGGACATTGGAGGCCTCATGAAGATGAGAAACTCCGAGAGTTAG
TTGCTAAATATGGACCTCATAATTGGAATGCTATTGCTGAAAACTTACAAGGCAGAT
CAGGGAAGAGTTGTAGGTTGAGATGGTATAATCAATTGGATCCAAGAATAAATAGA

AGTCCTTTCACTGAAGAGGAAGAAGAAAGACTTCTTGCTTCTCACAGAATTCATG
GCAATCGATGGGCTATAATTGCTAGGCTTTTTCCTGGCCGAACTGATAATGCAGTGA
AAAATCATTGGCATGTTATTATGTCCAGAAGATGCAGAGAAAGATCTAAAATATATT
CCATGAGGAATATTAATAATTCTGCCCAAAATTCAACTACAAGCCCGCAAGATACTT
CCCAAAATACCAGAAGGGGAAGTCCCAACATGAACTCTATTGATCAGCAATTATTG
GGAAGATTTAATTACTACCCTAACCTCACTTTTAATAATTCCCTTTATCCAAAGGAG
CTTTATTTTGACCACCTTCGTCATCATTTGAAGATAAATGAAGACAAGAATCAGGA
AGTGGAATGCTATGACTTTCTACGAGTGAATACAGGCTCAAACAAAAGTGAAGTC
ATAGATCATATTGCAAGAAAGGGTACTGATGATGAAGAAGTGGAACAGGAGGCTG
GTTATCATTATCCTGCACAAAGCAAAGCTCCAGTGCAATTTATAGATTTCCTATCAG
TTGGAGACTCATCGTAA

>Peaxi162Scf00177g00512.1

ATGGGTAGAAGGCCTTGTTGTTCTAAGGAAGGCTTAAACAAAGGAGCATGGACTC
CTATGGAAGATAAGATTCTAATAGACTACATTAAAGTTCATGGTGAAGGGAAATGG
AGAAACCTTCCTAAAAAGGCTGGTCTTAAAAGATGTGGAAAGAGTTGTAGACTAA
GATGGCTGAATTATCTAAGGCCAGACATTAAGAGAGGAAATATAACTCGAGATGAA
GAAGATCTCATTATCAGACTTCATAAGCTTCTTGGAAACAGATGGTCTCTGATAGCT
GGAAGGCTACCAGGACGAACAGACAATGAAATCAAGAACTATTGGAATACAAACA
TTGGCAAAAAATTACAACACCAAAGAGCTGCAGCTAATTCTGGACATGCCAAGTC
CAGACCACCAGTTACTACCCAAGACATTGTAGGATCAGGATCTTCATTATCTTCCTC
GGCCTCCCCATGTTTAGTTGTTCGGACAAAGGCAATGAGGTGCACTAAAGTTTTCA
TTCCTACGCCCAAAAATACTAGTCATGACAACTCAATTACTACTAATTGTAATAATG
ATGATGATGACAAGGTAATGGCAGTAGAAACTACTGCTTTAGTAGCATCCTCATCCT
CATCATTTACCCTATCTTCAATATTATCCGAGCAACAACCAATATCAGGATCATCACC
AGTGTCTTTGTCTGGAGATCAATTCATGGAAAATAGCTTTAACTTCATGTTTAATTT
TGACATGGACGATCCCTTTCTATCTGAGCTTCTTAATGCAGCTGAAAATACAACTAC
TATTGGTGGTGACCAAGTTGGAGATAGTTTCAACAGAAATGAAAAGGAAAGAAGT
TATTTTCCTCCAAGTTCTAGTCAAAGTGCATTGTTCTCAGAAGAGACGCAACACAA
CGATTTGGAACTTTGGATTAATGGGTTCTCTTCTTGA

>Peaxi162Scf00191g01323.1

ATGGGGAGAACACCGTGTTGTGATAAGAAAGGTTTAAAGAAAGGACCATGGACAC
CTGAGGAAGATGAAAAACTCGTTGAATATATCAAGAAGCATGGTCATACTAGCTGG
CGTTCTCTTCCTAACCTTGCAGGTCTTGATCGTTGCGGGAAGAGTTGCCGTCTTCG
GTGGACAAATTATCTAAGACCAGACATCAAACGAGGTCCATTTAGCCAAGAGGAA
GAGAAGCTTGTCATACAGTTACATGGAATTCTTGGCAACAGGTGGGCAGCAATCG
CATCACAACTGCCTGGAAGAACAGACAATGAGATAAAGAACTTATGGAATACTCA
CCTGAAGAAACGCCTCCTTTCCATGGGAATTGATCCTCAGACTCATCAACCCTCTA
CAGCACCCAATGGACTACTAAGAAGACCACCTACATCGTCTTCAGCCCGTCACATG
GCACAATGGGAAAAAGCAAGGCTCGAAGCTGAGGCTCGTCTTTCCAGGGAACCA
CAACTCTTGGTTCCATCATTAGTAGGAAAGTCTGAAACTGACTACTTTTTGCGCATA
TGGAATTCAGGGATAGGAGAAGCATTTAGGAAATTGAAGAAAGAGGAAAAGACT
ACTTGTCAAAGTCCAGCCTCTCAAGAATCATCATCCACAAAATATGGATCTGATTC
GGGCATCACAACTGAGATAGACCTTGGTTTAGCTGGTTCTCCAGCTGCAGGAAGC
AATCAAAATGAAGATACAAAGTGGAAGAATGCTCAACCATATACTGAAAATTTCTT
GCAAGGATCTGATACCTCTAGCTCCAGTGGATTGGAAGATTCTTCTGAATCGGCAC
TGCAACTTCTTTTGGATTTCCCGAGTCCAGCTGCAGAAAGGCTTTGTATTTTGACT
CCTCTAGAAGTG

>Peaxi162Scf00194g00218.1

ATGGGGAGGCATTCTTGTTGTTACAAACAGAAGTTAAGGAAGGGCCTTTGGTCTC
CTGAGGAAGATGAGAAACTTGTAAAGCATATTACTAAATATGGTCACGGTTGCTGG
AGTTCAGTCCCTAAACTAGCAGGTCTTCAAAGGTGTGGAAAAAGCTGCAGGCTGA
GGTGGATTAACTACTTGAGGCCAGATTTGAAAAGAGGGACATTCTCACAGGAAGA
AGAGAATTTGATCGTTGAACTTCATGCAGTTCTTGGCAACAAATGGTCTCAAATTG
CAGCTAGATTACCTGGACGAACAGATAACGAAATTAAGAACTTATGGAACTCTTCC
ATTAAGAAAAAGCTAAGACAAAAAGGGATTGATCCAAATACTCACAAGCCACTTT
CTGAAGTTGAGAATGAAGAGAAAGCGTCGGCAAACAGTAACAAGAACAATGAGA
AAGTAATTTCTGAAGACTCAAATGATCAGCTCAATTTTATTGAAGCTCATGAGAGT
ATTTCAAAACATATTGGAATTGCAACAGAAAAGTCAAGCACGCTATCTACAATGAC
AAATAATATGGACCGTTATCCACTTATTCATGAGACCAATAATATTGTCCCACCAACT
CATGAATTCTTCACCAAGTCTCCTCATGATTTGGCTAGTTATTTCTCCTTTCAGCAAT

TGAATAATTATAGTCCCAATAGTATTGGCTTATCCATGACTAATTCCAATACCAATAA
TCTTATCTTCAATAATTCTACCAATTCCAAGAATTCACATGACATGGTATCAGATCAA
TTCAATTCTTGTACCATGGCAACTGATCCTGAAGAAATCAAATGGTCAGAATATATG
CAAACCCCATATTTACTAAGTGCTAATCATCATCAAATGTCTCAGCATCAAGACTTA
TACGGCGATGCAAAATCGGAGGCACAATTCATAACACAAGGTTCATTGAATTTGAG
TAATACCACATGGCTTCATCAGAGCCAACAGCAACAATCTTCTCTACAAACTGCAG
ACTTATACAATAAGAATTTTCAGAGGCTACCCGCCGCCTACGGACAATATTCT
>Peaxi162Scf00203g01315.1
ATGAAGACTTGTGCAGTTAGGGACAGTGATGAGAAGGACAAAGGTATGGGCCATC
ACTGCTGCAGCAAACAGAAAGTTAAGAGGGGTCTTTGGTCACCAGAAGAAGATG
AGAAACTCGTTAGACACATCACTACCCATGGCCATGGCTGCTGGAGTTCTGTTCCT
AAACTAGCAGGACTACAAAGGTGCGGAAAGAGTTGTAGGTTGAGATGGATAAATT
ACTTGAGGCCAGATCTAAGAAGGGGTTCATTCACTGAACAAGAAGAAAGAACCAT
AATTGATGTTCATAGAATATTGGGAAACAGATGGGCACAAATAGCCAAACATTTAC
CTGGTAGGACTGATAATGAAGTGAAGAATTTCTGGAACTCTTGCATTAAGAAGAA
ACTTATTGCTCAAGGGTTAGATCCAAATACTCACAATCTCCTTTCAACTCATCAAAA
CAAGAACAACAAAACAAACAATTCATCCAAAACCAAAAGTTATCATCAAGATTCC
ACCTCAGTTTTCACCATTGATACTTCAACAAATAAAGAAGTCATTTCAATGGATATA
AAAGCAACTCTTGCAGCCTTGCCTCCTTTTCTTCACAGTAATAATAATACTTCTAGT
ACGTACCATTACACAACTCCTATTGTTCCCATCATTGAATACCAAAACCCTTCAACC
TTCACATTAAGTGAGAGGAATAATAATGGAAGTACTACTACACAACATTCCGTGTT
GGATTTTGCGAGCAACACTTCAATTAATAGCACTACGAATAATGTGTCATCTTCAAC
GTTAACTCCATTAGAATTTGGTTATATAAATGAGAATTGCATGTGGGCTGGAACTGG
CCTAGAACCAACAACTTTAAATCCTGCCACGGGCGGAACAGAAGAAATGCAAGTA
CAATTACAAGGGGAGCAGTTTCCAATTCAGACAAAGTTTTGTGATCAAGAGGATG
TTTACAAGGTTAATCAAACGGTGGAGAATACGTTTGATAACTCTAACTTTGATTTTG
ATTTTGTGGATTCTGCATTGGTGCCTTGTGGATTGTACAATAGTGTTAATTCTATGGA
TCAACTTTCATGGGATTGTTAG
>Peaxi162Scf00222g00521.1
ATGACATCATATTCTTGTTCTCCTATGGGCACTACTACTACTACTACTTCAATGGGAA

TGTTTTATGCAGATATGAATTCACTTTCCATTACTTCTATAAACTATGTAACAAGTGA
TGGTGGAGTAGGAGAAAATAGTAATGAAACTATTGATCTTAATGCTTCTTGTTGCTT
TAATGAAGAGAAACAAATTACCATGCATAGTAATTTTAGTAGTGGTAATAATGGCAA
AGAAATAGAGAGTGGACAATCAAAGCTTTGTGCTAGAGGTCATTGGAGACCTGCT
GAAGATGCTAAGCTTAAGGAACTTGTTGCTATTTATGGTCCTCAAAATTGGAACCT
TATTGCTGATAAATTGAGATGGTTTAACCAGTTGGATCCAAGAATAAACAGAAGAG
CATTTACAGAAGAGGAAGAGGAAAGACTAATGGCAGCACATAGATTGTATGGTAAT
AAATGGGCTATGATAGCAAGGCTATTTCCAGGGAGAACAGATAATGCAGTGAAAA
ACCATTGGCATGTGATAATGGCAAGGAAATATAGAGAACAATCAAGTGCTTACAGG
AGGAGAAAAATGGGACAATTTGTTTACAGAAGAACAACAACATCATTAGTGGAAG
AAGAAGATTCAAGTTTTGTCAGTAGTAATAGTAGTAGTGGCAAAGAAGTTGTAGCA
GCAACCATGAAAGGAGAAAATCCAACACCAATCATCAGTGGCCAAACTGCTGCTA
ACCCCTTTGCAAGTCTGAAAATTAATAATGATGGTCCTAGTGGTTGGGTAGTTTATG
GACCAAATGGTTCATCCCACATGGCTGCTTCTGCTGGAGAAGCAGCACCTCCTAGC
ACCAATGTCATTGTGGGAATGAAATGA

>Peaxi162Scf00222g00715.1

ATGGGAAAGTCCAAGTGTTGTGATAAAGAGGGGTTGAAAAAAGGACCATGGACA
CCTGAAGAAGACCAAAAACTCTTGCTTAGATGGATAAATTATCTACGGCCTGATAT
CAAGAGAGGAAAGTTTAGTTTACAGGAAGAAAGAACCATCATTCAGCTTCATGCT
CTTCTTGGAAACAGATGGTCAGCAATGGCAGCATACTTGCCTAGCAGAACAGACA
ACGAGATAAAGAACTACTGGAACTCACGTTTGAAGAAAAGATTAACAAAAATGGG
CATTGATCCAATGACTCATAAGCCAAAGACAGACAATGGCAACTCAAGTCAATCA
AAATATGTTGCAAACCTTAGTCACATGGCTCAATGGGAAAGTGCAAGACTAGAAG
CAGAAGCTAGACTTGTTCGTAGATCAAAAGACTCAAAAATGCTCTTCAATAACACT
CACAATTACAACCAAATTACTTCACAACCTTACTATCAACTTCCTTGTCTTGACATA
TTAAAAGCGTGGCAAAGGTCTAGCACAAAACCAACAACAAATGACATTAATGCTA
TTCTTCTTGATGGTAATTTTGCTACTAGGAACAAGAGCCTCGAATCATCAATACCAT
CAACGTCAAATAATTTCTCGGGGAATGTGGTCATGAACAACGTCATAACAACAACT
ACTACTACTACTACAACAGTTGGTGACAATCTTCCTTTGTCTTCAATCAATTGTATG
GATGAAGACCTTTGTCCAAGTAATTTCATGCAAGAGTTCTCAGAGCTATTTCCAGA

ATATAATACATGTGCCCAAAATCCAGAAAATTATTCTACTGGACAATTGGATAATTTT
ATGGGAGGCTGCTTTTTGGATTTTGAAGATAACAAGTACTTTAACTGGAACAATTT
CTCTCATTTGGTCACTTCACCAGTTGGTTCTCCAGTATTTTAA
>Peaxi162Scf00222g00821.1
ATGGGAAAGTCCAAGTGTTGTGATAAAGAGGGGTTGAAGAAAGGACCATGGACA
CCTGAAGAAGACCAAAAACTCTTGTCTTGTATTGAGGAACATGGTTGTGGTAGCT
GGCGTGCTTTGCCTGCTAAAGCTGGAAAGTTTAGTTTACAGGAAGAAAGAACCAT
CATTCAGCTTCATGCTCTTCTTGGAAACAGATGGTCAGCAATAGCAGCATACTTGC
CTAGTAGAACAGACAACGAGATAAAGAACTACTGGAACTCACGTTTAAAGAAAAG
ATTAACAAAAATGGGCATTGATCCAATGACTCATAAGCCAAAGACAGACAATGGC
AACTCAAGTCAATCAAAATATGTTGCAAACCTTAGTCACATGGCTCAATGGGAAAG
TGCAAGACTAGAAGCAGAAGCTAGACTTGTTCGTAGATCAAAAGACTCAAAAATG
CTCTTCAATAACACTCACAATTACAACCAAATTACTTCACAACCTTACTATCAACTT
CCTTGTCTTGACATATTAAAAGCATGGCAAAGGTCTAGCACAAAACCAACAACAA
ATGACATTAATGCTATTCTTCTTGATGGTAATTTTGCTACTAGGAACAAGAACCTCG
AATCATCAATACCATCAACGTCAAATAATTTCTCGGGGAATGTGGTCATGAACAAC
GTCATAACAACAACTACTACTACAACAGTTGGTGACAATCTTCCTTTGTCTTCAATC
AATTGTATGGATGAGGACCTTTGTCCAAGTAATTTCATGCAAGAGTTCTCAGAGCT
ATTTCCGGAATATAATACTGGACAATTGGATAATTTTATGGGAGGCTGCTCTTTGGA
TTTTGAAGATAACAAGTACTTTAGCTGGAACAATTTCTCTCATTTGGTCACTTCACC
AGTTGGTTCTCCA
>Peaxi162Scf00228g00914.1
ATGGATCATCATCATCATGTTAAAGTTGGTTCGAGCCATGGTGGTGGAGCTAATTGT
AATAAGAATAATCAACAACAACATGATGAAGATATGGACCTCCGACGAGGTCCATG
GACTGTTGAAGAAGATTTCGCTCTCATGAATTACATAGCTCATCATGGTGAAGGTC
GTTGGAATTCTCTCGCCCGTTGTGCAGGTTTGAAACGAACTGGGAAAAGTTGCAG
ATTGAGATGGCTGAATTATCTTCGTCCTGATGTTCGACGTGGAAACATCACTCTTGA
GGAACAACTTTTGATTCTTGAACTACATTCTCGTTGGGGCAATCGGTGGTCAAAAA
TTGCACAACATTTGCCAGGAAGAACTGATAATGAGATCAAGAATTACTGGAGAAC
ACGAGTGCAAAAGCATGCCAAACAATTGAAATGTGACGTGAACAGCAAGCAATTT

AAGGATACAATGCGTTATCTATGGATGCCAAGGCTAGTTGAGAGAATTCAAGCAGC
TGCTGCTAATAATACCACAAACTCCAATAACCCCAGCAAAAGCCAAATGGTTCATC
AGACAATTAATTCAAACATGAGTGGTATGTCTGAATTTGTGCCTATTCATGACAACG
TTGTTAGTACTAATAAGCCAAGATTAATGAGTACAGAGAATTCAAGTACGGCAGCA
GCTTCATCAGAAAATTCATTTGGGACACAAGTTTCACCAGTTAACTCTGATCTCAC
TGATTGTTGCTACAATTACCCACCAGTTAATCAAGATTATTTTCAAGTTAGTCATCAT
CAGAGTACTACTAATAATGATCAATTGTGTCATGGAGAATCTTTAACTAGCCCCACG
GGTTACTTTCACCATGGTACTGGATTAGATCAATTCCAGGGAATGGATCAGCAGCA
AAACATGAACAGTAGTCAATGGATGGACGGTGGGAATAATTTTTCCGATAGTTTGT
GGAACATTGACGACATGTGGTTCTTACAGCAGCAATTGAACAACAACAATAACAA
TATCGTCTGA

>Peaxi162Scf00268g00046.1

ATGGGACGTTCACCTTGTTGTGAAAAAGCTCATACGAATAAAGGAGCATGGACTA
AAGAAGAAGACCAACGCCTCATCAACTACATCCGTGCTCATGGTGAAGGATTGCT
AAGATGTGGAAAGAGCTGCAGATTGAGATGGATAAATTACCTAAGGCCTGATCTCA
AAAGAGGGAACTTTACTGAAGAAGAAGATGACTTGATTATCAAACTCCATAGTTTA
CTTGGAAACAAATGGTCTGTTATAGCTGGAAGATTACCGGGAAGAACTGACAATG
AAATCAAGAATTATTGGAATACACACATTAAGAGAAAACTCATCAGCCATGGCATT
GATCCTCAAACTCATCGTCCCCTCAACGCCGCTGCAAACCCCGCCGCTACCACCAC
TATAACCACCAACACAACCAAAAACATCTGCATGGACTTCAGAAACAACGTTGAC
CAAAAACCCCATATTAATATTATCAACAAGACTAATGCCACAGATTCATCAAACAAT
GAAACAAAATGCAGTAGTGTTACAACTGAGGAAACACAACCGTTAGAGCTACCGA
AGAAAAATACACAAGTGATGATAAATCTTGAACTTTCAATTGGGTTGCCATTTCAT
GCCAAGACTGATCATATTTATTCATCCAATTCAGCTGAGTCAACGGCCCCGTACAA
CTTCTTGGCAGCAGCAGCAGCACCGCCGCCAACGGTGGCTGCTGTACCGGTGGTG
GCCATGGCGGAGTTGGTGGCTGAAACAGCTTGTTTATGTTGGCAAATTGGCTTTCA
AGGTGGTCAGTCTTGTGGTAAATGTAAAACCACAACTGGATTTTACAGATTTTGCT
GA

>Peaxi162Scf00269g00088.1

ATGGGAAGATCACCATGTTGTGATAAAAATGGACTGAAGAAAGGTCCATGGACTC

CAGAGGAAGATCACAAACTCATTCAGTATATTCAAGTTCATGGCCCTGGGAACTGG
CGTAACCTTCCGAAAAATGCTGGACTTCAAAGGTGTGGAAAGAGTTGTCGTCTTA
GGTGGACGAATTATTTGAGACCTGATATTAGGAGAGGAAGATTCTCGTTTGAAGAA
GAAGAAACTATTATCCAACTTCATAGTGTTCTTGGCAACAAAACAGATAATGAAAT
CAAGAATTATTGGAATACACATATAAGGAAAAGGCTATTGAGGATGGGAATTGATC
CAGTGACTCATAGCCCTCGTCTTGATTTCTTAGACTTATCATCTCTTCTGAACTCAA
CTCAACTTAATCTTTCCAGCTTACTTGGACTACAAGCACTCGTAAACCCTGAAATT
CTGAAACTTGCAAATACTCTTTTTCTACCACACTCTGAAAATCAAGAACTCTTGTT
ACTTAAGCTTCAAGAAAACCAATTATTGAACCCTCAGCATATTCAAAACTCTGAAG
GGCCTGAAATGTTACTCCAAAAACTTCTACAAAGTCAATTAGTGAATTCCCCATTG
CATAACCAGTCCTCTATTTTCCAACATAATACTAATCAGATTCACAACCAAATTCCT
GAAATACCAAATTGCACCCAACAAAATGTTTCGTGTTCATCTTCCCAGTCCATGCA
AGGTAACATGGGACGTTATTTGATGAATGGACAAATATTCCAAGAAAATATGATGCT
ACCTCCACAAAACTATAGTCATACTTCTACGCCTGATGCATCTGAGAACTCAACATT
TCAGTCCTTAAACAATAGCAGCAATCAGAATAGTCAGAATTTCAGCTTTGATACAC
CTTTGTCAGGCACAGAAGAAGAGAAAGAGAGTTATTGCAGTAATTTCATGAAATTT
GAAATTCCGGAGAGTTTAGATTTTGATGATTTGCTGTAA

>Peaxi162Scf00273g00087.1

TTCAAGTTTCACCCGAAGGACCCTAAAAAAGCAAACGTCATTGTTTGTGCTCTTGG
AAACATCTGTCGCCACTGGCAGTCTCTCCTGTTGGTCTCGTTGCTACTACTCTTGG
CAATTGAACTCTGTACTCTCTCAGTCTCTCTTGCTTTCCTTGATGCAGTTTCCACTC
TGAAGGGTCCTGAAGAAAGAAACATTCTTCTTCAATGTGGTAAAAGTTGCTGTCTT
CGTTGGACGAATTATCTGAGGCCAGACATCAAACGAGGTCCGTTTAGCGAGGAGG
AAGAAAAGCTTATTATACAGTTACATGGCATTCTTGGCAACAGATGGGCTGCAGTA
GCATCACAACTTCCTGGAAGGACGGACAATGAGATTAAGAATTTATGGAATACTCA
CCTGAAGAAGCGCCTGTTTTCGATGGGCATTGATCCTCACACTCATGAACCATCAT
CTGTGCCTAGTGGGCTGGTGAGGAGACCACCTACATCATCTTCAACACGTCACATG
GCGCAATGGGGAAGTGCTAGGCTCCAAGCTGAAGCTCGTCTAAAGATTCACATTTT
TCGATTCCCTCTC

>Peaxi162Scf00274g00625.1

ATGGGAAGACCACCTTGTTGTGATAAAATTGGGGTGAAGAAAGGACCATGGACAC
CAGAAGAGGATATCATCTTGGTTTCATACATTCAACAACATGGTCCTGGTAACTGG
AGAGCTGTTCCCACTAACACTGGTTTGCTTAGATGCAGCAAGAGCTGTAGGCTTAG
ATGGACTAATTATCTCAGGCCTGGTATCAAACGTGGCAACTTCACAGAACATGAAG
AGAAAATGATTATTCACCTCCAAGCTCTTCTTGGCAACAGATGGGCAGCGATAGCA
TCATATCTGCCACAAAGAACGGACAATGATATAAAAAATTACTGGAATACTCATCTA
AGAAAGAAGCTGAAGAAACTTCAAGGGAATGATGATGAGAATAGCAGTAGTACTA
GTCAAGAAGGAATAAGTTCATCAAATATCTCAAAAGGAAAGTGGGAGAGGAGGCT
ACAAACAGATATTCACATGGCTAAAAAAGCTCTTTGTGAGGCTTTGTCCCTTGACA
AACCTATTGCGAATCCTACCCAAACTCCTGTCCAAGAATCTGTTCAACCATGTACTA
CTTATGCATCAAGTGCAGAAAATATTTCTAGGTTGCTTCAAAATTGGATGAAAAATT
CTCCAAAATCATCTCAATTAAGTCAATCAAATTCGGAGAGTACTACTACTCAAAGC
AGCTCCTACAACAATTTTTCAATGGGACAAGTTGGTTCCAGCTCTAGTCCTAGTGA
AGGGACCATAAGTGTTGCTACACCTGAGGGTTTTGACTCTCTCTTTAGCTTCAATT
CATCGTCCGTGGACGAGGGTAATGCTGCGATTTTCCAAGTTGATAGCAAACCAAAT
TTGCCAAATCTCAATGCTGCAAATGGTTTTTTCCTAGAGGAGAGCAAACCAACTTT
GGAGTCACAAGTACCATTAACTTTGTTGGAGAAGTGGCTCTTTGATGATGCTATTA
ATGCACCAGCACAAGAAGAACTAATGGGAATTGGAATGGGAATGGCCTTAGGTGA
AGCTGCTGACTTGTTTTGA

>Peaxi162Scf00276g00412.1

ATGGAAAGGAGAACCAGTGGTCCTAAGAGACGTTCCAGACAGTGGAATGCTGAG
GAGATTTTACTTGCAGCGGAATGTTTCAATGACCGGACAGATGTACAATGCTTGCA
TAGGTGGCAGAAAGTTCTCGATCCCGAACTTGTCAAAGGTTCATGGACTAAAGAG
GAGGATGAAATACTGATTGAATTAGTGAACACATATGGTGCCAAAAAATGGTCCAC
TGTTGCACAACATTTAGCAGGACGCATTGGAAAGCAATGTCGGGAAAGGTGGCAC
AATCATCTTAATCCAGCAATAAACAAAGAAGCTTGGACACAAGAAGAGGAATTGG
CTCTTATCCGTGCCCACCAAATTTATGGAAACAAGTGGGCAGAGTTATCAAAAGTT
TTGCTTGGAAGGACAGACAATGCAATAAAAAATCACTGGAATAGCTCTGTTAAAA
AGAAACTGGACTCGTATTGGGCATCAGGTTTACTTGCACAGTTCCCTTCTCTGTCT
AATATCAACCATCAGAGCCAATCAATCCCTTCTTCTTCTATGAAGTTGCTACACAAC

AGTGACGATGAAAGTGTCCACCAAGACGGAATAGTAAAGGAGGAAGTTTCAGAAT

GCAGTCAAGGTTCGCCTTTGGCTGGCTGTTCCCACTGTACAAGTGACTGGGGCAA

CACATTTGTACATATAAGAGAGAATGATAGGATGTCGGAGGAATCACTTAATAAAA

ACAATGCAAGCTCCAGCGCAGCGCCATGTTCTAGAAACTATACACCAGATTTTCAT

GATGTTTCTTGTTCACTGCCAGAAGTTCCCACCGAACTTGAGGATTCCAAGTTCCT

TGAGCATAATTACTCACATGACCTGGGAAATTCCATTGGGAAAGATTGGCAATTTA

ATAGGGAAGACATACCTAATATATCTCTGGAGGTTATTCAGGAATCCTCAGGGCTTT

TCACGCACCTTCTAAATTGCAATGAAAACCATAATATGGTACCCTTTCCATTGCAAA

CTCTAACGGGATTTGGAACTGCTAATGCTGGAAATGTGGTTGAGGGTCCATATAGG

CCCAATGAAATGTTTTCTAGTGTGGATGGTTGCAGGATCATACACCCTGAGGAAGG

AATTTCTCAATGCTCTCCGTATGAAACTGGGGTTAATGGAAATGGTGCACCTGAAG

ATTCTTTAATTTACCAATCATCAAACGATCTGATCCTTGAAACAAGAGATATGGCTC

CACAAAACTGCAATGCTTCAAGTTTACATGATTTTGAAGCTTCACGCCATCAGGCA

TTTTCTGTTCACTCACAATTTTCTTCAGAGAATAGATCGATCGTGTTTGGTATTGCTT

CAAATCATCCCCTTATTTCTCCGCTTGAAAACCCAATTCAAGAGTCATTCACCAGT

GGATGTGATGGTTTTATATATCCAAGTGAGTCAGCCGCTCCTGCCGACAATGGCAC

AGACAATTCATTCCTGGCAGATCGACTTGATCACACAAATGATTCCTGTAGGCTAG

TTTCTGTGAATGATCTTGGCTCAACTACATCAAATACTGTTCAAAAAGGTATGCTTA

ATGAAAACACATTTGTACCGGAAGAACAGAAAGATGGAGGAGCTTTATGCTACGA

GCCTCCTCGATTTCCAAGCTTGGATGTTCCTTTTTTGAGTTGTGATCTTATACAATCT

GGTTCAGATACACAGCAAGAGTACAGCCCTCTTGGCATCCGCCAATTGATGATGGC

TTCCGCGAACTGCCTTACTCCATTAAGATTGTGGGACTCACCATCAAGAGATGACA

GTCCAGATGCTATCTTGAAAAGTGCTGCCAAAACTTTCACATGGACACCTTCTATAT

TAAAGAAGCGACACCGTCATTTAGTGACACCTTTGTCAGAAAAGAGATGTGAAAA

AAGGCTAGAAAAGGATTTCAATCAGGAATCATTTTCTATTATGACTGCGGAATTTTC

CCGACCACAGGATATGTTTGATCAGTCAGCAAATGAAAAAGCCTCTACTGAAGAC

AAAGAAAATGGGCATCCATCCTCAGAAGCTGGACGAAAGGAGGAGGGTGATGGA

GTAACTGGACTTTCATGTTGTGGAAACTCAGAGAGACGGTTAGATGGTGGTGCTC

ACTACCGTAAAGAGCCCCATGGTAAAAGTGCTGGAGCCAATGATGGAATAGGAAA

CGTAAAACAGCCTCCTGGAGTTCTGGTCGAGCTTAGCTCAAATGACCTTTTCTTCT

CTCCTGATCGTGTCTTAGTCAAGTGTGATAGAGCTACAAGCCTAAGTAATAAAGCT
CTGGCTAACCACTATGCCCGACGACTTGAAGCTGCATCAAAACAAGTGACGGTTT
CATCATCTTTTGAGACTTCATGCTCGTCTGTTGGTTGCTCTCCTGCCGTTCATGGAA
AATGCAGAAGCAGTTTTGTCATAGCTACATCAACTGCATTGGAGAATACAGCTGAA
AATTCTGAAAACGGATTTCGTGCTGAGACTGAAAGCATATTTGGAGAGACGCCTTT
TAAAAGAAGCATTGAATCTCCTTCAGCATGGAATTCTCCATGGTTCATGAATTCTTT
TCTGCGGAGCCCAAGATGTGAAGTAGAACTTGCCTTTGAGGATATTGCGCTTTTTA
TGAGCCCAGGTGACAGAAGTTATGATGCTATTGGGTTAATGAAGCAATTAAGTGAG
CAAACTGCAGCTTCAATTGCAGATGCCCATCAGATTTTGGGAAATGATACTCCAGA
AACAATCCTGTTGCAAACGAATTCCAAAAAACAGAAAGCAGATGAAAATTGCTCC
CTTCTGACTTCAAATGCTGTGAGCGAGAGACGCACACTTGATTTCAGTGAATGTGG
TGGAACACCACCGGCAAAGGGAAAAGGAACTACCAAATTTGACAGTGGCACCAG
CTTTTCAAGTCCATCCTCATACCTGTTAAAATGTTGTAGGTAG

>Peaxi162Scf00284g00077.1

ATGGAGGGTACTACTGGAGACAAGATCAAAGGTTCATGGAGTCCTGAAGAGGACA
ACATGTTGATCAAGTTAGTGGACCAACATGGTCCAAGGAATTGGTCCCTTATTAGT
ACTGGCATTCCTGGTAGATCCGGTAAGTCGTGCCGGTTGCGGTGGTGCAATCAGCT
CAGCCCCACCGTGCAACACCGACCTTTTACACCTTCTGAGGATGCTATTATCCTTC
AGGCACATGCTCTTCATGGCAATAGGTGGGCTACTATAGCCCGCTTATTGCCTGGA
AGAACTGATAATGCTATCAAGAATCATTGGAACTCCACGCTCCGCCGTAAACGCCA
CGGGCCGCCGCTTTCAAGGAGTGGTTCATCGTCTGATCAGTCCAACTCGAAGAGG
CATTGTACTCGTGCATCACAGGAGCAGAGCAGTTGTGGGCTGGATTGTGATGATTT
GGGCCTTGATGGGCCTAATGGGCTTGATGGTGATGAATTGGGCCTTTATGGGCCTG
AGACTTCACTAACGTTGTCGCTACCAGGGGGCGGGTCGATAGATTCACCTATGAAG
GAAGATGTTCCTGTGAAGGAATCCGAGCCGCTGATGAACAGTGATAATAAGGTGA
AGGAGGAGAAACGTACGGTGGAGATTGAAGAGACATGTTTGGTGACAATCATGCA
GCGTATGATAGCACATGAGGTTCGTTGTTACATTGACAAATTACGGGCTCAGGGTG
GGCTTGGAATAGGGCCTGGCGTTGAGTTTGAGGTTCCAAAGAATCCTTAG

>Peaxi162Scf00295g01125.1

ATGGGAAGGTCTCCATGCTGTGATGAGAGTGGTCTCAAGAAAGGTCCCTGGACTC

CTGAAGAAGATCAAAAACTCACCAACTATATCAACAAACATGGCCATGGCAGCTG
GAGAGCCCTCCCTAAACTTGCAGGTCGAACTGATAATGAAATCAAGAATTTTTGGA
ACACTCATTTGAAGAAGAAGCTAATTCAGATGGGATATGATCCAATGACTCATCGA
CCTCGAACCGATATCTTTGATAGTTTGCAACAACTAATAGCTTTGGCAAAGTTGAA
GGAGTTAATGGAACATCACTCATGGGAAGAACAAGCTATGAGATTACAATCAGAA
GTGGCTAAACTTAACTACTTGCAAAATTTTCTACAACCTCACAACACGAGTTTCAA
TGCTATACAAGATATAGAAGCTTACAATCTTTTAAATTCACTATCTTCATTTAAAGAT
AGCCTAGTCTTAGGCACTAATAACCATCTGCAAATTCCCACAATTTCTACTTCTTCA
AGTCTTCAAGCTATTCAAGATTCAATCCCTTTTTCCCATTTGCCTGAATTACAAACC
CCTCGTAGTAATTTCAAAACTTCTCTAAACAAAGATAGTGTTCGACCTCAAAACAC
TGAATTTAATGTTATGAGCCATGGAGAAACGTCACCTACTTCTCCATGGCTTACTTC
ATCTTTATCTCCATCTCCTCCTCCTCCGGTAATAAATGATGAAACAAAAAACGAGA
ATTCATTAGAAGTGATGATCTCGTCTTGCTTGCCTGAAGAGAGGAAGCTCTCAGAC
CATCTTTTTCAGCCAAATAACGAGCGATCACTCAGTAATGAAGGAGCTCCACCTTC
TGTCTGGCCTGACCTTCTTCTCGAGGACTCTTTTTCCAGGATATTGTAAAGTTTTA
A

>Peaxi162Scf00304g00074.1

ATGAAGGTTCTGATAAAGGGAGGAGTATGGAAGAATACAGAGGACGAAGTCTTAA
AGGCCTTGGTGATGAAGTATGGTAAGAACCACTGGGCTCGTATTTCATCCTTGCTC
GTTCATAAGACCGCCAAGCAATGTAAGGCTCGTTGGTATGAATGGCTCGATCCTTC
GATTAAAAAGATTGAATGGACACGAAAAGAAGATGAGAAACTACTTCATCTTGCA
AATCTCATGCCCTCACAATGGAGAACCATTGCACCAGTTGTTGGTCATACACCATC
CCAGTGTCTTGTACGTTATGAGAAGCTTCTTGATGACAATGAGAACTATGACCTTA
GTGATGATGATCCAAGAAAATTGCGTCCTGGAGAGATTGATCCGAATCCAGAATCA
AGGCCTGCTTGTCCTGATCCTGATGATATGGAGGAAGATGAGAAAGAAATGCTTTT
TGAAGCATGGGCTCGGTTAGCCAACGCAAGAGGCAAGAAGGCTAAGAGGAAATC
TAGAGAAAAGCAGCAGCTTGAAGAGGCTCGTAGGCTTGCTTCTTTACAGGAGAGA
AGAGAACTGAGGGCTGCCGGATTAATAGATGTCCATCAAAGGAAGAGAAAAAGG
AGTGGAATTGATTACAATGCTGAAATCCCCTTTGAAAAGAAGCCTCCTCCAGGTTT
CTATGATGTTACTGATGAAGAATGTACTGTAGGACTGCAGCCTAAGTTTCCATATAC

CATTGAAGAACTTGAAGGGGAAAGGAGAGTCGATAAGGAAGCTCGTCTAAGATAG

>Peaxi162Scf00304g00919.1

ATGGTCTTGCTAGCTGCAGCCATGAAGAATATGCAGGAACCGTATGACAAAAACGT
GGGCTTGAATTCAAACAAGGGGGCTTGGTCTCCTGAGGAAGACCAAAAATTGATA
TCTTATATCATGAAATATCGCATTTGGAACTGGAATCAGATTCCCAAATTTGCAGAT
TTAAAGAGAGGTCCATTTAGTATGGAAGAAGTGGAAACCATTATCAGAATGTATCA
ATCGCTTGGTAACAGGTGGTCAGCTATATCTAAAGAACTGCCTGGAAGAACTGACA
ATGAAATCAAAAATTTCTTCCACACTCATTTAAAGAAGCAATTTGGAACAAAGGTT
AATGATCATGTTCAAGTGAAACGTAAGGCATCATGCAAAAAATCTAAGAAAGCTA
AAGAATTGGAGATGAGAACAAGGGGTGAAACTCAAGAGAAACCAAATCAATCAC
TAGATATGTCCTCCAGTGACATCACATTTGATGAAAACCATAACTGGGATTTCACAA
ATTTCTCAGAAACATCATCGGAAGACAACTACTGTACTGTGAATTCACTTGTCAAT
CAAGAAGCAGTTCCAATTATTGACAGCATGGTTATATTAGAGAGCAATCCTACGAC
ATCTGAAAATCATGATCTTTATGTCCAAGATTTCATGGATGAGACTGGTATTAATTC
ATCTAATAAACTGCACAACAAAAACATGGGCACTAATTCAAAGAAAGGACCTTGG
TCTCCTGAGGAAGACCTTAAATTGACATCTTATATCATGAAGTATCGAATTTGGAAC
TGGAACCAGATGCCCAAATTTGCAGGGCTTTCAAGAACTGGAAAAAGTTGCAGAC
TTAGATGGGTCAACTACCTAAGGCCAGATTTAAAGAGAGGACCATTTAGTATGGAA
GAAGTGGAAACTGTGATCAGAATGTATCAATCACTTGGTAACAGGTGGTCAGCTAT
ATCTAAAGAATTGCCTGGAAGAACTGACAATGAAATCAAAAATTTCTTCCACACTC
ATTTAAAGAAGCAATTTGGAACAAAGGTTAATGATCATGTTCAAGTGAAACGTAAG
GCAACATGCAAAAAATCTAAGAAAGCTAAAGAATTGGAGATGAGAACAAGGGGT
GAAACTCAAGAGAAACCAAATCAATCACTAGATATGTCCTCCAGTGACATCACATT
TGATGAAAACCATAACTGGGATTTCGCAAATTTCTCAGAAACATCCTCGGAAGACA
ACTACTGTGTGAATTCACTTGGAGCTGTTCCAATTATTGACAGCATGGTTATATTGG
AGAGCAATCCAACAACATCTGAAGATCCTGATTTTTCTGTCCAAGATTTCAGGGAT
GAGACTAGTATAAATTCATCTAATGTGGATTTCTGGTTGGAGCTATACATGGCAGCA
GAAAATCTGGCA

>Peaxi162Scf00304g01018.1

ATGCAGAAACTGCACAACAAAAACATGGGCACTAATTCAAAGAAAGGACCTTGGT

CTCCTGAGGAAGACCAAAAATTGACATCTTATATCATGAAATATCGAATTTGGAACT
GGAACCAGATGCCCAAATTTGCAGGGCTTTCAAGAACTGGAAAAAGTTGCAGACT
TAGATGGGTCAACTACCTAAGGCCAGATTTAAAGAGAGGACCATTTAGTATGGAAG
AAGTGGAAACTGTGATCAGAATGTATCAATCACTTGGTAACAGGTGGTCAGCTATA
TCTAAAGAATTGCCTGGAAGAACTGACAATGAAATCAAAAATTTCTTCCACACTCA
TTTAAAGAAGCAATTTGGAACAAAGGTTAATGATCATGTTCAAGTGAAACGTAAG
GCAACATGCAAAAAATCTAAGAAAGCTAAAGAATTGGAGATGAGAACAAGGGGT
GAAACTCAAGAGAAACCAAATCAATCACTAGATATGTCCTCCAGTGACATCACATT
TGATGAAAACCATAACTGGGATTTCGCAAATTTCTCAGAAACATCCTCGGAAGACA
ACTACTGTGTGAATTCACTTGGAGCTGTTCCAATTATTGACAGCATGGTTATATTGG
AGAGCAATCCAACAACATCTGAAGATCCTGATTTTTCTGTCCAAGATTTCAGGGAT
GAGACTAGTATAAATTCATCTAATGTGGATTTCTGGTTGGAGCTATACATGGCAGCA
GAAAATCTGGCAGGTTAA

>Peaxi162Scf00305g00045.1

ATGGGGAGACAACCATGTTGTGACAAAGTTGGATTAAAGAAAGGTCCATGGACAG
CTGATGAAGACAAGAAACTCATTAACTTCATTCTCAGCAATGGCCAATGCTGCTGG
AGAGCTGTTCCTAAACTTGCAGTTATATTGGATGATGGCATTGGTGCAGGGCTTTTA
AGATGTGGAAAGAGTTGTAGATTGAGATGGACAAATTATTTGAGACCAGACTTGA
AAAGAGGACTCTTATCAGAATATGAAGAGAAGATGGTCATTGATCTTCATGCTCAA
CTTGGCAACAGGTGGTCAAAGATTGCCTCTCATTTACCTGGTAGAACTGATAATGA
AATAAAAAATCACTGGAATACACACATCAAGAAAAAGCTGAAGAAAATGGGGATT
GATCCAGTTACTCACAAGCCACTATCCACTATTACTAATGACCATCCAAACAAACA
ACAACCAAAAGATTTGCCAGTTATTCAACAAGAAAATTTACAAGAAATAATTATGC
CACCTTCCTCAGTCAATGATGTCTCAGAAATGGATATTGAAACTCCAATTGAGCAA
TCAGCTATTTCAGAAATCAAAGTAGAAGAAGACAACAATAACAAGAACATGGAGA
CTACAAGTTGCAAAAATAATATCAATATTAGTTTTGACTCAACTACAGTGGAAGTCA
ACAATAATGGCTTTTGTACCGATGAAGTCCCATTAATTGAACCCCATGAGATTTTAG
TCCCTAATTCTGAATCAACCCCCTCAACATCATCATCATCATTATCAACTTCTTCTTC
TTCATCATCATCAATCCTTGAAGACTTGAAATTTTTGCCAAGTTTTGATGAATGGCC
AAGTGATTATTACAACATGGAGCAAAACAACATGGGACTTGGCTGGGAAAATGAT

TTCAGTAGCACCTTGGATTTCTTGCTTAATGATGATAATGATATTAATCAATTTCCAA
GAGATGATGAATCTTGGAAGTTTGATCAGCTGTTGTAA
>Peaxi162Scf00311g01112.1
ATGGGAAGAGCTCCTTGTTGTGACAAAGAAAATGTAAAAAGGGGTCCATGGTCAC
CTGAAGAAGATGCAACACTAAAGTCCTACATAGAGAAACATGGTATTGGAGGGAA
TTGGATTGCTCTTCCACAAAAAGCTGGGCTTAGAAGATGTGGAAAAAGCTGCAGA
TTGAGATGGTTAAATTATCTTAGACCAAACATTAAACATGGAGAATTCTCAGATGA
AGAAGACAGAATAATTTGCAGCCTTTATGCTAACATTGGAAGCAGGTGGTCAATAA
TAGCTGCTCAGCTACCAGGCAGGACTGACAATGATATCAAAAACTATTGGAATACC
AAGCTCAAGAAGAAACTCATGGCTCTTGACCTATCATCTCAGCAAATCAGTCCAAA
TTACCAAAGTACTCCTACATATCACTCATCAATTTCAGCTCTTCAATCCTCATCGCAT
AATTCATCACTATATTCTTCTCCATCACCATCTCTCTCATTAAAATCAACAGCTTATA
AATATACCAATAGCCATTTCTATCCTATTTCAACAGCTACTAATTTCCCATGCAATGA
AACCATTATTTCTAGTCCATCAAGTAAATTCTTGAACTCTCCTAGTTATATGTACAAT
GGGGTAGAAGAGAAACAAAGTAACTCCAAGTTCTTGATTACAAATGGTAATGATTA
TGCTGAAAAAAAGCCTAAAGGAAAAGGTTGTTATGACGGGGAAAATTCATTAGAA
TGTAGTTTGGAGGAGATTAAGAAGCTTATTAGCACTACTAGTATCCATGATAACAAC
TTCAACAACTTCTTTAGTGACGAAAACAAGATAGAAGAACAAGTCATGATGTCTA
CTGATGTTGATCATAATTGA
>Peaxi162Scf00330g00418.1
ATGGATGAATTGATGAAATTAGAAGGCTGCTGTACTGAGAATAAGCAATCAACAGC
TGCTATGAGCTCGTCGGTTTCTGAAAATAGTGGTAGTCTAACTGTAAAGTCATTGG
AAGTATGTAGCCCAACGCCTACATCACCAGCTCATCGGAGAGCCACTGGTCCAATT
AGACGAGCGAAGGGTGGTTGGACTCCTGAGGAGGAAGAAAAAAAAAGTTGGCTT
TGTTTAACCACTGATGCATATGCAGCTGAATTCTTTACGGATAGATCAGAAGTGCAA
TGCCTGCATCGATGGCAGAAGGTTCTGAATCCAGAACTTGTAAAAGGACCGTGGA
CTCAAGAGGCTGGTTTTTCTTTTCCTTTATATCAATCTGAGGATGATAAGATCATTG
AACTGGTGGCAAAGTATGGCCCTATAAAATGGTCTGTCATAGCCAAATCATTGCCT
GGTCGAATAGGGAAGCAATGCCGTGAGAGGTGGCACAATCATTTGAATCCAAATAT
TAGAAAAGATGCCTGGACGTTGGAGGAGGAATTAGCACTCGTAAATGCTCACCGG

GTCTACGGGAACAAGTGGGCTGAAATAGCTAAAGTCCTACCTGGAAGGACTGATA
ATTCAATCAAGAACCATTGGAATAGCTCTTTGAAGAAGAAGTTAGACTTTTATTTG
GCAACTGGAAACCTTCCACCTGTTGCTAGAGATGTCCTTCAAAATGGTTGTAAGGA
CACTGTCAAAACAGCTTCAGCTGAAAAGGCACTTGTTTGCTCACATAAAGGGTCA
GATTCAACTGAAGTTGCCTCATCAGGAACAACTGACGTATGTAAACTAGAAAATG
GTGACAAAAACCATCAAGATACCAAATTGCTGGTTGCGAATATCGGTGCTTCAATT
AGTGGACCTCAAAATGGTTCTGATTCTGAAGTGGTTAGGTGGGAGGCAGAGTCAC
CAGTAATAGATACTATCCACGTCAAGCCAATTACACAAATCAGGTTTGAAGGCTAC
GGAATAAATAGTGAAGTTGATCAATTTAAGGTCATTGAAACTCCTTTTTCTTGTGAA
ATACCTACATGTGGTACATTGTACTACGAACCACCACAGCTGGGAAGCTGTATTTC
AGTGGATTCAGACCATCTGAATATTCGATCGCGACTTTGTGAATCTGATGCAAGTCC
AAGTTTGTCACCCAGTTCTTTCTCGACTCCATCTAGTCTGAAGGGTAGTAGCTTGT
ATGCACAAACCCCTGAATCTATTCTGAAAATTGCTGCTAGAAGCTTCCCAAATACA
CCTTCCATATTCAGGAAGAGGAAGTTGAGCTCCACGTCAAGTAATAAAATTGGGA
ATGCAGATGGAGATACAAGCAAGGATGAAGTGCATGATGCTTGTGAGGAGAAACA
AACTGACAGTTCAGAAAAATATGGCATGCATAGGGAAAGCTCTTCAAGAAATCCTT
CTTACAATGGCATCGGGCCATGCAATACTAAAGCTTTTAATGCCTCTCCTCCGTATC
GATTAAGGTCTAAGCGAAAATCCATTTTCAAATCAGTGGAGAAACGGCTTAAGTTT
GCATTCGACAATGAGCAGCCTGATAGTACTGGAATTAGTGATTCAGCTGCTAAAGA
GACGTCACAAGTAGCCAAAGATTGA
>Peaxi162Scf00342g00113.1
ATGAGCATCACAAGCGAAACTGATGACTGGATGACATCCAAAGTTGACATGGATTC
GCCAGACGAAGCTAATGGTGGAGGAAATGTCGGAGGAAGTTTACCACTCAAAAA
AGGTCCCTGGACTTCTGCGGAAGATGCAATTTTAGTGGAATACGTCATGAAACATG
GTGAAGGGAATTGGAATGCTGTCCAGAAGCATTCCGGACTTGCTCGTTGTGGTAA
AAGTTGCCGTTTGCGGTGGGCAAATCACCTGAGACCTGATTTAAAGAAAGGTGCA
TTCACTTTGGAGGAAGAGCGGCTGATAATTGAGCTGCATGCTAAGATGGGAAACA
AATGGGCGCGAATGGCTGCTGAGTTGCCTGGCCGCACAGATAATGAGATAAAGAA
CTACTGGAACACCAGAATAAAGAGACTACAGCGTGCAGGCTTGCCAGTTTACCCT
CCCGATATTTGTTTCCTGGCAAGTCAGAACAAACAAAATGAGGAGTTGGCTGGATT

ATCCTCTACAGATGCACAACATCCTGGTCTCGTGGCAACTAACAATTTTGATATTCC

TCCAGTGGAGTTCAAAAATTTAGAACTCAATCAGCTGTTGTGTCCACCAGCACATG

TTGAAATTCCTGCAAGTAGCTTGCTTGATATTCCTGCAAGTGGCTTCCTAGCCCAG

GTTCATAGCGCTCCCTATAGTAGTACGTCTCTTCTTTCTACAGTGCATCCCTCCAAG

CGTATACGAGGATCAGAATCCATGTTCTCTGGTTCAAATGGCGATCTTTTCCTAGCC

TCCTGTCAATATCAGAATGGTGGTTCTTTGATTGCTCAGCCCTTGGGATTCTCATCA

TACAATCATAATTTAACATATGATGGTCAGCAATCATTCTCGAATGTAATTCCGGGC

AGCCATGCCCCTTTAAATGGCAACTCCTCTACTTCAGAGCCCACATGGGCACTGAA

GGTGGAGCTCCCTTCACTCCAAAATCAGACAGCCAATTGGGACTCACCTTCCTCTC

CTCTTCATTCACTAGAGTCCGTTGATACACTGATTCAGTCACCTCTAGCTGGACAC

AGTGAATCGGTTAGTCTATCACCTAGGAACAGTGGTCTACTGGACGCTGTGCTTTA

TGGATCACAAACTATGAACTCTTCGAAGGATAACTCAAACCAGGCTAAGGAGGAG

ACGTCTGCTGATGCAGTCGGTAATTCATGTCCAGATCTCCTTGAGAAAGGGTGGGA

AACTTATGGCGATCCAATCTCTCCGTTAAGTCGTTTACCTGCATCAGTATTTACTGA

ATGCACCCCTACCACTGGAAACTCATTACATGAGCTCCAGTCAACATCCAGAATGC

CAGGTGAGAATGGATGCAATGTCAAGCAAGAGAATGCTGATCAAGCCCCCTCTGA

TGGGAAGGATGACATGTTGAACCAGACGATCTTTGCATCTAATTGTTCTAGCCCCA

AGACACAGCACACTAAGAACAATCTGGCACTGAAAAATGCTTTTGGTTCTGGCTT

TTTCGATGATTATGGCTGGGACTGCAAGCAAGTCCATTCAGTAGCCACATCATCAG

GTCAAGCTTGTGGACGAGATTCTGCTTGGGATGCCATGTCCGCTATGTAG

>Peaxi162Scf00349g00057.1

ATGAGAACCCCATCATCATCATCAACAACAAGCAACAAAGTAACACCATGTTGCA

GCAAGGTAGGGTTAAAAAGAGGTCCATGGACACCAGAAGAAGATGAAATATTGAC

AAATTATATAAATAAAGAAGGAGAAGGACGGTGGCGAACGTTGCCAAAAAAGGCG

GGGCTCCTCCGTTGTGGAAAAAGCTGCCGCCTTCGGTGGATGAATTACCTCCGTCC

TTCAGTTAAACGTGGCCATATTGCACCTGATGAAGAAGATCTCATTCTTCGCCTCCA

TCGTCTCCTTGGCAACAGGTGGTCCTTGATAGCTGGGAGAATTCCAGGGAGAACA

GATAATGAGATAAAGAATTATTGGAACACTCACCTTAGCAAGAAGTTAATCAGTCA

CGGAATAGATCCTAGAACTCACAAGCCATTGAAAAACTCTAATTCCTCAAGTGATG

ATATTACCAACAAATTAGCTTCTTCTTCTCCTCCTTCATCCTCAAAAGCAAATGATC

TCAATCCAATTCTAAGCCCCACTTACATTTCTAGTTTTCAAATGGAAGAACCATTAG
GAAAAATCAATACTCACCCGGGAGAAATTACTAGTCTTGATGATCAATATCAAAGT
AACGCGATTCTTGCCGAGTATGGTGATGATCTGAATATTGCGGTTACTATTGAGGAA
GATGTTGAAATGAATTGTTGCACGGATGATGTTTTCTCCTCTTTCTTGAATTCTTTG
ATCAATGAAGATATGTTCGCTTGCCAAAACCAACAAACCAACGGGACGTTCCAAG
ATTTTGATCCTTTCATGGCTTCATCATCTACACCTTCATCTGATCAATATAATCCCAG
TTAG

>Peaxi162Scf00354g00068.1

ATGTTTTCAGGGTTAAAACGATGTGGAAAGAGTTGCAGACTAAGATGGCTCAATTA
CCTTAGACCGGACATAAAGCATGGAGGTTTCACTGAGGAAGAAGATAACATCATAT
TAACTCTCTACACTCAAATTGGAAGCAGTTCAATATTGGTGAAAATTAGGTGGTCA
GTAATAGCTTCAAATCTACAAGGAAGGACAGACAATGATGTGAAGAATTATTGGAA
TACTAAGTTGAAGAAGAAACTTTTGGAAGCTGCTGCAACAAATACTCTCAGCAAT
TCAAATGCTTTCACTCATGATAAAACTTATATGAATACTATTCCAAGAACTTGCTATG
GGACTGATTCTACTAATTGTTCTAATATGAAATTCTACACATTTTCACATGCGATGGA
CACAAATTTATTAGGACATTCAAGTCGTCAATTATTCCCTCTGCTTCCTGAGCTTAT
GGAAATTCAAGAAAATGGTATACTTCAGCAAGCTGAAGAAGTCAAGTGCAGCTCC
CTTGAGAACAGCTTTGCATCATTTTCTGGTAATATTGGAGTAGTCCAAGGCAATGA
AGAGGATGGTTCTCTTATGTCTTTGAGCTCTGGAATTTCATCATCTTATTATTATGAT
CTTTTAAATGGCTTTAACTTTCAAGAGAATGTCGTTATGGGAGTTAACACAAACTTT
TCCAATTACTCTTCCAACTCCAACTTGTTGCAGCTGCAAGCTAACATTCCTGACAG
TACTGATCAACCGTTCAAAGGGTTCGAGACTTATTAA

>Peaxi162Scf00362g00831.1

ATGGGAAGATCACCATGTTGTGAAAAAGCACATACAAATAAAGGAGCATGGACTA
AAGAAGAAGATGAAAGGCTAATTGCTTATATTAGAACTCATGGTGAAGGTTGTTGG
AGATCACTTCCTAAAGCTGCTGGACTTTTAAGGTGTGGCAAAAGTTGTCGTCTTCG
ATGGATTAATTACTTGAGACCTGACCTTAAACGTGGTAACTTTACTGATGAAGAAG
ATGAACTCATTATCAAACTACATAGCCTTCTTGGTAACAAGTGGTCACTTATAGCTG
GAAGATTACCAGGAAGAACAGATAATGAGATAAAGAACTATTGGAATACACATATA
AGAAGGAAACTTATGAGTCGAGGTATTGACCCTACAACACACAGGGCTATTAACG

AGTCTAGTACCACCACACAAAAAGTTACCACAATTTCTTTTGGTGCTGGAAATAAA
AATAAAGATATTGAGGATCTGAAGATGATCAATGTCAAAGCTGAATCTGGACTTAG
CCAAGAAGATGAAAATAGTAGCAGCAGCCAATTATTTCAAGAACAGTGTCCTGATT
TGAATCTTGAGCTCAGAATTAGCCCTCCTTACCAACAAAACCAACCAGATCACCAA
GCTTTGAAACAAAGTCCAACAAGGGGCCATTTGTGTTTTGCATGCAGTTTGGGTAT
CCAAAACAGTAACGATTGCAATTGCAGTAATGGTTGCAGTACTAACATGGGTATGA
ATATTGCAAGTTATGATTTTTTAGGATTAAAAGCTAATGGTGTTTTGGACTACAGAA
CCTTGGAGACTACTAAGTGA

>Peaxi162Scf00371g00087.1

ATGGGAAGAACACCTTGTTGTGACAAAAACGGACTTAAGAAAGGGCCATGGACT
GCAGAAGAAGATCAAAAGCTCATTGATTATATTCAGAAGCATGGCTCTGGTAATTG
GAGGACACTTCCCAAGAATGCTGGACTTCAAAGGTGTGGAAAGAGTTGCAGGCTA
CGTTGGAGTAACTATCTAAGGCCAGATATTAAGAGGGGAAAGTTCTCCTTTGAAGA
AGAAGAGACAATCATTCAACTGCATAGTATTCTTGGAAACAAGTGGTCTGCCATTG
CTGCTCGCTTGCCTGGAAGGACTGATAATGAAATCAAAAACTACTGGAATACCAAC
ATTCGTAAAAAACTTTTGAGAATGGGAATTGATCCAGTTACTCATAGCCCTCGTCTT
GATCTTCTTGATCTTACCTCCATTTTCAACCCATCACTCTACAATCCATCTCATATGA
ATAATATGTCCAAGTTGTTAGGTGTACAACCCCTATTAAATCCAGAGATTTTGAGAT
TAGCCACTTCTCTCTTATCTTCCCAGCGCCAAAACCAAAATTTCTTATTGCCAAGTA
ATTTTCAAGAAAATCAATCATGCGATTCCCAAGTCCAAAACCAAATGACCCCATTT
GTCCAGGCTAGCCAAATTCAAATCCCTATTCAAAATATTTCAATTTGCACCCCTTTA
AGTACTCCATGTGTTCCATTTTCTAGTGAAGCTCAGGTTATGCAACAACCAAATATG
GAACAATTCCCATCAAATCTTTCCTACTTTAGCGCACAAAATTGCCAGCAAAATGA
GTGGCAATTAAACAATGGAATTGCATCTAAGATGAATGAACATAATTTTCCTTCACA
AAATTACGTTTTCTCAACTTTATCAACGCCTTCATCAAGTCCTGCCTCATTCAATTC
GAACTCAACATGCATCAACAGCAGCAGCACAACTTCTGAAGGAGAGAGAGAAAG
TTATAGCAGCAGCATGCTGAACTTTGATATTCCAAATATTTTCGATGTTAATGAATTC
TTGTAA

>Peaxi162Scf00377g00925.1

ATGAGTGAGATAATAAAGAAAGGACCTTGGAAAGAGGAAGAAGATGCAGTGTTG

ATAAAGCATGTTAAGAAATATGGGCCCCGAGATTGGAGCTCCATTCGATCCAAAGG
GCTTTTACAGCGTACTGGCAAGTCTTGTCGTCTTCGTTGGGTTAATAAACTTCGCCC
CAACTTGAAAAATGGAGTGAAGTTTTCTGCAGAGGAGGAGAGAACTGTGATTGA
ACTTCAGGCACAGTTTGGGAACAAATGGGCTAGAATTGCTACATATTTGCCTGGAA
GAACTGATAATGATGTCAAGAATTTTTGGAGTAGCCGCCAGAAAAGATTAGCTAAG
ATTCTGCGGAACTCAGCATCACAGCCTAGTAAGCCACAAAAGGATAACAACAAGG
AAGCCCCTGCTCTTCAAAAAGTTCCCTCAGTAGAGGAACCAAAATTGAGTTCACC
AGCAGAGGAAAGGTCTTTACCCATGTCCCAGTGTTGCTCGTCATCCTATATGAATA
ACTCTGACACAATCAACATGGTCCCATTGCCAGAGCTAGAGAATTCAACTTCACTT
CCTTTTGAGCCAAACCTGCTGCAATTTGAGTTCACTCCTAATGACAAGAATTACCA
GTACATTGGGACACAGACGTCACTCTCCTTTCCTCAGATTCCACTCCAAACTGACT
TTGGTCATCCATTGGGAAGTCAAGAATTACCAATGAAACTTGAGGAGACTGACTTC
TTAGATTTTTTCGGGCAACTCAGTACTGCTTCTGACATAGGAAATGTGCAAGTCCC
CCTTGTACCGTTGTGTTCAGGACCAGACAAAAGCTCTGAAATTGTTGTGAAGAGA
GAAATGGACAGCCCCCTTACACAAGATAGCTTCATCGATGACTTCCCCATGGATGT
GTTTGATCTTATTGATCCACTGCCGAGCCCATCCGAT
>Peaxi162Scf00391g00621.1
ATGGGACGTTCACCTTGTTGTGAGAAAGCTCATACTAACAAAGGAGCTTGGACTA
AAGAAGAAGATCAACGCCTTATTAATTATATTCGTACTCATGGTGAAGGTTGCTGGC
GTTCTCTTCCTAAAGCTGCAGGATTGCAAAGGTGTGGAAAGAGCTGTAGATTGAG
ATGGATAAATTATCTAAGGCCTGATCTGAAAAGAGGGAACTTTACTGAAGAAGAA
GATGAATTGATTATCAAACTCCATAGTTTGCTTGGAAACAAATGGTCAGTGATAGC
AGGGAGATTGCCAGGTAGAACTGATAATGAAATCAAGAACTATTGGAACACACAC
ATTAAGAGAAAACTCATCAGTCGTGGCCTTGATCCACAAACTCATCGTCCACTCAA
CTCTACCATCACTACTAACATAACCACTTCTACCAAAAACATCTGCTTGGACTTCAG
AAAAAACACATCAATAGCAGCTACCCCTTTATCTCAAAATGATCAAAAACCAATGT
CAAGTTCAATATCTTCACTTGAAGATACCAAATGTAACAGTAGCACTACTGAGGAA
GTACAGCCGTTAGATTCGCCACCGGCAGCACCTCCGGTTACCGGTGAAGTGATGAT
CAATCTTGAACTTTCAATAGGACTACCAATATTTCAGGCCAAGATGACTGATCAGA
GTAGTTCTATTTCCTCCAATTCAGCTAAGGAGATGGGAGGAAAAACAGTGTGTTTG

TGTTGGCAAGTAGGATCTCAAAATGGTCAGCAGTTTTGT

>Peaxi162Scf00411g00008.1

ATGGTGCAAGAGGAAGTCAGAAGAGGTCCATGGACAGAACAAGAAGATCTTCAA
CTTGTATTTTATGTAAAATTATTTGGAGATCGAAGATGGGATTTTCTTGCCAAAGTTT
CAGGTTTGAAAAGAACAGGAAAGAGTTGCAGGTTACGTTGGGTTAATTACTTGAA
TCCTGATCTTAAACGTGGCAAGATGACTCCTCAAGAAGAACGCCTTGTTCTTGAAC
TTCACGCTAAATGGGGAAATAGGTGGTCAAGAATTGCCCGAAAAATACCAGGGCG
AACTGATAATGAAATTAAGAATTATTGGAGAACCCACATGAGGAAGAAGGCTCAA
GAACAAAGGAAAAAAACTTGTGTTTCTCCATCCTCCTCTTTTTCCAACTGTTCATC
TTCTTCTTCCATCACTCATGAAGAAAATGAGAGAAACTTTTATGATACTGGTGGAC
TCGAGCTCTTGCAATCAGCTGATGGACAAAAGAAAGTAAGTGGTCATGAAAAAGG
CGAGAGCATGAAAGTGTACTCCATGGATGAAATTTGGAAAGATATTGAATTATCGG
AAGAAAATGACACAATTACGAGTAATAAATTACCAGTTATGGCAAATGTCTCACCC
CTGTGGGATTATTGCCCAGCCTCACTATGGATATCTGATGAAGAAGAAAGTAAGAT
GTTTCCACCCTTTAGCGAACATGTCGATGATCATCTGCAGGTTTATAGCTTTGACAA
AAAGAATAGGACATTTTAA

>Peaxi162Scf00420g00240.1

ATGGGAAGGCAACCTTGTTGTGATAGAGTTGGATTGAAGAGAGGTCCATGGACTAT
AGAAGAAGACCATAAGCTTGTCAACTTTCTTCTTAATAATGGTATACAATGCTGGA
GAACTGTTCCCAAGCTTGCAGGCTTGCAAAGATGTGGAAAAAGTTGCAGATTGAG
ATGGATTAATTATTTGAGACCTGATCTTAAAAGAGGAACACTGTCTGAAGATGAAG
AAGATCAAATTATACAGCTTCATGCTCGTCTTGGTAACAGGTGGTCTAAAATAGCAT
CCAATTTTCCAGGGCGAACGGATAATGAGATCAAGAACCATTGGAACACTAGAATT
AAGAAGAGATTAAAGCTTATGGGAATAGACCCTTTAACTCACAAACCACTTGAGC
CAATTGACCACAAAAAATACAAACATCAACCAGATGATCATAGAACAGAATTGTCA
AGAACTGAGAACAGCAATTTTGACAATGCTAATAATGATTTTTCAGTGCCAAATGA
TGAGTACAACAACATGTTGTGTGGAAATTTAGAAGTGGAGTTGTGGAACAAAAGT
TGTAAAACTATGTCAAGTGTGACTTGTTATAGCTCTTCAACTTCTTTGGATGCTGAT
GATTCTATCAATTTATCAACAGTATCAGCTGCTACTACTGAATCTTCAAGTAATTTAG
CACCTGTTGAAGATCAACAAGATTCTATACAACAATGGATGGATTCACTTTTTTCAT

GTTCTGTTAATCAATTAGAAGATGACATGTTCTTCTTGAGAAAATACAATTAA

>Peaxi162Scf00428g00117.1

ATGAGGAAGCCAGAACATGGATCAGTGATGAAGGAGAAAGGAAATAATAACAATA

AGGCTAAACTAAGAAAAGGGTTATGGTCACCAGAAGAAGATGAGAAGCTCATGA

ATTACATGTTAACAAATGGACAAGGGTGTTGGAGTGACATTGCTAGAAATGCTGGC

TTGCAAAGATGTGGAAAGAGTTGTCGTCTCCGATGGATTAATTACTTGAGGCCTGA

CCTCAAACGTGGAGCCTTTTCACCTCAAGAAGAAGAACTCATTGTTCATTTGCATT

CTATTCTTGGAAATAGGTGGTCACAAATCGCAGCTCGTCTTCCCGGGAGGACAGAT

AATGAAATCAAGAATTTTTGGAATTCCACAATAAAAAAGAGGTTGAAGAATAACA

ACAACAACAACAACAACAATAACAGTAGTAACAACAATACACCATCTCCTAACAC

AAGCGATTCATCTTCTGATCTTAGGGTTCTCATGGGAGGATCAATATTTCCCATGCA

AGGCCACGACGTAAATGTAATGGCAGGCTTATGCATGGATAATTCCTCATCTACAAC

CTCAGCATCATCCTTGCAAGCCATAGTTCCAAACAACCATTTTAACCCTTTCCCTCA

GCTTGATAGTACAAATTATGACATAATAGGTGCTGCAGGTTTGTACAATTTGCCACC

ATGTTTAGGTCAATTTGGAAGTAGTAGTGGTCATGATGGCGGCGTTGTGGATTATGG

TGTTGTGGAAGCTTATAGTAGTATGGGGTTAGGAAGTGACTTTTCAGTTCCTCCATT

GGAGAGTGGTAATAAGGGTACAATGGGGGAGAACAGTAATATTGTTAATTTTAGTA

GTAATGTTAGTGCTGTTAATGACTATTCTCTGTTTGACAAAAAGACAAATGGTAAC

AACCAGCAGTTGATGAGTGATAGTAGTGATCAAAGCCTCAAAGTTGAAGATTATAT

GGTTGGTTTTGGGAATAGTCATCATCATCATTGGCATGGAGAAAGCTTAAGAATTG

GAGAATTTGATTGGGAAGGTTTGTTGGCAAATGTTTCCTCCTTACCTTACCTTGATT

TTCAAGTTGAATAA

>Peaxi162Scf00451g00059.1

ATGGGTCGATCTCCATGTTGTGATAAAGTTGGCTTAAAAAAAGGACCTTGGACACC

TGAAGAAGATCAGAAACTCTTAGCTTATATTGAACAACATGGCCATGGAAGTTGGC

GTGCTTTGCCTATCAAAGCTGCTTCTTCAAGTATTACATATACCGCTTATGGATTTGA

TGACTTGCAATTTGTAGGCCTTCAAAGATGTGGGAAGAGTTGTAGGCTCAGATGG

ACTAATTATCTGAGACCTGATATTAAGAGAGGAAAATTCACTCAACAAGAAGAACA

AACCATCATTCAACTCCATGCTCTCTTAGGGAACAGGTGGTCAGCAATAGCCACTC

ACTTGTTCAAAAGAACAGACAATGAAATCAAGAATTACTGGAATACACATCTCAA

GAAACGACTAACAAAAATGGGAATTGATCCAGTAACTCACAAGCCAATGAATGAT
ACCCTTTTGTCCACTAATGATAATGTTCAGTCCAAGAACTCTGCCAATCTCAGCCA
CATTGCTCAATGGGAAAGTGCACGTCTTGAAGCTGAAGCTAGACTCGTTAAACAA
TCAAAATTTCATTCCTATAAATTGGACACACCCCGGACGAGTACATTTTTTGACCAC
TATAGTTCCCAAGAATTGTTAACCCCTTCTTCTTCTTCTAGTCATTTAAAAAATTTGA
TGCCAGTTACATCAGGACTTCAAGATTTCAATTTTGTGGAGAGGTCTACAGGTTTT
TTGCTTTGTACTTCAGCTGATGGGAATTTATCATCAGGTGGCATGGATTCTGATAGC
AGTGGAAATGCTAGTGAACAATGTGAGGATAACAATTATTGGAATAGTATTCTTGAT
TTGGTGAATTCTTCACCTACTGAATCCACAATGTTTTAG
>Peaxi162Scf00452g00412.1
ATGGGTAAAGGGAGAGCACCATGTTGTGATAAAAGCAAGGTGAAGAAGGGGCCA
TGGAGTTCAGAAGAGGATCTCAGGCTCATTACTTTCATTCAGAAGCATGGTCATGG
CAATTGGCGCTCCCTTCCAAAACAAGCTGGGCTACTGAGGTGTGGAAAGAGTTGC
CGTTTGAGGTGGATTAATTACCTAAGACCTGACGTAAAGCGAGGAAATTTCACTCC
ACAGGAAGAGGCCACTATTGTCAAATTGCATCAATCTTTCGGAAACAAGTGGTCA
AAGATTGCATCTTATTTGCCTGGAAGAACCGATAACGAGATTAAAAATGTATGGAA
TACACACTTGAAGAAACGTTTAATGAAAAAAATAGATGGTAATCAAGCTAAAGATG
GTCGTAGTTGTGTTTCACCACCCTTTTCCCCTTCTTCATCAACTTCAGTAGTGTCAC
ATACTAATTGGAGTCAAGATATCAGTACTAAAGCCAATGAGGTAGAATTCAAGGAT
GACATGTTAGCTGAGAAAGAACCTAAAGAAGCTACAAAATCTTGCTCTCCAACTA
CTTCATATGGATCAAATCTCTCCAACTTGAGCCAAGTTGAGATTTCAAGTCCAAAA
GGATTAGATATGGATAATTGGATACTAAGCCCAAAGGTGCATGATATCCATGATGAT
GGAGTGATCGAAATCCCATCAATGGAATCTGATATTGATTTTTGGGACATGTTAGAT
ATTTTGGACCCTTCTCCTACTACTACAACTACTACTTCTAATTCAGACTCCAAACAG
GACAATCAGCCAGTTTGTCAAGGAGTATCAGATTCTGAATGTCAAAAGTGGTTGA
GATACTTGGAAAATGAATTGGGTTTAAGTCAAAGTACTGGAGCCAATTGTTACGAG
TGCACTGATGCTGTTACATCCCAAGACAAAGAAGCCCATCCGTTCACTACTTAA
>Peaxi162Scf00485g00916.1
ATGGGAAGATCACCAATTACTTCTGATAAAAGTGGACTCAAGAAAGGTCCATGGA
CCCCAGAAGAAGATCTCAAGCTCATCCAGTATATTGAAGTTCATGGTCCCGGAAAT

TGGCGTAGCCTCCCTAAAAATGCTGGTCTTCAAAGGTGTGGAAAGAGTTGTCGTC
TTCGCTGGACGAACTACTTGAGACCAGATATTAAGAGAGGAAGATTCTCTTTTGAA
GAAGAAGAAACTATTATCCAACTTCACAGTGTTCTTGGCAACAAGTGGTCAGCCAT
AGCAGCTCGCTTGCCAGGAAGAACTGACAATGAAATCAAGAATTATTGGAATACT
CACATAAGGAAAAGGCTTCTAAGGATGGGACTTGATCCAGTAACACACAGCCCTC
GTCTTGATTTATTAGACTTATCATCCCTCTTCAACTCCACACAACTTAACCTTTCAA
GTTTACTAGGACTACAAGCACTTGTAAACCCTCAATTTTTGAGACTCGCCACTACT
CTTTTGACATCCCACACTGAAAATAATCAAGAAATGTTATTACAAAGACTTCAAGC
AAACCCTACTGTATAG

>Peaxi162Scf00503g00613.1

ATGCAGAATTTGAAGAAAAGTGGAAATAGTAGTGGTGATGTTGCTGCTGCAAAGC
CTAAAGAAAGACATATTGTTTCTTGGTCTCAAGAGGAGGATGATATATTAAGAGAG
CAAATTCGAATCCATGGAACTGACAATTGGACGATCATCGCTTCAAAGTTCAAGGA
TAAAACAACAAGGCAATGCAGAAGAAGATGGTTCACTTATTTGAACTCTGATTTCA
AGAAAGGGGGGTGGTCACCTGAAGAAGATATGCTTTTATGTGAGGCTCAAAAGAT
TTTTGGTAACAGATGGACTGAAATAGCCAAGGTGGTTTCAGGCAGAACGGATAAT
GCTGTGAAGAATCGATTCACTACACTATGCAAAAAGAGAGCAAAACATGAAGCTT
TGGCTAAAGAAAACAGCAATTCATTCATCAACTTAAATAACAAGAGAGTTATATTT
CCAGATGGGCTCAGTATTGACAAAATAACAGAAGCTGCTGCTCCTATTAAAAAGCT
GAGGATGAGCGGTGTTTCAGATGTCCCTCAGGATGGTAGCAGTAAAGGGAACTCT
TTCGGTGATTGTGGAACCACCCATCCATTACTAAGACACCCATTTGCTGTACTAGCT
CAAAACTTCCATAATGCTGCGGGAAACTTGGCATCTCATCAGCAGGTCAACAACAT
GAAAGAATCAACTGAAAATGCAACTGACAGTAAAACTCAAGGAACATTCCTTAAG
AAAGATGATCCAAAGATACACGCTTTGATGCAACAAGCCGAGCTGCTGAGCTCAC
TAGCAATGAAAGTGAATACAGAGAATACAGATCAAAGCCTTGAAAATGCTTGGAA
GATACTCCAAGATTTTCTTCATCAAACAAAGGAAGGTGATATGCTCAAGTTCCAGC
TCCCTGAGATGAATATTGATCTTGATGACTATAAAGACCTGATGGCGGATTCAAGG
AGCAGCAATGAAGGCAGTCGACCATCTTGGAGGCAACCTGCTTTATCCGAAGATT
CTGCTGGCAGCTCTGAGTATAGTACTGGATCAACTCTGCTATCCCATGCACTAGCTG
ATAAAACAGAAGAAAGCCAAGCTGAGGGGTGTGCACACCTTCAGGACATCGAATC

TGAATTACGAAATTCTCAGATGAGTGATCAAGGTGGGATTCATGAATCAGAAAATG
GAACTTCCTGCCGTGTATCCACCACTCCAGATACTTTGCCAGTTTGTGATGAAGAA
AAAGCAAATAATGGACCAGCTACGGCCGAATGTGAATTTCTGAATACAGATTTCAG
TTCTCCTCTTCAAGTGACTCCATTGTTCAGATCTTTAGCTGAAACAATTCCCACTCC
AAAGTTTTCAGAAAGTGAGAGGCAGTTCCTATTGAAAACACTTGGAATGGAGTCT
ATAACCCCACATCCAGGCACAAATCTTTCACAACCTCCATCGTGCAAAAGAGCCCT
CCTCCATAGTCTATAA
>Peaxi162Scf00515g00013.1
ATGGCTTTTCAACTAACCATGCAAGAAGAAGAATTGCGAAAAGGGTCTTGGCACG
AAGAGGAAGACGAGAGACTGGCATCTATTGTTGCCATTTTAGGTGAACGTCGCTG
GGATGCCTTAGCAAAGGCTTCAGGGCTAAGGAGGAGTGGGAAAAGCTGCAGATT
GAGATGGATGAATTACCTTAGACCCAACTTAAAGCATGGTTATATTACTGCAGATGA
AGAACATCTGATTGTCAAACTTCAAAAACAGCTTGGAAACAAGTGGTCAAAGATT
GCTAAACAATTGCCAGGAAGAACTGATAATGAAATAAAGAACCATTGGAGAAGTC
ACTTGAGAAAGAAATCACTTATTTATGAACAAGAATGCTGTGGAAGCAATACAAGT
AAATCAGGACAAATATCATCTGCTTCGAAAAGTGATAGTATCAATACTCCTAATAAT
AGTACTGGTGACTCCATTTCTGGAAAAGATGATTGCTCCTCTGCTGATTCAAATGA
AACAGGATTATCGGATTGGATACCAAGTTGGTCATATGAACAAAGTCAAATGGAAC
ATCACATGTACCTTTGTAGATTAAACTTATGTTTTTGTCATCCTCAGTGTTTTTCTGA
AGATAATAATATTAGTACATGGGGTGATACTTCATCTTCTATCTGGGAACAGTAG
>Peaxi162Scf00516g00673.1
ATGGGAAGATCAGCAGAATCTGATGATATGAGTGGGTTAAAGAAAGGTCCTTGGA
CTCCTGAAGAAGATCAACAGCTACTAGATTACATCCACAAGAATGGCCATGGAAAT
TGGAGAGCTCTTCCTAAACTTGCTGGACTTAATCGCTGTGGAAAGAGTTGTCGCCT
CAGGTGGACTAATTATCTCAGGCCTGATATTAAAAGAGGCAAATTCTCCGAAGATG
AAGAGCGACTCATCATCAAACTCCATTCACTCCTTGGAAACAAATGGTCAGCAATT
GCAACACGTCTACCAGGACGAACTGATAATGAGATAAAGAATTATTGGAACACTCA
TCTGAGAAAGAAGCTACTCCAGATGGGGATTGATCCAGTAACTCATAGGCCTAGAA
CTGATCATATCAATATTTTTAATGCCTTAATTGGGAATATACCTCCTCAGCTGCTTGC
TGCTGCTTCCTCCAACTTTATGAATACCGATAATACTTCCATCAACAATCTCACTAA

CATATTGTTTTCAGATACGGCTCAACAACTAGTTCACCAAATCCAATTGTTACAGAA
CTCTAGTAGCTTATTAGTTCATTCCCTATTTAATAACGCCGCCACGTCAACTGCTTGT
CCCTTAAACATGGAAGGTTTGAACCAAAACTTTGGATTACAAAATCTTTGTGATCA
GAATCAGCTTCAGGAGTACAACTCTATCCTAATGAATTACCAGCAATTTCTATCAAA
CACTAGTGACTCATTAACTGGTTTTGCTCTCAATTCCTCAAACAGTACAGATATTAG
TGCAACTAATGGATGTGATCAACTGATGGTGATGAGTGGTACATGCAGCACATCAT
CATCATGTGGGAATAATGCTGCAAGTGTTACTCATGCAAATGATGATCAAATGAAA
AATATCAGTAAAGCTGCACTTCATCATGACACCCCTGAGTGTCCAAGCAGTAGTAA
CAAAATGCCACAGGGAAACATGATCAGCGATATCTACCCGAAACAAGAACTCGTC
CAGCCTTCATCTGCATCAATGTCCACCTTTGAACCGTGGGGACAAATTATGGGTGA
TGAAGAAGCAAGCGACTCTTACTGGAGAGACATCATAGAGTAA
>Peaxi162Scf00521g00814.1
ATGGCAGATAAAGGACAAAGTTCTTCATCTGTAAATACTCCGGCTGATTCTCAAGA
TGTGGTGGCTCCTCGGATGTTAGTTTCAGGAAAGACATCGAAAGTAGCTGAAATA
GAATTCTCTGAAGAAGAAGAAGACTTGATCATTAGGATGTATAATTTGGTTGGCGA
GAGATGGTCTCTTATAGCTGGAAGAATCCCAGGAAGAAGTGCAGAAGAGATTGAG
AAATATTGGAATACTCGATCTTCAACCAGCCAATAA
>Peaxi162Scf00523g00004.1
ATGAATAGGCTATTTGAGTCAGATTCAGTCAAATTCAGTAAATACTCAAATAGTTTG
TGGAATGCTCTCTCATCTGGTGCTAATTTTAACAGGAGAACTAGTGGTCCTACAAG
ACGTTCCACAAAGGGGCAGTGGACTCCAGAAGAAGATGAAATCCTCCGCAAAGC
TGTCCAACGTTTTAAGGGGAAAAACTGGAAAAAGATAGCGGAATGTTTTAAGGAC
CGAACTGATGTACAGTGCTTGCACAGGTGGCAAAAAGTTCTTAACCCTGAACTTG
TCAAGGGTCCTTGGTCTAAAGAGGAGGATGAAGTAATCGTTGAATTAGTTAAAAA
GTATGGCCCGAAAAAGTGGTCCACTATTGCTCAACATTTGCCAGGGCGTATTGGAA
AACAGTGTCGGGAAAGGTGGCACAATCATTTGAATCCTTCAATAAACAAGGAAGC
TTGGACACAAGAGGAGGAGTTGACTCTGATTCGTGCCCATCAAATATATGGGAACA
AGTGGGCAGAGTTAACAAAATATTTGCCTGGAAGGACAGATAATTCAATAAAAAAT
CACTGGAATAGTTCTGTCAAAAAGAAATTGGACTCGTACTTGGCATCAGGTTTACT
TGCACAGTTCCCTGCTCTACCTAATGTCAACCATCAGAACCAATCAATCCCCTCTTC

TGTGAAGTTGCAACAGAGTAGTGAAGATGATAGTGTTCGTAAAGAAGGAACAGAA
ATGGAGGAAGTTTCAGAATGTAGTCAAGGTTCAAATCTTGCTGGCTGTTCCCAGTC
TACAAGTGACATGGGCAACAGATTTGTACATACAAGAGAGGACCGCAAACTGCTG
GAGGATTCAAATTATAGGATGGACCCAAGCTCCAGCTCAGCACCATGTTCAGAATA
CTATACCCCGGCTTTTGATGATATTACCTTTTCAATGGAGGAAGTGCCTAGCGAACT
TTCCGAATCCAAGTTCCTTGAGCAGAACCTCTCACATGACTGGCGAACTTCCATGG
GGAAAGATTGGCAGTTTAATCCAGATGATGACATACCAGATATTTCTCCTCTGGAGT
TGATGCAGGAATCTTCAGGGCTCTTCATGCAATGTTTAACTGGTAATGGGAATCAC
GATATGGTAACCTTTCCACAGAAAAATGCAATGTCTTTCGGGACGACGACTAATAT
GGGAGGCATGGTTGTGGGTTTAGATAAGCCCACCGAGATGTTTACTTCTGTGGACG
ATTGCAGGATGGTATACCCTGAGGCAGGAATTCCTCAATGCTTTCCTTCTGAAACT
GGTATGAATGGTGTTGATGAAACTGCGGATTCTTTGATTTACCAATCATCGAATTAT
CAGATCTCTGAAGCTGATATGTCTATAGAAAAGTGCAACCCTCTGTGTTCAGATGT
TATGGGAAGTCAACCATTTTCTGTTCCTTCACAGTTTTCTTCAGAGCAAAGCTCCT
CCATGTTTGGTACTGTCTCGAATCAGTTTCATGATCTTTCGCATGGAAACCCAGCAC
TGGAGTCCCACACAAGTAAATGTGATGCTTTTCTATATCCCGTTGAATCTGGTACTC
CTTGTGACAACATAATGAACGATCCTCTCCTGAAAGAGCAACTGCATCAAACAGA
AGATTCTGTACAGCTAGTTTCTGTGAATGACTTTAGCTCCACTCCTCCAAGTACTCT
TCAAACACGTCCATTGGTGAATGAAAATTTGCCTGTGCCAGAAGAGCAGAAGGAT
GCGGGAGCCTTATGCTATGAGCCTCCTCGTTTTCCAAGCTTGGACGTTCCATTTTTC
AGTTGTGATCTTATACAATCTGGTACAGATGCACAGCAAGAGTACAGCCCTCTTGG
CATCCGCCAGTTGATGATGAGTTCTGTGAACTGTCTTACTCCATTTAGGTTGTGGGA
TTCACCGTCGAGAGATGGTAGTCCAGATGCTCTTTTGAGAAGTGCTGCCAAAACTT
TCACAAGCACACCCTCTATATTAAAGAAGCGACACCGTGATTTGGTGTCACCTTTG
TCAGAAAAGAGATGCGAAAAGAAGCTTGAAAGTGACTTCCGGCGAGAAGCATTT
TCTGATCTGGCTAGGGATTTTTCTCGACTAGATGTTATGGTTGACGAGTCTGCAAAC
GAGAAAGCAACAATTTCTTCTGCAACTGCAGATCAAGCGCTGGAACTTCGAGCTT
CATCTGAAGATAAAGAAAACATAAGTCCAACTGAAGACGGAAGAAAGGAGGAGG
ACAAGGGACATAATGGACTTTTGAGCAGCGGAACTTCTGAGAGACAGTTAGATGG
AGCTGATGTTCACTACAAAGAGCATGGAACAAGGGAGGGCAAAAGGGTTGGAGC

CAATGATGCAAAGGGAAAGATAAAACAGCCTTCTGGAGTTCTGGTTGAACTGAAC
ACAAGTGACCTGTTCTTTTCTCCTGATCGATTTGGAGCCAAGTCAGATAGAGCTAC
AAATCTAAGCAATAAAGCTCTAGGAAATCAGTATGCTAGACGACTAGAAGCTGCAT
CAAATCAAGGTTCTGTTTCTTCTTCATTTGAGACTTCATGTTTGTCTGTTATTTGCTC
ACCACGTATACGTGGAAAGAAGGATGGAAGTAGTTTTATCATCACTACGTCAATGC
AATCTGCTACAACTTCAACTGCCTTGGACAACTCAGCTGAAACTTCAGGAAATGG
AGTTGGCGCAGAGACTGTAAACATATCTGGAGAAACACCTTATAAAAGGAGTTTT
GAATCTCCTTCAGCTTGGAAATCTCCATGGTTCATCAACTCTTTTATGTCGAGCCCA
AGGCTTGATACTGAACTTACTTTTGAGGATCTTGCGCTGTTTTTGAGCCCGGGCGA
GAGAAGCTATGATGCTATTGGATTAATGAAACAATTAAGTGAGCAGACTGCTGGGG
CATTTGCAGATGCCCAGGAAGTCTTGGGAGGTGAAACTCCAGAGTCTATCATCCGG
GGAAGGAACTCTAAAAACCAGAAAGCAAATGAAAATCACTCTCTATTGTCAGCAA
ATGCTGTGAGTGAGAGGCGCACGCTTGATTTCAGTGAATGTGGATCACCTGGAAA
AGGAAAGGACGCTGAAAATTTTTGCACAAACGACCGTAGCTTTTCAAGTCCTTCT
TCCTACCTCTTGAAAGGCTGCCGGTAG
>Peaxi162Scf00526g00820.1
ATGGTTGCAATAAAAATTAGACGCGATAAGGATATGAGTATGTCAAGTGAAAGCGA
TGACATGATGACATCAAAAGTTGGTGTGGATTCGCCATCTGTCGAAGAAGCTTGTG
GTGGAGGAAATACCGGAGGAGGTCTACCACTAAAAAAAGGCCCCTGGACTTCTGC
AGAAGATGCAATTTTAGTGGATTACGTCACGAAACACGGTGAGGGGAACTGGAAT
GCCGTCCAAAAGCATTCAGGACTTGCTCGCTGCGGCAAAAGTTGCCGTTTGCGGT
GGGCCAATCACCTGAGACCGGATTTAAAGAAAGGTGCATTCACTCCAGAGGAAGA
GCGGCGCATAATTGAACTGCATGCTAAGATGGGAAACAAATGGGCAAGAATGGCT
GCTGAGTTGCCTGGCCGCACAGATAATGAGATAAAGAACTACTGGAATACCAGAAT
AAAGAGACGACAGCGTGCAGGCTTGCCAGTTTACCCTCCAGACATTTGTTTTCAG
GCGATCAGCGAAAGCAAACAAAATGAAGATTTGGGTACGTTCTCTACAGACGGAC
AATATTCTGATTTCTTGCCGGTGAACAATTATGAGATTCCAGCTGTGGAGTTCAAAA
AACTGGAGTTCAATGAGCCGTTGTGTCCACCATCACTTCTTGATATTCCCGCTGGTA
GCTTGCTTGATATTCCTGCGAGAAGCCTGTTGGCACAGGGTCTTAATTCTGCCTATT
ATAGCCGGTCTTTCCTCTCAACGATGCATCCATCGAAGCGTATACGAGGCTCAGAA

TCTTTGTTCTCTGCTTTAAATGGTGATTCTTCTCCATTGAAAAATGAAGATTCTTTTC

CAACCTGCCATCAATATCAGAATGATGGTACGTTGCTTGCTCAGTCCTTGGGTTTTT

CATCTCCATATAATCAAAATCCAACATCTGATCATCACCCATCATCCTTGGGTGTTCC

TGGCAGCCATGCCCCTTTAAATGGCAACTCCTCTTCTTCAGAGCCCTCATGGGCAA

AGAAGCTCGAGCTCCCTTCACTCCAAAGTCAGATGGCAAGCTGGGGCTTACCTCC

TTCCCCTCTTCCTTCGCTAGAGTCTGTTGATACAATGATTCAGTCCCCTCCAACTGA

GCATACTGAATCATGCAATCTGTCACCTAGGAACAGTGGTCTGTTGGACGCTGTAC

TTTATGAATCGCAAACTATGAGAGCTTCAAAAGATAATTTACACCAGGAGAATTCT

GGTGATGGGGCAGATGATTCATGTCCAGATCTCCATGAGACCGAGTGGGAAGCATA

TGGTGATCCAATCTCTCCTTTAGGTCATTCTGCTGCATCTGAGAAGTTTGACGGGA

AGGACGATACATTGAACCCGTTCTTTTCCAGGCCAGACTACTTGCTTGAATCCAAT

TGTTTTAGTCCTATGCAGAGT

>Peaxi162Scf00540g00619.1

GGTGCAAGTGCTTTTCATCCCTATCAAAATTTACCAAAAAATAATCTTCATGGTTTA

CACCCAAATCTTCCTTTAACTGCTATAGATAGGTTCTTACTAGGTCATGAAAATTATT

TTTCTCCTTTAAGTGGTGCAAATGCAACTAGTAGCCATGCAAATGAGGTTTCTTGG

CCAACTCTAGAGCCAAGTTTTATGGAATTAGGGTATTTTCTCCATGAGCAAAGTGC

TATCAATTACATGGATGATCATCAAGAGAATATCATGATAGTTGAAGCAGAGTCATC

TCAAACAAACAAAAAGGAGAGCGTAAAAAAAGCAAAAGAAGGCTATTCAACAAC

AAACTTGATCAAGGGTCAGTGGACTGATGAAGAAGACAGGAAATTGCTTAGGTTG

GTAAAGCAATTTGGAGTAAGAAAATGGGCTCAAATAGCTGAGAAAATGGAAGCCA

GAGGAGGGAAACAATGTAGAGAAAGATGGCATAATCATCTGCGTCCAGATATCAA

GAAAGATAGTTGGAGTGAAGAAGAAGAGTTGATGTTAGTGGAAGCACATAAGCA

ACTTGGAAATAGATGGGCGGAAATTGCTAAAAGAATTCCTGGAAGGACTGAAAAT

TCTATCAAGAACCATTGGAATGCCACTAAAAGAAGGCAAAATTCAAGGAGAAACA

AAAACAGAAAAAATCCACAAGTTGGCCAAAATGAAGGCAAACATCGCTCTAATGT

TCTTCAAGATTATATTAGAAGCAAATACTTCAATGACGACTCTTCCTCCACCACTAC

CTCACACAAAGAATTTTTACATTATCAGACCAACACCATAACGCCAACTAATAGTA

CCACCACCAATGCAACCCCTTCGTACTCTAATGATGATTCGACATCTTTACTCACTC

ACCAAACATATGATGAAGAATTGCAATTCATGCAAAATTTATTTGGGAAGAATTCG

ATCTTGGATAACAATGGTGCTAAAGCCATTGATCACTCCATGGAGGACAAAGTAAC
TCAAAATCTTTTTGACAATAATAACTCATCATCAAGCTTTTCTGCCTTCAGTTCCTT
GGACTTTAGTTCGGATCCGCTGGCCTCTGAGACAAACATCACAACAAAAAGGGTC
ATACACGATGCTGAAAATTACATGAACGATTATTATAAAGAGTCTACTATACATTGTC
CCGACCTTTATCTTTCCTACCTTCTAGACGCTTCTACTTATTCTTCAATGCCTTTTGC
TGAGGCCTCTGGATATGGAAGCATGAATATGGGAATGTTGATGAATCAAGGTAGTT
GTTCAAGTGGAAACAACAAGGAAGTTGATTTTATGGAGATGGCTTCATCTAATTCT
CAATTCTCTCAAGGAAACTTTACCAAACCCTTCTTTTGA
>Peaxi162Scf00543g00414.1
TCATTCAAAGAAGCAAGATCAGCAAGTAAACAAAGCAAAGGAAAAATGGGAAGA
GCTCCTTGTTGTGAAAAGATGGGGCTGAAGAAAGGGCCATGGACTCCTGAAGAA
GATCAAATTCTTGTCTCTTATATTGAAAAGAATGGTCATGGCAACTGGAGAGCCCT
CCCTAAGCTAGCTGGACTTTTGAGATGTGGGAAGAGTTGCAGACTCCGTTGGACT
AACTATTTGCGTCCAGATATTAAGAGGGGCAACTTCACAAGAGAAGAAGAAGATA
CCATTATCCAGTTACATGAAATGCTTGGCAATAGATGGTCTGCAATAGCAGCGAGAT
TACCGGGACGAACGGACAACGAAATTAAAAATGTATGGCACACTCACTTGAAGAA
AAGGCTTAAAAATTACCAGCCTCCTCAGAACTCCAAAAGACACTCCAAGAACAAC
CATGATTCCAAAGCACCTAGTACTTCTAAAATGTTAGACAATTCAGAAAGTTTTAG
CACCATTCAAGAAAACATTAATGAGCCCATGACCGGTCCGAACTCGCCACAACGG
TCATCTAGCGAGTCATCAACTGTCACGGCCGATTCATTGGCCGCGACAGATGTTAC
AAACGATGATCAAACGTTTATTAAGCACGAGGAAATGGACTCGTATGAAAATTTTC
CAGAGATTGATGAGAGCTTTTGGACGGAGGATTTATCCATGGGAGATAATTTGGAT
CTTGATATGGAGGTTGCTGGTGAAAAATCACAAGTTCAATTTCCATATTCTCATGAC
ATGAAGGAACAAAGTGTCGACATGGTTGGAGCAAAATTAGAGGACGACATGGACT
TTTGGTACAATGTTTTCATAAAGGCTGAGGACTTATTAGACTTACCAGAATTTTGA
>Peaxi162Scf00566g00051.1
ATGGAAAATTTTAACAGATGTTCAACATCAACTTCATCTTCATCTGAATCATCATCT
TCTGAATCTTCATTGAATAAAGCTGAGAGAATAAAAGGACCATGGAGTGCTGAAG
AAGACAAGATATTAACAAAACTTGTTGAACGTTATGGTGCAAGAAACTGGTCTTTA
ATAAGTAAATACATTAAGGGTAGGTCTGGAAAATCATGCAGGCTAAGATGGTGTAA

TCAATTAAGTCCAAATGTACAACACAGACCATTTTCTCATGCAGAAGATGAAACCA
TTTTAGCAGCTCATGCTAAATATGGAAATAGATGGGCTACTATTGCCCGTTTATTACC
AGGTCGAACTGATAATGCTGTTAAGAATCATTGGAATTCTACGTTAAAGAGACGTT
ACCAGCAGCAAAAAAATCACAACACAGTTATTTTTCCTGATATGAAAAATGGATCC
GGGTCGGGTTCTGGATCCGGATCCGGATCCAGGTCGGGGTCCGGGTCATGTATGGA
TTATTTGAATGTTGATGAAAGTCCAAGAGGGACTAATGTGAAAGTTGTTGTAAATA
ACTGTAACAGTGAATATGATGACCCAATGACGGAGTTGTCCCTAGCGCCGCCAGG
GATGGGCGGGGATGAGTTGCCGGAGAGGAAAACAGAGAGTTTTTCTGCGGGATTT
TGGGATGTTATGAGAGACGTAATTGCTAAAGAAGTGAGGGAATATGTCGCTTCGTC
GTTTAATGGAGCTTCTACAGGGTTTCCTTAA

>Peaxi162Scf00575g00016.1

ATGGGAAGGCCTCCTTGTTGTGATAAAATAGGTATCAAGAAAGGTCCTTGGACTCC
TGAAGAAGATATCATCTTAGTCTCATATATCCAAGAACATGGTCCAGGAAATTGGA
GATCAGTCCCTACTAATACTGGTAATTCAGTTGGTTTTACTATCTTTTATATATTATGT
TCTTCTCTCTTGTATTACTTTTTAGAATACTGTTACAATGAAGCAATTTCTTTGTTTT
TAAAAGGGTTGATGAGGTGCAGTAAAAGTTGTAGGCTTAGATGGACAAATTACTTG
AGACCTGGAATCAAAAGGGGTAATTTCACATCACACGAAGAAGGAATGATTATAC
ATCTTCAAGCTTTATTGGGTAACAAATGGGCTGCTATAGCTTCCTATCTACCACAAA
GGACAGACAATGACATAAAGAACTATTGGAACACTCATCTAAAGAAAAAGCTCAA
GAAATTTCAAGCAACAGGTTTGGATTCACAGAGTTTAGCTCCTTCAGATTCTAACA
CTTATCAGTTCTCCTGGAATTTCCATGACTCAAAGTTACAACAGAACCAAAACTCT
AGTACTTCTTCCTCTTCAACCTTGTATGCTTCAAGTACAGAGAATATTTCAAGACTT
CTAGAAGGATGGATGAGATCTTCTCCAAACCCTTCTACAAAGAATATTGATGATGA
GATCTTGCATGAAAATGATCAAAATCAAGAAGTTTATAAAAATTGTGAAAGTTCTT
GGCTTCATGGAGGACGCAATAATACTATTGGAAGTACAAGTATTCCAAAAGAATAT
TACAAATCAGTTATTTCTGAAAAATCAGCTTGTGATCAGAGCGGTGGAGCTGTTGA
AAAGAGTTGTACTAATAATGCTCCTCCATACACATATCTTGAAAAGTGGCTGCTCG
ATGAAAGTGCTGGTCAAGTTGAAGAACTTATGGAACTTCCGACCATATTCACATGA

>Peaxi162Scf00578g00007.1

ATGAAAACTTCTGTTTTTACGTCGTCGGGGGTACTGAGGAAAGGTTCATGGACTGA

AGAAGAAGATATTCTTTTGAGAAAATGTATTGAGAAGTACGGGGAAGGAAAGTGG

CATCAAGTTCCTGTTAGAGCTGGTTTGAATAGATGCAGGAAGAGTTGCAGACTAA

GGTGGATGAATTATCTAAGGCCACATATAAAGAGAGGTGACTTCTCTCCAGATGAA

GTGGATCTTATTTTGAGGCTTCATAAGCTTCTTGGCAACAGGTGGTCACTTATTGCT

GGGAGACTTCCGGGAAGAACAGCAAACGATGTGAAAAACTACTGGAACACTAAC

CTTCTAACGAGGGCAAAGTTAGGTCCTCCTCAGCAACACGATAGGAAATGTCCTA

AAGCAATTAAGACCATGGCCAAGAATGCCATAATAAGACCTCAACCTTGGAACCTC

TCAAAATTAGCAAAGAATAACGTCAGTACTATACACAAAGAAGAACATAGCAAAC

AGGAAATTATCATTGAGAAGCCAACAACGGCCGAAGTCGTGTCCAGAGACGAGA

ACGTTGAATGGTGGACGAATTTATTACTGGATAACAGCAACGGATTTGAGAAGGCA

GCAACTGAAAGCACTTCAGCTTTTCAGAACATAGAAAGTTTGTTAAACGAAGAAC

TATTATCACCATCGATAAATGGTGGAACCTACTATCCCATGCAAGAAACTGGAGAC

ATGGGTTGGAGTGACCTTTCTATTGATGCTGACCTCTGGGAATTACTATAA

>Peaxi162Scf00578g00008.1

ATGAAAACTTCTGTTTTTACGTCGTCGGGGGTACTGAGGAAAGGTTCATGGACTGA

AGAAGAAGATATTCTTTTGAGAAAATGTATTGAGAAGTACGGGGAAGGAAAGTGG

CATCAAGTTCTTGTTAGAGCTGGTTTGAATAGATGCAGGAAGAGTTGCAGACTAAG

GTGGATGAATTATCTAAGGCCACATATAAAGAGAGGTGACTTCTCTCCAGATGAAG

TGGATCTTATTTTGAGGCTTCATAAGCTTCTTGGCAACAGGTGGTCACTTATTGCTG

GGAGACTTCCGGGAAGAACAGCAAACGATGTGAAAAACTACTGGAACACTAACC

TTCTAACGAGGTCAAAGTTTGGTCCTCCTCAGCAACACGATAGGAAATGTCCTAAA

GCAATTAAGACCATGGCTAAGAATGCCATAATAAGACCTCAACCTTGGAACCTCTC

AAAATTAGCAAAGAATAACGTCAGTACTATACACAAGGATGAACATAGCAAACAG

GAAATTATCATTGAGAAGCCAACAACGGCCGAAGTCGTGTCCAGAGACGAGAACG

TTGAATGGTGGACGAATTTATTACTGGATAACAGCAACGGATTTGAGAAGGAAGC

AACTGAAAGCACTTCAGCTTTTCAGAACATAGAAAGTTTGTTAAACGAAGAACTA

TTATCACCATCGATAAATGGTGGAACCTACTATCCCATGCAAGAAACTGGAGACAT

GGGTTGGAGTGACCTTTCTATTGATGCTGACCTCTAG

>Peaxi162Scf00579g00055.1

ATGGTGAGAGCTCCTTGTTGTGAGAAGATGGGGTTGAAAAAAGGGCCATGGACTC

CTAACGAAGACCAAATTCTCATCTCTTACATTCGAAAAAATGGCCATGGTAACTGG
CGAGCACTCCCTAAACAAGCTGGATTATTGAGGTGTGGGAAGAGTTGCAGGCTCC
GGTGGACAAATTATTTGCGTCCAGATATAAAGAGGGGAAACTTCACTAAAGAGGA
AGAAGATACCATAATCCAGTCACATGAAATGCTCGGCAATAGATTACCAGGACGAA
CAGATAATGAAATAAAAAATGTTTGGCACACCCACTTGAAGAAAAGGCTCAAGAA
TTATAAACCTCCTCATAACACCAAAAGACACATCAAGAATGATGATTCGAAGGGTC
CTACTACTTCTGAATCATCCATTAAGTCTGATTTAATAGTGACACACAAAAAAACA
TTAATTCACCCCAACATTCATCAAGTGAAATATCAACTGTGACAGCCGAATCAGTA
GTCATGACGCAGTCGCAGGCGATCAACGATCATGATCAAATGATGATTAAGCATGA
AGAAATGGAGTCCTCGGAGTATTTTCCTGAGATCGATGAGAGTTTTTGGACGGATG
AATTATCAACAGACAATAACTGCAATCACGTTGTGACGGCTGTTCCATTTTCCAGT
GTGAGGGAGGAAAATGTGGACATGTTGACTACAAGAATGGAGGACGACATGGACT
TTTGGTACAATGAATTCATAAAGACTGATGACTTGCAAGAATTACCAGAATTTTAA
>Peaxi162Scf00606g00073.1
ATGGGGAGGCATTCTTGCTGTTACAAGCAGAAGCTGAGGAAAGGACTCTGGTCCC
CTGAGGAAGATGAGAAACTTATAAATCATATTACTAAATATGGTCATGGCTGTTGGA
GTTCAGTCCCTAAACTAGCAGGTCTTCAGAGGTGTGGAAAGAGCTGCAGGCTAAG
GTGGATTAACTACTTGAGACCTGATTTGAAAAGAGGAACATTCTCACAAGAGGAA
GAGAATTTGATCATTGAACTTCATGCAGTTCTGGGGAACAAGTGGTCTCAAATTGC
TGCTAGATTACCTGGAAGAACAGACAATGAAATCAAGAACTTATGGAACTCTTCCA
TTAAGAAAAAACTAAGGCAAAGAGGGATTGATCCAAATATTCACAAGCCACTTTC
CGAGGTTGAGATTGATGAAAAGGAGTTAGCAAACAGCAAGAACAATGAGAAGGC
TTCTGAAGGCTCCAGTGACCTCAATTTTATTGAAGTTCATGAGAATTCAAACTTAC
GAATTGCAACAGAGAAACCAAAGCCATCAGCTTCTTTAGTGACTATGGACCGATAT
CCAAATGGTATGAGCAGTGCAGTCCCACCAACACATGAATTCTTCCTTAATAGGTT
TGTCACCTCACATGAAAGCTCCACCGCTACTTGCAAGCCTCTTGACTTGGCAAGTT
ATCTCTCTTTTCAGCAGTTGAATTATGGCTCAAACATTGGCTTGTCCATGAATCCAA
ACACCAATCCTCTTCTTTTCAACTCCAATTCAAAGAACTCAGAAATGGTTTCTCAT
CATCAGTTCAATTCAAACATGCCAAATGATATTCTCCCTTCCATTTCAAACTCAATT
CTGACATCTCCTGTAGCAGCAGCAGCATGTAGTAGTAATATTGAATTGCAACGAAA

CTCTTCTTTCTTTGATAACAATGCATTCTCATGGGGAGCTGCAGATCATTGTGGTAA
ATCAGAAAAAGGAGCTAATAATATTCATTCATCAGAAAGTGATACCGAAGACATCA
AATGGTCTGAATATCTCCATACACAATTTTTACCAGGCAACACAAATATCCACAGTC
ATCAAACATCGATTCAACACTTATACAGCGAAAAATCAGGCACACAATTCACGACG
GAAGTTTCATTGAGTACTACACCATGGCTGCCGAACCAGCAGCAACAGCCTTCTAT
ACAAACTGCAGACTTATATAATAAGCATTTTCAGAGGCTACCAGATGCCTTTGGAC
AATTTTCTTAG

>Peaxi162Scf00658g00110.1

ATGAATACTACTATTCCCAAGTCGTCTGGACTAGTGAGGAAAGGTGCATGGACTGA
AGAAGAAGATGTTCTTTTGAGAAAATGTATTGAGAAGTTCGGCGAAGGAAAGTGG
CATCAAGTTCCTGTAAGAGCTGGTCTGAATAGATGCAGAAAGAGCTGCAGACTTA
GGTGGTTGAATTATCTAAGGCCACATATAAAGAGAGGTGACTTCTCTCCTGATGAA
GTGGATCTTATTTTGAGGCTTCATAAGCTTCTAGGCAACAGATGGTCACTTATCGCG
GGTAGACTTCCGGGAAGAACAGCAAACGATGTCAAAAATTACTGGAACACACACC
TGCAGAGGAAGTTAATTGCTCCTCCGCGACAAGAGATAAGAAAGTGCAGAGCCCT
TAAGATCACTGAGAACAACATAGTAAGACCTCGACCTCGGACCTTCTCAAATAATG
CACAGAATATTTCTTGGTGCAGCAACAAAAGTATCACAAACAGTACTATAGATAAA
GATGGAAGCAACAATGAATGTATAAGGATTAGTGATAAGAAGCCAATGGCCGAGG
TGTCAAGAGACGATGGAGTTCGATGGTGGACTAGTTTACTAGCTAACTGCAACGA
AAATGATGAAACAGCAGTTGAGAACATGTCATATGATAAGTTACCGAGTTTGTTGC
ACGAGGAAATATCACCAACGATAAATGGTGGAATTAGCAACTGCATGCAAGAAGG
AAAAACTGGTTGGGATGACTTCTCTGTTGATATTGATCACCTATGGAATCTACTTAA
CTAG

>Peaxi162Scf00682g00009.1

ATGGGAAGAGCTCCTTGTTGTGACAAGGCAAATGTGAAGAGAGGACCATGGTCAC
CTGAAGAAGATGCTAAGCTCAAAGACTTCATTCACAAATATGGTACTGGTGGAAAT
TGGATTGCTCTTCCTCAAAAAGCTGGACTAAAGAGATGTGGGAAGAGTTGTAGAT
TAAGATGGCTTAATTATCTAAGACCTAACATCAAACATGGTGATTTTTCTGATGAAG
AAGATAGAATTATCTGCAGCTTGTATTCTACCATTGGAAGCAGATGGTCAATTATAG
CAGCTCAATTACCAGGAAGAACTGATAATGATATCAAGAATTACTGGAACACTAAG

CTTAAGAAGAAACTCATGGGATTAATACCCTCTTCAATTATTCAAAGAAAATCACC
ATATATGTTTCCACCTACTACTCTTCATGCAACACAAGTCCAACCAAATAATTTGTA
CACCCCAAATCTTTCGTACACCAACAATAACATGAATTTTCCTTTAGGTGCTACAAA
TCATCAATACTCATATAATTTTCAGAGTCATCATCAAGATAGTTTGATAAATCCTATG
CAAATTTACCCACAATTGAAAGATAATTTACTAATGTTTGGAGGGACTACAGAAGC
AAGTTGTTCATCATCAGATGGAAGTTGCAGCCAGTTGAGTTTTGGTCATAAAGAAA
TCAAGAAAGAGGATATTATTATGGGTAACTTTTCACATGGTCAAATTTCAAGTGTTG
CATTTGAAGAAAATCAGAATTTTATGAGTTGGGTTGATCAAAAACCAAATGGTTAT
TTTGGGAATAACAATAATCAAAGTGCTGATCAAGTACTACTTCAGTATGATCTTGAT
CAAGAAGTGAAGCAGCAGCAGCTAACAAGTTGTAGCAATGGCAACAATGGTACTA
CTACTAGTGAATGTAATAACAACAGTATGTTCTATTACTGA
>Peaxi162Scf00683g00242.1
ATGGATATGAATGGAGCAACTTATATTAGTGTACGATTTTTAATGGGTTTTGAGTGG
GTTGGATCGGACGCTGATGATGACATCGAGTTGGTTCGTAGTATTCAAGAGCGATT
CGCTTTATCAACTGAGCTTCGTGAGCCTTTAAGTTTGAAGCCTTTGTACTCGATACA
TCCACGTGGCGGCTCAGAGTTTGACGATGATGATGATGATGGCGATGATTTTGAAA
CCCTACGTGCTATTCAGCGCCGCTTTGCTGCTTACACTGACGAGAGAACTAATCCT
GAGGAAGGGTTTCCCGCTTGTGTAGATCGAACCACACATTTTTCTGAAGGATGCAG
TTATGACATTGCTGCTAGGTCTCAAACTCTCATTGAGTGGCATGATGATGACTCTGG
TGCTGAAAACACAGCCGTGTCAGCTAATTCTTCTAGCTTCCCAAAATCAGCACAGG
CATTTGTTGATGCCATCAAGAAGAATAGGTCTTGTCAGAAAATGATTAGAAACAAG
ATGATGCAAGTTGAAGCAAGGATGGAAGAGTTGAAAAAATTAAAGGAACGGGTC
AAGATTCTTAAGAGCTTTCAGCTCACTTGCAGAAAGAGAATGGGGCGGGCTCTGT
CACAGAAAAGAGATGCACGTGTCCAGTTAATTTCTTTGCCAAAACAGAGGTTCAG
TTCAAAGCTCCAGGGAAAAAAGCTGTCTGCCATACATTATGGACCTCCCGAGAATT
CTCATGTTGCTAGCTTTAAGGAGGCATTGACAGAGTTTGCTGTCTCATTGAGTCGT
AAAGAGTGGTCCAAGGAAGAGAGGGAGAAACTTGCCAAAGGCGTGAAACAGCA
ATTTCAAGAGATGTTGCTTCAACACTCAGTTAATTTACGAAGTGATCAGGATGGAT
GTTCTGGAGAGTCTGGTGATCTTGATGACACTATTGCCTCAATCAGAGATCTTGTG
AACAAGATTGCCCCTGAGACCATGAGATTGTTTCTGCCCAAGGTCAATTGGGATCA

AGTAGCTTCCATGTGGCTGAATTGGGAAGACCCATTAATTAAGCATGAAGGCTGGG
ATACAGTAGAGGAAAAAAGCCTTTTGCATGCTGTCCAACAAAAGGAGATGAGTGA
CTGGATTGGAATTGCTGCATCATTAGGAGTATGTAGGACTCCGTTTCAGTGCTTATC
ACATTACCAGAGGAGTCTCAACGCTTCAATCATTAGGCGTGACTGGACAGAGGAG
GAGGATATTAGACTCTGCTCTCTTGTAGAGACTTTTGGTGAGAGTAACTGGCAAGT
TGTTGCTTCCTTCATGGAAGGCCGGACAGGCACACAGTGCTCCAATAGGTGGATA
AAATCACTTCATCCAGCAAGGAAGAGGTCTGGTAAATGGAGTGCTGATGAAGACA
AACGATTGAAAGTTGCTGTGATGCTTTTTTACCCAAAAACTTGGAGGAATATAGGT
CAATCTGTGCCTTGGCGAACACCAATTTGGAAGAAGATAGCTCAGTTTGTACCTGG
GCGGACTCATGTACAATGTAGAGAAAGATGGGTCAATAGCTTAGATCCTTCCTTGA
AGTTGGATGAATGGACAGAAGAGGAGGATTTGAAGTTAAAATCTGCCATAGATGA
ACATGGGTATTCCTGGTCTAAGGTTGCTGCTTGCGTTCCTCCACGTACGGATAACC
AGTGTCGGAGGAGATGGATGGTGCTATTTCCAGATGAAGTGCCTATGCTGCAAGAA
GCTAAAAAGATACATAGAGAAGCTTTTATTTCCAACTTCGTTGACAGGGAGGAGCA
AAGACCCGCTCTCAAACCAGATGATCTAGTTCCGACCCATAAGCTTAGAGATAAAG
CTGGATGTGAGACTACTTCTGCAAATAAAAAAAGAAAACGACGGCCCAGAGCAG
GAAGGGAAGATACGACTCCTAGGAGTGATGCAATATGTGAATTGGAGAAGCAGCA
TTCAGATGGTGAGGAGGGGTTTGAACAGGATTTTCATGATGCTTCAAAAAATAGGA
GACCTTCTAAGCTGCGTGTTAGAAAGACTCAAAATTTTGCACCAAATGATAATGTC
CCAGATGCTTCTGCTTCCAGTCAGGTTGAGTCTATTATTGGAGATGGCAATAAATGC
AAGAGGAGGAAGAGAACCCATTTGCTTATGAAGAAAAAGGCTAAAAATGCTGCCC
TACAAGAAAGTTCATCATCTTTTCCAAATCAGTCTTCGTCAATGACGGCTGCTGAA
GAGGCTGACATTCGAGACAGCAGGAAGACCAATAATATTATGGATGGACGTCATTC
CATAAGGGAATGTGATAATCAGTTGAAGGCCATAGATAATCGGCCGGAAACCACAG
AGGACTGCACGACTCTTGCTTCCTTTATTTCTAAATCTCGTGCAAAGGGGCGTTTG
TTGTCTTCTGCTAAGGTTGTGAAGCATCCTGAGTTAGGTCAAAGTAAGACTATGGC
AGACGCAGACAATAACTCTAGTTGCACGACTCTTGCTTCCTTTATTTCTAAATCTCA
TGCAAAGAGGCGTTCATCGTCTTCTGCTAAGGTTGTGAAGCATCCTGAGTTAGGTC
AAAGTAAGACTATGGCAGACAATACCTCTAGTTCCATATCTGGTCACGATGGGCTG
GGGAAGACGATGACGGAAGATTGTACCAGTTCCAATGGGGTAGTGGGGGCTGAAG

TTGGGGACGACATGCCTCTTTCTCTTTTTATGAGCAGAGCTAAGAGAAAACGTTAA

>Peaxi162Scf00689g00121.1

ATGGATGATGATTTTAGCATAAACTCTAAAAATTATCTTCAAGATTTTGAGCACCTT

GATCTTAATTTTTCTATTCCTACATTCAACAACCCTGATCACAATTTTATGATAGAAA

CAAATAGTTATGACCCTTTTGATCCATTCTCAAATATTGAAGATGATTTAGGAGATTT

TAATTTTTGTTATGAGCGTAAACCATTTGAGGAAAATATTGGAGCAAGTAGTAGCAT

AGTTGTAAACAATATTCATGGAGCTGATGAATTCTCAATCAACAAGGAGAATTTTAT

GGATGTCAACATGTACAATGAGAAGTCATTTTCTAGTGTTTGTTGTGAAGATGTGA

AGCCATTGAACTTTGTTGTGCCTGATGAAAGTTCATCATGTGTCACTGCTGATATTA

ATGGTTCTTGCAAAGAAATTGGTGGAAGAAAGAGTAAGAAGATGAAAAGAAATG

ATAACAACTTATCTTCATCAATGAAAAAGCCTAGTAGAGGGAGGAAAAAATCAAA

ATCCGCCAAAGGACAATGGACGATTGAAGAAGACAGGATTTTGATCCATTTAGTAG

AGAAATTTGGGATTAGAAAATGGTCTCAAATTGCTCAAATGCTCAAAGGGAGGATA

GGCAAGCAATCTCATGCAGAAGTGGGAAATAAATGGGCAGAAATTGCAAAAAGAC

TTCCTGGAAGAACTGAAAATTCCATTAAGAATCATTGGAATGCAACAAAAAGAAG

GCAATTTTCAAGAAGAAAATGTCGTACCAAATGGCCAAGGCCAAGTTCTCTCCTTC

AAAATTATATCAAAAGTTTGAATTTGGAAAAAGGAAGCAGCAAAAAGAATTTTCAT

GACACAAACCCTAATGCAAAAGTCATGAAGGTATCAACTAAGCTAGAGCCAGTTG

ACAATTTTTGCCAAGGCAATAGTATTGAGCAAGTTCATGACTATGGTTTCAGTGAA

GTCCAAGAATTTGCATTGGATGATAAATTGTTTGATGATATTCCTATTGGTCCTTCAA

ATATGGATGACAATATTGAGAAATTCATGGGTTTGGAGATGATTCCTTATGAATTGT

CTACATTGGTGCAAGGTGAAGTCAATAAGGAGATTGATTTGATGGAAATGATTTCT

AGAGTTAATAAGCCATAG

>Peaxi162Scf00700g00732.1

ATGGGGAGGCCTCCTTGTTGTGACAAAACAAATGTGAAAAGAGGTCCTTGGACAG

CTGAAGAAGATGCAAAAATTCTTGCTTATGTTGCTAGTCATGGAATTGGTAACTGG

ACTTTGGTCCCTCAAAAAGCAGGTCTCAATAGGTGTGGAAAAAGCTGTAGGCTAA

GATGGACTAATTATCTGCGTCCTGATCTTAAACATGACAACTTTACTCCTCAAGAAG

AAGAAATCATTCTTGAATTTCACAAAACCGTGGGTAGCAGGTGGTCTTTAATAGCA

AAGCAACTACCTGGGAGAACAGACAATGATGTGAAAAATTACTGGAACACAAAG

CTAAAGAAAAAGCTAGTGAACATGGGAATTGATCCTGTTACACATAAACCATTTTC
TCAAGTCTTTGCTGAATATGGAAAAATCTGCAATCTCCCTATTCAAAATGCAAGAA
ATCATATTATTTTGTCCAATAATTCCACTGGCAAAAACCAAGTCTTTCAAGAACCAA
ATCAATTTCAATTCTCAAATGAAACTCCTCATGATACTATTCAAACACACAGTTTTA
GTGAAAAGGAGAATCCTAGTTGTGTGCAAGTTACTCAAGCTGAGGAAAAGAAATC
TGTGGATTCATTCGTTGATAATATCTTGGCTCGGGACAAGCAAATGCTACTGGATTA
TCCTCCACTTCTAGATGTATACTTGGATTATTGA

>Peaxi162Scf00714g00029.1

ATGAATCAAGTCCTTTTGAGGATATACTTTGGCTACAAGATCATGATACAAGTTAAG
GAGGAAGTTCAGACCCTAGACTTTTGTGGATTTGCTTCTTGTTCATCTTTCTCCGAT
AGTAGTTACGAGGCTAGCACTTCGAGATACTCCTCTGAGCCTGGTTGTCGCAGGAG
CTCAGGTCCAACTAAACGTTCATCTCAGGCAGGATGGACAGAAGAAGAGGATTAT
CTGTTGAGTGAAGTGGTGAAAAGGTACAATGGAAGAAACTGGAAAAAAATAGCT
GAGTGCATGAGTGGAAGGACCGATGTGCAGTGCTTGCATCGCTGGCAGAAGGTTT
TGAATCCTGAGCTTGTTAAGGGTCCTTGGACAAAGGAGGAGGATGACGTGATCGT
CGAGTTAGTTGAGAAATATGGCTGTAAGAAATGGTCTCTTATTGCAAAGTCTTTGC
CTGGTCGCATTGGCAAGCAATGTCGCGAAAGGTGGCACAACCATCTTGACCCAAC
AATAAAAAGAGATGCCTGGACTGAACAGGAAGAATCGGTCCTACTCCACTATCAC
CAAATATACGGGAACAAGTGGGCAGACATTGCGAGGTTTCTGCCTGGAAGGACTG
ATAATGCAATTAAAAATCATTGGAATTCCTCAGCCAAAAAAAGATTGAGCTTGAAT
ATCCAAAGTGAGGGATCTCCGAATTTCTCAAGTCACGAGAAAAAGCTAGAGATCG
GGAAGTATTCACTACAGGCTCAAAGCGTAGAACAAACAATATTTTCAGGTGAGCA
AAAAGGAGTAGATAATGCTGCTGATGCTTGCTCAACTGATTTGACAATTGGATGTA
CTTATTCTGCACAAAATGCTCTGCGCAAGGATACTTCTTTATTTGGAGCCTGTAAAT
CATCAGAAGAAAATGTGAGGGACCTTATAAAGCCTCTTGGTGGAATACAATTTGGC
AAGGCAGAGATTATTCCAATTGGCGAGACAGATAAACCATGCCATTCCAATTTAAA
TCACACTAAAATATTATATCCTCTCTCAACCTCTTCTTTAGAGTTTCCTTTGGATCGG
TCGCACATCACCAGCTTGAGTAGTTCTCAAGTCGAGGCTGTTCATCCTGCTTCTTTT
GGGAGAATGTATGAAACTCCCAAAAGGTCTAGGCATGATGTTAATAATCCTGACCA
AGATTTTTTGAGCTTATCATTGCCTGGCTTTACTGAGGTTCATTCCCAAGGTAGCAA

GAGGAATAAATCATTTGATATGCAATCTTCTTTGGATCTCAATCAGCATGGCTGCCT
GTATTATGAACCACCACAAGTAAAGGACTTGATGATTCCTTTAACAGATGAAAATC
TTAGTAGCGATAACATTATCGGGGCAGATCATGGTCCGCCATTTTGCTCTACACCTC
CTAGTCTTGAATTAACAGTCTCTGGTAATGGTAGCAGCAGTCCAGAATCCATGTTA
AGGAATTCTGCAATGAGTTACACAAAAACTCCTTCAATCATAAGAAAGAAGACTT
CCAAATTTTCAGAAGCTGTGAGGCATTCTAATTGCACAGGCACCACGACACCCATG
TATAGTTTCCTTGGTGATCCTGATAGGGGAGACAGCTCAAACCTGAAGGACAGGAT
ATCTGGATGTAAAACATCAGTTTCGGGAAAATCTCTTGGAAGACGGTTGGAATATG
CCTTTGATATGGAATGGGATGCTTCTAGATGTCGCACACCAGTTTCTGCAGCTCCAC
CTTGTGGACTTAATCTTGGTGCTATTGGTGCTAATACAATCCAAATCATACGAATAG
ATGGTTCTATAAGGGACTTCTTTGACTCATGTCAATTTGAGGCAACGAAGATCAGT
GAAGCAATGACAATGCTGAGACTCTCGAGTCTTGAATAA

>Peaxi162Scf00753g00338.1

ATGAGCAAAGGCATAGTAATGAATAGTTGCTCACATGAAGATGAATTTGAGCTAAG
AAGAGGACCATGGACTCTTGAGGAAGATAACCTTCTTATCCATTACATATCTAGTCA
TGGTGAAGGTCGTTGGAATGCCTTAGCTAAATGTGCTGGGCTAAAGAGAACAGGA
AAAAGCTGTAGACTTAGGTGGCTAAATTACTTGAAACCTGATATTAAACGTGGAAA
CCTCACCCCACAAGAACAACTCTTAATACTTGAACTCCACTCCAAATGGGGAAAC
AGGTGGTCAAAGATTGCTCAACATCTACCAGGAAGAACAGATAATGAAATCAAGA
ACTACTGGAGAACAAGGGTGCAAAAACAAGCACGCCAACTTAAGGTTGATTCAA
ATAGTAAGAAGTTTGTTGAAGCAATTAAGAGTCTTTGGATGCCTAGATTACTTGAA
AAAATGGAGCAACAATGTTCCTCATCAATATCATCCCCTGCTTCTTCCTCCTCAAAA
ATATCCATTGAAAAACAAAGTTCTTCACTTCCTTCTCCATTAATCAACCATCAAGAA
CCTTACAAGTCTCATGATAACACATGCAATTATGACAATGAAAAAACAAACTCACT
AGAACATCCAAGAATCTATACATCAAGTTCCATGATTCAAGAAGAGGGGTACCATG
TGGAGAGTTTCAACCAGCAGCCAGACTTTTCATCTCAAGAAATGTCAATATCAGAA
TGTGAGATGGGAGAAACCAATTGGTTTACAGATGAAATGGGAGGATCATTATGGAA
CATGGATGAGTTTTGGCAATTCAGAAAGCTAGGAGATGTAGACATCTAG

>Peaxi162Scf00763g00441.1

ATGGGGCGCCATTCTAGTTTTGTGAAGGAGAAACTAAGGAAAGGATTATGGTCAC

CAGAAGAAGATGAAAAATTGTACAATTATATCACCAAATTTGGTGTTGGCTGTTGG
AGTTCAGTCCCTAAGCTAGCTGGTCTACAAAGATGTGGAAAGAGTTGTAGATTGA
GATGGATAAATTACTTGAGACCTGATCTTAAAAGAGGAATGTTCTCACAGGAAGAA
GAGGATATGATCATTTCTCTCCATCAAGTTCTTGGAAACAGGACAGATAATGAGAT
AAAGAATTTTTGGAATTCATATTTGAAGAAGAAGCTAATAAAGCAAGGGATTGATC
CAAATACTCACCAGCCACTAAGTGAAAATCAAGTAAGAAATGAGACAGATTGTAA
AGATAAGGCCTCAACTTCAACTCAAATACAGCAACCATTTAACCTTAGTAAAAGAA
ATTTCAACTATGAAGCAAGTAGACAGTTAACAGAAGCTTCAAAAGACCAACTTGT
GAGTAAACAAGTCTTTGATCCTCTGTTTCTGTATGAATTCCAAGCAAATGTCAATCC
AAGTGGCTACAATTTGAGACCTTATGACCATAATCAGAACCAAATTGAGGGGAATA
CAAATTTTGGGTTCTTCTCAATGCCAAGTTTAACAAATTTTGAACAAGGACACATG
ACAGAAACAGATTTTTCTGAAAGTTCAAGTTCAAGAATGAGTTTATTATATATCAGT
GAAGCCAAGGAGAGCTCAAGCAACAGCTCAAATATGATTAGCCATCATAATGCTGC
TGGAATTCAGATGAATGAAATGTTGGAAAATTCTCAAGCTTTGTCATGGGATGTTG
ATAACAAAATTGATTCTCTGTTTCAGTATCCTTATGATGGGATCAAGAATGAAGAAG
ATTTTAATAATCCATTGAGTTCCTTATCTGAAGACTTAAGTGGGGAAAATCTTGATG
TTTTCCACCAAATCTAA

>Peaxi162Scf00777g00521.1

ATGGGTAGACCCCCTTGTTGTGACAAAGTAGGGGTGAAGAAAGGACCATGGACTC
CTGAAGAAGATATAATGTTGGTGTCTTATGTTCAAGAACATGGTCCAGGGAATTGG
AGGGCTGTTCCAACCAAAACAGGGTTGAGAAGATGTAGTAAGAGCTGCAGGCTA
AGATGGACTAACTACCTGAGGCCAGGGATCAAGCGGGGTAACTTTACAGATCAAG
AGGAGAAGATGATTATCCAACTTCAAGCTCTTTTAGGCAACAAATGGGCAGCCATA
GCTTCATATCTACCAGAGAGAACAGACAATGACATAAAAAACTATTGGAATACTCA
TTTAAAGAAAAAGCTGAAAAAACTTCAAGAAACTGGAGATGATCTATTCTCTAGA
GAAAATGGGTATTTCTCATCTTCAAACTCAACTTCTAAGGGCCAATGGGAAAGGAC
ACTTCAAGCTGATATAAATATGGCTAAACAAGCTTTACATAATGCGTTGACCTTGGA
AAATTTAAGCTCATGTGTTAAACAAGAAAGTCAAGTTTCTACTTATGCATCAAGTA
CTGAGAATATAGCACGATTGCTAAAAGGATGGATGGGTTCTTCTACAAACAATTCT
GACTATTCAAAAACTTCATCTAACAACGTTGCGACAGCTGATTCTTCTTCTTGTGAT

GGGACTCCAAGTGCAGAAAGTGAAATCGGTTTGATGGAATCCTTCAAGTCACTTT
TTGGGTTCGAGTCATTTGAATCGTCAAGTTCAGATCAATTTTCTCAAACTGCATCTC
CTGATGCTAGTAAACTTAAAGGTGAAATCAAGAAGGAAGCAAATGAACAAATGCC
TTTGTCCGTTATGCTAGAGAATTGGCTGCTTGATGAAAATACAATTCAAGGAAAAG
ATGATTTGAGTAACTTCTCCTTTGATGAAACTAATGACCTTTTTTAG
>Peaxi162Scf00779g00512.1
ATGGGAAGAGCTCCTTGTTGTGACAAAAATGGACTTAAAAAAGGTCCATGGACAC
CTGAAGAAGATCAAAAGCTCATTGATTACATCAAGGAACATGGTTATGGAAATTGG
AGGACTCTTCCAAAGAATGCTGGGCTTCAAAGGTGTGGAAAGAGTTGTAGGCTTC
GTTGGACGAATTATCTAAGGCCAGATATTAAAAGGGGAAGGTTCTCTTTCGAAGAA
GAAGAGACAATCATTCAACTCCACAGTGTTTTAGGGAACAAGTGGTCTGCAATTG
CTGCACGTTTGCCCGGTAGAACTGACAATGAAATCAAAAACTATTGGAATACACAC
ATCAGAAAAAGGCTTCTGAGAATGGGGATTGATCCAGTGACTCATAGTCCCCGTCT
TGATCTCCTTGATATCTCTTCCATTTTAAATTACCCTGCACTCTACAATAATTCGTCT
CATCATCAAGTGAACTTTTCAAGATTGTTAGGTCATGTGCAACCTTTGGTAAATCCT
GAAGTCTTGAGATTAGCTACTTCTCTTTTATCTTCACAACGCCAAAACTCTAATTTC
TTGATGCCAAATAATGTTCAAGAAAATCAAATATGCATTTCCCAAGTCCAAAACCA
AGTGCACCCAATGGTACAAGTTACGAGCCAAGTTCAAAATCCTATTCAAAATATTC
CGACATGTACCACATTAAGTATTACTCCATCTGTTCCATTTTATGACGAAGCTCGAC
TCATGGAGCAATTTTCATCAAATCTTGTGAATTCTAGTTCACAAAATTGCCAAGTTA
ATGAGTGGCAAAGCAGTGGGATGGCCTCAAATTTAACAGATCAAGATTATTTTAAT
AATCCTTTACAAAATTATGGCTACTATCATGAGCTGGATCAATCTATAAGGGATCCTC
CACTGTCCGATGCTTCAACATTTCAATCCAACGACAGCAACAACTTCAGCTTTCAA
TCTGTTTTGTCAAATTTATCAACACCGTCATCAAGTCCTACACCATTGAATTCCAAC
TCAACTACTACTGAAGATGAGCGGGATAATAGCTACTGCAGCAACATGTTGAACTT
CGATAATATTCCAAATATTTGGGATTCTACCAATGAATTCATGTAG
>Peaxi162Scf00786g00439.1
ATGGATCAAAATCTCCATCACCAGCCCAAGATCATGCACCGATGTTGCAGCCATGA
AGTTGTTTCTTTTGTTTTAATTTGGAAAACAGAAGTTAATAGTATGGAGTGGGAGTT
CATCAGCATGACTAAGCAAGAAGAAGATCTTATTTACAGAATGCACAAGCTTGTTG

GAGACAGGTGGGGACTAATAGCAGGAAGAATACCAGGGAGAACAGCAGAAGAAA
TAGAAAGGTTTTGGATAATGAGACACAGTGATGGCTTTGCACACAAGAGACGACA
ATTGAGAAAAGTGTAG
>Peaxi162Scf00797g00013.1
ATGGGGAAACAACCTTGTTGTGACAACGTTGGACACAATAGGGGTCCTTGGACTG
TTGAAGAAGATCATAAGCTCACTAACTTCATCCTCAACAATGGTATACAAAACTGG
CGCCATGTTCCAAAACTAGCAGGTCTAGTGAGATGTGGGAAGAGCTGTAGATTAA
GGTGGATGAATCATCTAAGACCCGATCTTAAAAAAGGAGCATTTACAGAAGATGA
AGAATATATGCTCATTAAGCTTCAATCTCAACTTGGCAATAGATGGTCCAAAATAGC
CGCGCATTTTCCAGGGCGAACAGATAATGAGATCAAGAATCATTGGAATACAAGGA
TCAAGAAGAAGCTATACACAGAACAAACTAGAGATATTAAAGAAGGAAGTACTAA
ACAGAAATATTCTGGACAACAGAGTTTACCTTCTCTTGAGTATTTAGTGAAGGATC
AACAGCAGCTTAAAGAACCAATAGAGATTTCAGAAATGGGGTCAAATGATGTGAA
CCAAAATTCAGAACCTGTTGAGACAATTTCCATTGAA
>Peaxi162Scf00840g00121.1
ATGGGAAGGACACCTTGTTGTGACAAGAACAAAGTGAAAAGGGGGCAATGGTCT
CCAGATGAAGATGAAATTCTCAAGAATTACCTTCTCAAGCATGGAACTGGTGGCAA
TTGGATTACCCTTCCTCTCAGAGCTGGTTCTCTTCTCTCTCTCTCTCGGCACACACG
TCAAAATTATGTTATATATTATTTAATAATAACCCAAGTTTATGAACTTTTTTTACTAT
GCAGATGGTCCATTATAGCATCTAAGCTACCAGGAAGAACAGACAATGATGTGAAA
AATCACTGGAACACTAAGTTGAAAAAGAAGCTACTAGCAAAAAATGCTAACCCCA
CAAGCAGTTTTGATGAAAGCAATGAAGTTTTTAATACCTCAACACCTAATGCACTT
CTATTTATGGTACCTAAAGAGGAAGAAAAACAAGCACCCTATCAATTTCCATTTCC
AAATATGGACTCTGCTGGGCTCAGTATTAATACCAACAACATTGACCAAAAACATT
TTCCTATTCCAAGTTTCACTCAAGAAAATTCCAAGAATTCTGGAACATCTTCAGTTT
CTTTTGATGAGACTAATTGGTTTGAGAAAGGAGCTAATGATTCATTTGAGGGGTTT
CCATTGGGTTTCAGCACTGATGAGATCATGGATGGTATATGGAGTACTCAACAATCA
TTATTTGAAGAAATTGATTTCCCATCCACTTTTATCTGA
>Peaxi162Scf00847g00211.1
ATGGGAAGAGCTCCGTGTTGTGACAAAGCAAATGTGAAGAAAGGGCCATGGTCA

CCTGAAGAAGATGCAAAACTAAAGGAATATATCGAGAAATCTGGCACTGGAGGCA
ACTGGATTGCTCTTCCACATAAAGCTGGGCTAAGAAGATGTGGAAAAAGCTGTAG
ATTAAGATGGCTAAATTATCTTAGACCAAACATTAAACATGGAGAATTCTCAGATGA
AGAAGACAGAGTCATTTGCACACTTTATGCTAGCATAGGAAGCAGGTGGTCAATAA
TAGCAGCTCAGTTACCAGGTAGGACTGATAATGATATCAAGAATTATTGGAACACA
AAGCTCAAGAAGAAACTCATGGGACTTGTTTCTACATCTCAGAAAATTAGGCCTCT
TCATAATCAGCACCAAATTATCACTAATTATAATTACTACCCACAAATTCCATTTCAT
CAAACTTCATCCTTAGCTTCATCTTCACCATATTCTTCGACAACAACAATTCCATGC
TATGAAAGTACTATTCCTACACTTGCATCAAGTAGTTTCTTGAATACTGCATCAGCT
AGTTGTACTTCAGGCATAAGTGGTAGTACTTCAGGCACTGTGCTACAAGTTCAAGA
AAGTTATGTAGGGGGTCCCACAACTTCTTCTGATGGGAGTTACACTAATCAGATGA
GTCAAGGAAGAGATCAATTAGAGTATGATTATGGTGTTTCAAATGGTGGAGAAAAT
TTGGATTTTCATAACTATCTTTATAATAATGGGATTATTGGTCTAGAAGATCAGAGTA
CTAGTTCCAAGTTCTTGAATATTGAAGCTGCTGATGAAAAGCCAATTATTAATGCAA
ATAGTTGTTATGTTCAACAAGAACAAAATCCGTTAGACTATAGTTTGGAGGAGATT
AAGCAGTTAATTAGCACTAATAATGTTACATGTAACAGCAACTTCTTTCTTGATGAA
AACAAGATAGAAGAAAAAGTCATGATGTACTAT
>Peaxi162Scf00876g00221.1
ATGGGCAGGAAGCCATGTTGTTCTAAAGTTGGCATGAGAAAAGGTGCATGGACTG
CAGCAGAAGATAGGCTGCTGACTAATTACATTGAGCTAAATGGTGAAGGAAACTG
GAGATCTTTGCCTATGAAAGCTGGCCTTCTTAGATGTGGTAAGAGTTGCAGACTTA
GATGGGTGAACTATCTTCGACCAGGTATTAAGAGAGGAAATTTCAGTCCTGAAGA
GGATGATCTTATCATCCGTCTTCACACACTCCTTGGTGGTCGATGGTCTCTAATTGC
TGGAAGATTGTCAGGTAGAACTGATAATGAGATAAAAAATTATTGGAACACCTATC
TCTTGAAGAAACTCAAGGCTGCAGGAATAGAACCAAAAGCTAAAACAAAGAAAT
ATAGTACAAATACTAGGCAAAACAAAGAAATGAAGCGAAGACGGGCAAAAAATA
AGACAGATAATCTGCTACATCAGGCTGAAAAGCCCCGGGGCAAGAAGGAAGATTG
TGAAGTAGTTGAGAAGATCCAAGTGCATCGTCCGAAAGCAATAAGGCTTACAAAG
GGATATTCTTCTTCTACTTCTTCTTCTTTTTCTTCTTCTTCGAGCCAAAGTAGTTGTC
AAGATAATGCACCCAAATTTAATTTTCCATTGAGTTCTGAAGAAGTTGACAATAAG

AAGGAAGAAAAAATAGAAGACAATGCAATTTATGAGAAGCTTCAATTGTTTGATA
AATTGCTGAATGGATGTGATATTTCAACCGAGTGCTCAGAGCAGACTAGCAGTTGT
TGTTACATGCTAGATGAG
>Peaxi162Scf00886g00028.1
ATGGTGAGGGCACCTTGTTGTGAGAAAGTTGGCATAAAGAGAGGAAGATGGACA
GCTGAAGAAGATGAACTACTGCTCAAGTATATTCAAGCTAATGGAGAAGGCTCATG
GAGGTCTCTTCCCAAGAATGCTGGTTTATTAAGATGTGGAAAGAGTTGTCGATTAA
GATGGACGAACTACTTGAGACCAAATTTGAAGAGAGGGAAATTTACTTCAGAAGA
AGATGAAACTATCTTCAAATTGCAATGCTCCTTGGGAAATAGGTGGTCTTTGATGG
CAAGTTATTTACCCGGACGAACTGACAACGAGATAAAAAACTACTGGAATTCTCAC
TTACGTAGGAGAATTTATACCTTTGGGATGAAAAAGAAACCCATCAAGACCGCTGC
AGAGATGCCCAATAAGACAATTGTTGCTGATGGCCTTAATTGTGAATCCTTGAAAA
AACGCGGCAGAGTAAGCCGATCCAAGGCCAAAAAATACAATAACAATACCACCAC
CACTACCACAGCCTATATCTCCACTTTAAAACCCAAGTCCTCAGGTGTTGGAGCCG
GTGGTGGAGCTATATGCTCTGAAGGAGATTCAATTGTTGATGCAGGAATAGGACTA
GACATCCAACAACATGATGAAGATCATGCAGGATCAGCGATAGGTAAGCCAAGGA
ATGAGGAAACAGAAGGCACGAACCAAAAACACATCAATGCTACAGCTGAAAAAC
AAGAAGTGCGCAATGGTATCTTGTCCTTTGAGGAACAAGGACAACAAGTACTGGA
TGAGCATATACTAATTGGACCGCATGAAAAAAATGTTGGTGATGAAACTGTTCATC
TTCAGCAGCCTAACTACTGTTTGCATGATTTTGGTAATCAAGTTAGCTTAAGTGGGG
TTTTGGAAGTTGACGAGGAGAGTCATGAAAATTGGTGGTCCACTATGAATTCTGAT
AATTTCTTGGAGGATGAATTATGGGTTGATCAGTGCAGCAGCTTGGACTTGGAATT
TGGATCCATTGAAGAGTGTGATGACATGTTACTTCCGCTATGGGATGATAATTAA
>Peaxi162Scf00911g00012.1
ATGGGAAGGTCACCTTGCTGTGAGAAACTGGGATTGAAGAAAGGGCCATGGACA
CCTGAAGAAGACCAAAAGCTCATGGCTTGCATTGAAAAACATGGCTGTGGCAGCT
GGCGTGCTTTGCCAGCTAAAGCTGGACTTAAGAGGTGTGGAAAGAGCTGCAGATT
AAGATGGATCAATTATCTAAGACCAGATATTAAGAGAGGAAAGTTCAGTTTGCAAG
AAGAACAGACCATCATTCAACTTCATGCCCTTCTTGGAAACAGGACTGACAATGA
GATAAAAAATTACTGGAACACACACTTGAGGAAGAGATTGACTAAGATGGGCATT

GATCCAATTACTCATAAACCAAAGACCAACATTTTTGGTTCTTCTCAATTAAGTCAC
ATGGCTCAGTGGGAAAATGCTCGTCTTGAAGCTGAAGCTAGGCTTGTTCGTGAATC
CAAGAAACAAATTTCCAATAACAATATTCATGACTTTGGTTCTTTGGCTAATACTAG
ATTAGCTCATCATCAACTATTCAATCAGAGATCCAGAATAAACAACATTGTACCCCT
TCAAGCAAAAGTACCACCATTTCTTGACGTACTAAAAGTATGGCAAGACTCAAATT
GGACAAAGCCAAAAATTACCAAAGACACTTCATCATCATCAGTTCTTGATTCTGTT
TTGACTTCCATGAATAATGGAAGTTTCGATTCTCCAACATCCACGAACAACTTATTC
ATGGCACCAAATAATAATATTCCTAGATTAGTAGACAATTCATGTTTGGTTAATGCA
GGAAGTTTCATGGAAAACCCAGAGGTTGAGATAATTACAAAAGAAGAAGTCCAAA
CAGATCAAGTCCAGATTAATGATATTTCATCAATTAATTATATGAGCATAACAGACCA
ATCTTCAGGATTTCCAAGTTTCATTCAAGGGTTCACGCACTTGGAAAATATTGTTGG
AAGCTCCGAAGACAATCTTGAAGATAACAAGTACAGTTATTGGAATACCATACTAA
ATTCTTGTACCTCACCTGCTGGTTCACCAGTATTTTAA

>Peaxi162Scf00929g00071.1

ATGGAAGGAAGTAGTTCAACTTGTTCATCATCATCATCTGAATCTTCACTGTCAGCA
GGTACTCCTAAAACACCAAGAGATGGAAACAATAACAAACCTGAAAGAATAAAAG
GTCCATGGAGTGCTGAAGAAGACAAGATATTGACAAGATTTGTTGAGCGTTACGG
TGCTAAAAACTGGTCCTTAATAAGCAAATACATAAAGGGTCGTTCTGGAAAATCAT
GTAGGTTACGATGGTGTAATCAGTTAAGTCCAGAAGTGGAACACCGACCATTTTCA
CCAGCTGAAGATGACACCATTTTAGCAGCTCATGCTAAATATGGAAATAGATGGGC
TACTATTGCAAGATTACTTCATGGTCGTACTGATAATGCTGTTAAGAATCATTGGAAT
TCTACGTTAAAAAGAAGGCAACAAATTCGTAATAACGTATCTGTAGTTGAGAGTCC
AATAATTATTATAAGAAGAAATGATATTATTGTTAATGTTAATAACTGGGATGAAGTT
GACCCAATGACAACTTTGTCATTAGCTCCACCGGGTTGTCGGAGAAGAGTTTGCC
GGTGGAGTTTTGGGATGTAA

>Peaxi162Scf00944g00140.1

ATGGGAAGAGCTCCTTGTTGTGACAAAACCAAAGTTAAGAGAGGGCCATGGTCTC
CTGATGAAGACAACACTCTCAAGAACTTTGTTGAGAAAAATGGCACTGGTGGTAA
TTGGATTGCTTTGCCTCAAAAAGCAGATATCAACCATGGAGCCTTCACTGAGGAAG
AAGATAACATTATTTAACTCTCTACAGTCAAATTGGAAGCAGGTGGTCTGTAATAG

CTGCAAATCTACAAGGAAGGACAGATAATGATGTGAAGAATCATTGGAATACCAA
GTTGAAGAAAAAGCTTTTGGCAGCAGCTGAAATCACTAACATTAGCAGCAACTAC
AACTCTGTTTTGAATCACAATTACTGTGCAACAGAGTCTACTTGTTTTATTTCCAAC
ACGTTAACAAGTCTTGTGGAACCAAATTTAACTATGTCATCAAACTTGGTTCCAGC
AATGTTAAATGTAGAAACAAGTTGTCAATTTCCACTACCAGGCCTAATGGAAATTC
AAGAAAATGGTGCAATTACAACACTTCAAGAAGATGGTAATTATCTTATGGATTTTA
GCTCTGGGATTTCATCAACTTACTATGATATATTAAATGGCTTTGATTTTCAAGAGAA
AGTTATAACAGCTGATCCAAATTGTTCTAGTTCTTCTTGTTCAACTTTGGTCCAGCA
GCAAGGTTACATTCCTGACATTGATCAATATCCTTTCAAAGGAGTTGAAAATTAG
>Peaxi162Scf00945g00023.1
ATGGATATTATTACAAAGAAAGGTAAACATATTGATCGTGTTAAAGGTCCATGGAGT
CCTGAAGAAGATGAGTTATTAACACAACTTGTTAATAAACATGGTCCAAGAAACTG
GTCTTTAATAAGCAAAAGTATTCCCGGTAGATCCGGTAAATCATGCAGGTTACGTTG
GTGTAATCAGTTATCACCACAAGTTGAACATCGGGCTTTTACTATTGAAGAAGATG
AAACTATTATTCGGGCCCATGCAAGATTTGGTAATAAATGGGCTACTATAGCCCGTT
TACTTAATGGAAGGACTGATAATGCTATTAAGAATCATTGGAATAGTACTTTGAAGA
GGAAATGTTCTTCTCTTAGTGCTGATGAAGGTAATCAACTTGCTGATCAAATTTTTC
AGAATGATAATCAACCACCGTTAAAGCGATCCGTTAGTGCTGGATCTGCTTTACCG
GTTTCGGGCTTTAATTTTAGCCCGGGGAGCCCGTCGGGCTCCGATAGTGATTCGAG
TTTTCATGTTAGTTCGTCCTCCCAGTGTTGTGTTTTTAAGCCTGTTCCTAGAACAGC
CGGAGTTTTGCCTCCGGGTTTGGCGTGTCCCATGGACACGTCATCCTCTCCGGAGG
CAGGAGATCCGCCTACTTTGCTTAGTCTTGCGCTACCTGGAGTCGACTCAGCTGAA
CACTCAGCCGAGTCGACTCAGGTGAAAGCTTTGCTGCTGCCTCAAGCGACGCAGA
TTCCACCACCACCGCCGCCACTGCAGGCAGTGCCGATTCAGCACGGAGAACAGCA
AGATAAGGTTTTCGTGCCGTTTAGCCAGGAGTTGTTGGGAGTTATGCAAGAAATGA
TAAAAACAGAAGTGAGGAACTATATGATGGGAATTGAACAACACCACCCTCAGCA
GCAACGTCAGCAACAACAACAGCAGCAGCAATATCATCAGCAACAAAATCATCAA
TTGCCTAATGGGATTGGACTTGGATTATGTATACAACAAGCTACTGATGGGTTCAGG
AATACAGCTGCTAATCGCATTTAG
>Peaxi162Scf00978g00214.1

ATGACCAGTGAATGCGTTGACAGGATGACTTCAAAAGTTGGCGTGGATTCGTCAG

CTGTTGAAGAAGCTAGTGGTGGAGGAAATGCTGGAGGAGGTGTCCCACTGAAAA

AAGGTCCCTGGACTAAAGCAGAAGATGCAATTTTAGTAGATTACATCACAAGACAT

GGGGAGGGGAACTGGAGTGCTGTTAGGAAGCATTCAGGACTTGCTCGCTGTGGGA

AAAGTTGCCGTTTGCGGTGGGCAAATCACCTCAGACCAGATTTAAGGAAAGGTGC

TTTCACTCCAGAGGAAGAGCGTCTCATAATTGAACTGCATGCTAAGATGGGAAAC

AAATGGGCACGAATGGCTGCTGAGTTGCCCGGTCGCACAGATAATGAGATAAAGA

ACTACTGGAACACCAGAATAAAGAGACAACAGCGTGCAGGCATGCCCATGTACCC

TCCAGATATTTGCTACCAGGCGTTTCGTGAGAACAAACAAAATAAGGAGCTGGGC

ACATTCTCGTCTGCAGATTCACATCCTGATTTCTCACCTATTAACAGTTTCGAGATT

CCAGCTGTGGAATTCAAAACTTTGGAACTCAGCCAGCAACGGTATCCTCCGGCAC

TTCTTGATATTGCTGCTAATAGCTCTCTTGATATTCCTGCAACTAGCCTGCTGGCCCA

GGGTCTTAATTTTTCCTGCAATACTCGGTCTTTCCTCTCAACAATGCATCCGTCCAA

GCGTATACGAGGATTAGAATCTTGGTTTTCTGGTCTAAATGATGATTTTTCCAAGC

TTGTCATCAATTTGAAAATGATGGTTCATTGTTTGCTGAATCCTTAGGGTATTCTTCT

CCTCATACCGATAATCTGATATCTGTTCATCACCCAGCATTCTCGGGTGTATTTAATG

GCAGCCATGCCTCTTTAAATGGCAACTCTTCTTCAGAGCCCAAATGGGCTAAGAAG

CTGGAGCTCCCTTCACTCCAAACTCAGATGGCAAGCTGGGGTTCACCTTCCCCACT

TCCTTCACTAGAGTCCTATGATACTTTGATTCAGCTCCCTCCAAGCGAGCATATTGA

TTCGGGCAGTCTGTCACCTGGGAACAGCGGTCTTTTGGATGCCGTACTTTATGAAT

CACAAGCATTCAGAGCTTCAAAGAATAACTTACATCAGGAGACTTGTGATGTAGTA

GATGATTCATGTCCTGATGTCCAAGCGACATGGGGAACTCACAGTGGCCCAAACTC

TCCTTTAGGTCATTCTGCTGCATCAGTGTTTAGTGAATACACCCCCATCTTTGGAGG

TTCATTGCATGAGCCCCGATCGGTAGCGTCACTGTTTGGTGAGAATGGATGCAAGA

TTAAGCAAGAGGATGTTGACTTAGCTCCCACTGATAGGAACAATGATGTATCAAAC

CACCAGATCTTTGCTTGGACTGAGTCGTGCTTTGCTCCAACTGTTTTGGTCCCACA

ACAGAATAGGGTCCTTTGCCTAACCCTCCTTCCGTTTGTATCACCACCGTCTGAAA

GCTGGGCCCAGACACTTCTAGATTTTCCGCCTCCACTGCTACAATCATCCTCACAA

CCAGCCACTATTTTAAGAAGCTCTGAC

>Peaxi162Scf00986g00227.1

ATGGTACAAGAGGAAATTATAAGAAGAGGTCCATGGACTGAACAAGAAGATTTTC
AGCTAGTATTTTATGTGAACCTATTTGGTGATCGACGCTGGGATTTTCTGGCGAAAG
TTTCAGGTTTGAAAAGAACTGGCAAGAGTTGCAGATTACGTTGGGTTAATTACTTG
AATCCTGGTCTCAAACGTGGCAAAATGACCCCTCAAGAAGAACGCCTTATTCTTGA
ACTCCATTCCAAATATGGAAATAGATGGTCGAAAATTGCTCGGAAATTACCAGGGC
GAACCGATAATGAAATAAAGAATTACTGGAGAACTCACATGAGAAAGCAAGCTCA
AGATCAAAGGAAAAATGCTTTCATCTCTCCATCTTCATCCTTCTCAAACTGTTCATC
TAATTCCTCTGCCAATAGTCCTGCTGTGGACTCAATTCCCATCACTAAACAAAACA
AAAGAAACCTGGAAACTGCAGAAGAAAAGAAAATATATAATCATGAAGAAGCAG
AGGAAAACCAGAATATGGTCTACACCATGGATGAAATTTGGAAAAATGTTGAATCA
TCACAAGAAACTGAAACAATGAGTAAATTACCAGTTATGGCTTCACCAATATGGGA
TTATTGCCCTGATTCATTGTGGATGACTGATTTTAATTACTTTGACAATCAAGATATC
CCTTTTTTTACTGGCTAA
>Peaxi162Scf01007g00117.1
ATGAGTGAACAAATGGATAGCTGGGGAATGACAAAACAAGGGTGGAGAAAGGGA
CCTTGGACTTCTGAAGAAGACAGATTACTCATTGAATATGTTAATTTGCATGGGGA
AGGCAGATGGAATTCTGTTGCTAGGGTTGCAGGGTTGAAAAGGAATGGGAAAAGT
TGTAGGTTGAGATGGGTGAACTACTTAAGGCCAGACCTAAAAAAGGGACAAATTA
CTCCTCATGAAGAAAAATTAATTCTAGAGCTACATGCTAGATATGGGAACAGGTGG
TCAACTATTGCTAGAAACTTGCCAGGGAGAACTGATAATGAGATCAAGAACTATTG
GAGGACTCATTTCAAGAAAAAAACCAAAAATTCAAGAGACAAATCTGAAAAATC
AAAGGTGCGTCTAGTGAAACGACAGCAATGGCAGCAACAAAAACAGCAGCAGAA
ACTGAACACTCAGACTGATAGTAATAGAGTCGTGTCACTCAACAACAAGAAGAAT
TATTACGCTTCAATCCTAAATAAGCTGGGGAATAAGATCGTGTCATTGCTTGATAAA
AATGAGAATAGAATATTACCACTTGTGACTCAAAAAGAAAACCAAGAAATGGCCA
ATTTATACTCAAATACAGCTGATCAAGAGGAAGATGACTTTCTTAATTCCATACTCA
ATGACTATACTTGTGTATCTTTGTTGGAGTCCTCGTTCAATGAAGATATGATGTGGG
ATGATTTTTGGAATAACTTGGATGAATATCAATGCAATGTCAGCACTACTATCTAG
>Peaxi162Scf01010g00010.1
ATGGAAGAGGCGGAGCAGCAGAGCGATTTTAAACTGATTGACGCCGCCGTCAATG

GCGGTGATACCATGGCGGTTGATGAAAACAACGGCGGCGGTGGTGGTGGAAA

AGTGAAGGGACCGTGGTCACAGGATGAAGACACAGTGTTGAGTGAACTGGTGAG

TAAGTTCGGGGCGAGGAATTGGAGTTTGATCGCCCGAGGAATTCCGGGTCGTTCT

GGAAAATCGTGTCGCCTTCGTTGGTGTAATCAGCTCGACCCTTCTGTTAAGCGCAA

GCCTTTTACCGAGGAAGAAGACAACATTATAATTCAAGCACATGCAGTGCATGGAA

ATAGATGGGCCTCGATTGCAAAGTTGCTTCCTGGTAGAACAGATAATGCAATTAAG

AACCACTGGAACTCTACATTGAGGCGCCGGTTTGCTGGGCTTAAAAGGGTCAACC

CTAGTCACTTTGAGATGTTAGATAGTAGCAACATTGACAGAGTTAAGGCGACATCT

GAAGAAACCAGGTCAGGTGATGATCATAATCAATCAAAATCCTTGGAGGGAAGGG

AAGCAAGCGTCCAGCAATGCAAACGTACCCTGCTTGAAGACAGAGCTCCAGTGA

GTGAGATGTTTGGCGTTCCTGAGAAAGTCGCACACCTTGTTTCTGGAAGCTGTCAT

TCCACTATTAATGAAAATAATCCATCTGTTTCTCGCCCAACTGCAAAAGTTGGCGC

ATTTGATGTGCATAACCTTTCTAGCGCTTCCTTTGCATCTTCAAGAACAGTGCCCAT

ACAGGGGAATCTACTCCAGGCCTTCTCACCTGACTTTGGTATCTGTAAACTTCTTG

AAGGTCTACCTGATGAACCTATAATACCTTCGAGCTGCGGGCATGGTTGTTGTTCA

GCTCACAGCAAGAGTTCCTCTGCTAGTTCTTTACTGGGGCCTGAGTTTGTCGAATA

TGAGGATCTACCCGCTGTCTCTAGCCATGAGTTATTTGCCATGGCCACAGATTTAAA

CAATATCGCTTGGATCAAAAGTGGACTTGAGAATGCTGGTAAATTACCTGACAAAA

CCACTAGCGGAAGAGATCATCAAGGTAGTTCTACTTCAACTCCAATGCCAATGCCA

ATGACAAGCTTTGTCTTGCCAGCTCAAGTTGAAAGCTTGAGCTGA

>Peaxi162Scf01068g00314.1

ATGAGACTATTACGTTTTTGTGTACTTGCTTCTTGTGATTTTCAAGAAATGAACTTT

TTAACTCAAAACTCTTTGGTTATGGACTATAACAAAGGGAGACACTGTTTGAGTAA

TGGTTCTACAGATGGTAAAGTACTAGAAAATCCTTGGATATTTCATTCAATGGTGGA

AGATGAGAACGGAACAAAGTTGAATGTTGAAACAGAGGAAGTAGATTTAGTAGG

GAGGAAAAATAATGGTGGTACAAAGCTATGTGCTAGAGGTCATTGGAGACCTCATG

AAGATGCAAAGCTTAAAGAACTTGTTGCTCAATATGGTCCACAAAATTGGAATCTT

ATTGCTGAAAAACTTGAAGGAAGATCAGGGAAAAGCTGCAGATTGAGATGGTTTA

ATCAACTAGATCCAAGGATTAATAGAAGAGCATTCTCAGAGGAAGAAGAGGAAAG

ACTATTAACAGCACACAAAATGTATGGCAACAAATGGGCAATGATTGCTAGGCTAT

TCCCTGGCAGGACAGATAATGCAGTCAAGAACCATTGGCACGTTATAATGGCTAGA

AAACACAGAGAACAAAACAGTGTCTATAGAAGAAGAAAGCCTTCAAACATGCAA

CAATTTCACTCTATGGGATTCCCTCATGTCTCTGGTTCTGATAATGCTATCCAAAGC

AATAATTTGAACAGTGATTCAACCATTTCTAGCACCACCAACAACATGGATGAGCA

CTGTGCTTCTACTTGCACTGATCTTTCACTCACTCCCTCTTCTTCTAAAGTCATTTTC

ATGAAAAGATTAACTCATATGCAGCATCACCACCACCCACTTCTTGAAGCAAGCAA

AGGCTTCTCAAGAGATGCACAAGAGGTGAAAATGGAAAGTGGGGTTCACCCAAA

ATTCTTGCATGGAAAGGAGCCAAGGGCAGACACAGTAGATGCAAGTCATCAATAT

GGATGTGGTGGGTCAGATACAAACTCAGATATTTCAGCATCTGAGTCAGTAGCCAA

CAACATGACCAATGTTAAGATGTATGGTCAAAATCATGAAAATGTGAAACTAGTAG

AAAAGCAGATGAAGAGCAAAACTATGCCTGCCTTCATTGATTTTCTTGGGGTTGGA

GCCACATAA

>Peaxi162Scf01101g00048.1

ATGGGAAGGTCTCCTTGTTGTGATGAAACTGGCCTTAAGAAAGGTCCATGGACTCC

TGAAGAAGATCAAAAACTCATCAATTATATCAAAAAACATGGCCATGGTAGCTGGA

GAGCCCTCCCTAAACTTGCAGGTCTTAATAGATGTGGAAAAAGTTGTAGATTGCGG

TGGACTAATTATCTTAGGCCTGATATCAAGAGAGGCAAATTTTCTCAAGAAGAAGA

ACAAACAATTCTCAATCTTCATGCTATTCTTGGCAACAAGTGGTCTGCAATTGCAA

CTCATCTACCGGGGAGAACTGACAATGAAATCAAGAATTTCTGGAACACTCATCTA

AAAAAGAAGTTGATTCAGATGGGGTATGATCCAATGACTCATCGACCTCGAACCGA

TATATTTAACAGCTTGCCTCATCTTATAGCTTTGGCAAATTTGAAGGAGTTAGTGGA

ACAATCAGAAGTGGCTAGGATCAAATATTTGCAATACCTTCTTCAACCTTCAATGG

CAGCTAATTTAGCAAACAGCATGAGTACTAGTAACACTTGTAGTGTGGCAAATATC

TCAGATATGGAAGCTTACAATAATCTTTTAAACATAGGCACGAACCATTTGGAAAAT

TCCACAAACAACATTCCACCACCTTCGACTACTCTTCAAGCTATTCAAGATTCAAT

CACTTTCTCACATTTGCCTGAATTACAAGCTACACCAAATTGTAACTTCCAAAATTC

TTTGAACGAAGAAAATATGGTTCAAACCCATGTTATTAGCCAAGGTGAAAATACAC

CTACTTCACCATGGCAGCTTCCTTCTTCTTTATCTCCACCAGAAAATGATAATCAGC

CTATTAATTCCTACATTAATAATATTGGGGGTAGTTGTAATCCTCCTTTCTTTGGAGG

AGCACATCCATCAGTTTGGCCTGAGATTCTCCTTGAGGACTCTTTGTTCCAGGATAT

TGCTTAG

>Peaxi162Scf01194g00013.1

ATGGATCATCAACATGCTAAAGTTGATTTGAGATCAAAAGAAGCAGCTTGTAGTAC

TAACAATATTATTCAAAAAGTTGAGGAAGATGATATGGACTTCAAGAGAGGTTCAT

GGACAGTTGAAGAAGATTTTACCCTTATTAATCACATCGCTCTCCATGGTGAAGGT

CGTTGGAATTCTCTCGCTCGTTCTTCAGGTTTGAAAAGAACGGGGAAAAGCTGTA

GGTTAAGATGGCTTAATTATCTTCGACCTGATGTTCGACGTGGAAATATTACTCTTG

AAGAACAACTCTTGATTCTTGAACTTCATTCTCGTTGGGGCAATAGGTGGTCGAAA

ATTGCCCAGCATTTGCCAGGAAGAACAGATAACGAGATTAAGAATTACTGGAGAA

CTCGAGTTCAAAAGCATGCCAAACAGCTCAAATGTGATGTCAATAGCAAACAATTC

AAAGATACACTGCGTTATCTATGGATGCCAAGGCTAGTTGAGAGAATTCAAGCTTC

TAATTCAAGCAAAAATCAAGTGATTCAACAAACAAGTAACAATAACATTAGTAACA

ATATTATTCCAAGCATGAGCTTCACACAGGAGAATTCTAGTACGACAACTTCATCA

GAAAATTCACTTGGGACACAAGTAATTTCACAAGTTTCAGACACGTCCGATTGTTG

CTACAATTACAGTACCATTAATCAAAGTGATACACTATGTTATGGAGAATCCTTGAC

TAGTCCAACAGGTTATTTTCATCAAGAAGGTGCACTTGACTTCAGAACAGTGGATA

TTGATGACAATCAACAAACCAACCAATTTTTGGATGTTTCAGACAATTTGTGGAAC

ATTGAAGACATGTGGTTCTTACAGGAGCAACTCAATTAA

>Peaxi162Scf01210g00002.1

ATGAATACTTCTGTTTTTACGTCGTCGGGGGTACTGAGGAAAGGAGCATGGGCTGA

AGAAGAAGATATTCTCTTAAGAAAATGTATTGAGAAGTACGGGGAAGGAAAGTGG

CATCAAGTTCCTGTTAGAGCTGGTTTAAATAGATGCAGGAAGAGCTGCAGGCTAAG

GTGGTTGAATTATCTGAGACCACATATAAAGAGAGGTGACTTTTGTCCGGAGGAAG

TGGACCTTATTCAGAGGCTTCATAAGCTTCTCGGCAACAGGTGGTCACTTATTGCC

GGGAGACTTCCGGGAAGAACGGCAAACGATGTGAAAAACTACTGGAATACTCAC

CTTCTAAGGAGGTCAAACTTTGCTTCTCCTCCCCAGCAACACGAAAGGAAATGTA

CTAAAGAAATTAGGACCATGGCCAAGAATGCCATAATAAGACCTCAACCTCGGAAT

CTCTCAAAATTAGCAAAGAATAACGTCTCAAACCACAGTACTATACACAAGGATGA

ATATAGCAAACAGAAAATGTTCATCGAGAAGCCAACAATGGCCGAAGTCGTGTCG

AGAGATAACAACGTTGAATGGTGGACGAATTTATTACTGGATAACTGCAACGGATT

TGAAAAGGCAGCACCTGAAAGCTCTTCAACATTTAAGAACATAGAAAGTTTGTTA
AACGAAGAACTATTATCAGCATCGATAAATGGTGGAACCAACTATCCCATGCAAGA
AACTGGAGACATGGGTTGGAGTGACTTTTGTATTGATTCTGACCCCTGGGAATTGC
TACTCCAATGA

>Peaxi162Scf01221g00042.1

ATGGGAAGGTCACCTTGTTGTGAGAAAGCACATACAAATAAAGGAGCATGGACTA
AAGAAGAAGATGAAAGACTTATAGCTTATATTAAAGCTCATGGTGAAGGTTGTTGG
AGGTCACTTCCTAAGGCTGCTGGGCTTTTGCGTTGTGGCAAAAGTTGCCGTCTCCG
TTGGATTAATTACTTAAGGCCTGACCTCAAACGTGGTAATTTTACTGAAGATGAAG
ATGAACTCATTATCAAACTCCACAGCCTCCTCGGTAACAAGTGGTCACTTATAGCT
GGAAGACTACCGGGAAGAACAGATAATGAGATAAAGAATTATTGGAATACTCATAT
ACGAAGGAAACTACTGAGTCGTGGTATTGATCCTTCTACACACAGGATCATGAACG
AGCCTAGCACACAAAAAGTCACAACAATTTCTTTTGCTGCTGGAAATGAAGATATT
AAAGATCAGAAGATCAGCATTAAAGCAGAATTTGAACAAATCAAGGACGATGAAA
TTATTAGCAAACCAATTAAAGAACAGTGTCCTGATTTGAATCTTGAGCTCAAAATT
AGCCCTCCTTACCAACAACATAGTGACAGGGCATTGCAACAGAGTACTACTGGTTC
AGGGGGGGCTAGTACTATATGTTTTACATGCAGTTTGGGTTTAAAAAACAATAAAG
GTTGCAGTTGTAGCAGGAATAGAAGTATGAATGTTGCTGGTTATGACTTTCTAGGAT
TAAAGACTAACGGTTTGGACTACAGAACATTGGAAACTAGGACTAAGTGA

>Peaxi162Scf01251g00013.1

ATGGGTAGAACTCCATGCTGTGAGAAACTAGGGTTGAATAGAGGTCCATGGAGTA
AGAAAGAAGATGATATACTCATAAATTACATTGATAAACACGGCCACCCTAACTGG
CGTCAACTTCCCAAACTTGCAGCATTCACGTGGTCTGTAATAGCAGCAAAATTACC
AGGACGAACAGACAACGAAATAAAAAACATTTGGCATACTCGTCTGAAGAAGAA
AATGAATCAGTCTCAGATTCAAGAGAAAACACCAGACATACGAGAACAACCAAGT
GAAACATCAAAATCCGAGGATTCAACAAATATACAAGAAAATACTGAAATATCAAG
TCCTAAACCAAATTCCAATAATCAACACGAACCAAGTTCATCGTTACGTTCATCGT
CTTCAATAACATCATCAAGTGAAGGTTCATGTTCAAACACAACCACAACGAGTTCA
CATATTGACGAGTCTCGAGACCAAATGAACTTGGAGAATTTGCTTGAAGTTGACGA
CAATTTTTGGTCAGAAGTACTATGGGCAACACCTGCAGATGATTCTAAGGACAATA

ATCTTGATTTTTCATTATCTTCAGTGGAGAAAAATTACGAGCTTGACTCCAGCTTGA
ATGATAACTGGTTATGGGATGATCTCTTTTCAAGAGCCAACGAGTTTTTGCTAGAAT
TCCCTGAATTAGTATGA
>Peaxi162Scf01256g00017.1
ATGGGAAGGCCACCTTGTTGTGATAAAATTGGTGTCAAAAAAGGACCATGGACAC
CAGAAGAGGACATCATTTTAGTTTCATACATTCAAGAACATGGTCCTGGTAACTGG
AGAGCTGTTCCTACTAATACTGGATTGCTTAGATGCAGTAAGAGTTGTAGACTTAG
ATGGACTAACTATCTCCGTCCAGGCATCAAACGTGGCAACTTTACTGAACATGAAG
AGAAGATGATTATTCACCTCCAAGCTCTTCTTGGCAACCGATGGGCAGCTATTGCT
TCATATCTTCCACAAAGAACAGACAATGACATAAAAAATTATTGGAATACTCATTTG
CAAAAGAAGCTTAAGAAGCTTCAAGGTCATGATGATCAGAATAATCAAAAGGGAA
AATCTCAATCAATCTCAAAGGGACAATGGGAGAGAAGACTTCAAACAGACATCCA
CACAGCCAAACAAGCTCTTGTTGATGCTTTGTCCCTTGACAAAAAAACTACTAATG
TTGTTTCACCAAATATTAATTCTAGTGCTAATTCTCTTCCTGTTGGTCAAACATCTTC
CTATGCATCTAGTGCTGAGAACATATCTCGATTGCTTCAAAATTGGATGAAAAACTC
TCCTAAATCATCTCAAACCACATCTAATTCCACCACCAGTCAAAGTTCTTTTAACAA
TTTATCAATGGTTTCGAGTTCGAGTCCTAGTGAAGTGACCATGAGTGCAACAACAC
CAGAGGGATTAGACTCACTCTTTAGCTTCAATTCATCCTATAATAATTCTGATGTTTC
ACAATCCATGTTGACCGACGAGGTTGTCGCTGGAATTTTCCAAGATGGAAGCAAA
CAAAACTGGCAAAATTTCAAGGATGAAAGTGGAATTTTCCAAAAGGGAAGCAAA
CAAAATATGGAGACACAAGTTCCACTAACTTTGCTTGAAAATTGGTTGTTTGATGA
TGCTAATGCACAAACGCAAGAAGAGCTAATGGGAATTGGAGTAGGCATGGAATGG
CCTTGA
>Peaxi162Scf01302g00033.1
ATGGAAATTCATTCTCTTGAAATTCTAGAAACTTCCAATCCCGTAGAGGGTGGCAG
TTACGGCGGCGACAGCGGCGGTGAAGATGAGATGTTAGAGAATGAAAGTGGAAG
CAGTGAAAATGTTGTAAAAAAGAATAAAAGAACAAGTGGAGAAAGAGTAAAAGG
ACCATGGTCACCTGAAGAAGATGCAATACTAAGTAAATTAGTTAGTAATTTTGGAG
CAAGAAATTGGAGTTTAATTGCCCGTGGTATTCCAGGTAGATCCGGTAAATCTTGTC
GTTTAAGATGGTGTAATCAGCTTGATCCTTCTGTTAAACGCAAACCCTTTTCAGATG

AAGAGGACCGACTTATTCTTCAGGCCCATGCTATTCATGGGAACAAATGGGCATCA
ATTGCAAGGCTTTTGCCAGGTAGAACAGATAATGCAATTAAAAATCATTGGAATTC
AACCTTGAGGCGCCGGCACAACGGTAAGTTAAGAAGTGACCCCAGCAATGTTCTG
GAGGATGTTAGTGCCGAAAAATCAAAAGCATCGTCAGAGGAAACTCAATCATGTG
GAGATGTTAATTCTTTGAAGGCTATTGAAGGAAAAGATGTCAGCTCTCAGGAAAAT
CAAGAAGATAATCACCATGAAGATAAGAGTGAAGCAGAAGTTCAACGGAGTGATG
CAGCAAATGATCCTCCTACTCTTGTCCGTCCTGTGGCTCGTATAAGTGCTTTTAGCG
TGTATGGTTCTTTGGATGTCCCAGAAGCCCAAATACAAACTCCAAGGCTAACTCCT
GTACAAGCACCGGATCTTGGGATAAGCAAATTGCTTGAAGGATGTTTCACTGACAG
ATTAGTCCCACACCAATGCGGCCATGGCTGTTGTGGCAATGCCAGTCAGGAAAAC
AACGGAAGTTCACTGCTGGGACCTGAGTTTGTTGACTATGCTGAGCCTCCATCCTT
CTCAAGCCATGAGTTGGCCGCATTAGCCACAGAGATAAGCAATGTTGCTTGGTGTA
AAAGTGGGTTGGAGAACAGCAACATTGAGGTTATCTGCAATCCAACAACCAGGGC
AACATCTGGTACCTCTCTCTTGCCAAACAGACCATTCTAA

>Peaxi162Scf01314g00012.1

ATGGGGAGAGCTCCTTGTTGTGACAGAAGTAGTGTCAAGAAAGGGCCATGGTCAC
CTGAAGAGGATGCTAAGTTGAAGGCATATATTGAGCAGAATGGTACTGGTGGGAAT
TGGATTGCTTTGCCTCAAAAAATCGGGCTTAAGAGATGTGGAAAGAGTTGTAGGTT
AAGATGGTTAAATTATTTGCGACCAAACGTCAAGCATGGAGGGTTCTCAGAAGAA
GAAGATAGGATCATTTGCAGCCTCTACATCATTTTGCAGCCAATACCTTATTCAGAT
TATGAACCGCGATTGAATGGCGCTTCAATCAGAAAACTATTAATAAAGCTTGGAGG
TAAATTTTCAGAAGATGATCAACCGATAAGTGGAGAACAAGATCCTCAATATCCTAT
GAATAATTCAATTATTTCGATGCATTCGATATATGAGAATCCCATCAATTTGATATCTT
CTCCATTAGATGTCTTAAATGCTCAATACAACATGAATCATGGGACAGCTTTGACTA
CAAGTCCATTACAAGGCAATTTTACAGCTGAACGTATGATTTGCAATATTAATTCAC
AAAAATTGGGGGATCTTGAATTTTTATATGA

>Peaxi162Scf01390g00030.1

ATGTGCACAAGTGGCATCACAGGTTTGCATAAGGGTGCTTTGATAACTCTTGGCAG
CATTGTAGCCATTGCAGCTGGTTTTATGACTGTGATTACAAAACTTGAAAATAGTGA
TGTCTGTTTGGCTTACCTCCCTTTTGCTCATGTTTTTGAGCTGGCTACTGAAACTGT

AATTCTAACTGTACGCATTCAGGGTTACGTTTCGGATGGTACTGTTAAGAAGGTTG
AGACGAAGGGAGTTTCTGCCAAGAATCGAATAGAAACCAAATTTGGTGCCGTTCT
AGAAGACACAACTGATATAACCGAGAGAAGCATGGTAATAGGAGATGAGAAGGTA
GGTATACCCCGGAAGGAGAAGAAGAAATCCAAGAAAAACAATAAGAATGCTGATA
TCAGTGGTCTAGGAATGACAATGGCTGATGCTGCAGAAACCAGGAAAGATAGTGT
TAAGGCAGAAAGTGAAACTAAGCCAACAGGAGGTAGTGTTAATCATGACATCAAG
AAAAAGAAAAAGAAAAAGAGAGAGGATGGAACACATCCTGATAAGTCCAATAAA
GACAGTGATGAAAGTGCTGCAAAATGCGTTGAAAAAAAGAAACACAAAAAGTTG
AAGAGAAGCCACCAGGATGAGCAGCAGATAATTCTTGACGCTCCTATAAACACAA
AGAGCGTCGCTTATGCAAGTGCAAGGGAAAGGGGGGACAGTGAGAACGTTATTA
AGATACTCGGTGAGGTCTCTGGTGGGAACGCTGTGGAGGAGATTAGGAGGAAAA
AGAAATCCAAGAAAAGCAATAAACATGCTGTTGAGGGTGATTTAAGTTCAGAGGA
AAAGAAGCTGTGTGTTGTGAAGGGAAAGAAAGAAAGGGATAAGGTCAATATGGA
AACTAAAGCAGCAGGAGAGAGTGACGTTTGTGATGTCAAGAAAAACAAAGAAAG
AGAAGGCGAGAAACATCCTGGCAAGTCCACTAAAGACAGTGACAAGGATGATTTG
GGAAGTGTTGAAAAAAAGAAAAGTAAAAAGTTGAAGAAAAGCTGCCGCGATGAG
TCTGAGACAATGCATGGTATTGAGGACGAACAGGAAGCTATTGTGGCTAAAGTCA
ATCGAGGTGATATTTCTTCAACTGTGGAAGAGATAGAAGAACAGAGCAGAACGGA
TACTGGTAAAATCAGAAAGAAGAAAAAGGCAACAATAGGACACAGTTCTGAAGA
TCCTACACATGAAAAGAGTGAAAAGAAAGTGAGATTCTCTGGTCATGTTGAGATTT
TTCCTTCATCCAGTGATCCAAGTGATGAGAAGCATGAAACTGAGGAAGAAAATTTA
CTGCGTGGCAAACGATTCTCAACATTAGAAGATGAAATTGTCAAAGAAGCTGTTC
ATAAATACATAGAGATGCATAACTTGGGCGAAGAAGGGCTGGAAATGATTTTAAAT
TCTAGATCTCATCCTGAATTAAAGGGTTGCTGGAAAGAAATAGGGAGCGCTATACC
GTACAGGCCTTATACTGCGATTTATTATCGTGCACAGGTCTTGTTTCGAAGAAGTGA
GAAGCGTAAATGGACTGAAGAAGAGTATGAGATGGTACGAAAATACCATGAAGTA
CATGGGAACAAGTGGAAGGACTTGGCTGATGAACTTGGAAAACATCGGTTTCACG
TGAAGGATACATGGCGAAGACTAAAATTGACCAATCGGAACAAAGGGCAATGGAC
TCAGGAGGAGTACCAGACTTTGTTTGATTTAGTAAACACTGATCTGAGACTGAAGC
TCTCTGAAGAGAAGAAATCTAAGCATGGTATGTTACGGGATAATATTGCCTGGACT

GCAATAAGTGGCAACTTGTCCACAAGAACTGATGCAAATTGTTGCTTGAAATGGTA
TGACCAATTAACATCACCCATGGTATCCAAAGGTGAGTGGGCGGATACTGATGACT
ATCGCCTTATTGATGCATTATTTGAGCTAGATGCCAGCTGCATAGAGGACGTAGATT
GGGACAATCTTGTTGACCACAGGCCTGGAGAGATATGCCGAAAGAGATGGAACCA
AATGGTTCTTCACATAGGTCAATATGTAAACAAGTCATTTTCTGAACAAGTAGAAG
TTCTAGCTAAGAGATACCGTCCGGAGTTGGTTGAAGTAAGAGAGGCTTGGGATAG
TAAACCAATTGTATACTATTGCCGAAGCAAAGCTGGAAATGCTAGTGGAGGATCAG
ACATTATTGAAGAGTTAACTGTTGATCAAGAGATCAATCAAACTGCAACTGAAACT
CGAGATGAAGGTTTTGACAGTGTGTTGTTAACAAAACATAAACAGAAAAATGTCA
CAGAATCAACCAAGCTATGGCCATCAGCAGCTATCTCATCGAGTGGCAAGGACAA
CAACTGCAGTCACAGTGAGTGGTTGACTCATAGTGCTTTGCGGATTAACGCTGGTG
GCCACCGTTATCTGCCTAGTTGTAGCCACCCCTTTGTTGCTGATGTTCAGCCCTCTT
CTTGTCCAGCCATGATATCCATTTTCTTGATCCTTGCAGGGTTCTTGGCTTCTGGAG
GTTTTGGTGCTACTGCTACTTTTATCCTAATCAAGCCCTCGTTTGCAAGGTTGGTGA
AGAACTAA
>Peaxi162Scf01464g00024.1
ATGGGGTGCAAGATAGTTGAAAAGACAAAGCAAAAACACAAGAAGGGATTATGG
TCCCCTGATGAAGATGACAAACTCAGAAACTATATCATCAATCATGGTCATGGCTG
CTGGAGCTCTGTCCCCATTAATGCTGGCTTGCAAAGAAATGGGAAAAGTTGCAGAT
TGAGGTGGATTAATTACTTAAGGCCTGGGCTAAAGCGAGGGGCATTTAGCTTAGAA
GAGGAAGACACAATCTTGACCCTTCATGCCATGTTTGGCAACAAGTGGTCTCAAAT
TGCACAGCACTTGCCTGGAAGGACAGATAATGAGATAAAGAACCACTGGCACTCT
TATCTAAAGAAGAGAGTGGCCAAGAATGCAGAAAATGAAAGCCAAACTCAATCTG
GAAATCGCGAATCATTGCTATTTTCTAAGAAGTGGACGCCCCAAAATTCTAGTTTG
GATTCATTTGAACACATAGAAGGATCATTAGCAGATTCAGATCAATCTGGTTCGCA
GGTGGACTTTCCAAAAGAGCATCAAAAAAGTAATTTGCCCAAAGTATTATTTGCTG
AGTGGCTTTGTTTAGATAAATTTCATGGTCAAGATTTCCAGAACTCAGGGACTTTT
GATCTTACCAAGAATAACTTTGGGTATAATAACTCGGAGTCGCAACACGCCTTCAT
GCATGGTTTACTGATGAATGAAGGTAGCTATGGGAGTTGCATGAATCAAGAAGTTA
ACAATGGAACAGTAGATGATATGTTTCAACCCCAACTCAAGTTTGAGGATTCAATG

TCTGCAAATGGATTTGAGGACTTTATGTCGGGGGAATTCATAAACACTGATGTTATG
TACATATGA
>Peaxi162Scf01819g00004.1
ATGGAAGGTGGTAGTGGAGGAGATGGAAGTGCTTATCAACCATACCAGAATATACC
AACTAACCTTTCTATTTTTCAACAAGGTCCTCCTTTAATAGCCATTAATAGGTACTTA
TTGAGTCATCAAAGCCAATTTTCTTCGCAACAAAGTGTAAACAACCAGAGCATTTT
CTCTCAATGTGGTGGTTTATGTGATTTTTCGTCTTATAGTGGTATTAGTCATGCAAAT
GGGATTTCTTGGTCAAGTGTGCCCGAGCCAAGCTTCGTAGATAGAATTTTTCTCAG
TCACGAACAAAATGGTCTCCATTGGAATAATCAAGAGAATGGCATAGCAGAAGAG
ATGATATCATCTCAGCAGAATTCAAAGGAGGCCGGAAAAAAAGCGAAGGAAGGG
CCTTCCTCTACTACTGTACTGGTCAAGGGTCAGTGGACGGAGGAAGAAGACAGGA
AATTAGTGAAGCTGGTAAAGCAGTTTGGAATGAGGAGATGGGCTCAGATAGCTGA
GAATATGGTCGGTAGAGCAGGAAAGCAATGTCGCGAGAGATGGCATAACCATTTG
CGTCCAGATATCAAGAAAGATGCATGGAGTGAACAAGAAGAACTAATCTTAGTGG
AAGCTCACCAGCAAATTGGAAACAAATGGGCAGAGATTGCTAAGAAAATTCCTGG
AAGAACTGAAAATTCAATCAAGAATCACTGGAATGCCACTAAAAGAAGGCAACAT
TCGAGGAGAAACAAATTTAAGAAACAGGAAAAAGATGGCCAAAATGATTCCAAG
TATCGATCTAACATTCTTCGAGATTATATAAGGAGCAAGTACTTCAGTGACAATTCT
CCTCCCACTGCCAGTACTCCAAGTAATAGTATCAGCACCAACGCAACCCCACCTTA
TTCCGATGATGACTCCCCATCTTTGCTCACTCGTCAAACATATGATGAAGAAATGAA
CTTCATGCAAAATTTATTTGGGGAGAACTCATTGGTGGAAAATAATGGTAAAGTTG
CGGAGGCAATGGAAGCAAAAACAAGGCAATGTCCTTTTGACAATAAGTCATCATC
TAGCTCATGCCCTTTCAACTCCTTGGTAGCGGGATATACAAAGAGTGATAATCAGAT
AGTTAACGACAACAGTGAATATGGCTATAACATGTACATGGCTGACCATTATTCTAA
AGTGACTCAGCAGTCTGATTCAGACCAGTGTTCTGGAATTTATCTTTCCTACCTCCT
TGATCATTCTACATCAGCTAATTCTTTGCCTTGTTCTGGGAGCATAGGATATGGAAA
CATGAATGCGGGCACTCTGGTGAATCAAGGTTCCTCCTCATCGAAAGGAAACAAA
GAAGTTGATTTGATGGAGATCGTTTCATCTACTCTCTACCGTCAACAAACGTCTCAT
ACTACTTTTTTTAATTAA
>Peaxi162Scf01969g00021.1

ATGAGGTGCAAGCCAGTAGAAAGTCCAAAGAAAAAACACAGGAAGGGACTGTGG
TCTCCAGATGAAGACCACAAGCTCAAAAACTACATTCTTGAGCATGGCCATGGCTG
CTGGAGCTCTGTCCCCATAAATGCTGGCTTGCAAAGGAATGGGAAGAGTTGCAGA
TTGAGGTGGATTAATTATTTAAGGCCTGGCCTAAAACGAGGGGCATTTAGCATAGA
AGAGGAGGAGACAATTATGACCCTTCATGGCATGATAGGCAACAAGTGGTCTCAG
ATGGCACAACATTTGCCTGGACGGACAGATAATGAAATTAAGAACCATTGGCACTC
TTATTTAAAGAAGAGAGTTGCCAAAATTGCAGAAAATGAAGCCAAGAGCCAAAAT
ATGGATGGACTATCTTCATCTTCCTTGAAATTGACTTCGCGAAATTCTAGTTTGGAT
TCGTTTGCACAAATAGAAGGATCATTAACAGATACAGATCAATCTATTTCACAAATA
GAATTCTTAAAAGAACCTCGCGAAAGTAATTTTCAGAAAGTATTATTCGCTGAGTG
GTTTACGTTAGATCAATTTAAAAGCCAAGATTTTCAGAATTCAGGCAATTCAAATC
ATTCCAGGAATAATAACTTTGGCTACAATAAATCAGATTTCCAAGATGGTTTCATTC
ATGGCTTATTTATGAGTGAAGGTACTTATGGGGCTGACATAAATATTCCAGGGGTAC
TCAACAATGAAACAGTAGATGATATGTTTCAAGCATCACAATTCAAGTTTGAAGAT
CATATGTCTGCAAATGGATTTGAAGAATTTATATCTGATGAATTCAACATTACCGAA
GATGTGATGTACATATGA
>Peaxi162Scf03779g00019.1
ATGAGAAAGGCTTGCTGTGATAACAAGGAGGAAATGCATAGAGGAGCTTGGTCTA
AGCAAGAAGACCAGAAGCTCATTGATTATATCACTAAGCATGGCGCAGGTTGCTGG
CGAAATTTACCTAAAGCTGCCGGTCTGCTTCGTTGCGGTAAAAGTTGCAGGCTAAG
ATGGATGAATTATCTTAGCCCAAACCTTAAAAGAGGAAATTTTTCTGAGGATGAAG
AGGATCTCATCATCAAGCTTCATGCCCTACTTGGCAACAGGTGGTCTCTAATAGCG
GGTAGATTGCCAGGGCGCACTGATAATGAAGTGAAGAACTATTGGAATTCCCATTT
GAGAAGAAAACTTATAAAAATGGGAATTGATCCAAAGAATCATAGGATATCTCATT
ATCTTCATAGGAAAAGACTTGAGTATTGGTCAGAAAATAGCAGCAGAGGAACCGA
TCATGAAGTGGTCTCTGATGCTGGAAGTTCTTGTGCAAAACATCAACCAAGTTCCT
TGCCTGATCTCAATTCCCCTCCTTCAATTCACAGTTCTTGTGCACAACCGTAG
>Peaxi162Scf03974g00003.1
ATGGGAAGAATCCCATGTTGCGAAAAAGAGAACGTCAAGAGGGGGCAATGGACT
CCTGAGGAAGACCACAAACTTTCATCTTACATTGCACAACATGGAACTCGTAACTG

GCGTCTCATTCCTAAGCATGCTGGTCTACAAAGATGTGGAAAGAGCTGCAGATTAC
GATGGACAAATTATCTCCGGCCTGATCTCAAACATGGCCAGTTTTCAGAGGCAGAA
GAGCAGACCATTGTCACACTTCACTCCGTTCTTGGCAATCGATGGTCTGTTATTGCT
GCTCAGCTGCCGGGCCGTACTGACAATGATGTTAAGAACCATTGGAACACCAAGC
TCAAAAAGAAGCTATCTGGGATGGGAATTGACCCAGTAACGCACAAGCCATTCTC
TCACCTGATAAGTGAGATTGCTACCAATTTGGCTCCACCACAAGTTCCTCATCTAG
CAGAAGCAGCCCTCGGATGCTTCAAGGATGAAATGCTACATCTTCTCACAAAAAA
ACGCATCGGCTTCCAGTTTCAGCAATTTGGAACCAGTACGGCCCCAAGTACCAGTA
CTGTCAAAGTTGAGGATAATAAAGAAGAAACAATTGAGAAAATCAAGTATGGATT
ATCAAGGGCTATTAAGGAATCTGACATGTTACCCTCTAACAAGCATTGGGACCCAA
GTGGTGGAGCAAGATCTACTAACCTTGCTGAACCCAGCAGTGGTTTTCCCCATGTA
TCTGATGGAGGATTTCAGTACAACTTTGCTTCTTTGCTACATGAAGATGCGGCGGA
AGGGTCACCCTGGAACCAAAGTTTGTGTACTGGAAGTACGTGCACAGTAGGTGAA
CAACAGCAAGTTCATCAACTACATAAGAAACTTAACAGTAATGACAACTGCGGCG
AGGATAATTCCGACGGTGCGAAGGAAACAAGAAATGGATCAACAACCATGTTCCA
TTCAGACTGCATTCTGTGGGACATATCATCTGATGATCTCTTGAATCCAATGGTTTG
A

>Peaxi162Scf25743g00028.1

ATGCACTTTCTGAACCGGATGCAAGCTGCATCGAGGATGTGGATTGGGACAATCTT
CTTAATCACAGGGCTGGAGAGTTATGTCGGAAACGAAATGGTTCGTCAGCTAAGT
GGTGCTTCAACCATAGAAGAGATAGAAGATAGCATAACAGATAATGGTAATATCAG
AAAAAGGAAAAAGACAAAATTAAGACACAATTATGAGGATCTTCCACATCAAAAG
AGTGACGATAGAGTGAGATTTTCTTGTAATGTTTACCCTCCATCCCGTGACACAATC
GATGAGAAGCATGAAATTGAGGAAGAAAGTAATGTGGATATTGTTACTGGAGTGA
ATCGAGGTGTGGTTTCAACCATAGAAGAGATAGAAGATAGCAAAATAGATGATGCT
ACTATCAGAAAGAGGAAAAAGACAAAATTAGGACACAATTCCGAGGATCTTACGC
TTGAAAAGGGTGAAAAAAGAGTGCGGTTTTCAGGTGATGTACAGGTTTTCCCTCC
ATTGAGTTATTCAACTGATGAGAATCATGAAGTAAAGGATGGAAGTTTACTGCGCG
GCAAACGATTCACAAGTGAAGAAGATGAAATTATCAATGACGCTGTCTATAGATAC
ATAGAGGTACATAACTTGGTAAGGACTTGCTGGAAAACAATTGGGAGGGCTATACC

GTACAGGCCTTATAGAGCAGTCTATAATCATGCACAACTCTTTTTTCATCGGGGTGA
GAAGCATAAATGGACTGAAGAAGAGTTTGAGATGATACAGAAGTTCCATGGAGAA
CATGGGCCCAAGTGGAGGGTCTTGGGTGATAAACTTGGGAAATATTGGGTTCATGT
GGGAAATGCATGGCAAAAGATAAAACTGGCCAATCGGAACAAAGGAAGTTGGAC
TCAGGAGGAGACCCACCCAGACTTTATATCTAAGCATGGGATGTTACGGGGTAATA
TTTGCTGGAGTGCAATTAGTGACAGATTGTCCACCAGGATTAAACAACACTGCTGC
AATAAATGGTACAAACAGTTAACATCTCCGATGGTGCCCACAGGTGAATGGGCAG
ATACTGATGATTACGCTTAA

Part II. The protein sequences of PaMYB gene family.

>Peaxi162Scf00001g00231.1

MGRAPCCEKVGLKRGRWTAEEDETLTNYILTNGEGSWRSLPKNAGLLRCGKSCRLR

WINYLRSDLKRGNITSEEENIIIKLHATLGNRWSLIAGHLPGRTDNEIKNYWNSHLSRK

VESLRIPSDEKLPQAVVDLARKGALKQIKRKVGRTSRSTMMRNRKSKNSAVSSLSMP

KQLKESSEPLNATVPIMPSTPNLEKEALSRTSTISSWLDGNNAMDSMQKEVANIAAPN

PLMESREAHSSLSSDGMEWLEEIMPLVIDDQDMDPNFIFTCLENGQGESAVKVTEEAD

NNFLNTSKINERDNNNRESSEAAVSINIHDEQVHEKSNETSSLMKDEGTFDQWDWKE

ITHDGGKGWPWDDNNSLQLWDTTTDDTGFFQNCINEATVEMDSVHFENQKHSALVA

WLLS*

>Peaxi162Scf00001g00599.1

MEGRRPSSSYHHYRYHFHHEDELGEIKKGPWKAEEGEVLLNHVKKYGPRDWSSIRS

KGLLQRTGKSCRLYWVNKLRPNLKNGVKFSAEEERTVIELQAQFGNKWARIATHLHG

RTDNDAPKFSSSADQEEFLSKSQSCSSSYIDNSNMIYLVPLNPNSTDFEANLLQLDFTA

NEKKLKIDSHIQLPFTKLQNDFALPLQTHEFPMPNFIDVFGQQLNGSELDNVQVPFVS

TCSGPDRSCFENPSSPDSFIDDFPLDMFDHIEPLPSQSE

>Peaxi162Scf00002g00037.1

MGRQPCCDKLGVKKGPWTAEEDKKLISFILTNGQCCWRAVPKLAGLRRCGKSCRLR

WTNYLRPDLKRGLLSDAEEKLVIDLHSRLGNRWSKIAARLPGRTDNEIKNHWNTHIK

KKLLKMGIDPVTHEPLKKEANLSDQPNTESDQNKENGHQQVQVVPQSTNVTAAAAT

STEFDNNSSFSSSASSSENSSCTTNESKLIFDNLSENDPLLSCLLEADTPLIDSPWEFPMS

STTTAEEPKSFDSIISNMTSWEDTFNWLSGCEEFGINDFGFDNCFNHVELDIFKTIDNV

ENRHG*

>Peaxi162Scf00004g02328.1

MRKPEFSSSGKNGTNSNNNINNANMKLRKGLWSPEEDEKLMHYMLTNGQGCWSDV

ARNAGLQRCGKSCRLRWINYLRPDLKRGAFSPQEEELIIHLHSLLGNRWSQIAARMPG

RTDNEIKNFWNSTLKKRLKNSSSCSTPSPNASDSSSEPCKDNLNMGINQGLIMSMQNH

NLMSMFMDSTSSSSSSMALNTIIEPLPMLEQTLINMPNGLSAPPYLTTQPCMTQGRSIV

TNGSLFYGNNHGIFGGNLGMEGELYIPPLESVSIHEYQNVENGNLVERSSQNNNNPTN

SMTNLTSHFNSSSNNIKVENFGGVGNYWEGDELKVGEWDLEELMKDVSPFPFLDFQ

VE*

>Peaxi162Scf00006g00098.1

MARTRCYDKSGLKKGTWTPEEDRKLAAYVTRYGCWNWRQLPKFAGLARCGKSCRL

RWLNYLQPNIKRGNYTKEEDQIIMMLHAEIGNKWSAIAAHLPGRSDSEIKNHWHTSL

KKRSTQEESTSSTNSKKRSPINSKYISSSRKKRSGSGNQISVKSNVPQLSPKQSCSEVSS

CASIHVESMHVESEGYQEEYLFEESSGISFWTEPFLVDNSSTTNDFFVPSELDHGLVSP

FSPVISFDEFICSYD*

>Peaxi162Scf00007g00207.1

MVRTPCCDENGRKKGTWTPEEDRKLAAYVTKYGCWNWRQLPKYAGENFCTIYTNI

APREGLARCGKSCRLRWMNYLRPNVKRGNYTKEEDEIILKLHAELGNRWSAIAAQLP

GRSDNEIKNHWHTSLKKRANYGPNSSESSKKCNKNSESKRKRVENQNASHETILESS

HMSPKQSIGEELCSNTTDYKQQDVASVIREEELYYEEALAEISGNFWTEPFLLDSFSNR

FDFRAPSIDCGLVCPPSPFIGHELLSSFDFDDSNW*

>Peaxi162Scf00007g00823.1

MAPDDRGMKNGGASTGRSNGAGSSRQVLKKGPWTAAEDSILMEYVKKHGEGNWN

AVQRNSGLMRCGKSCRLRWANHLRPNLKKGAFTVEEERIIIELHAKLGNKWARMAA

QLPGRTDNEIKNYWNTRLKRRQRAGLPIYPQELQQQNQHENNNQPHSLLSSSYDPQN

STNYNSPSLSLLDIFNPSTMKPSITQQFPINTPSLCLPSTNNNNIFRNTPKGLSLTLPSSM

RNSQFSSLPNNNFTQGLSSNSIQVPPFQHNYPNFNINRPFTGISSNPNGLICGMGINTIN

YPSGQSSMPVTASSSENTGSDFGSSDNANNYANTNGLSRGNSGLLEDLLEESQTLNRP

GMKIEDNFLDLKEDQEADYKGKSMLWEDYGLVEDAEEAILTEESAYSFAHGVDHVA

QNKNSESSSPHSPPNSSSGIFMKKEDSFHGTNQADDDIMCLLDNFPLAVPVPEWYEDE

DDKNNCNGQSSNVTNCDHIAENQAEDSKSPALTLNSGTRNHDWEFGGCCWNNMPSF

C*

>Peaxi162Scf00007g02122.1

MVRTPCRDEYGRKKGTWTPEEDRKLAAYVTKNGCLNWRQLPKHAGLARCGKSCRL

RWMNYLRPNVKRGNYTNEEDGIILKLHTQLGNRWSAIAAHLPGRSDNDIKNHWHTS

LKKRANYESSKKYNKNRESTNTKNQASSRRKSTVENENASSLSCSMSTHETILEGSQ

WSLKQSSSEEISSCTADYQQLDIASDIREVKVFEEEAYVAESCGSFWTEPFVVDSFSSR

LHDCVAPSIDFELVSPPSPFIGHGFISSFNLDDYLMLSSLFD*

>Peaxi162Scf00007g02125.1

MVRTPSIDKNGMKRGAWSEEEDNKLRAYVERFGHPNWRKLPRYAGLMRCGKSCRL

RWMNNLRPGLKKGNYSHEEEQLIIKLHNQLGNRWSTIAAKLPGRSDNDIKNHWHAH

LKKRTKTNANSSTMEQMSTESSQSGCQAEQSNSKLPELENYCDPIDTSSPPEVSSSDLS

KLFSSCSLLNGMDWIEDDQIRSMEQLSNIDSVEPLLDSFSCWTKPIDNFQTEHCFDNV

WSEHFDNFWTQPFL*

>Peaxi162Scf00007g02128.1

MVRTPSVDKNGVKRGAWTEEEDNKLRAYVERFGHWNWRQLPKYAGLMRCGKSCR

LRWMNYLRPGLKKGNYSNEEGQLIIKLHNELGNRWSAIAEKLPGRSDNDIKNQWHS

HLKKRAKTNSYSSTLMEQMSTESSQSGSQSEQSCKLSELEACDKKEVTSAAIDRLDS

VPPVSLEVSSSSLLNGIMDYWMEEEDRMFRPSMELLSIMVRTPSIDKSGMKRGSWSE

EEDDKLRSYVERYGHPNWRQLPRYAGLMRCGKSCRLRWMNYLRPGLKKGKYSHEE

EQLIIKLHNQLGNRWSTIAEKLPGRSDNDIKNHWHAHLKKRSKSNTNSSAIMMKQLIE

CSQSESQDDEHSNSKFAEHDSFSPIDSVQPVSSDVSSSSVLNGMDCWFEEDNHIFSSM

EPLPDFFNFSWTKPIYNFQTEPFDHYWTEAMDNFWTEPFF*

>Peaxi162Scf00007g02414.1

MGRSPCCDKNGLKKGPWTPEEDLKLIQYIQVHGPGNWRTLPKNAGLQRCGKSCRLR

WTNYLRPDIKRGRFSFDEEETIIQLHSVLGNKWSAIAARLPGRTDNEIKNYWNTHIRK

RLLRNGIDPVTHSPRLDLLDLSSLLNSTQFNLSSLLGLQALVNPEVFRLATTLLASHNN

ENPELLLQKLQENQLLNTQLQQNQLLNTQLGNHLQVFQPNSQFQNQIQEIPTFTPSNV

PCSSSQPMQLGPVELSYLMNGQMLPPQYGYCASNVSDSSNLQSLNNSNNQNSSNFSL

DSVLSTPLSSTEDEKESYCSNLMKFEIPASLNFDDFM*

>Peaxi162Scf00008g02024.1

MGRAPCCDKKGLKKGPWAPEEDEKLIEYIKNNGHNGWRSLPKLAGLLRCGKSCRLR

WTNYLRPDIKRGPFSEEEEKLIIQLHGILGNRWAAIAAQLPGRTDNEIKNLWNTHLKK

RLFSMGIDPHIYEPSSAPSGLVRRPPASSSTRHMAQWESARLQAEARLSKDSHLSIPSL

VGKSQSDYILRIWNSEIGEAFRNFKGVEKTTSESSSPKCGSTSSITTEMDITLTGSSVEG

TNQHKDAEWKNCKLYTGNILRGSDTSSSNDFEESSESALQLLLDVPSKYDLSFLGQSD

AYSMYPAFLSESCLNCSSTEHEVCYV*

>Peaxi162Scf00009g00022.1

MEFDHSSNQNLSLQPYNNSLIRGEMDDVFCTVSKDYLQDFHHLDLTFNNPHDNLLIE
TNGYDSLFDPILNEGNLSSLDHQDFNLNYEFKPFEQNSASGSTLVMKNFENSMDMHT
SNDENLLSLCSEDMKPLSFVNVQDESSCITADNNYYDNKICRRKKNKKVSSKENILSP
CMGKLGKGKKKSKSAKGQWTTEEDRLLIHLVEKFGVRKWSQIAQMLKGRIGKQCRE
RWYNHLRPDIKKDLWTEDEDRILIEAHAEVGNKWTEIAKRLPGRTENSIKNHWNATK
RRQFSRRKCRTKWARPSCLLQNYIKSLNFEKSIRSSHDTTINAPKQEPFGNIPDYDLTSE
VPEFAFDDKLFDENINMDALMDQIHDRCLDLEIPYDELPKLMQGDANKEPDSIDLISEI

>Peaxi162Scf00009g00621.1

SQKVRWSNRLKPDLKEDNFTPQEEELIIKLHATIGSRWSIIAQQLPGKTDNDVKNLWN
TKLKKKLSAMGIDPVTHKPFSQILTDYGNIGGFPKARTHFVSLNRELKGAFMSRPEQF
QHPLEIFSNFNSHCLTTIKLPKAEASEDCFLSNTNDSSNNQPPVDLLTELQAIKFVTDAS
NYSSPKAIFSRSNPINDYSSSPLSSSSSSSASHSLSKTQVERASPGLNWCDYLLDDAFIP
SNFQVQEDTLTIEDKLASGAAQDGQSNMPSMKDSETSLSTRGASSSSSSFVEDMLECE
NEMFLNFPGLSEDPFY*

>Peaxi162Scf00013g00223.1

MGRPPCCDKIGVKKGPWTPEEDIILVSYIQEHGPGNWRAVPTNTGLLRCSKSCRLRWT
NYLRPGIKRGNFSEHEEKMIIHLQALLGNRWAAIASYLPQRTDNDIKNYWNTHLKKK
LNKKMEGHDHENINEVGKSSSSSQSNVPKGQWERRLQTDIHMAKQALCEALSLDTP
SNVDSPNNNNNPTPKILPVDQQPVQTSTTYASSAENIAKLLENWMKNSPKSSESRSSS
KTTQMSSLNNLSIGAVSSSSPSEGTRNVLDSVFSFNSSNNSDVSQSMSIDEGGNFTPEN
NNATIFQVESKPINLPNFKEDNGIFQQVSKPNLETQQVPLTLLEKWLLDDANAQAAQE
EFMGIGMGMTLGETADLF*

>Peaxi162Scf00013g01023.1

MGRTPCCEKLGLNRGPWSKKEDDILINYIDKHGHPNWRQLPKLAGLLRCGKSCRLR
WTNYLRPEIKRGNFTPEEEDTIIKLHQVLGNRWSVIAAKLPGRTDNEIKNIWHTRLKK
KMNQSQIQEKTPDIREQPSETSKSEDSTNIQENTEISSPKPNSNNQHEPSSSLRSSSSITS
SSEGSCSNTTTTSSHIDESRDQMNLENLLEVDDNFWSEVLWATPADDSKDNNLDFSLS
SVEKNYELDSSLNDNWLWDDLFSRANEFLLEFPELV*

>Peaxi162Scf00015g01036.1

MTSLSKSSSSSSDDDIGLRRGPWTVEEDTLLVHYISHHGEGRWNILAKRAGLKRTGKS

CRLRWLNYLKPDVKRGNLTPQEQLLILELHSKIGNRWSKIAQYLPGRTDNEIKNYWR
TRVQKQARHLKIDSSISECSNLIPPGDSGLNTFIKAHFPLDGESYDMDTFSPATCSFKDV
LYYDQMSGENNAPGDVLADSFWSMDEF*
>Peaxi162Scf00015g03118.1
MVRAPCCEKMGLKRGPWTSEEDQILISFIQRYGHENWRALPRQAGLLRCGKSCRLR
WINYLRPDIKRGNFSEEEEETIIEMHQVLGNRWSAIASRLPGRTDNEIKNFWHTHLKK
KLEHNDLRATTTAKRSPHEMIPRWKIEHHISSNYQASQNIANQYPTCHDEDLQENNST
SDTQANKEESMQPTNTQIGHDGDASISNDM
>Peaxi162Scf00018g00228.1
MGRTRCYDKSGLKKGTWTPEEDRKLTAYVTRYGSWNWRQLPKFAGLSRCGKSCRLR
WLNYLQPNIRRGNYTKEEDEMIMKLHAEIGNKWSVIAAHLPGRSDNEVKNHWHTSL
KKRSTQEESTSSTGSERRSNNSKYISSSRNKRRENGTQISANSNIPQLSPKQSCSELSSC
ASVDQHVENMHAEREVFQEEYLFEESSLISFWTEPFIVDNFSTTSDFFVTSQTDGGLVS
PFSPFTRVIWVIFQFCAYHPWHLDGTPDKRDISLAALVVSKSKSLVESFDTKLKENMM
NDEPKKLIIELSVLKMKKLRNHCRPCARAVDPRERSERNNGGRLEKRRAPVMNRQSN
RWPPHA*
>Peaxi162Scf00020g02349.1
MVGMMGWGANSDQQEWRKGPWTPEEDKLLSEYVNLHGEGRWSSVSRCAGLNRTG
KSCRLRWVNYLRPGLKRGHITPQEEGIIIELHALWGNKWSTIARYLPGRTDNEIKNYW
RTHFKKKEKASSKQDKRKTLRQRINNQPQLQHDTNYKFSPQPEEVIMQNSDEINEHN
DTLTFTYHPNMENTIVDLPPVRTSSDISYIWTDNFVMDGLWGGLWNLDVDDHSQPAS
DKCKVAIQNQPTDYQ
>Peaxi162Scf00029g00131.1
MVLGDEKEGMLRKQEKKSKKSNKKAAINGLALTTADVKAIKGDFSFDEEKLTVVET
LIQRDKEEMETKAAGEIDQCGVKRKKKRGGNHLRKSNKDSDEDAAEGVEEKKHKL
KKSCKDEQQTMQYAPLDTEKSAPASASETWDNDKVVETFGVVSSGNAVDEIRRKNK
SKKSIKTSATSGEVTNTEVNQGAVFPSIEEREEGSRTENGNIRKRKNVKLGQRPEDPIH
ENSEKRVRFSGHVQVFPSPNDPSDEKHEIEEETLLRGKRFSKLEDEIIKNAVHKYIEIHN
MGEEGLQKILNSRSHPEIKGCWKEIGSAIPYRPYISVYYRAQVLFRRCETRKWTEEEY
EMVRKFHREHGPKWKVLADELGKHRFHVKDTWRRLKLSNQKKGQWTQEEYQTLF

DLVNTDLRLKLSEDKKSKYGMLRDNIPWSAISDKLSTRTAPSCCLKWYDQLTSPMVA

KGEWADADDYHLIDALFVLDASCVEDVDWDNLLDHRPGEICRKRWNQMVLHIDQC

GNRSFAEQIEVLAKRYRPDLAEVRETWDSKPIVP*

>Peaxi162Scf00033g00251.1

MAQRKESLDRIKGPWSPEEDELLQRLVDQHGPRNWSLISKSVPGRSGKSCRLRWCNQ

LSPQVEHRAFTPEEDETIIRAHAKFGNKWATIARLLSGRTDNAIKNHWNSTLKRKCCS

VSEDLSFETPEQPPLKRSSSVGPGPGPSSPSGSDLSDSGLSCFPQPPLVYRPVARPGPIFP

PPPPVQIPNPQKPDPKPDPVVQPDPPTSLCLSLPGSENQAVQSPTTAQLPPVAPLPAQQG

YEYGGATSATEKQFFTPEFLGVLQEMIRKEVRNYMSAGFVEKNGMCMMQTDAIRNA

VVKRMGISKIE*

>Peaxi162Scf00036g00710.1

MGHHCCSKQKVKRGLWSPEEDEKLIRYISNHGHNCWSSVPKLTGLQRCGKSCRLRWI

NYLRPDVKRGSFSEAEERTIIDVHRILGNRWAQIAKHLPGRTDNEVKNFWNSCIKKKPI

SQGLDPNTHNLLSRNSTQNNKNNASCKVKSTSVFTLETMPSSNKEVPMDMIKSSLAA

FLPLPHNSKTISTAAYNDNMNSINVPRTTTNIDFPRSSLMESTSNNYLSSLTPPGYGIINY

ENYCMWAVSGVERHDFGPSINGHEEMRIVEQEGGQVVQLQEKVFQAEEVYKINHDQ

FNINGQKTYVDDANFSFESVDSALMMPYGIYTSVKSIDHFAWN*

>Peaxi162Scf00038g01640.1

MGHHSCCNKQKVKRGLWSPEEDEKLIKYITTYGHGCWSSVPKLAGLQRCGKSCRLR

WINYLRPDLKRGSFSHQEAALIIELHSILGNRWAQIAKHLPGRTDNEVKNFWNSSIKK

KLLSHGTLSDHLSMFTNFTNPNPSPSFDNFYTLINPNNPVNLLPAINSPLILQVDQMNN

VNEDLNANSMAPSLIPFSFDSIPNDPSWFFNYPHSQHDLDYKQDNSNSIVSSNISLNYT

DAAGGILMDSRNDPIKSSTHQDLLVVPNLLPRLSPPAPLQQNFDSMVVNTSTN

>Peaxi162Scf00038g01818.1

AHVMMDDILIKKGPWKEEEDAVLIKHVKKYGPRDWSSIRSKGLLHRTGKSCRLRWV

NKLRPNLKNGVKFSAEEETTVIELQAQFGNKWARIATYLPGRTDNDVKNFWSSRQKR

LAKILRNSASQPSTPQKNNNKEALALQKVPSVEEPKLSSPAEERSLTMSQCCSSSYMN

NSDTINMVPLPELENSTSLPFEPNLLQFEFTPNDKNYQYIETQMSLSFPQIPLQTDLGHP

LGSQELPMKLEETDLLDFFGQLSTASDIGNVQVPLVPLCSGPDKSSEIVVKREMDSPLT

QDSFIDDLPMDMFDLIDPLPSPSDWWNSDLSISERKKGNCN*

>Peaxi162Scf00041g01811.1

MGRSPCCDENGLKKGPWTPEEDKKLVDYIDKHGHGSWRALPKLAGLNRCGKSCRL
RWTNYLRPDIKRGKFSEEEEQTILHLHSILGNKWSAIATHLPGRTDNEIKNFWNTHLK
KKLIQMGYDPMTHRPRTDLFASLPNIIALASLLQHHPLEDHAVRLQAEAAQVAKLQY
LQWLFQSSSSSSNNYPIATSLNTTQYSNLEDFGPFNLSNSTKESTPSINLSLLENQALFS
NENSGSQLLHNPDTSLPLTDTQEVPFNFQAHLNNNNNSNNTSGDNGQDFNFEICSPSS
PLHNIIPSPPSSSRLPPLTEISISNNQGDASSTNSSNAAEGTSSYWPELFFEDHFMHEIA*

>Peaxi162Scf00042g02519.1

MGRSPCCEKAHTNKGAWTKEEDERLVAYIRAHGEGCWRSLPKAAGLLRCGKSCRLR
WINYLRPDLKRGNFTEEEDELIIKLHSLLGNKWSLIAGRLPGRTDNEIKNYWNTHIRR
KLLSRGIDPTTHRPINEQPTISTQKVTTISFAADNNKDDQDQKIINIKSEFETTSKLQDEI
QERCPDLNLELRISPPHDHQQFNDQSIDELGRRNSLCFACSLGIQNSKDCSCNTNGNG
CSSNVSMNIASYDFLGLKTNGLLDYRTLES

>Peaxi162Scf00045g00138.1

MGRSPCCTNNNLNKGAWTKEEDEILGNYVKIHGAAKWNILCKKAGLNRCGKSCRLR
WTNYLRPDIKRGNFSEEEDSLIINLHSSLGNKWSKIASQLPGRTDNEIKNHWNTKLRK
KLNKLGIEPQTHKQIPNFNLFNSLQMPPLFSQQDVTQLAKLQLLHFQTLLQTINAITTT
FPSNPLMPNINYPMQDDVNGILNFSMPNFEEDDDNLKKGPWTEDEDEKLIDYIKQNG
HTNWQSLARKAGLNRCGKSCRLRWNNYLRPDIKRGRFSPEEEEIIINLHSDLGNKWS
KIAAHLPGRTDNEIKNFYNTHLRKKLLRLGIDPKTHKPISKLNSLVNLSHHLTSNSNYF
LSLASVLSLQAQFSLLAKIQQVIQSPLPTINTINLLSSIQGNILSNSSPQLESLRNETEMS
KFLDSSLANTTTTPCLDNTNL

>Peaxi162Scf00047g01824.1

MAPNGGGVKTSLARNISHGGTRHVLKKGPWTAAEDAILMEYVKKHGEGNWNAVQR
NSGLMRCGKSCRLRWANHLRPNLKKGAFSLEEERLIVELHAKLGNKWAHNEIKNYW
NTRLKRRQRAGLPIYPQDIQPQNQHEHNISSLFDHPQNSNFINPPLSLLDIFNPSTMKPS
VNSQQYQFNNPSPFLTNTNNNNNHFKLFHDPRVSLSLTLASSIRNSQLSSLVAPVPKTF
SQGLTTSMQVPPLQHNYPSFSTNTRPYTAISTNPNGLILGMGIETTTCSRSTGNDYMK
ATSSSDADNYNVVDPGLSRGNSGLLEDLLEESQTLTRAEKIEENCLVDENEASQGKLV
WEEYGLTEESTYSFAHGGDDTTPNSSSGITTKDASLELANQVDDDIMRFLDNFPVAVP

APDWCDDENEQNNTCNGHYFEPRDQMD*

>Peaxi162Scf00050g00512.1

MNPRGEDFFGFQKHHITHSFYLYKQVFSSNIFQPTINHKFKKIPFGSKLVEGKKILKMG

RAPCCDKASVKKGPWSPEEDARLKAYIEEHGTGNNWIALPQKIGLKRCGKSCRLRWL

NYLRPNIKHGGFTEEEDNIICSLYISIGSRWSIIAAQLPGRTDNDIKNYWNTRLKKKLLG

KRKQSQMNRLLLAGGQDLKETNGLEENSLLQNLSNSALERLQLHMQLQSLQNPLSF

YNNPALWPKLTPLQQKMIQTLQATGLSIENQSSLLAQISPSANQVDQLGQKVGINEFA

NTMSTRFKVNNEVEKSTVNNGISSSDSPIDFSNQKDVLDTNIGQENTGEIQGIQGFTQA

EIDDLILINNKGLIADQFDCFKEMDGSSSRDNLAWWSNDFDTNTTSSNSWGSSSNNIV

QQTEGMYQDYALGYNLQ*

>Peaxi162Scf00056g00026.1

MGRRPCCEKKGLKKGPWTPDEDDKLIEYINKNGHGSWRSLPKLAGLLRCGKSCRLR

WTNYLRPDIKRGPFSHEEEKLVIQLHGILGNRWAAIAAQLPGRTDNEIKNLWNTHLKK

RLLSMGIDPQTHEPASAPNGLLRRPPASLSARHMAQWESARLEAEARLSRESQLLVPS

PVGRSETDYFLRIWNSEIGDAFRKFKKGAKTACHSSASQASSCTKCGSASGIKTEVEL

RVAAGSPVTGSNQTEFAEWRNEQPHTEDILQGSDTSSSDAIEDSSESALQLLLDFPSNN

DMSFLGQSDSYSMYPFLTASSLICP*

>Peaxi162Scf00058g00238.1

MEEVKATQVTYSSGMPVTVKLAEDNIEMRKGPWTLDEDSILIHYISLLGQGRWDSLA

QFAGLKRSGKSCRLRWLNYLRPNLRGGKFTPQEQLLILLLHFRFGNRNDYYLCFRWA

KIAEHLPGRTDNEVKNYWRTKVQKHANQLHCNVNSQEFRDVLRYQWLPSLAEQIRV

TPSSSLHEYLMASSSTAHNKTTEVKENISPFLETGIGSTSITSPNVVSSTNSSEAELPPLM

DNSLWSFSSSNWCSSFEDGYYLYSSQDQCHSWASVAGSELVPANETLVSMGKDLASH

GNISSEDTKTSYDEEVRRQCIRP

>Peaxi162Scf00065g00016.1

MNNLKPILEIRGNFAEDEDDLIIRLHALLGNRWSLIAGRLPWRTGDEVKNYWSSHLKQ

KLRNMGIDPMTYRISDYVCRKSHLDLWHESRETETNEKPCDARSSWGQDDHEPSSLP

HLKS*

>Peaxi162Scf00066g01813.1

MAEGGGSGDDTRSSCPRGHWRPAEDERLRQLVEQYGPQNWNSIAEKLQGRSGKSCR

LRWFNQLDPRINRRPFTEDEEERLLAAHRVHGNKWALISRLFPGRTDNAVKNHWHVL
MARKQREQSKICGKRSYPQDNFLSDSKSPSYGFRRRNNNNNNTNIPKTQEGYNYGTK
FSLFEFQNPTKDRVFSVSTYSSSPSWNSPDQLFRRNGSHLLKESSNFSGQKSLCQNNLS
FSTHGGGGATLFPVPNNYKKTTARNPFSYSNNGSDGMTEKVMNISNSTFSFNKILRAS
IEQQRRQQQQHAEEAIEKKDIPFIDFLGVGISS*
>Peaxi162Scf00071g00135.1
MGRHSCCYKQKLRKGLWSPEEDEKLIKHITKYGHGCWSSVPKLAGTFSQEEENSIIEL
HALLGNKWSQIAARLPGRTDNEIKNLWNSSIKKKLRQSGIDPNTHKPLSEIENEHQKA
STTNTKNNNKSTSECNSAFNSEKPKPSSLTIDQSYPLIETNMSSSTGYNLSFQQLNYEP
NTTTIELQNNCNLFDNNNGFSWGAVDSVKSEKEAQEIKWSEYLQTPFLLGNTIQQNH
QSTIIPQDLYSETRSETQFATQGSLSATWLQSQHQQQPNSLQTPDLYHKSFLRLPAAFG
QYS*
>Peaxi162Scf00073g01731.1
MDEIKKGAWSPEEDQKLKAYIKKYGIWNWSQMPKFAGLSRTGKSCRLRWMNYLSPD
VKRGPFSIDEVEIVIKTYQELGNRWSAIAARLPGRTDNEVKNFFHTHLKKHMSLKND
HALMKKKARSKRVMKKTRENEKTNTGKAQERPAVEVVPTKIPMIGSSSNKNSPSPNS
SSSQCNSIITCQENPTDQDCYNNIISSSRVDQPVIMNMESPFILESNPETSESSLGDTNCN
QLIHQFDQYPHDSCNTISDGSSFFDSFDQFDMIISLFLTSLLNFNLVMVAGLSRTGKSCR
LRWVNYLRPDIKRGPFSMEEVEIVMKMYQECGNRWSTIAANLPGRTDNEVKNFFHT
HLKMHLGVKNDVTVKTKGRSKRVKKTNKNEKKLADNAQERSADVASPCSSVVICEE
NHMMDVMDFSKTYQDCYNVSSLVDQSVINMENTVILESNPETSETIDAANCQLHQFD
YSHLCSSLFDDHRFNSLDMNSFWSD
>Peaxi162Scf00074g00355.1
MGRAPCCVKEGLRKGPWSAKEDSLLTNYINQHGEGQWRSLPKNAGNRWSLIAGRLP
GRTDNEIKNYWNTHLLKKLKSVGIVPKPHKIFSKKASKKGQSIQVEKTKVYVPKPIRI
SCGISRNNSFENVTFSTTCSSNSYEDVDVEIENNKDGIMNNENEVNFDICDEFLMEDW
CHFSNKGSLPMNESMVDKVYEEYLLLLSENCYLQEDQREHPLTGNVSEL
>Peaxi162Scf00074g01820.1
MGHHSCCNQQKVKRGLWSPEEDEKLIRYITTHGYGCWSEVPEKAGLQRCGKSCRLR
WINYLRPDIRRGRFSPEEEKLIISLHGAVGNRWAHIASHLPGRTDNEIKNYWNSWIKKK

LKKPSKPSTNTTSCTEHQQQQRSQFSYNTTSQPEILFTQDLGVTKSQILQDSALFTSPN
PLFYFDNGNSLETMTNNVNDRSTNASLFQETSVLNSEFWQVDLQQQVHTSYTTGIHS
NYLPPLLEMPHMEIPSSNNNMMVEGQDHHQLNEWNMDTQQCCPSYLFWDQETGTL
GGDHEFVDPNNTSNNIGQILSSFPSSL*

>Peaxi162Scf00078g00024.1

MCSRGHWRPHEDQKLRELVEKYGPHNWNAIAEKLQGRSGKSCRLRWFNQLDPRINR
NPFTEEEEERLLSAHRIHGNRWAIIARLFPGRTDNAVKNHWHVIMARKCRERSKIYAK
RATAASATTIAHQKSTSDEQELSSLMQQDKRQISNEHTTRSFNPFVDAHQQYFFSERFA
YPCNLTYNYSLYPKLLHKDYLFHHAKVNQDEKQRTEFYDFLQVNTDSNKSEVIDHIN
ASARIMRDDEEVEQEAVDLHRSKSSRGFIDFLSVGDSI*

>Peaxi162Scf00080g00064.1

MDKKPCNSQDAEVRKGPWTMEEDLILINYIANHGEGVWNSLAKSAGLKRTGKSCRL
RWLNYLRPDVRRGNITPEEQLLIMELHAKWGNRWSKIAKHLPGRTDNEIKNYWRTRI
QKHIKQAETMNGQAASSEQNDHQEACTSQMSNGPNDNTIDQTYSPTSYSGNVDTFQ
AGPNFLTEANDNMWSMEDIWSMQLLNGD*

>Peaxi162Scf00088g01621.1

MKDKEVKARMKRGFWKPEEDLILKNCVETHGEGNWATISEKSGLMRSGKSCRLRW
KNYLRPNIKRGMMSEDEKDLIIRLHKLLGNRWSLIAGRLPGRTDNEVKNFWNTHLNK
RSCKGKKKHNKAEEANHQRKNDREYPAETARNQEVATKTVLDSWIEEMQDFNCSLL
SPLPMNSVAFLQDEPFFPILDDIVLLEAFTSTGKEVWPDIQPFL*

>Peaxi162Scf00089g01411.1

MGRHACCVKQKLRKGLWSPEEDEKLCNYITKFGVGSWSSVPKLAGRTDNEIKNFWN
SSLKKKLMKQGIDPNTHMPLSENTQVRDEENCTDKTSMLQMPHLNGLPSAEMDQTF
HTTNRSFNTEAKNGHLTEASKEDQFVSKQVFDIMFLHGYQSNVNPSAYDSEVLAQYQ
QIIRSYDHPSEFEENPNYGICSETSLTKFEHGQTTETDFASSSSNSSNICGYQNNATRIRT
NGMLEINETLSWDVENKMESIFQYPYIEIKNEELKQRPGDFSNCPLSCLTEELCGANL
DVIQQI*

>Peaxi162Scf00090g00168.1

MGRSPCCEKEHTNKGAWTKEEDERLTKYIKEHGEGCWRTLPKAAGLLRCGKSCRLR
WINYLRPDLKRGNFTEEEDELIINLHSLLGNNSTNLNKKITSTTAGASTTTSQCFQQLQ

AHSSGTLLWTRSGLSDSIINNIFISSMVKSDSAAEDSNSSSDLSEEMVHPHLNLELSISL
PQPLEGKELRKEEQLFHEPSFTSASSVTQP
>Peaxi162Scf00096g01718.1
MGRAPCCDKANVKRGPWSPEEDAKLKAYIELHGTGGNWITLPQKVGLKRCGKSCRL
RWLNYLRPNIKHGEFTEEEDNIICSLYISIGSRWSIIAAQLPGRTDNDIKNYWNTRLKK
KLLGKQRKDHRHHKLEMMKENENYFASQDINAYSWPPQPLLFSSLVAPTNEYQQSGS
SGNRHNFQYSTEVSAQFDPSKFSQSSAFVNPLSGNTCNSLSCYYPISNGVITNSFQEYN
NYISVGLDHDVLNVSNLQQVDQSVIEMVNSSSTIISTTSPEISTSWEELSPIVFPPSVSNF
EIQQEISPYFVFEEPRYLGLLKQ*
>Peaxi162Scf00102g01226.1
MGRKPCCDKVGVKKGPWTTEEDKKLISFLVTNNIGQCCWRSVPKLAGLRRCGKSCR
LRWTNYLRPDLKRGLLTEAEENLVIDLHSCLGNRWSKIAARLPGRTDNEIKNHWNTHI
KKKLLKMGIDPVTHEPLNKEEKSRDQSTKTDDIDNKQNGHDDQQVHVVLESTNVTA
AATSLELENSSLCSSSSFSPNENSSCITDESQMVLDTFSENNPLLSSMLHVDAPLIDFQ
WELSASGTTQKQNLDNENFNWLVDCQDFGIHDFDFDCFNMEMEYFLDSIGDIKNEK*
>Peaxi162Scf00102g01858.1
MGRAPCCAKEGLRKGPWSAKEDLLLTNYIKEHGEGQWRSLPNKAGLLRCGKSCRLR
WMNYLKPGIKRGNLSQDEEDLILRLHSLLGNRWSLIAGRLPGRTDNEIKNYWNTHLI
KKLKSAGIEPKVNKSFSKYCSKKQATTDKPRKKQVKKKKNKKIKDQPLVQDTSEPPQ
VVFIPKPIRISSGHSRNYSVDQNVALSTSSSNSADNNNNISNNEGKQAEVSFDPLFDEV
VLDGCCELSPEWSLPTDDSMLEKVYEEYLQLISEECFLQLDDPLAENASHHPMLM*
>Peaxi162Scf00110g00004.1
MQEQGQRRGQWLEEEDERLAMIVASLGERLRRSGKSCRLRWLNYLRPNLNHGHITE
DEERLIIQLQKQLGNK*
>Peaxi162Scf00114g00127.1
MGRAPCCDKANVKRGPWSPEEDAKLKEFIEKYGTGGNWIALPQKAAYTPTLEAGKK
NISYIEVKLIHLCDRWSIIAAQLPGRTDNDIKNYWNTKLKKKLMGLMHSSNQRKSPFF
PSTTLQTITPTQPQTISSLFRYSYQEPNISSVQPNFMYNNNHMNFQLGTNNQYSYFHCQ
ESLVNPIKQEEMGNCLEGQTPRTFDHETQKFTLDYGNNIGGGGDHNLSAWAHDQKPN
GYLANNFQTQFQYDVEEIKELISSSSNGSGCNNNSSLLFVNDENKPNERGMFYY*

>Peaxi162Scf00118g00310.1

MSTSNASTSGVRKGAWTEEEDLLLRECIEKYGEGKWHLVPVRAGLNRCRKSCRLRW

LNYLRPHIKRGDFSLDEVELILRLHKLLGNRWSLIAGRLPGRTANDVKNYWNTHLRK

KLIAPHDQKQESKS*

>Peaxi162Scf00125g01321.1

MLCQLMSSEQMGSWGVIEEGWRKGPWTAEEDKLLIEYVKLHGEGRWNSVARLAGL

KRNGKSCRLRWVNYLRPDLKRGQITPHEERIILELHARWGNRWSTIARSLPGRTDNEI

KNYWRTHFKKKAKNSSANSEKSRARLWKRQQFQQLQQQQQQQQQINDHIDIKKM

MSLFDQNENKGQLMPQGKQEMAMLYPNTTNQQEQVGLFYSMLNGCASVSLPEPSS

NEDTMWDGLWNLDDFYGHFINTSTTYNKATPCLQTMATIPPAFY*

>Peaxi162Scf00128g00742.1

MGRQPCCDKIGLKRGPWTIEEDHKLMNFILNNGIQCWRLVPKLAGRTDNEIKNHWN

TRIKKKLKLLGLDPLTHKPIEQFDHLKNDDIEQQDSSEKEEKEKLELLEKNLPNSLDYT

QEQEIQNSTSSSSLDDMNNFQNSESLETFSKEMFNSGLEDPFQNWI

>Peaxi162Scf00129g00228.1

MGRPPCCDKIGVKKGPWTPEEDIMLVSYVQEHGPGNWRAVPNSTGLRRCSKSCRLR

WTNYLRPGIKRGSFTDQEEKMIIQLQALLGNKWAAIASYLPERTDNDIKNYWNTHLK

KKLKKLEAASCGSDLYSKDSCLSSLSSTSKGQWERTLQADINTAKLALQNALSLEKPS

AIQEYMATDVKPKNICYPYIKQEGNNSTSTYASSAENIAKLLKQWTKSSSSTNNSENS

KGSSSTQLSCNYNNATNDFESISNFESFEQSKSDQFSQATTPEESKFYGESKRELDDQV

PLSVMLENWLFDENEDLF*

>Peaxi162Scf00129g01231.1

MDKRTCNSQDVEVRKGPWTMEEDLILINYIANHGEGVWNSLARSAGLKRTGKSCRL

RWLNYLRPDVRRGNITPEEQLLIMELHAKWGNRWSKIAKHLPGRTDNEIKNYWRTRI

QKHIKQADQNMNKSSKCEQHNDQQAISTSQASTGPTDTIDSYSPPSYTGDTNNNMGN

ITFQGNFPTETNENIWSMEDLWSLQLLNDATN*

>Peaxi162Scf00132g01412.1

MGCKQMDKPKQKHKRGLWSPDEDQKLKDYILRHGHGCWSSVPINAGLQRNGKSCR

LRWINYLRPGLKRGTFSIEEEETVLTLHGVFGNKTDNEIKNHWHSYLKKRVIKKAENE

GDARSGYSSPQIGNEESSHSSMKLASQNSSLDSFEHIECSLLADTDQSSSLQVDFAREN

CKSNLPRVLFSEWLSFDQLNGQDFKNSSNQESKNNFGYNNSVFQDSCMHGPLMNDI
NQALNRGMVDDMFQPTLEFEDHISADGFEELISGEFNINGDMMYI*
>Peaxi162Scf00134g00189.1
MGRQPCCDKAGVKKGPWSAEEDKKLINFILNNGQCCWRALPRLAGLMRCGKSCRL
RWTNYLRPDLKRGLLSGYEETMVIELHAQLGNRWSKIASHLPGRTDNEIKNHWNTHI
KKKLKKMGIDPVTHKPITSDQPNIEQPTKDQPITSDQPTIEQPTKNQQEKQNIMPPSTV
HVVQEMDIDQNKELVETPIQSTVTVTKLEEGTSPKNMEPIQVNNGFCTDEVPLIEPHEI
LVPSESTPSTSSSSSSSPLEDMQFLPSFDNWQCDFNNMDNIGINWAHDFSSTLDYLLND
DDNDMKNISLQDWSQVLEV*
>Peaxi162Scf00135g00313.1
MRIMIKGGVWKNTEDEILKAAVMKYGKNQWARISSLLVRKSAKQCKARWYEWLDP
SIKKAKDEKLLHLAKLMPTQWRTIAPIVGRTPSQCLERYEKLLDAACAKDENYDPND
DPRKLRPGEIDPNPESKPARPDPVDMDEDEKEMLSEARARLANTRGKKAKRKAREK
QLEEARRLASLQKRRELKAAGIDARQRKRKRRGIDYNAEIPFEKKPPPGFYDVAEEDR
PVEQPKFPTTIEELEGERRVDREARLRKQDVARNKIAQRQDAPTAILHANKLNDPEAV
RRRSKLNLPAPQIPDHELEAIAKIGIASDLLGGDELSEGNAATRALLANYAQTPQHAM
TPMRTPQRTPATKQDAIMMEAENQRRLNQSQTPLLGGDNPMLHPSDFSGVTPKKREV
QTPNPLLTPSATPGSSGLTPRIGMTPSRDSYGMTPKGTPMRDELHINEEMDMHNNAKL
GQLSSKKELLSGLKSLPQPKNEYQIVVQPAPEENEEPEEKIEEDMSDRIAREKAEEEAK
RQALLRKRSKVLQRELPRPPIGSLELIRSSLLRADGEQSSFVPPTLIEQADEMIRKELLS
LLEHDNSKYPLDEKAEKEKKKGVKRKLAAEPLIEDFEEDELKEADGLIKDESQFLCV
AMGHESESLDEFVEAHKTTLNDIMYFPSRNAYGLSSVAGNMEKLAALQKEFENVKK
KMDDDTKKATKLEQKIKVLTNGYQMRAGKLWSQIEGTFKQMDTAGTELECFQALQK
QEQLAASHRINNLWDEVQKQKELERTLQKRYGDLISEKEKIQHIMDEYRIQAQMQEE
NAAKNRALELAKAEAEAVKNQQAPHPVAEAPNDQGDMSVDPAPEGSLDGMAEKQS
SPPAAVEASLTDVVQVKLTDEVQVKLTDEVQVKPLATGSEAVDSSVSSQCSNAEENSA
SASQSTVIEVSPTDEVHEKPVDTSSEAVNTIVSSQCSNPEDNSASASQSVASETPPTYDP
IPS*
>Peaxi162Scf00137g01812.1
MGRTPCCEKNGLKKGPWTQEEDQKLIDYIQKHGYGNWRVLPKSAGLQRCGKSCRLR

WTNYLRPDIKRGRFSFEEEETIIQLHSILGNKWSAIAARLPGRTDNEIKNYWNTHIRKK
LLRMGIDPVTHTPRLDLLEFSSILNPSLYNSSQLKVSRLLGGQPLVNPEVLRILANSLLS
SQYTVKPSELVDQNLLMSRNLQGNQLSNPQAQNQLPPLVQDISCGPCSNEAQLMQQA
NLEQFSSNMSNYSSQNCQLNDWQNQEMPSHLTEDYNYHLHNYGYYQQDQSIMDPPP
SDASAFQSNDSNVSYQSILSTPSSSPTPLNSNSSYINSTTTEDERESYCSNTFNFDIQNM
LDANALL*

>Peaxi162Scf00147g00136.1

MGRAPCCEKVGLKRGRWTAEEDEILTKYIHANGEGSWRSLPKNAGLLRCGKSCRLR
WINYLKTDLKRGNITPEEEAIIIKLRATLGNRWSLIAEHLPGRTDNEIKNYWNSHLSRK
VESLRIPSDERLPKAVVELAKKGTQQLNKQRRGRVSRSAMQKNRNNLSSTKGTSTAS
TLSKPQQPPKEINTISMPSTPNIIEKEVLSSTTTSSGQDASNTHLTSDNYASELDNEMLW
HEELDPDFIFTCLWNEEGENLETVENNNSHNSNTEILSVNVDMVSPGNSKINETSTLM
EDGTSLDWDWQYLSEWNEIWAEQEENMSNNNISSRQLSNEALVEIDPVQQVDYNHS
ELVAWLLS*

>Peaxi162Scf00160g00722.1

MGRAPCCDKNSVKKGPWSPEEDAKLKAYIEQHGTCGNWIALPQKIGLKRCGKSCRL
RWLNYLRPNIKHGGFSEEEDNIILSLYISIGSRTDNDIKNYWNTKLKKKLFGKQRKNL
KGNNQKQGSRKGREMSSSMSMVSNENIIANPSWPELPILQPIPYSNEEPRFNDHSSIRK
LLMKLGGKFSDDDNDDEKPMNEPPSNPQYPMDNSLIQPIYDQDCINMMHSPFTNNTH
YNMDAKALCWADTDTERRKLGERMGSDTPVVPTVNDGCNFINELEHMMYTNPQKL
SGLEMLYVDMLNNKHATTLGGSLDLEDMNNLVFPLPPLDASNTEGHQHGTLLQGGA
LNELRLQAMSHPPCVWLQQPGKGWLSEAIMIRESRVEGLSLNGSK

>Peaxi162Scf00164g00073.1

MDHHHKTKVGLGRRGEDYRTSNVMNQENIREEDIMDLRRGPWTVEEDFTLINFIAH
HGEGRWNSLARCAGKSCRLRWLNYLRPDVRRGNITLEEQLLILELHSRWGNRWSKIA
QHLPGRTDNEIKNYWRTRVQKHAKQLKCDVNSKQFKDTMKYLWMPRLVERIQAAA
TTAATSTTTTNTYIQNLENQQSVPNMSHMSQFLPIQLENHKKTNSINHSSMTLTLENSS
TTTSSDNSIGRQVSPTSDLTDCYYNFSINQSSNNQDYTPFNQNYGESLISATGYFPQAV
DQQNSQWMDSEYVSDNSWNIEDMWFLQQQLNNNL*

>Peaxi162Scf00164g00216.1

MAEQMGNWGVIEDEWRKGPWTAEEDRLLIEYVNLHGEGRWNSVARLAGLKRNGKS
CRLRWVNYLRPDLKRGQITPYEERIILELHAIWGNRWSTIARSLPGRTDNEIKNYWRT
HFKKKVKKSIDNSEKTKARLLKKQQFQQHQQEQLKNQIYMKKVMSLLEGNYENEVP
ILPQRKQEMDFMYQNTTEQEQGGGFFYSMLNGYATVPVPEVSSNEDMIMWDHGLW
NLEDVNANFNTAAYNKTLPCLQPLATPFY*
>Peaxi162Scf00165g00111.1
MRERQRWRSEEDALLRAYVRQYGPKEWHLVSQRMNTPLNRDAKSCLERWKNYLKP
GIKKGSLTEDEQRLVIQLQAKHGNKWKKIAAEVPGRTAKRLGKWWEVFKEKQQREH
KENNKVVDPVDEGKYDHILETFAEKIVKERSVPGLLMATSNGGYLHADAPAPSPQAL
LPPWLSNSTAASTVRSPSPSVTLSLSPSTVPPTPTPGIPWLQTDRGPENAPIVLSSFPHH
GIAPPCGENPFVTELVECCKELDEGHRAWAAHKKEAAWRLRRVELQLESEKACKVRE
KMEEIEAKMKALREEQKASLDRIEAEYKEQLAGLRRDAEAKEQKLAEQWASKHLRL
AKFLEQMGCQSRLAEPNGGR*
>Peaxi162Scf00165g00128.1
MDELKVEDCCTENKQSAVASSSSVSENSGSVTLRSPAVSSPTPTSPTHRRTTGPIRRAK
GGWTPEEIKSPFPQDDTLRRAVAAYRGKCWKKIAEFFPDRSEVQCLHRWQKVLNPEL
VKGPWNREEDEKIIELVAKYGPTKWSVIARSLPGRIGKQCRERWHNHLDPTIKKDAW
TLEEERALMDAHRIHGNKWAEIAKVLPGRTDNAIKNHWNSSLRKKLDFYLATGNLPP
ATGEGLQNGCRNTDGTAKAEEFLLGSNKRSEAAVSSGITDTCKIENGGKNHYNARLQ
VAEISASTGGPQTESTDSEVARWDAQSPEIDAIQHIKPSTESEKRYDGRRINSAVDQLN
VIEAPFPCEIPTYGTLYYEPPSLESRSPLDSNLLNICWDQCESDASPSPSSNSFFTPPSTK
GSSLYAQTPESVLKYAARSFPNTPSILRKRKTQGNFSTLINKMGKTGGDICKDKPTDA
CEVNRIDSSEKSGMHNEGLPRNFSNGIGLFNSQPFNASPPYRLRSKRTSIFKSVEKQLE
FAFNNEKKHDTSNGFSDSTDNEISHAVEKATHSPESAS*
>Peaxi162Scf00166g01118.1
MGRSPCCDKVGLKKGPWTPEEDQKLLAYIEEHGHGSWRALPAKAGLQRCGKSCRLR
WTNYLRPDIKRGKFTLQEEQTIIQLHALLGNRWSAIATHLPKRTDNEIKNYWNTHLKK
RLVKMGIDPVTHKPKNDALLSHDGQSKNAANLSHMAQWESARLEAEARLVRQSKL
RSNSFQNPLASQELFTCPTPSSPLHKPIVTPTKAPGSPRCLDVLKAWNGVWTKPMNDI
LRADGSTSATVSVNALGLDLESPTSTLSYFENAQQISNGMIQENSTSLFEFVGNSSGSS

EGGIMNEESEEDWKGFGNSSTGHLPEYKDGINENSMSLNSTLKDLTMPMDTTWTAES
LRSNAEDISHGNNFVETFTDLLLSTSGDGDGGLSGNGTDSDNGGGSGNDPSETCGDN
KNYWNSIFNLVNSSPSDSAMF*
>Peaxi162Scf00170g01335.1
MGRYPCCKLDNDLKKGPWTAEEDEKLKEYIQGHGHGNWQLVPKRAGLNRCGKSCR
LRWTNYLRPDIIRGGFSDDEEQMIINLHSVLGNKWSRIAAHLPGRTDNEIKNLWNTHL
KKKLLKAGIDPVTHQPINDPRLLLSLSNLMNPLESVLRLQAEVTEMAKIHLIQNIIQVL
TPPLIPNLQENNFPMQQLYNLALYENIVTNVTHIDEPSPISNSSDMLLSPSVVENNITGF
SNVSNVNSEHSLPSLVEATPENSPSEQNDIIPNYFGSFSETKDFDAWEKSLDDEASNSF
WQDIL*
>Peaxi162Scf00170g01336.1
MGRYPCCKDDEDLKKGPWAPDEDEKLMDYINKHGHTNWQLLPKKAGLNRCGKSCR
LRWSNYLKPDIKRGKFSIEEEEMIINLHSLLGNKWSRIAAHLPGRTDNEIKNFYNTHLR
KKLLRLGIDPRTHKPISDLNYLINLSQSFTSNNNNHLMNNPLASVLRLQAEVTEMAKI
QQYLQGLFLPPINTNIPFYSSNIQENFARYSSHLFPPLEGHVTNVTNTQFLDSWLVDCA
KSPNLDNSTSCISNSSQSYHQGELNSKVDNFEGLNSSSKTYNDYSNSLPVLAPSSPEAA
H
>Peaxi162Scf00171g00047.1
MCSRGHWRPHEDEKLRELVAKYGPHNWNAIAENLQGRSGKSCRLRWYNQLDPRINR
SPFTEEEEERLLASHRIHGNRWAIIARLFPGRTDNAVKNHWHVIMSRRCRERSKIYSM
RNINNSAQNSTTSPQDTSQNTRRGSPNMNSIDQQLLGRFNYYPNLTFNNSLYPKELYF
DHLRHHLKINEDKNQEVECYDFLRVNTGSNKSEVIDHIARKGTDDEEVEQEAGYHYP
AQSKAPVQFIDFLSVGDSS*
>Peaxi162Scf00177g00512.1
MGRRPCCSKEGLNKGAWTPMEDKILIDYIKVHGEGKWRNLPKKAGLKRCGKSCRLR
WLNYLRPDIKRGNITRDEEDLIIRLHKLLGNRWSLIAGRLPGRTDNEIKNYWNTNIGK
KLQHQRAAANSGHAKSRPPVTTQDIVGSGSSLSSSASPCLVVRTKAMRCTKVFIPTPK
NTSHDNSITTNCNNDDDDKVMAVETTALVASSSSSFTLSSILSEQQPISGSSPVSLSGDQ
FMENSFNFMFNFDMDDPFLSELLNAAENTTTIGGDQVGDSFNRNEKERSYFPPSSSQS
ALFSEETQHNDLELWINGFSS*

>Peaxi162Scf00191g01323.1

MGRTPCCDKKGLKKGPWTPEEDEKLVEYIKKHGHTSWRSLPNLAGLDRCGKSCRLR

WTNYLRPDIKRGPFSQEEEKLVIQLHGILGNRWAAIASQLPGRTDNEIKNLWNTHLKK

RLLSMGIDPQTHQPSTAPNGLLRRPPTSSSARHMAQWEKARLEAEARLSREPQLLVPS

LVGKSETDYFLRIWNSGIGEAFRKLKKEEKTTCQSPASQESSSTKYGSDSGITTEIDLG

LAGSPAAGSNQNEDTKWKNAQPYTENFLQGSDTSSSSGLEDSSESALQLLLDFPSPAA

ERLCILTPLEV

>Peaxi162Scf00194g00218.1

MGRHSCCYKQKLRKGLWSPEEDEKLVKHITKYGHGCWSSVPKLAGLQRCGKSCRLR

WINYLRPDLKRGTFSQEEENLIVELHAVLGNKWSQIAARLPGRTDNEIKNLWNSSIKK

KLRQKGIDPNTHKPLSEVENEEKASANSNKNNEKVISEDSNDQLNFIEAHESISKHIGI

ATEKSSTLSTMTNNMDRYPLIHETNNIVPPTHEFFTKSPHDLASYFSFQQLNNYSPNSI

GLSMTNSNTNNLIFNNSTNSKNSHDMVSDQFNSCTMATDPEEIKWSEYMQTPYLLSA

NHHQMSQHQDLYGDAKSEAQFITQGSLNLSNTTWLHQSQQQQSSLQTADLYNKNFQ

RLPAAYGQYS

>Peaxi162Scf00203g01315.1

MKTCAVRDSDEKDKGMGHHCCSKQKVKRGLWSPEEDEKLVRHITTHGHGCWSSVP

KLAGLQRCGKSCRLRWINYLRPDLRRGSFTEQEERTIIDVHRILGNRWAQIAKHLPGR

TDNEVKNFWNSCIKKKLIAQGLDPNTHNLLSTHQNKNNKTNNSSKTKSYHQDSTSVF

TIDTSTNKEVISMDIKATLAALPPFLHSNNNTSSTYHYTTPIVPIIEYQNPSTFTLSERNN

NGSTTTQHSVLDFASNTSINSTTNNVSSSTLTPLEFGYINENCMWAGTGLEPTTLNPAT

GGTEEMQVQLQGEQFPIQTKFCDQEDVYKVNQTVENTFDNSNFDFDFVDSALVPCGL

YNSVNSMDQLSWDC*

>Peaxi162Scf00222g00521.1

MTSYSCSPMGTTTTTTSMGMFYADMNSLSITSINYVTSDGGVGENSNETIDLNASCCF

NEEKQITMHSNFSSGNNGKEIESGQSKLCARGHWRPAEDAKLKELVAIYGPQNWNLI

ADKLRWFNQLDPRINRRAFTEEEEERLMAAHRLYGNKWAMIARLFPGRTDNAVKNH

WHVIMARKYREQSSAYRRRKMGQFVYRRTTTSLVEEEDSSFVSSNSSSGKEVVAATM

KGENPTPIISGQTAANPFASLKINNDGPSGWVVYGPNGSSHMAASAGEAAPPSTNVIV

GMK*

>Peaxi162Scf00222g00715.1

MGKSKCCDKEGLKKGPWTPEEDQKLLLRWINYLRPDIKRGKFSLQEERTIIQLHALLG
NRWSAMAAYLPSRTDNEIKNYWNSRLKKRLTKMGIDPMTHKPKTDNGNSSQSKYVA
NLSHMAQWESARLEAEARLVRRSKDSKMLFNNTHNYNQITSQPYYQLPCLDILKAW
QRSSTKPTTNDINAILLDGNFATRNKSLESSIPSTSNNFSGNVVMNNVITTTTTTTTVG
DNLPLSSINCMDEDLCPSNFMQEFSELFPEYNTCAQNPENYSTGQLDNFMGGCFLDFE
DNKYFNWNNFSHLVTSPVGSPVF*

>Peaxi162Scf00222g00821.1

MGKSKCCDKEGLKKGPWTPEEDQKLLSCIEEHGCGSWRALPAKAGKFSLQEERTIIQ
LHALLGNRWSAIAAYLPSRTDNEIKNYWNSRLKKRLTKMGIDPMTHKPKTDNGNSSQ
SKYVANLSHMAQWESARLEAEARLVRRSKDSKMLFNNTHNYNQITSQPYYQLPCLDI
LKAWQRSSTKPTTNDINAILLDGNFATRNKNLESSIPSTSNNFSGNVVMNNVITTTTTT
TVGDNLPLSSINCMDEDLCPSNFMQEFSELFPEYNTGQLDNFMGGCSLDFEDNKYFS
WNNFSHLVTSPVGSP

>Peaxi162Scf00228g00914.1

MDHHHHVKVGSSHGGGANCNKNNQQQHDEDMDLRRGPWTVEEDFALMNYIAHH
GEGRWNSLARCAGLKRTGKSCRLRWLNYLRPDVRRGNITLEEQLLILELHSRWGNR
WSKIAQHLPGRTDNEIKNYWRTRVQKHAKQLKCDVNSKQFKDTMRYLWMPRLVERI
QAAAANNTTNSNNPSKSQMVHQTINSNMSGMSEFVPIHDNVVSTNKPRLMSTENSST
AAASSENSFGTQVSPVNSDLTDCCYNYPPVNQDYFQVSHHQSTTNNDQLCHGESLTS
PTGYFHHGTGLDQFQGMDQQQNMNSSQWMDGGNNFSDSLWNIDDMWFLQQQLNN
NNNNIV*

>Peaxi162Scf00268g00046.1

MGRSPCCEKAHTNKGAWTKEEDQRLINYIRAHGEGLLRCGKSCRLRWINYLRPDLK
RGNFTEEEDDLIIKLHSLLGNKWSVIAGRLPGRTDNEIKNYWNTHIKRKLISHGIDPQT
HRPLNAAANPAATTTITTNTTKNICMDFRNNVDQKPHINIINKTNATDSSNNETKCSSV
TTEETQPLELPKKNTQVMINLELSIGLPFHAKTDHIYSSNSAESTAPYNFLAAAAAPPP
TVAAVPVVAMAELVAETACLCWQIGFQGGQSCGKCKTTTGFYRFC*

>Peaxi162Scf00269g00088.1

MGRSPCCDKNGLKKGPWTPEEDHKLIQYIQVHGPGNWRNLPKNAGLQRCGKSCRLR

WTNYLRPDIRRGRFSFEEEETIIQLHSVLGNKTDNEIKNYWNTHIRKRLLRMGIDPVT
HSPRLDFLDLSSLLNSTQLNLSSLLGLQALVNPEILKLANTLFLPHSENQELLLLKLQE
NQLLNPQHIQNSEGPEMLLQKLLQSQLVNSPLHNQSSIFQHNTNQIHNQIPEIPNCTQQ
NVSCSSSQSMQGNMGRYLMNGQIFQENMMLPPQNYSHTSTPDASENSTFQSLNNSSN
QNSQNFSFDTPLSGTEEEKESYCSNFMKFEIPESLDFDDLL*
>Peaxi162Scf00273g00087.1
FKFHPKDPKKANVIVCALGNICRHWQSLLLVSLLLLLAIELCTLSVSLAFLDAVSTLKG
PEERNILLQCGKSCCLRWTNYLRPDIKRGPFSEEEEKLIIQLHGILGNRWAAVASQLPG
RTDNEIKNLWNTHLKKRLFSMGIDPHTHEPSSVPSGLVRRPPTSSSTRHMAQWGSARL
QAEARLKIHIFRFPL
>Peaxi162Scf00274g00625.1
MGRPPCCDKIGVKKGPWTPEEDIILVSYIQQHGPGNWRAVPTNTGLLRCSKSCRLRW
TNYLRPGIKRGNFTEHEEKMIIHLQALLGNRWAAIASYLPQRTDNDIKNYWNTHLRK
KLKKLQGNDDENSSSTSQEGISSSNISKGKWERRLQTDIHMAKKALCEALSLDKPIAN
PTQTPVQESVQPCTTYASSAENISRLLQNWMKNSPKSSQLSQSNSESTTTQSSSYNNFS
MGQVGSSSSPSEGTISVATPEGFDSLFSFNSSSVDEGNAAIFQVDSKPNLPNLNAANGF
FLEESKPTLESQVPLTLLEKWLFDDAINAPAQEELMGIGMGMALGEAADLF*
>Peaxi162Scf00276g00412.1
MERRTSGPKRRSRQWNAEEILLAAECFNDRTDVQCLHRWQKVLDPELVKGSWTKEE
DEILIELVNTYGAKKWSTVAQHLAGRIGKQCRERWHNHLNPAINKEAWTQEEELALI
RAHQIYGNKWAELSKVLLGRTDNAIKNHWNSSVKKKLDSYWASGLLAQFPSLSNIN
HQSQSIPSSSMKLLHNSDDESVHQDGIVKEEVSECSQGSPLAGCSHCTSDWGNTFVHI
RENDRMSEESLNKNNASSSAAPCSRNYTPDFHDVSCSLPEVPTELEDSKFLEHNYSHD
LGNSIGKDWQFNREDIPNISLEVIQESSGLFTHLLNCNENHNMVPFPLQTLTGFGTANA
GNVVEGPYRPNEMFSSVDGCRIIHPEEGISQCSPYETGVNGNGAPEDSLIYQSSNDLIL
ETRDMAPQNCNASSLHDFEASRHQAFSVHSQFSSENRSIVFGIASNHPLISPLENPIQES
FTSGCDGFIYPSESAAPADNGTDNSFLADRLDHTNDSCRLVSVNDLGSTTSNTVQKG
MLNENTFVPEEQKDGGALCYEPPRFPSLDVPFLSCDLIQSGSDTQQEYSPLGIRQLMM
ASANCLTPLRLWDSPSRDDSPDAILKSAAKTFTWTPSILKKRHRHLVTPLSEKRCEKRL
EKDFNQESFSIMTAEFSRPQDMFDQSANEKASTEDKENGHPSSEAGRKEEGDGVTGL

SCCGNSERRLDGGAHYRKEPHGKSAGANDGIGNVKQPPGVLVELSSNDLFFSPDRVL
VKCDRATSLSNKALANHYARRLEAASKQVTVSSSFETSCSSVGCSPAVHGKCRSSFVI
ATSTALENTAENSENGFRAETESIFGETPFKRSIESPSAWNSPWFMNSFLRSPRCEVELA
FEDIALFMSPGDRSYDAIGLMKQLSEQTAASIADAHQILGNDTPETILLQTNSKKQKA
DENCSLLTSNAVSERRTLDFSECGGTPPAKGKGTTKFDSGTSFSSPSSYLLKCCR*
>Peaxi162Scf00284g00077.1
MEGTTGDKIKGSWSPEEDNMLIKLVDQHGPRNWSLISTGIPGRSGKSCRLRWCNQLS
PTVQHRPFTPSEDAIILQAHALHGNRWATIARLLPGRTDNAIKNHWNSTLRRKRHGPP
LSRSGSSSDQSNSKRHCTRASQEQSSCGLDCDDLGLDGPNGLDGDELGLYGPETSLTL
SLPGGGSIDSPMKEDVPVKESEPLMNSDNKVKEEKRTVEIEETCLVTIMQRMIAHEVR
CYIDKLRAQGGLGIGPGVEFEVPKNP*
>Peaxi162Scf00295g01125.1
MGRSPCCDESGLKKGPWTPEEDQKLTNYINKHGHGSWRALPKLAGRTDNEIKNFWN
THLKKKLIQMGYDPMTHRPRTDIFDSLQQLIALAKLKELMEHHSWEEQAMRLQSEVA
KLNYLQNFLQPHNTSFNAIQDIEAYNLLNSLSSFKDSLVLGTNNHLQIPTISTSSSLQAI
QDSIPFSHLPELQTPRSNFKTSLNKDSVRPQNTEFNVMSHGETSPTSPWLTSSLSPSPPP
PVINDETKNENSLEVMISSCLPEERKLSDHLFQPNNERSLSNEGAPPSVWPDLLLEDSF
FQDIVKF*
>Peaxi162Scf00304g00074.1
MKVLIKGGVWKNTEDEVLKALVMKYGKNHWARISSLLVHKTAKQCKARWYEWLD
PSIKKIEWTRKEDEKLLHLANLMPSQWRTIAPVVGHTPSQCLVRYEKLLDDNENYDL
SDDDPRKLRPGEIDPNPESRPACPDPDDMEEDEKEMLFEAWARLANARGKKAKRKSR
EKQQLEEARRLASLQERRELRAAGLIDVHQRKRKRSGIDYNAEIPFEKKPPPGFYDVT
DEECTVGLQPKFPYTIEELEGERRVDKEARLR*
>Peaxi162Scf00304g00919.1
MVLLAAAMKNMQEPYDKNVGLNSNKGAWSPEEDQKLISYIMKYRIWNWNQIPKFA
DLKRGPFSMEEVETIIRMYQSLGNRWSAISKELPGRTDNEIKNFFHTHLKKQFGTKVN
DHVQVKRKASCKKSKKAKELEMRTRGETQEKPNQSLDMSSSDITFDENHNWDFTNF
SETSSEDNYCTVNSLVNQEAVPIIDSMVILESNPTTSENHDLYVQDFMDETGINSSNKL
HNKNMGTNSKKGPWSPEEDLKLTSYIMKYRIWNWNQMPKFAGLSRTGKSCRLRWV

NYLRPDLKRGPFSMEEVETVIRMYQSLGNRWSAISKELPGRTDNEIKNFFHTHLKKQF
GTKVNDHVQVKRKATCKKSKKAKELEMRTRGETQEKPNQSLDMSSSDITFDENHNW
DFANFSETSSEDNYCVNSLGAVPIIDSMVILESNPTTSEDPDFSVQDFRDETSINSSNVD
FWLELYMAAENLA
>Peaxi162Scf00304g01018.1
MQKLHNKNMGTNSKKGPWSPEEDQKLTSYIMKYRIWNWNQMPKFAGLSRTGKSCR
LRWVNYLRPDLKRGPFSMEEVETVIRMYQSLGNRWSAISKELPGRTDNEIKNFFHTH
LKKQFGTKVNDHVQVKRKATCKKSKKAKELEMRTRGETQEKPNQSLDMSSSDITFD
ENHNWDFANFSETSSEDNYCVNSLGAVPIIDSMVILESNPTTSEDPDFSVQDFRDETSI
NSSNVDFWLELYMAAENLAG*
>Peaxi162Scf00305g00045.1
MGRQPCCDKVGLKKGPWTADEDKKLINFILSNGQCCWRAVPKLAVILDDGIGAGLLR
CGKSCRLRWTNYLRPDLKRGLLSEYEEKMVIDLHAQLGNRWSKIASHLPGRTDNEIK
NHWNTHIKKKLKKMGIDPVTHKPLSTITNDHPNKQQPKDLPVIQQENLQEIIMPPSSV
NDVSEMDIETPIEQSAISEIKVEEDNNNKNMETTSCKNNINISFDSTTVEVNNNGFCTD
EVPLIEPHEILVPNSESTPSTSSSSLSTSSSSSSSSILEDLKFLPSFDEWPSDYYNMEQNNM
GLGWENDFSSTLDFLLNDDNDINQFPRDDESWKFDQLL*
>Peaxi162Scf00311g01112.1
MGRAPCCDKENVKRGPWSPEEDATLKSYIEKHGIGGNWIALPQKAGLRRCGKSCRL
RWLNYLRPNIKHGEFSDEEDRIICSLYANIGSRWSIIAAQLPGRTDNDIKNYWNTKLKK
KLMALDLSSQQISPNYQSTPTYHSSISALQSSSHNSSLYSSPSPSLSLKSTAYKYTNSHF
YPISTATNFPCNETIISSPSSKFLNSPSYMYNGVEEKQSNSKFLITNGNDYAEKKPKGKG
CYDGENSLECSLEEIKKLISTTSIHDNNFNNFFSDENKIEEQVMMSTDVDHN*
>Peaxi162Scf00330g00418.1
MDELMKLEGCCTENKQSTAAMSSSVSENSGSLTVKSLEVCSPTPTSPAHRRATGPIRR
AKGGWTPEEEEKKSWLCLTTDAYAAEFFTDRSEVQCLHRWQKVLNPELVKGPWTQE
AGFSFPLYQSEDDKIIELVAKYGPIKWSVIAKSLPGRIGKQCRERWHNHLNPNIRKDA
WTLEEELALVNAHRVYGNKWAEIAKVLPGRTDNSIKNHWNSSLKKKLDFYLATGNL
PPVARDVLQNGCKDTVKTASAEKALVCSHKGSDSTEVASSGTTDVCKLENGDKNHQ
DTKLLVANIGASISGPQNGSDSEVVRWEAESPVIDTIHVKPITQIRFEGYGINSEVDQFK

VIETPFSCEIPTCGTLYYEPPQLGSCISVDSDHLNIRSRLCESDASPSLSPSSFSTPSSLKG
SSLYAQTPESILKIAARSFPNTPSIFRKRKLSSTSSNKIGNADGDTSKDEVHDACEEKQT
DSSEKYGMHRESSSRNPSYNGIGPCNTKAFNASPPYRLRSKRKSIFKSVEKRLKFAFD
NEQPDSTGISDSAAKETSQVAKD*
>Peaxi162Scf00342g00113.1
MSITSETDDWMTSKVDMDSPDEANGGGNVGGSLPLKKGPWTSAEDAILVEYVMKH
GEGNWNAVQKHSGLARCGKSCRLRWANHLRPDLKKGAFTLEEERLIIELHAKMGNK
WARMAAELPGRTDNEIKNYWNTRIKRLQRAGLPVYPPDICFLASQNKQNEELAGLSS
TDAQHPGLVATNNFDIPPVEFKNLELNQLLCPPAHVEIPASSLLDIPASGFLAQVHSAPY
SSTSLLSTVHPSKRIRGSESMFSGSNGDLFLASCQYQNGGSLIAQPLGFSSYNHNLTYD
GQQSFSNVIPGSHAPLNGNSSTSEPTWALKVELPSLQNQTANWDSPSSPLHSLESVDT
LIQSPLAGHSESVSLSPRNSGLLDAVLYGSQTMNSSKDNSNQAKEETSADAVGNSCPD
LLEKGWETYGDPISPLSRLPASVFTECTPTTGNSLHELQSTSRMPGENGCNVKQENAD
QAPSDGKDDMLNQTIFASNCSSPKTQHTKNNLALKNAFGSGFFDDYGWDCKQVHSV
ATSSGQACGRDSAWDAMSAM*
>Peaxi162Scf00349g00057.1
MRTPSSSSTTSNKVTPCCSKVGLKRGPWTPEEDEILTNYINKEGEGRWRTLPKKAGLL
RCGKSCRLRWMNYLRPSVKRGHIAPDEEDLILRLHRLLGNRWSLIAGRIPGRTDNEIK
NYWNTHLSKKLISHGIDPRTHKPLKNSNSSSDDITNKLASSSPPSSSKANDLNPILSPTY
ISSFQMEEPLGKINTHPGEITSLDDQYQSNAILAEYGDDLNIAVTIEEDVEMNCCTDDV
FSSFLNSLINEDMFACQNQQTNGTFQDFDPFMASSSTPSSDQYNPS*
>Peaxi162Scf00354g00068.1
MFSGLKRCGKSCRLRWLNYLRPDIKHGGFTEEEDNIILTLYTQIGSSSILVKIRWSVIAS
NLQGRTDNDVKNYWNTKLKKKLLEAAATNTLSNSNAFTHDKTYMNTIPRTCYGTDS
TNCSNMKFYTFSHAMDTNLLGHSSRQLFPLLPELMEIQENGILQQAEEVKCSSLENSF
ASFSGNIGVVQGNEEDGSLMSLSSGISSSYYYDLLNGFNFQENVVMGVNTNFSNYSS
NSNLLQLQANIPDSTDQPFKGFETY*
>Peaxi162Scf00362g00831.1
MGRSPCCEKAHTNKGAWTKEEDERLIAYIRTHGEGCWRSLPKAAGLLRCGKSCRLR
WINYLRPDLKRGNFTDEEDELIIKLHSLLGNKWSLIAGRLPGRTDNEIKNYWNTHIRR

KLMSRGIDPTTHRAINESSTTTQKVTTISFGAGNKNKDIEDLKMINVKAESGLSQEDE
NSSSSQLFQEQCPDLNLELRISPPYQQNQPDHQALKQSPTRGHLCFACSLGIQNSNDC
NCSNGCSTNMGMNIASYDFLGLKANGVLDYRTLETTK*
>Peaxi162Scf00371g00087.1
MGRTPCCDKNGLKKGPWTAEEDQKLIDYIQKHGSGNWRTLPKNAGLQRCGKSCRLR
WSNYLRPDIKRGKFSFEEEETIIQLHSILGNKWSAIAARLPGRTDNEIKNYWNTNIRKK
LLRMGIDPVTHSPRLDLLDLTSIFNPSLYNPSHMNNMSKLLGVQPLLNPEILRLATSLL
SSQRQNQNFLLPSNFQENQSCDSQVQNQMTPFVQASQIQIPIQNISICTPLSTPCVPFSS
EAQVMQQPNMEQFPSNLSYFSAQNCQQNEWQLNNGIASKMNEHNFPSQNYVFSTLS
TPSSSPASFNSNSTCINSSSTTSEGERESYSSSMLNFDIPNIFDVNEFL*
>Peaxi162Scf00377g00925.1
MSEIIKKGPWKEEEDAVLIKHVKKYGPRDWSSIRSKGLLQRTGKSCRLRWVNKLRPN
LKNGVKFSAEEERTVIELQAQFGNKWARIATYLPGRTDNDVKNFWSSRQKRLAKILR
NSASQPSKPQKDNNKEAPALQKVPSVEEPKLSSPAEERSLPMSQCCSSSYMNNSDTIN
MVPLPELENSTSLPFEPNLLQFEFTPNDKNYQYIGTQTSLSFPQIPLQTDFGHPLGSQEL
PMKLEETDFLDFFGQLSTASDIGNVQVPLVPLCSGPDKSSEIVVKREMDSPLTQDSFID
DFPMDVFDLIDPLPSPSD
>Peaxi162Scf00391g00621.1
MGRSPCCEKAHTNKGAWTKEEDQRLINYIRTHGEGCWRSLPKAAGLQRCGKSCRLR
WINYLRPDLKRGNFTEEEDELIIKLHSLLGNKWSVIAGRLPGRTDNEIKNYWNTHIKR
KLISRGLDPQTHRPLNSTITTNITTSTKNICLDFRKNTSIAATPLSQNDQKPMSSSISSLE
DTKCNSSTTEEVQPLDSPPAAPPVTGEVMINLELSIGLPIFQAKMTDQSSSISSNSAKEM
GGKTVCLCWQVGSQNGQQFC
>Peaxi162Scf00411g00008.1
MVQEEVRRGPWTEQEDLQLVFYVKLFGDRRWDFLAKVSGLKRTGKSCRLRWVNYL
NPDLKRGKMTPQEERLVLELHAKWGNRWSRIARKIPGRTDNEIKNYWRTHMRKKAQ
EQRKKTCVSPSSSFSNCSSSSSITHEENERNFYDTGGLELLQSADGQKKVSGHEKGES
MKVYSMDEIWKDIELSEENDTITSNKLPVMANVSPLWDYCPASLWISDEEESKMFPPF
SEHVDDHLQVYSFDKKNRTF*
>Peaxi162Scf00420g00240.1

MGRQPCCDRVGLKRGPWTIEEDHKLVNFLLNNGIQCWRTVPKLAGLQRCGKSCRLR
WINYLRPDLKRGTLSEDEEDQIIQLHARLGNRWSKIASNFPGRTDNEIKNHWNTRIKK
RLKLMGIDPLTHKPLEPIDHKKYKHQPDDHRTELSRTENSNFDNANNDFSVPNDEYN
NMLCGNLEVELWNKSCKTMSSVTCYSSSTSLDADDSINLSTVSAATTESSSNLAPVED
QQDSIQQWMDSLFSCSVNQLEDDMFFLRKYN*
>Peaxi162Scf00428g00117.1
MRKPEHGSVMKEKGNNNNKAKLRKGLWSPEEDEKLMNYMLTNGQGCWSDIARNA
GLQRCGKSCRLRWINYLRPDLKRGAFSPQEEELIVHLHSILGNRWSQIAARLPGRTDN
EIKNFWNSTIKKRLKNNNNNNNNNNSSNNNTPSPNTSDSSSDLRVLMGGSIFPMQGH
DVNVMAGLCMDNSSSTTSASSLQAIVPNNHFNPFPQLDSTNYDIIGAAGLYNLPPCLG
QFGSSSGHDGGVVDYGVVEAYSSMGLGSDFSVPPLESGNKGTMGENSNIVNFSSNVS
AVNDYSLFDKKTNGNNQQLMSDSSDQSLKVEDYMVGFGNSHHHHWHGESLRIGEF
DWEGLLANVSSLPYLDFQVE*
>Peaxi162Scf00451g00059.1
MGRSPCCDKVGLKKGPWTPEEDQKLLAYIEQHGHGSWRALPIKAASSSITYTAYGFD
DLQFVGLQRCGKSCRLRWTNYLRPDIKRGKFTQQEEQTIIQLHALLGNRWSAIATHLF
KRTDNEIKNYWNTHLKKRLTKMGIDPVTHKPMNDTLLSTNDNVQSKNSANLSHIAQ
WESARLEAEARLVKQSKFHSYKLDTPRTSTFFDHYSSQELLTPSSSSSHLKNLMPVTS
GLQDFNFVERSTGFLLCTSADGNLSSGGMDSDSSGNASEQCEDNNYWNSILDLVNSS
PTESTMF*
>Peaxi162Scf00452g00412.1
MGKGRAPCCDKSKVKKGPWSSEEDLRLITFIQKHGHGNWRSLPKQAGLLRCGKSCR
LRWINYLRPDVKRGNFTPQEEATIVKLHQSFGNKWSKIASYLPGRTDNEIKNVWNTH
LKKRLMKKIDGNQAKDGRSCVSPPFSPSSSTSVVSHTNWSQDISTKANEVEFKDDML
AEKEPKEATKSCSPTTSYGSNLSNLSQVEISSPKGLDMDNWILSPKVHDIHDDGVIEIP
SMESDIDFWDMLDILDPSPTTTTTTSNSDSKQDNQPVCQGVSDSECQKWLRYLENEL
GLSQSTGANCYECTDAVTSQDKEAHPFTT*
>Peaxi162Scf00485g00916.1
MGRSPITSDKSGLKKGPWTPEEDLKLIQYIEVHGPGNWRSLPKNAGLQRCGKSCRLR
WTNYLRPDIKRGRFSFEEEETIIQLHSVLGNKWSAIAARLPGRTDNEIKNYWNTHIRK

RLLRMGLDPVTHSPRLDLLDLSSLFNSTQLNLSSLLGLQALVNPQFLRLATTLLTSHTE

NNQEMLLQRLQANPTV*

>Peaxi162Scf00503g00613.1

MQNLKKSGNSSGDVAAAKPKERHIVSWSQEEDDILREQIRIHGTDNWTIIASKFKDKT

TRQCRRRWFTYLNSDFKKGGWSPEEDMLLCEAQKIFGNRWTEIAKVVSGRTDNAVK

NRFTTLCKKRAKHEALAKENSNSFINLNNKRVIFPDGLSIDKITEAAAPIKKLRMSGVS

DVPQDGSSKGNSFGDCGTTHPLLRHPFAVLAQNFHNAAGNLASHQQVNNMKESTEN

ATDSKTQGTFLKKDDPKIHALMQQAELLSSLAMKVNTENTDQSLENAWKILQDFLH

QTKEGDMLKFQLPEMNIDLDDYKDLMADSRSSNEGSRPSWRQPALSEDSAGSSEYST

GSTLLSHALADKTEESQAEGCAHLQDIESELRNSQMSDQGGIHESENGTSCRVSTTPD

TLPVCDEEKANNGPATAECEFLNTDFSSPLQVTPLFRSLAETIPTPKFSESERQFLLKTL

GMESITPHPGTNLSQPPSCKRALLHSL*

>Peaxi162Scf00515g00013.1

MAFQLTMQEEELRKGSWHEEEDERLASIVAILGERRWDALAKASGLRRSGKSCRLR

WMNYLRPNLKHGYITADEEHLIVKLQKQLGNKWSKIAKQLPGRTDNEIKNHWRSHL

RKKSLIYEQECCGSNTSKSGQISSASKSDSINTPNNSTGDSISGKDDCSSADSNETGLSD

WIPSWSYEQSQMEHHMYLCRLNLCFCHPQCFSEDNNISTWGDTSSSIWEQ*

>Peaxi162Scf00516g00673.1

MGRSAESDDMSGLKKGPWTPEEDQQLLDYIHKNGHGNWRALPKLAGLNRCGKSCR

LRWTNYLRPDIKRGKFSEDEERLIIKLHSLLGNKWSAIATRLPGRTDNEIKNYWNTHL

RKKLLQMGIDPVTHRPRTDHINIFNALIGNIPPQLLAAASSNFMNTDNTSINNLTNILFS

DTAQQLVHQIQLLQNSSSLLVHSLFNNAATSTACPLNMEGLNQNFGLQNLCDQNQLQ

EYNSILMNYQQFLSNTSDSLTGFALNSSNSTDISATNGCDQLMVMSGTCSTSSSCGNN

AASVTHANDDQMKNISKAALHHDTPECPSSSNKMPQGNMISDIYPKQELVQPSSASM

STFEPWGQIMGDEEASDSYWRDIIE*

>Peaxi162Scf00521g00814.1

MADKGQSSSSVNTPADSQDVVAPRMLVSGKTSKVAEIEFSEEEEDLIIRMYNLVGERW

SLIAGRIPGRSAEEIEKYWNTRSSTSQ*

>Peaxi162Scf00523g00004.1

MNRLFESDSVKFSKYSNSLWNALSSGANFNRRTSGPTRRSTKGQWTPEEDEILRKAV

QRFKGKNWKKIAECFKDRTDVQCLHRWQKVLNPELVKGPWSKEEDEVIVELVKKYG
PKKWSTIAQHLPGRIGKQCRERWHNHLNPSINKEAWTQEEELTLIRAHQIYGNKWAE
LTKYLPGRTDNSIKNHWNSSVKKKLDSYLASGLLAQFPALPNVNHQNQSIPSSVKLQ
QSSEDDSVRKEGTEMEEVSECSQGSNLAGCSQSTSDMGNRFVHTREDRKLLEDSNYR
MDPSSSSAPCSEYYTPAFDDITFSMEEVPSELSESKFLEQNLSHDWRTSMGKDWQFNP
DDDIPDISPLELMQESSGLFMQCLTGNGNHDMVTFPQKNAMSFGTTTNMGGMVVGL
DKPTEMFTSVDDCRMVYPEAGIPQCFPSETGMNGVDETADSLIYQSSNYQISEADMSI
EKCNPLCSDVMGSQPFSVPSQFSSEQSSSMFGTVSNQFHDLSHGNPALESHTSKCDAF
LYPVESGTPCDNIMNDPLLKEQLHQTEDSVQLVSVNDFSSTPPSTLQTRPLVNENLPVP
EEQKDAGALCYEPPRFPSLDVPFFSCDLIQSGTDAQQEYSPLGIRQLMMSSVNCLTPFR
LWDSPSRDGSPDALLRSAAKTFTSTPSILKKRHRDLVSPLSEKRCEKKLESDFRREAFS
DLARDFSRLDVMVDESANEKATISSATADQALELRASSEDKENISPTEDGRKEEDKGH
NGLLSSGTSERQLDGADVHYKEHGTREGKRVGANDAKGKIKQPSGVLVELNTSDLFF
SPDRFGAKSDRATNLSNKALGNQYARRLEAASNQGSVSSSFETSCLSVICSPRIRGKK
DGSSFIITTSMQSATTSTALDNSAETSGNGVGAETVNISGETPYKRSFESPSAWKSPWFI
NSFMSSPRLDTELTFEDLALFLSPGERSYDAIGLMKQLSEQTAGAFADAQEVLGGETP
ESIIRGRNSKNQKANENHSLLSANAVSERRTLDFSECGSPGKGKDAENFCTNDRSFSSP
SSYLLKGCR*

>Peaxi162Scf00526g00820.1

MVAIKIRRDKDMSMSSESDDMMTSKVGVDSPSVEEACGGGNTGGGLPLKKGPWTSA
EDAILVDYVTKHGEGNWNAVQKHSGLARCGKSCRLRWANHLRPDLKKGAFTPEEER
RIIELHAKMGNKWARMAAELPGRTDNEIKNYWNTRIKRRQRAGLPVYPPDICFQAISE
SKQNEDLGTFSTDGQYSDFLPVNNYEIPAVEFKKLEFNEPLCPPSLLDIPAGSLLDIPAR
SLLAQGLNSAYYSRSFLSTMHPSKRIRGSESLFSALNGDSSPLKNEDSFPTCHQYQND
GTLLAQSLGFSSPYNQNPTSDHHPSSLGVPGSHAPLNGNSSSSEPSWAKKLELPSLQSQ
MASWGLPPSPLPSLESVDTMIQSPPTEHTESCNLSPRNSGLLDAVLYESQTMRASKDN
LHQENSGDGADDSCPDLHETEWEAYGDPISPLGHSAASEKFDGKDDTLNPFFSRPDY
LLESNCFSPMQS

>Peaxi162Scf00540g00619.1

GASAFHPYQNLPKNNLHGLHPNLPLTAIDRFLLGHENYFSPLSGANATSSHANEVSWP

TLEPSFMELGYFLHEQSAINYMDDHQENIMIVEAESSQTNKKESVKKAKEGYSTTNLI
KGQWTDEEDRKLLRLVKQFGVRKWAQIAEKMEARGGKQCRERWHNHLRPDIKKDS
WSEEEELMLVEAHKQLGNRWAEIAKRIPGRTENSIKNHWNATKRRQNSRRNKNRKN
PQVGQNEGKHRSNVLQDYIRSKYFNDDSSSTTTSHKEFLHYQTNTITPTNSTTTNATP
SYSNDDSTSLLTHQTYDEELQFMQNLFGKNSILDNNGAKAIDHSMEDKVTQNLFDNN
NSSSSFSAFSSLDFSSDPLASETNITTKRVIHDAENYMNDYYKESTIHCPDLYLSYLLD
ASTYSSMPFAEASGYGSMNMGMLMNQGSCSSGNNKEVDFMEMASSNSQFSQGNFT
KPFF*
>Peaxi162Scf00543g00414.1
SFKEARSASKQSKGKMGRAPCCEKMGLKKGPWTPEEDQILVSYIEKNGHGNWRALP
KLAGLLRCGKSCRLRWTNYLRPDIKRGNFTREEEDTIIQLHEMLGNRWSAIAARLPGR
TDNEIKNVWHTHLKKRLKNYQPPQNSKRHSKNNHDSKAPSTSKMLDNSESFSTIQEN
INEPMTGPNSPQRSSSESSTVTADSLAATDVTNDDQTFIKHEEMDSYENFPEIDESFWT
EDLSMGDNLDLDMEVAGEKSQVQFPYSHDMKEQSVDMVGAKLEDDMDFWYNVFI
KAEDLLDLPEF*
>Peaxi162Scf00566g00051.1
MENFNRCSTSTSSSSESSSSESSLNKAERIKGPWSAEEDKILTKLVERYGARNWSLISK
YIKGRSGKSCRLRWCNQLSPNVQHRPFSHAEDETILAAHAKYGNRWATIARLLPGRT
DNAVKNHWNSTLKRRYQQQKNHNTVIFPDMKNGSGSGSGSGSGSRSGSGSCMDYL
NVDESPRGTNVKVVVNNCNSEYDDPMTELSLAPPGMGGDELPERKTESFSAGFWDV
MRDVIAKEVREYVASSFNGASTGFP*
>Peaxi162Scf00575g00016.1
MGRPPCCDKIGIKKGPWTPEEDIILVSYIQEHGPGNWRSVPTNTGNSVGFTIFYILCSSL
LYYFLEYCYNEAISLFLKGLMRCSKSCRLRWTNYLRPGIKRGNFTSHEEGMIIHLQAL
LGNKWAAIASYLPQRTDNDIKNYWNTHLKKKLKKFQATGLDSQSLAPSDSNTYQFS
WNFHDSKLQQNQNSSTSSSSTLYASSTENISRLLEGWMRSSPNPSTKNIDDEILHENDQ
NQEVYKNCESSWLHGGRNNTIGSTSIPKEYYKSVISEKSACDQSGGAVEKSCTNNAPP
YTYLEKWLLDESAGQVEELMELPTIFT*
>Peaxi162Scf00578g00007.1
MKTSVFTSSGVLRKGSWTEEEDILLRKCIEKYGEGKWHQVPVRAGLNRCRKSCRLR

WMNYLRPHIKRGDFSPDEVDLILRLHKLLGNRWSLIAGRLPGRTANDVKNYWNTNL
LTRAKLGPPQQHDRKCPKAIKTMAKNAIIRPQPWNLSKLAKNNVSTIHKEEHSKQEIII
EKPTTAEVVSRDENVEWWTNLLLDNSNGFEKAATESTSAFQNIESLLNEELLSPSING
GTYYPMQETGDMGWSDLSIDADLWELL*
>Peaxi162Scf00578g00008.1
MKTSVFTSSGVLRKGSWTEEEDILLRKCIEKYGEGKWHQVLVRAGLNRCRKSCRLR
WMNYLRPHIKRGDFSPDEVDLILRLHKLLGNRWSLIAGRLPGRTANDVKNYWNTNL
LTRSKFGPPQQHDRKCPKAIKTMAKNAIIRPQPWNLSKLAKNNVSTIHKDEHSKQEIII
EKPTTAEVVSRDENVEWWTNLLLDNSNGFEKEATESTSAFQNIESLLNEELLSPSINGG
TYYPMQETGDMGWSDLSIDADL*
>Peaxi162Scf00579g00055.1
MVRAPCCEKMGLKKGPWTPNEDQILISYIRKNGHGNWRALPKQAGLLRCGKSCRLR
WTNYLRPDIKRGNFTKEEEDTIIQSHEMLGNRLPGRTDNEIKNVWHTHLKKRLKNYK
PPHNTKRHIKNDDSKGPTTSESSIKSDFNSDTQKNINSPQHSSSEISTVTAESVVMTQSQ
AINDHDQMMIKHEEMESSEYFPEIDESFWTDELSTDNNCNHVVTAVPFSSVREENVD
MLTTRMEDDMDFWYNEFIKTDDLQELPEF*
>Peaxi162Scf00606g00073.1
MGRHSCCYKQKLRKGLWSPEEDEKLINHITKYGHGCWSSVPKLAGLQRCGKSCRLR
WINYLRPDLKRGTFSQEEENLIIELHAVLGNKWSQIAARLPGRTDNEIKNLWNSSIKKK
LRQRGIDPNIHKPLSEVEIDEKELANSKNNEKASEGSSDLNFIEVHENSNLRIATEKPKP
SASLVTMDRYPNGMSSAVPPTHEFFLNRFVTSHESSTATCKPLDLASYLSFQQLNYGS
NIGLSMNPNTNPLLFNSNSKNSEMVSHHQFNSNMPNDILPSISNSILTSPVAAAACSSNI
ELQRNSSFFDNNAFSWGAADHCGKSEKGANNIHSSESDTEDIKWSEYLHTQFLPGNT
NIHSHQTSIQHLYSEKSGTQFTTEVSLSTTPWLPNQQQQPSIQTADLYNKHFQRLPDAF
GQFS*
>Peaxi162Scf00658g00110.1
MNTTIPKSSGLVRKGAWTEEEDVLLRKCIEKFGEGKWHQVPVRAGLNRCRKSCRLR
WLNYLRPHIKRGDFSPDEVDLILRLHKLLGNRWSLIAGRLPGRTANDVKNYWNTHLQ
RKLIAPPRQEIRKCRALKITENNIVRPRPRTFSNNAQNISWCSNKSITNSTIDKDGSNNE
CIRISDKKPMAEVSRDDGVRWWTSLLANCNENDETAVENMSYDKLPSLLHEEISPTIN

GGISNCMQEGKTGWDDFSVDIDHLWNLLN*

>Peaxi162Scf00682g00009.1

MGRAPCCDKANVKRGPWSPEEDAKLKDFIHKYGTGGNWIALPQKAGLKRCGKSCR

LRWLNYLRPNIKHGDFSDEEDRIICSLYSTIGSRWSIIAAQLPGRTDNDIKNYWNTKLK

KKLMGLIPSSIIQRKSPYMFPPTTLHATQVQPNNLYTPNLSYTNNNMNFPLGATNHQY

SYNFQSHHQDSLINPMQIYPQLKDNLLMFGGTTEASCSSSDGSCSQLSFGHKEIKKEDI

IMGNFSHGQISSVAFEENQNFMSWVDQKPNGYFGNNNNQSADQVLLQYDLDQEVK

QQQLTSCSNGNNGTTTSECNNNSMFYY*

>Peaxi162Scf00683g00242.1

MDMNGATYISVRFLMGFEWVGSDADDDIELVRSIQERFALSTELREPLSLKPLYSIHPR

GGSEFDDDDDDGDDFETLRAIQRRFAAYTDERTNPEEGFPACVDRTTHFSEGCSYDIA

ARSQTLIEWHDDDSGAENTAVSANSSSFPKSAQAFVDAIKKNRSCQKMIRNKMMQV

EARMEELKKLKERVKILKSFQLTCRKRMGRALSQKRDARVQLISLPKQRFSSKLQGK

KLSAIHYGPPENSHVASFKEALTEFAVSLSRKEWSKEEREKLAKGVKQQFQEMLLQHS

VNLRSDQDGCSGESGDLDDTIASIRDLVNKIAPETMRLFLPKVNWDQVASMWLNWE

DPLIKHEGWDTVEEKSLLHAVQQKEMSDWIGIAASLGVCRTPFQCLSHYQRSLNASII

RRDWTEEEDIRLCSLVETFGESNWQVVASFMEGRTGTQCSNRWIKSLHPARKRSGKW

SADEDKRLKVAVMLFYPKTWRNIGQSVPWRTPIWKKIAQFVPGRTHVQCRERWVNS

LDPSLKLDEWTEEEDLKLKSAIDEHGYSWSKVAACVPPRTDNQCRRRWMVLFPDEV

PMLQEAKKIHREAFISNFVDREEQRPALKPDDLVPTHKLRDKAGCETTSANKKRKRR

PRAGREDTTPRSDAICELEKQHSDGEEGFEQDFHDASKNRRPSKLRVRKTQNFAPND

NVPDASASSQVESIIGDGNKCKRRKRTHLLMKKKAKNAALQESSSSFPNQSSSMTAA

EEADIRDSRKTNNIMDGRHSIRECDNQLKAIDNRPETTEDCTTLASFISKSRAKGRLLS

SAKVVKHPELGQSKTMADADNNSSCTTLASFISKSHAKRRSSSSAKVVKHPELGQSK

TMADNTSSSISGHDGLGKTMTEDCTSSNGVVGAEVGDDMPLSLFMSRAKRKR*

>Peaxi162Scf00689g00121.1

MDDDFSINSKNYLQDFEHLDLNFSIPTFNNPDHNFMIETNSYDPFDPFSNIEDDLGDFN

FCYERKPFEENIGASSSIVVNNIHGADEFSINKENFMDVNMYNEKSFSSVCCEDVKPL

NFVVPDESSSCVTADINGSCKEIGGRKSKKMKRNDNNLSSSMKKPSRGRKKSKSAKG

QWTIEEDRILIHLVEKFGIRKWSQIAQMLKGRIGKQSHAEVGNKWAEIAKRLPGRTEN

SIKNHWNATKRRQFSRRKCRTKWPRPSSLLQNYIKSLNLEKGSSKKNFHDTNPNAKV
MKVSTKLEPVDNFCQGNSIEQVHDYGFSEVQEFALDDKLFDDIPIGPSNMDDNIEKFM
GLEMIPYELSTLVQGEVNKEIDLMEMISRVNKP*
>Peaxi162Scf00700g00732.1
MGRPPCCDKTNVKRGPWTAEEDAKILAYVASHGIGNWTLVPQKAGLNRCGKSCRLR
WTNYLRPDLKHDNFTPQEEEIILEFHKTVGSRWSLIAKQLPGRTDNDVKNYWNTKLK
KKLVNMGIDPVTHKPFSQVFAEYGKICNLPIQNARNHIILSNNSTGKNQVFQEPNQFQ
FSNETPHDTIQTHSFSEKENPSCVQVTQAEEKKSVDSFVDNILARDKQMLLDYPPLLD
VYLDY*
>Peaxi162Scf00714g00029.1
MNQVLLRIYFGYKIMIQVKEEVQTLDFCGFASCSSFSDSSYEASTSRYSSEPGCRRSSG
PTKRSSQAGWTEEEDYLLSEVVKRYNGRNWKKIAECMSGRTDVQCLHRWQKVLNP
ELVKGPWTKEEDDVIVELVEKYGCKKWSLIAKSLPGRIGKQCRERWHNHLDPTIKRD
AWTEQEESVLLHYHQIYGNKWADIARFLPGRTDNAIKNHWNSSAKKRLSLNIQSEGS
PNFSSHEKKLEIGKYSLQAQSVEQTIFSGEQKGVDNAADACSTDLTIGCTYSAQNALR
KDTSLFGACKSSEENVRDLIKPLGGIQFGKAEIIPIGETDKPCHSNLNHTKILYPLSTSSL
EFPLDRSHITSLSSSQVEAVHPASFGRMYETPKRSRHDVNNPDQDFLSLSLPGFTEVHS
QGSKRNKSFDMQSSLDLNQHGCLYYEPPQVKDLMIPLTDENLSSDNIIGADHGPPFCS
TPPSLELTVSGNGSSSPESMLRNSAMSYTKTPSIIRKKTSKFSEAVRHSNCTGTTTPMY
SFLGDPDRGDSSNLKDRISGCKTSVSGKSLGRRLEYAFDMEWDASRCRTPVSAAPPC
GLNLGAIGANTIQIIRIDGSIRDFFDSCQFEATKISEAMTMLRLSSLE*
>Peaxi162Scf00753g00338.1
MSKGIVMNSCSHEDEFELRRGPWTLEEDNLLIHYISSHGEGRWNALAKCAGLKRTGK
SCRLRWLNYLKPDIKRGNLTPQEQLLILELHSKWGNRWSKIAQHLPGRTDNEIKNYW
RTRVQKQARQLKVDSNSKKFVEAIKSLWMPRLLEKMEQQCSSSISSPASSSSKISIEKQ
SSSLPSPLINHQEPYKSHDNTCNYDNEKTNSLEHPRIYTSSSMIQEEGYHVESFNQQPD
FSSQEMSISECEMGETNWFTDEMGGSLWNMDEFWQFRKLGDVDI*
>Peaxi162Scf00763g00441.1
MGRHSSFVKEKLRKGLWSPEEDEKLYNYITKFGVGCWSSVPKLAGLQRCGKSCRLR
WINYLRPDLKRGMFSQEEEDMIISLHQVLGNRTDNEIKNFWNSYLKKKLIKQGIDPNT

HQPLSENQVRNETDCKDKASTSTQIQQPFNLSKRNFNYEASRQLTEASKDQLVSKQV
FDPLFLYEFQANVNPSGYNLRPYDHNQNQIEGNTNFGFFSMPSLTNFEQGHMTETDFS
ESSSSRMSLLYISEAKESSSNSSNMISHHNAAGIQMNEMLENSQALSWDVDNKIDSLF
QYPYDGIKNEEDFNNPLSSLSEDLSGENLDVFHQI*
>Peaxi162Scf00777g00521.1
MGRPPCCDKVGVKKGPWTPEEDIMLVSYVQEHGPGNWRAVPTKTGLRRCSKSCRLR
WTNYLRPGIKRGNFTDQEEKMIIQLQALLGNKWAAIASYLPERTDNDIKNYWNTHLK
KKLKKLQETGDDLFSRENGYFSSSNSTSKGQWERTLQADINMAKQALHNALTLENLS
SCVKQESQVSTYASSTENIARLLKGWMGSSTNNSDYSKTSSNNVATADSSSCDGTPSA
ESEIGLMESFKSLFGFESFESSSSDQFSQTASPDASKLKGEIKKEANEQMPLSVMLENW
LLDENTIQGKDDLSNFSFDETNDLF*
>Peaxi162Scf00779g00512.1
MGRAPCCDKNGLKKGPWTPEEDQKLIDYIKEHGYGNWRTLPKNAGLQRCGKSCRL
RWTNYLRPDIKRGRFSFEEEETIIQLHSVLGNKWSAIAARLPGRTDNEIKNYWNTHIR
KRLLRMGIDPVTHSPRLDLLDISSILNYPALYNNSSHHQVNFSRLLGHVQPLVNPEVLR
LATSLLSSQRQNSNFLMPNNVQENQICISQVQNQVHPMVQVTSQVQNPIQNIPTCTTL
SITPSVPFYDEARLMEQFSSNLVNSSSQNCQVNEWQSSGMASNLTDQDYFNNPLQNY
GYYHELDQSIRDPPLSDASTFQSNDSNNFSFQSVLSNLSTPSSSPTPLNSNSTTTEDERD
NSYCSNMLNFDNIPNIWDSTNEFM*
>Peaxi162Scf00786g00439.1
MDQNLHHQPKIMHRCCSHEVVSFVLIWKTEVNSMEWEFISMTKQEEDLIYRMHKLV
GDRWGLIAGRIPGRTAEEIERFWIMRHSDGFAHKRRQLRKV*
>Peaxi162Scf00797g00013.1
MGKQPCCDNVGHNRGPWTVEEDHKLTNFILNNGIQNWRHVPKLAGLVRCGKSCRL
RWMNHLRPDLKKGAFTEDEEYMLIKLQSQLGNRWSKIAAHFPGRTDNEIKNHWNTR
IKKKLYTEQTRDIKEGSTKQKYSGQQSLPSLEYLVKDQQQLKEPIEISEMGSNDVNQN
SEPVETISIE
>Peaxi162Scf00840g00121.1
MGRTPCCDKNKVKRGQWSPDEDEILKNYLLKHGTGGNWITLPLRAGSLLSLSRHTR
QNYVIYYLIITQVYELFLLCRWSIIASKLPGRTDNDVKNHWNTKLKKKLLAKNANPTS

SFDESNEVFNTSTPNALLFMVPKEEEKQAPYQFPFPNMDSAGLSINTNNIDQKHFPIPS
FTQENSKNSGTSSVSFDETNWFEKGANDSFEGFPLGFSTDEIMDGIWSTQQSLFEEIDF
PSTFI*
>Peaxi162Scf00847g00211.1
MGRAPCCDKANVKKGPWSPEEDAKLKEYIEKSGTGGNWIALPHKAGLRRCGKSCRL
RWLNYLRPNIKHGEFSDEEDRVICTLYASIGSRWSIIAAQLPGRTDNDIKNYWNTKLK
KKLMGLVSTSQKIRPLHNQHQIITNYNYYPQIPFHQTSSLASSSPYSSTTTIPCYESTIPT
LASSSFLNTASASCTSGISGSTSGTVLQVQESYVGGPTTSSDGSYTNQMSQGRDQLEY
DYGVSNGGENLDFHNYLYNNGIIGLEDQSTSSKFLNIEAADEKPIINANSCYVQQEQN
PLDYSLEEIKQLISTNNVTCNSNFFLDENKIEEKVMMYY
>Peaxi162Scf00876g00221.1
MGRKPCCSKVGMRKGAWTAAEDRLLTNYIELNGEGNWRSLPMKAGLLRCGKSCRL
RWVNYLRPGIKRGNFSPEEDDLIIRLHTLLGGRWSLIAGRLSGRTDNEIKNYWNTYLL
KKLKAAGIEPKAKTKKYSTNTRQNKEMKRRRAKNKTDNLLHQAEKPRGKKEDCEV
VEKIQVHRPKAIRLTKGYSSSTSSSFSSSSSQSSCQDNAPKFNFPLSSEEVDNKKEEKIE
DNAIYEKLQLFDKLLNGCDISTECSEQTSSCCYMLDE
>Peaxi162Scf00886g00028.1
MVRAPCCEKVGIKRGRWTAEEDELLLKYIQANGEGSWRSLPKNAGLLRCGKSCRLR
WTNYLRPNLKRGKFTSEEDETIFKLQCSLGNRWSLMASYLPGRTDNEIKNYWNSHLR
RRIYTFGMKKKPIKTAAEMPNKTIVADGLNCESLKKRGRVSRSKAKKYNNNTTTTTT
AYISTLKPKSSGVGAGGGAICSEGDSIVDAGIGLDIQQHDEDHAGSAIGKPRNEETEGT
NQKHINATAEKQEVRNGILSFEEQGQQVLDEHILIGPHEKNVGDETVHLQQPNYCLH
DFGNQVSLSGVLEVDEESHENWWSTMNSDNFLEDELWVDQCSSLDLEFGSIEECDD
MLLPLWDDN*
>Peaxi162Scf00911g00012.1
MGRSPCCEKLGLKKGPWTPEEDQKLMACIEKHGCGSWRALPAKAGLKRCGKSCRLR
WINYLRPDIKRGKFSLQEEQTIIQLHALLGNRTDNEIKNYWNTHLRKRLTKMGIDPITH
KPKTNIFGSSQLSHMAQWENARLEAEARLVRESKKQISNNNIHDFGSLANTRLAHHQ
LFNQRSRINNIVPLQAKVPPFLDVLKVWQDSNWTKPKITKDTSSSSVLDSVLTSMNNG
SFDSPTSTNNLFMAPNNNIPRLVDNSCLVNAGSFMENPEVEIITKEEVQTDQVQINDIS

SINYMSITDQSSGFPSFIQGFTHLENIVGSSEDNLEDNKYSYWNTILNSCTSPAGSPVF*

>Peaxi162Scf00929g00071.1

MEGSSSTCSSSSSESSLSAGTPKTPRDGNNNKPERIKGPWSAEEDKILTRFVERYGAKN

WSLISKYIKGRSGKSCRLRWCNQLSPEVEHRPFSPAEDDTILAAHAKYGNRWATIARL

LHGRTDNAVKNHWNSTLKRRQQIRNNVSVVESPIIIIRRNDIIVNVNNWDEVDPMTTL

SLAPPGCRRRVCRWSFGM*

>Peaxi162Scf00944g00140.1

MGRAPCCDKTKVKRGPWSPDEDNTLKNFVEKNGTGGNWIALPQKADINHGAFTEEE

DNIILTLYSQIGSRWSVIAANLQGRTDNDVKNHWNTKLKKKLLAAAEITNISSNYNSV

LNHNYCATESTCFISNTLTSLVEPNLTMSSNLVPAMLNVETSCQFPLPGLMEIQENGAIT

TLQEDGNYLMDFSSGISSTYYDILNGFDFQEKVITADPNCSSSSCSTLVQQQGYIPDID

QYPFKGVEN*

>Peaxi162Scf00945g00023.1

MDIITKKGKHIDRVKGPWSPEEDELLTQLVNKHGPRNWSLISKSIPGRSGKSCRLRWC

NQLSPQVEHRAFTIEEDETIIRAHARFGNKWATIARLLNGRTDNAIKNHWNSTLKRKC

SSLSADEGNQLADQIFQNDNQPPLKRSVSAGSALPVSGFNFSPGSPSGSDSDSSFHVSS

SSQCCVFKPVPRTAGVLPPGLACPMDTSSSPEAGDPPTLLSLALPGVDSAEHSAESTQ

VKALLLPQATQIPPPPPPLQAVPIQHGEQQDKVFVPFSQELLGVMQEMIKTEVRNYM

MGIEQHHPQQQRQQQQQQQQYHQQQNHQLPNGIGLGLCIQQATDGFRNTAANRI*

>Peaxi162Scf00978g00214.1

MTSECVDRMTSKVGVDSSAVEEASGGGNAGGGVPLKKGPWTKAEDAILVDYITRHG

EGNWSAVRKHSGLARCGKSCRLRWANHLRPDLRKGAFTPEEERLIIELHAKMGNKW

ARMAAELPGRTDNEIKNYWNTRIKRQQRAGMPMYPPDICYQAFRENKQNKELGTFS

SADSHPDFSPINSFEIPAVEFKTLELSQQRYPPALLDIAANSSLDIPATSLLAQGLNFSCN

TRSFLSTMHPSKRIRGLESWFSGLNDDFFQACHQFENDGSLFAESLGYSSPHTDNLISV

HHPAFSGVFNGSHASLNGNSSSEPKWAKKLELPSLQTQMASWGSPSPLPSLESYDTLI

QLPPSEHIDSGSLSPGNSGLLDAVLYESQAFRASKNNLHQETCDVVDDSCPDVQATW

GTHSGPNSPLGHSAASVFSEYTPIFGGSLHEPRSVASLFGENGCKIKQEDVDLAPTDRN

NDVSNHQIFAWTESCFAPTVLVPQQNRVLCLTLLPFVSPPSESWAQTLLDFPPPLLQSSS

QPATILRSSD

>Peaxi162Scf00986g00227.1

MVQEEIIRRGPWTEQEDFQLVFYVNLFGDRRWDFLAKVSGLKRTGKSCRLRWVNYL
NPGLKRGKMTPQEERLILELHSKYGNRWSKIARKLPGRTDNEIKNYWRTHMRKQAQ
DQRKNAFISPSSSFSNCSSNSSANSPAVDSIPITKQNKRNLETAEEKKIYNHEEAEENQN
MVYTMDEIWKNVESSQETETMSKLPVMASPIWDYCPDSLWMTDFNYFDNQDIPFFT
G*

>Peaxi162Scf01007g00117.1

MSEQMDSWGMTKQGWRKGPWTSEEDRLLIEYVNLHGEGRWNSVARVAGLKRNGK
SCRLRWVNYLRPDLKKGQITPHEEKLILELHARYGNRWSTIARNLPGRTDNEIKNYW
RTHFKKKTKNSRDKSEKSKVRLVKRQQWQQQKQQQKLNTQTDSNRVVSLNNKKNY
YASILNKLGNKIVSLLDKNENRILPLVTQKENQEMANLYSNTADQEEDDFLNSILNDY
TCVSLLESSFNEDMMWDDFWNNLDEYQCNVSTTI*

>Peaxi162Scf01010g00010.1

MEEAEQQSDFKLIDAAVNGGDTMAVDENNGGGGGGGKVKGPWSQDEDTVLSELVS
KFGARNWSLIARGIPGRSGKSCRLRWCNQLDPSVKRKPFTEEEDNIIIQAHAVHGNRW
ASIAKLLPGRTDNAIKNHWNSTLRRRFAGLKRVNPSHFEMLDSSNIDRVKATSEETRS
GDDHNQSKSLEGREASVQQCKRTLLEDRAPVSEMFGVPEKVAHLVSGSCHSTINENN
PSVSRPTAKVGAFDVHNLSSASFASSRTVPIQGNLLQAFSPDFGICKLLEGLPDEPIIPSS
CGHGCCSAHSKSSSASSLLGPEFVEYEDLPAVSSHELFAMATDLNNIAWIKSGLENAG
KLPDKTTSGRDHQGSSTSTPMPMPMTSFVLPAQVESLS*

>Peaxi162Scf01068g00314.1

MRLLRFCVLASCDFQEMNFLTQNSLVMDYNKGRHCLSNGSTDGKVLENPWIFHSMV
EDENGTKLNVETEEVDLVGRKNNGGTKLCARGHWRPHEDAKLKELVAQYGPQNWN
LIAEKLEGRSGKSCRLRWFNQLDPRINRRAFSEEEEERLLTAHKMYGNKWAMIARLFP
GRTDNAVKNHWHVIMARKHREQNSVYRRRKPSNMQQFHSMGFPHVSGSDNAIQSN
NLNSDSTISSTTNNMDEHCASTCTDLSLTPSSSKVIFMKRLTHMQHHHHPLLEASKGF
SRDAQEVKMESGVHPKFLHGKEPRADTVDASHQYGCGGSDTNSDISASESVANNMT
NVKMYGQNHENVKLVEKQMKSKTMPAFIDFLGVGAT*

>Peaxi162Scf01101g00048.1

MGRSPCCDETGLKKGPWTPEEDQKLINYIKKHGHGSWRALPKLAGLNRCGKSCRLR

WTNYLRPDIKRGKFSQEEEQTILNLHAILGNKWSAIATHLPGRTDNEIKNFWNTHLKK
KLIQMGYDPMTHRPRTDIFNSLPHLIALANLKELVEQSEVARIKYLQYLLQPSMAANL
ANSMSTSNTCSVANISDMEAYNNLLNIGTNHLENSTNNIPPPSTTLQAIQDSITFSHLPE
LQATPNCNFQNSLNEENMVQTHVISQGENTPTSPWQLPSSLSPPENDNQPINSYINNIG
GSCNPPFFGGAHPSVWPEILLEDSLFQDIA*
>Peaxi162Scf01194g00013.1
MDHQHAKVDLRSKEAACSTNNIIQKVEEDDMDFKRGSWTVEEDFTLINHIALHGEG
RWNSLARSSGLKRTGKSCRLRWLNYLRPDVRRGNITLEEQLLILELHSRWGNRWSKI
AQHLPGRTDNEIKNYWRTRVQKHAKQLKCDVNSKQFKDTLRYLWMPRLVERIQASN
SSKNQVIQQTSNNNISNNIIPSMSFTQENSSTTTSSENSLGTQVISQVSDTSDCCYNYSTI
NQSDTLCYGESLTSPTGYFHQEGALDFRTVDIDDNQQTNQFLDVSDNLWNIEDMWFL
QEQLN*
>Peaxi162Scf01210g00002.1
MNTSVFTSSGVLRKGAWAEEEDILLRKCIEKYGEGKWHQVPVRAGLNRCRKSCRLR
WLNYLRPHIKRGDFCPEEVDLIQRLHKLLGNRWSLIAGRLPGRTANDVKNYWNTHLL
RRSNFASPPQQHERKCTKEIRTMAKNAIIRPQPRNLSKLAKNNVSNHSTIHKDEYSKQ
KMFIEKPTMAEVVSRDNNVEWWTNLLLDNCNGFEKAAPESSSTFKNIESLLNEELLS
ASINGGTNYPMQETGDMGWSDFCIDSDPWELLLQ*
>Peaxi162Scf01221g00042.1
MGRSPCCEKAHTNKGAWTKEEDERLIAYIKAHGEGCWRSLPKAAGLLRCGKSCRLR
WINYLRPDLKRGNFTEDEDELIIKLHSLLGNKWSLIAGRLPGRTDNEIKNYWNTHIRR
KLLSRGIDPSTHRIMNEPSTQKVTTISFAAGNEDIKDQKISIKAEFEQIKDDEIISKPIKEQ
CPDLNLELKISPPYQQHSDRALQQSTTGSGGASTICFTCSLGLKNNKGCSCSRNRSMN
VAGYDFLGLKTNGLDYRTLETRTK*
>Peaxi162Scf01251g00013.1
MGRTPCCEKLGLNRGPWSKKEDDILINYIDKHGHPNWRQLPKLAAFTWSVIAAKLPG
RTDNEIKNIWHTRLKKKMNQSQIQEKTPDIREQPSETSKSEDSTNIQENTEISSPKPNSN
NQHEPSSSLRSSSSITSSSEGSCSNTTTTSSHIDESRDQMNLENLLEVDDNFWSEVLWA
TPADDSKDNNLDFSLSSVEKNYELDSSLNDNWLWDDLFSRANEFLLEFPELV*
>Peaxi162Scf01256g00017.1

MGRPPCCDKIGVKKGPWTPEEDIILVSYIQEHGPGNWRAVPTNTGLLRCSKSCRLRWT
NYLRPGIKRGNFTEHEEKMIIHLQALLGNRWAAIASYLPQRTDNDIKNYWNTHLQKK
LKKLQGHDDQNNQKGKSQSISKGQWERRLQTDIHTAKQALVDALSLDKKTTNVVSP
NINSSANSLPVGQTSSYASSAENISRLLQNWMKNSPKSSQTTSNSTTSQSSFNNLSMVS
SSSPSEVTMSATTPEGLDSLFSFNSSYNNSDVSQSMLTDEVVAGIFQDGSKQNWQNFK
DESGIFQKGSKQNMETQVPLTLLENWLFDDANAQTQEELMGIGVGMEWP*
>Peaxi162Scf01302g00033.1
MEIHSLEILETSNPVEGGSYGGDSGGEDEMLENESGSSENVVKKNKRTSGERVKGPW
SPEEDAILSKLVSNFGARNWSLIARGIPGRSGKSCRLRWCNQLDPSVKRKPFSDEEDR
LILQAHAIHGNKWASIARLLPGRTDNAIKNHWNSTLRRRHNGKLRSDPSNVLEDVSA
EKSKASSEETQSCGDVNSLKAIEGKDVSSQENQEDNHHEDKSEAEVQRSDAANDPPT
LVRPVARISAFSVYGSLDVPEAQIQTPRLTPVQAPDLGISKLLEGCFTDRLVPHQCGHG
CCGNASQENNGSSLLGPEFVDYAEPPSFSSHELAALATEISNVAWCKSGLENSNIEVIC
NPTTRATSGTSLLPNRPF*
>Peaxi162Scf01314g00012.1
MGRAPCCDRSSVKKGPWSPEEDAKLKAYIEQNGTGGNWIALPQKIGLKRCGKSCRL
RWLNYLRPNVKHGGFSEEEDRIICSLYIILQPIPYSDYEPRLNGASIRKLLIKLGGKFSE
DDQPISGEQDPQYPMNNSIISMHSIYENPINLISSPLDVLNAQYNMNHGTALTTSPLQG
NFTAERMICNINSQKLGDLEFFI*
>Peaxi162Scf01390g00030.1
MCTSGITGLHKGALITLGSIVAIAAGFMTVITKLENSDVCLAYLPFAHVFELATETVILT
VRIQGYVSDGTVKKVETKGVSAKNRIETKFGAVLEDTTDITERSMVIGDEKVGIPRKE
KKKSKKNNKNADISGLGMTMADAAETRKDSVKAESETKPTGGSVNHDIKKKKKKK
REDGTHPDKSNKDSDESAAKCVEKKKHKKLKRSHQDEQQIILDAPINTKSVAYASAR
ERGDSENVIKILGEVSGGNAVEEIRRKKKSKKSNKHAVEGDLSSEEKKLCVVKGKKE
RDKVNMETKAAGESDVCDVKKNKEREGEKHPGKSTKDSDKDDLGSVEKKKSKKLK
KSCRDESETMHGIEDEQEAIVAKVNRGDISSTVEEIEEQSRTDTGKIRKKKKATIGHSSE
DPTHEKSEKKVRFSGHVEIFPSSSDPSDEKHETEEENLLRGKRFSTLEDEIVKEAVHKY
IEMHNLGEEGLEMILNSRSHPELKGCWKEIGSAIPYRPYTAIYYRAQVLFRRSEKRKW
TEEEYEMVRKYHEVHGNKWKDLADELGKHRFHVKDTWRRLKLTNRNKGQWTQEE

YQTLFDLVNTDLRLKLSEEKKSKHGMLRDNIAWTAISGNLSTRTDANCCLKWYDQLT
SPMVSKGEWADTDDYRLIDALFELDASCIEDVDWDNLVDHRPGEICRKRWNQMVLH
IGQYVNKSFSEQVEVLAKRYRPELVEVREAWDSKPIVYYCRSKAGNASGGSDIIEELT
VDQEINQTATETRDEGFDSVLLTKHKQKNVTESTKLWPSAAISSSGKDNNCSHSEWLT
HSALRINAGGHRYLPSCSHPFVADVQPSSCPAMISIFLILAGFLASGGFGATATFILIKPS
FARLVKN*

>Peaxi162Scf01464g00024.1

MGCKIVEKTKQKHKKGLWSPDEDDKLRNYIINHGHGCWSSVPINAGLQRNGKSCRL
RWINYLRPGLKRGAFSLEEEDTILTLHAMFGNKWSQIAQHLPGRTDNEIKNHWHSYL
KKRVAKNAENESQTQSGNRESLLFSKKWTPQNSSLDSFEHIEGSLADSDQSGSQVDFP
KEHQKSNLPKVLFAEWLCLDKFHGQDFQNSGTFDLTKNNFGYNNSESQHAFMHGLL
MNEGSYGSCMNQEVNNGTVDDMFQPQLKFEDSMSANGFEDFMSGEFINTDVMYI*

>Peaxi162Scf01819g00004.1

MEGGSGGDGSAYQPYQNIPTNLSIFQQGPPLIAINRYLLSHQSQFSSQQSVNNQSIFSQ
CGGLCDFSSYSGISHANGISWSSVPEPSFVDRIFLSHEQNGLHWNNQENGIAEEMISSQ
QNSKEAGKKAKEGPSSTTVLVKGQWTEEEDRKLVKLVKQFGMRRWAQIAENMVGR
AGKQCRERWHNHLRPDIKKDAWSEQEELILVEAHQQIGNKWAEIAKKIPGRTENSIKN
HWNATKRRQHSRRNKFKKQEKDGQNDSKYRSNILRDYIRSKYFSDNSPPTASTPSNSI
STNATPPYSDDDSPSLLTRQTYDEEMNFMQNLFGENSLVENNGKVAEAMEAKTRQCP
FDNKSSSSSCPFNSLVAGYTKSDNQIVNDNSEYGYNMYMADHYSKVTQQSDSDQCS
GIYLSYLLDHSTSANSLPCSGSIGYGNMNAGTLVNQGSSSSKGNKEVDLMEIVSSTLY
RQQTSHTTFFN*

>Peaxi162Scf01969g00021.1

MRCKPVESPKKKHRKGLWSPDEDHKLKNYILEHGHGCWSSVPINAGLQRNGKSCRL
RWINYLRPGLKRGAFSIEEEETIMTLHGMIGNKWSQMAQHLPGRTDNEIKNHWHSYL
KKRVAKIAENEAKSQNMDGLSSSSLKLTSRNSSLDSFAQIEGSLTDTDQSISQIEFLKEP
RESNFQKVLFAEWFTLDQFKSQDFQNSGNSNHSRNNNFGYNKSDFQDGFIHGLFMSE
GTYGADINIPGVLNNETVDDMFQASQFKFEDHMSANGFEEFISDEFNITEDVMYI*

>Peaxi162Scf03779g00019.1

MRKACCDNKEEMHRGAWSKQEDQKLIDYITKHGAGCWRNLPKAAGLLRCGKSCRL

RWMNYLSPNLKRGNFSEDEEDLIIKLHALLGNRWSLIAGRLPGRTDNEVKNYWNSHL
RRKLIKMGIDPKNHRISHYLHRKRLEYWSENSSRGTDHEVVSDAGSSCAKHQPSSLP
DLNSPPSIHSSCAQP*
>Peaxi162Scf03974g00003.1
MGRIPCCEKENVKRGQWTPEEDHKLSSYIAQHGTRNWRLIPKHAGLQRCGKSCRLR
WTNYLRPDLKHGQFSEAEEQTIVTLHSVLGNRWSVIAAQLPGRTDNDVKNHWNTKL
KKKLSGMGIDPVTHKPFSHLISEIATNLAPPQVPHLAEAALGCFKDEMLHLLTKKRIGF
QFQQFGTSTAPSTSTVKVEDNKEETIEKIKYGLSRAIKESDMLPSNKHWDPSGGARST
NLAEPSSGFPHVSDGGFQYNFASLLHEDAAEGSPWNQSLCTGSTCTVGEQQQVHQLH
KKLNSNDNCGEDNSDGAKETRNGSTTMFHSDCILWDISSDDLLNPMV*
>Peaxi162Scf25743g00028.1
MHFLNRMQAASRMWIGTIFLITGLESYVGNEMVRQLSGASTIEEIEDSITDNGNIRKR
KKTKLRHNYEDLPHQKSDDRVRFSCNVYPPSRDTIDEKHEIEEESNVDIVTGVNRGVV
STIEEIEDSKIDDATIRKRKKTKLGHNSEDLTLEKGEKRVRFSGDVQVFPPLSYSTDEN
HEVKDGSLLRGKRFTSEEDEIINDAVYRYIEVHNLVRTCWKTIGRAIPYRPYRAVYNH
AQLFFHRGEKHKWTEEEFEMIQKFHGEHGPKWRVLGDKLGKYWVHVGNAWQKIKL
ANRNKGSWTQEETHPDFISKHGMLRGNICWSAISDRLSTRIKQHCCNKWYKQLTSPM
VPTGEWADTDDYA*
