# Supplementary figures and images for: Genome-Wide Identification, Classification and Expression Analysis of the MYB Transcription Factor Family in Petunia
Source: Int J Mol Sci. 2021 May 3;22(9):4838. doi: 10.3390/ijms22094838 (PMC8124715; doi:10.3390/ijms22094838)

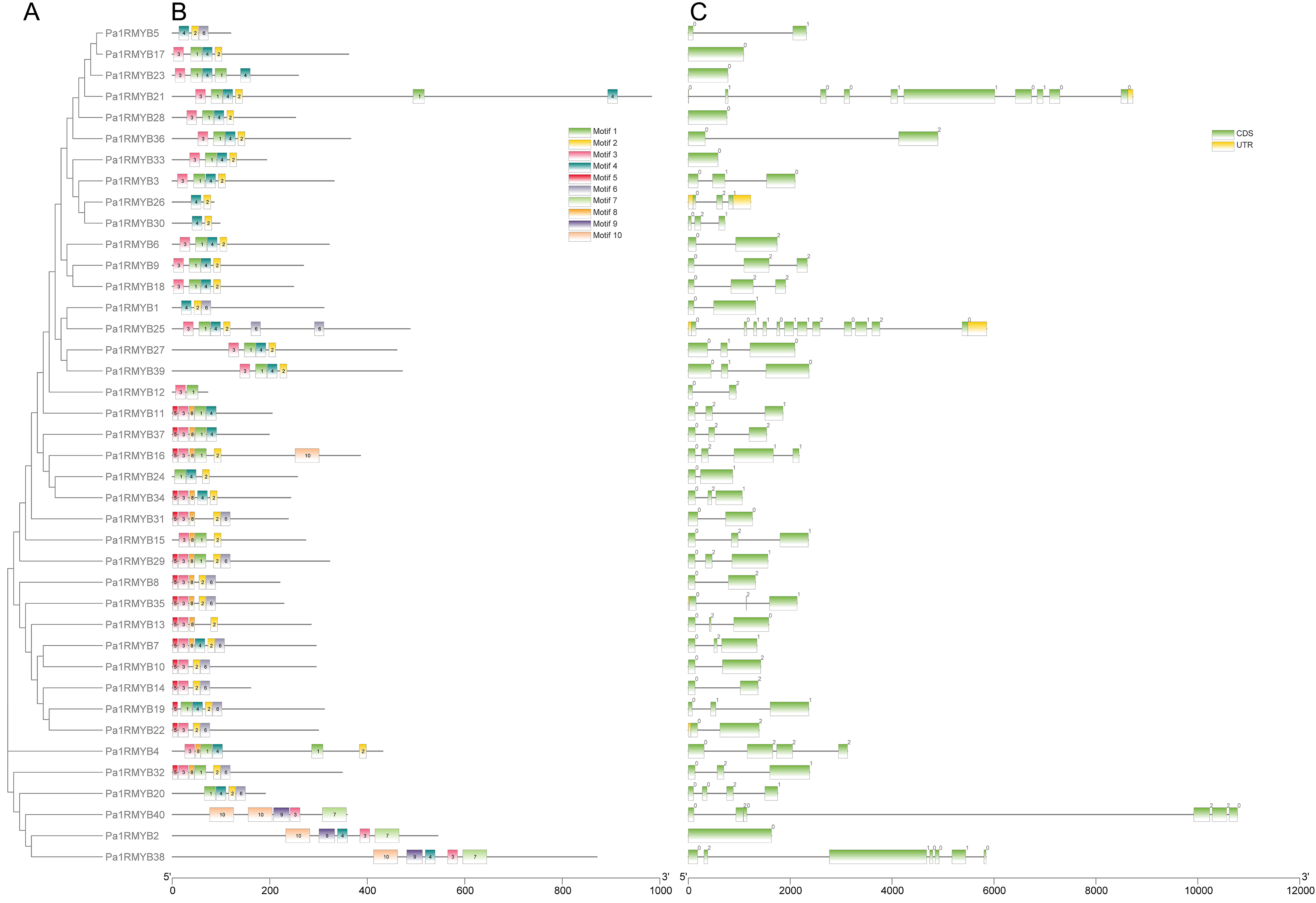

Supplement: Supplementary file 1 [file ijms-22-04838-s001.zip › supplementary files/supplementary Figure S1.tif]
